# Genomic Analysis and Surveillance of Respiratory Syncytial Virus Using Wastewater-Based Epidemiology

**DOI:** 10.1093/infdis/jiae205

**Published:** 2024-04-18

**Authors:** Danielle M Allen, Marina I Reyne, Pearce Allingham, Ashley Levickas, Stephen H Bell, Jonathan Lock, Jonathon D Coey, Stephen Carson, Andrew J Lee, Cormac McSparron, Behnam Firoozi Nejad, James McKenna, Mark Shannon, Kathy Li, Tanya Curran, Lindsay J Broadbent, Damian G Downey, Ultan F Power, Helen E Groves, Jennifer M McKinley, John W McGrath, Connor G G Bamford, Deirdre F Gilpin

**Affiliations:** School of Biological Sciences, Queen’s University Belfast, Belfast, United Kingdom; School of Biological Sciences, Queen’s University Belfast, Belfast, United Kingdom; School of Biological Sciences, Queen’s University Belfast, Belfast, United Kingdom; School of Biological Sciences, Queen’s University Belfast, Belfast, United Kingdom; School of Biological Sciences, Queen’s University Belfast, Belfast, United Kingdom; School of Biological Sciences, Queen’s University Belfast, Belfast, United Kingdom; School of Biological Sciences, Queen’s University Belfast, Belfast, United Kingdom; School of Biological Sciences, Queen’s University Belfast, Belfast, United Kingdom; School of Biological Sciences, Queen’s University Belfast, Belfast, United Kingdom; Geography, Archaeology and Palaeoecology, School of Natural and Built Environment, Queen's University Belfast, Belfast, United Kingdom; Geography, Archaeology and Palaeoecology, School of Natural and Built Environment, Queen's University Belfast, Belfast, United Kingdom; Regional Virus Laboratory (RVL), Belfast Health and Social Care Trust (BHSCT), Royal Victoria Hospital, Belfast, United Kingdom; Regional Virus Laboratory (RVL), Belfast Health and Social Care Trust (BHSCT), Royal Victoria Hospital, Belfast, United Kingdom; Regional Virus Laboratory (RVL), Belfast Health and Social Care Trust (BHSCT), Royal Victoria Hospital, Belfast, United Kingdom; Regional Virus Laboratory (RVL), Belfast Health and Social Care Trust (BHSCT), Royal Victoria Hospital, Belfast, United Kingdom; Section of Virology, School of Biosciences and Medicine, Faculty of Health and Medical Sciences, University of Surrey, Guildford, United Kingdom; School of Medicine, Dentistry and Biomedical Sciences, Wellcome-Wolfson Institute for Experimental Medicine (WWIEM), Queen's University Belfast, Belfast, United Kingdom; School of Medicine, Dentistry and Biomedical Sciences, Wellcome-Wolfson Institute for Experimental Medicine (WWIEM), Queen's University Belfast, Belfast, United Kingdom; School of Medicine, Dentistry and Biomedical Sciences, Wellcome-Wolfson Institute for Experimental Medicine (WWIEM), Queen's University Belfast, Belfast, United Kingdom; Geography, Archaeology and Palaeoecology, School of Natural and Built Environment, Queen's University Belfast, Belfast, United Kingdom; School of Biological Sciences, Queen’s University Belfast, Belfast, United Kingdom; School of Biological Sciences, Queen’s University Belfast, Belfast, United Kingdom; School of Pharmacy, Queen's University Belfast, Belfast, United Kingdom

**Keywords:** wastewater, respiratory syncytial virus, RSV, surveillance, genome

## Abstract

Respiratory syncytial virus (RSV) causes severe infections in infants, immunocompromised or elderly individuals resulting in annual epidemics of respiratory disease. Currently, limited clinical surveillance and the lack of predictable seasonal dynamics limit the public health response. Wastewater-based epidemiology (WBE) has recently been used globally as a key metric in determining prevalence of severe acute respiratory syndrome coronavirus 2 in the community, but its application to other respiratory viruses is limited. In this study, we present an integrated genomic WBE approach, applying reverse-transcription quantitative polymerase chain reaction and partial G-gene sequencing to track RSV levels and variants in the community. We report increasing detection of RSV in wastewater concomitant with increasing numbers of positive clinical cases. Analysis of wastewater-derived RSV sequences permitted identification of distinct circulating lineages within and between seasons. Altogether, our genomic WBE platform has the potential to complement ongoing global surveillance and aid the management of RSV by informing the timely deployment of pharmaceutical and nonpharmaceutical interventions.

## Teaser

The genomic wastewater-based epidemiology platform has the potential to complement ongoing global surveillance efforts and aid the management of respiratory syncytial virus by informing the timely deployment of pharmaceutical and nonpharmaceutical interventions.

Respiratory syncytial virus (RSV) a major cause of respiratory illness in humans, imposes substantial burdens on healthcare systems worldwide [1]. Globally, RSV is estimated to cause approximately 3.6 million hospitalizations annually, which in 2019 resulted in approximately 127 000 deaths in children <5 years old and approximately 14 000 deaths in adults aged ≥65 years [2, 3]. Disease management in the United Kingdom is currently limited to prevention, symptomatic relief, and short-term protection for high-risk infants by administration of monthly immunoprophylaxis [1]. However, the introduction of nirsevimab, a long-acting monoclonal antibody, and several promising vaccine candidates for older adults and pregnant women could protect individuals from RSV-related hospitalization during the RSV season [4, 5].

RSV typically causes seasonal epidemics between October and March in the northern hemisphere [6]. Clinical disease surveillance usually relies on the identification of infections in individuals with severe symptoms and/or comorbid conditions where hospitalization is required [7]. During the coronavirus disease 2019 (COVID-19) pandemic, RSV seasonal occurrence changed dramatically, with little or no evidence of RSV circulation in the first year of the pandemic, followed by a surge in off-season outbreaks across the globe [8], presumably due to imposed nonpharmaceutical interventions in response to the global pandemic [9]. Limited clinical surveillance and lack of predictable seasonal dynamics limits the public health response, affecting clinical resource planning and allocation.

Furthermore, unlike with pathogens such as severe acute respiratory syndrome coronavirus 2 (SARS-CoV-2) and influenza A virus, genomic RSV surveillance is either lacking or typically focuses on pediatric clinical samples [10]. With the expected introduction of new immunoprophylaxis and vaccines, global monitoring of RSV molecular epidemiology could play a vital role in identifying antigenic variations and potential immune escape mutations and tracking the effectiveness of available prophylactics, including emerging resistance [4, 8, 10, 11]. Therefore, establishing a robust surveillance system to rapidly determine the onset of the RSV season, the genotypes of circulating variants, and both community spread and the prevalence of RSV between seasons could support public health decision making regarding the initiation of immunoprophylaxis at the onset of the season.

Wastewater-based epidemiology (WBE) gained popularity as a public health surveillance system during the COVID-19 pandemic by supporting governmental policies and promoting quicker actions to prevent and control the spread of SARS-CoV-2 [12, 13]. Many studies have shown that wastewater surveillance can identify the magnitude, distribution, and community-level prevalence of SARS-CoV-2, irrespective of whether or individuals are symptomatic [12, 14]. Indeed, detection and quantification of SARS-CoV-2 RNA concentrations were significantly correlated with clinical cases and hospitalizations within the same wastewater catchment area, while sequence analysis facilitated tracking of variants and identification of cryptic lineages [15, 16]. Further research has shown that other viruses can be sequenced from wastewater, including influenza A virus, adenovirus, rotavirus and norovirus [17–19].

Several studies have detected RSV in wastewater settled solids [20, 21] and the wastewater liquid fraction [22, 23]. Furthermore, positive correlation between wastewater RSV concentrations and clinical cases has been reported [21, 23]. In this article, we describe the implementation of a WBE approach for RSV surveillance, based in Northern Ireland (NI), to track the community spread of RSV during 2 successive seasons (2021 and 2022). RSV levels in wastewater were correlated with RSV-positive clinical cases. Using a sequence-based approach and phylogenetics, we compared wastewater-derived RSV A and B G-gene sequences and linked clinical sequences to infer transmission dynamics within and between seasons.

## METHODS

### Sample Collection

Between August 2021 and February 2023, composite samples of primary untreated influent were collected weekly (n = 83 weeks) from 20 wastewater treatment works (WWTWs) across NI, covering 57% of the population (Supplementary Figure 1 and Supplementary Table 1). An Isco Glacier autosampler was used to collect 200 mL of sample every 15 minutes over a 24-hour period. This study was conducted using environmental samples and publicly available data. Under UK law, there is no requirement for ethical approval for these samples or data.

### Viral Concentration, Extraction and Reverse-Transcription Quantitative Polymerase Chain Reaction

For RSV detection, 50 mL of wastewater sample (n = 587) with 0.005% Tween 20 (Merck) was centrifuged at 4000 rpm for 10 minutes at 4°C before subsequent concentration of the supernatant using a concentrating pipette (CP) Select with hollow fiber-based ultrafiltration tips (InnovaPrep). Ultrafiltration tips were purged twice using an elution buffer containing 0.075% Tween 20 in 25 mmol/L Tris buffer. Nucleic acid was extracted using a MagNA Pure 96 DNA and Viral NA Small Volume Kit (Roche Diagnostic). Extracted nucleic acid was stored at −80°C. RSV RNA concentrations were tested retrospectively for the 2021 season and real time for the 2022 season. Reverse-transcription quantitative polymerase chain reaction (PCR) was performed on the QuantStudio 7 Real-Time PCR System (Thermo Fisher Scientific) in triplicate, using an RSV A and B N-gene assay described elsewhere (protocol S1) [21]. RSV RNA concentrations were normalized using wastewater flow rate [12] and expressed as gene copies (gc) per 100 000 population equivalents (pe) per day [19].

### Statistical Analysis

To reduce variability in the wastewater measurements, a B-spline regression model was fitted to the averaged RSV concentration (in gc per 100 000 pe per day) across 20 WWTWs (protocol S4; Supplementary Table 2 and Supplementary Figure 4) [24]. The numbers of positive RSV clinical cases per week for NI between August 2021 and February 2023 were provided by the Regional Virus Laboratory (RVL), Belfast Health and Social Care Trust. The relationship between RSV concentration in wastewater and clinical cases was investigated using linear regression. Additional analyses were performed to determine whether wastewater can be used to predict the onset of the RSV season by defining lead times (protocol S5 and Supplementary Table 3). All analyses and data visualization were performed using R software, version 4.2.2 (R Core Team 2021), unless otherwise stated.

### Amplicon Sequencing of RSV G Gene

Positive wastewater samples (RSV A, n = 281; RSV B, n = 233) and clinical samples (n = 6) from October 2022 (provided by the RVL) were sequenced. Extracted nucleic acid was reverse-transcribed using LunaScript RT SuperMix Kit (New England BioLabs), following the manufacturer's instructions. Complementary DNA was amplified in 2 subsequent PCR reactions (external and seminested) targeting the second half of the hypervariable G gene (Supplementary Table) [25]. The reaction and thermocycling conditions used are shown in Supplementary Tables 5 and 6. Amplicons were analyzed using the TapeStation D1000 assay (Agilent), and samples with the appropriate fragment size (Supplementary Table 4) were sequenced. Libraries were prepared following the minimized Nextera XT library preparation protocol [26] and sequenced on an Illumina MiSeq instrument using a MiSeq v2 300-cycle reagent kit. Reads were preprocessed by removing adaptors and low-quality reads (below Q30). Reads representing each sample were mapped onto the RSV A isolate 0594 (GenBank MW582528.1) and RSV B isolate 9671 (GenBank MW582529.1) to generate consensus sequences, which are recent dominant circulating genotypes harboring characteristic G-gene subregion duplication [27]. Finally, the sequences were deposited on GISAID (accession no. EPI_ISL_18110959–EPI_ISL_18110988) and GenBank (accession no. PP542651–PP542680).

### Sequence Analysis

Multiple sequence alignments using partial G-gene sequences were performed using MAFFT software, version 7.490 [28], and maximum likelihood phylogenies inferred (1000 bootstrap replicates) using IQ-TREE software, version 1.6.12 [29]. The best substitution model was identified using IQ-TREE, ranked by BIC (GTR + F + G4 (RSV A) and TIM3 + F + I + G4 (RSV B)). High coverage RSV A and B genome sequences, collected between 2017 and 2022, were downloaded from the GISAID database (7 November 2022), matching G-gene sequences extracted using the Basic Local Alignment Search Tool (BLAST) and analyzed alongside the sequences generated in this study [30]. Supplementary Tables 7 and 8 provides metadata on the GISAID genome sequences used in this study. Trees were visualized using FigTree software, version 1.4.4. Genotype predictions were carried out using a Nextclade online tool, version 3.4.0 [31]. Relevant aligned nucleic acid sequences were translated into the predicted protein sequence and visualized using Jalview software, version 2 [32].

## RESULTS

**Figure 1.**
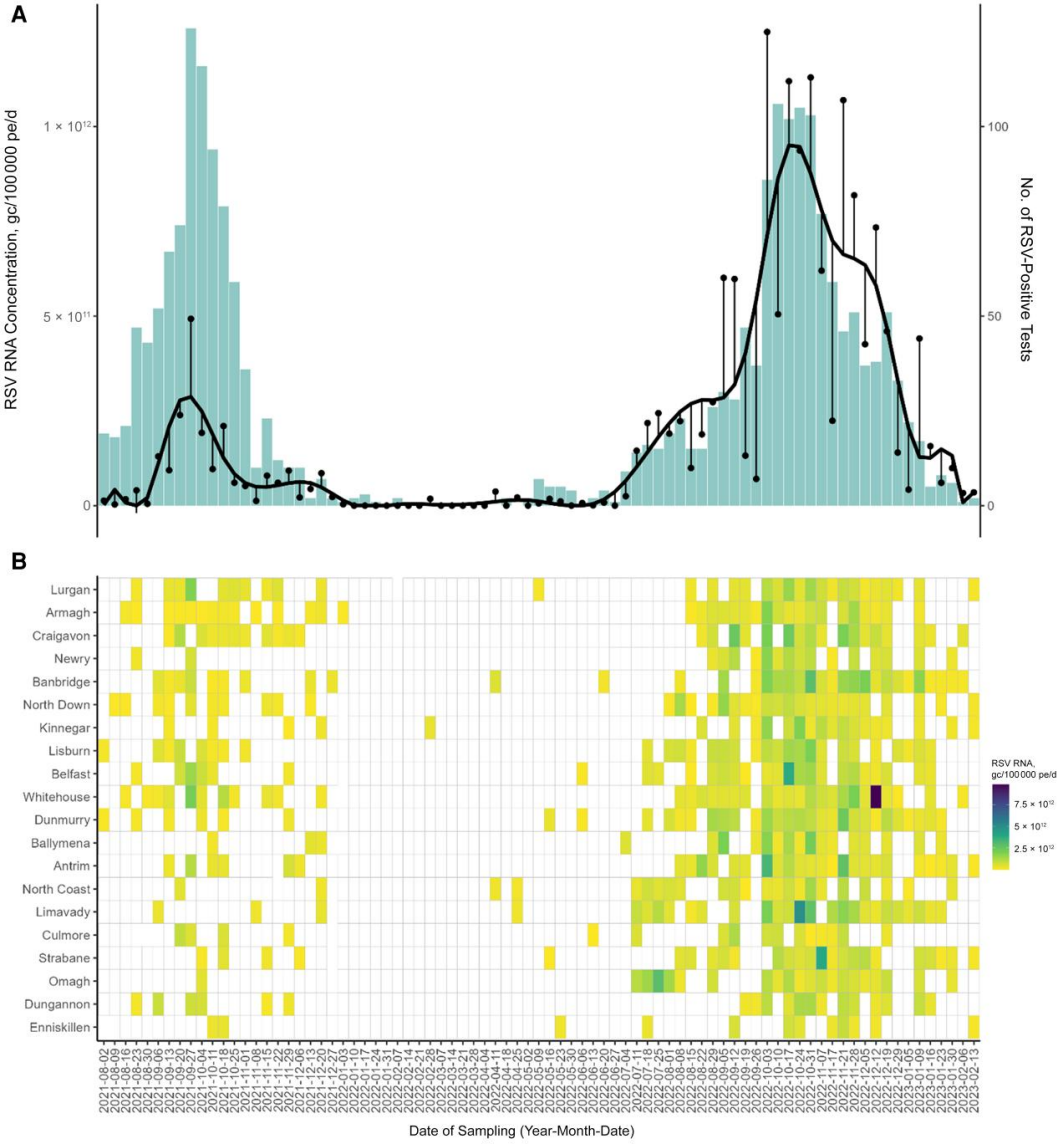
*A*, Averaged respiratory syncytial virus (RSV) RNA concentrations—in gene copies (gc) per 100 000 population equivalents (pe) per day—across 20 wastewater treatment works (WWTWs) in Northern Ireland (NI; *black circles*) and smoothed data based on fitting a B spline model (*black line*) from August 2021 to February 2023 (n = 83 weeks). Histogram showing the number of RSV-positive cases for NI during the same period. *B*, Heat map showing the distribution of RSV RNA concentrations in wastewater samples (n = 587) across the 20 WWTWs in NI between August 2021 and February 2023. White boxes indicate no detection of RSV RNA gene copies at the WWTW.

Two RSV epidemics were captured in NI (August 2021 to January 2022 and July 2022 to February 2023) (Figure 1*A*). During the 2021 season, RSV RNA was initially detected in August 2021 (average concentration, 1.31 × 10^10^ ± 3.45 × 10^10^ gc/100 000 pe/d across the 20 WWTWs), then subsequently increasing and peaking in September 2021 (average concentration, 4.93 × 10^11^ ± 6.81 × 10^11^ gc/100 000 pe/d) before decreasing from October 2021, and it was no longer detectable by January 2022. During the 2022 season, RSV concentrations increased and peaked in October 2022 (average concentration,1.25 × 10^12^ ± 8.09 × 10^11^ gc/100 000 pe/d). Between seasons, sporadic RSV detection in wastewater was noted (Figure 1*B*).

Across this sampling period, the first peak in RSV-positive clinical cases was observed in September 2021, and the second peak in October 2022. This was correlated with the temporal pattern of RSV gene fragment detection within wastewater (*F* = 86.38 [*df* = 76] for modeled data and *F* = 34.56 [*df* = 76] for raw data; both *P* < .01); a strong relationship was observed between RSV RNA concentration in wastewater and PCR-positive cases from infected individuals (*R*^2^ = 0.730 for modeled and *R*^2^ = 0.420 for raw data). The geographic distribution of RSV RNA across the 20 WWTWs varied in magnitude and spread within and between each season (Figure 1*B*). For example, in 2021 RSV RNA concentrations were highest in Lurgan, Craigavon, and Armagh, and in 2022 they were highest in Omagh, Limavady, and North Coast.

In this study, the reverse-transcription quantitative PCR assay targets a conserved portion of the N gene [21]. To provide greater resolution of RSV diversity and evolution, the second hypervariable region of the G gene was sequenced from positive wastewater samples [25]. Overall, 10 RSV A and 14 RSV B consensus sequences were derived from wastewater and 2 RSV A and 4 RSV B consensus sequences from clinical samples. Further analysis of the sequencing process, the sequencing depth for each sample, and further details on the wastewater-derived RSV A and B consensus sequences are detailed in Supplementary Tables 9–11. Supplementary Figure 4 shows the maximum likelihood phylogenetic trees of generated NI RSV A and B based on G-gene sequences.

**Figure 2.**
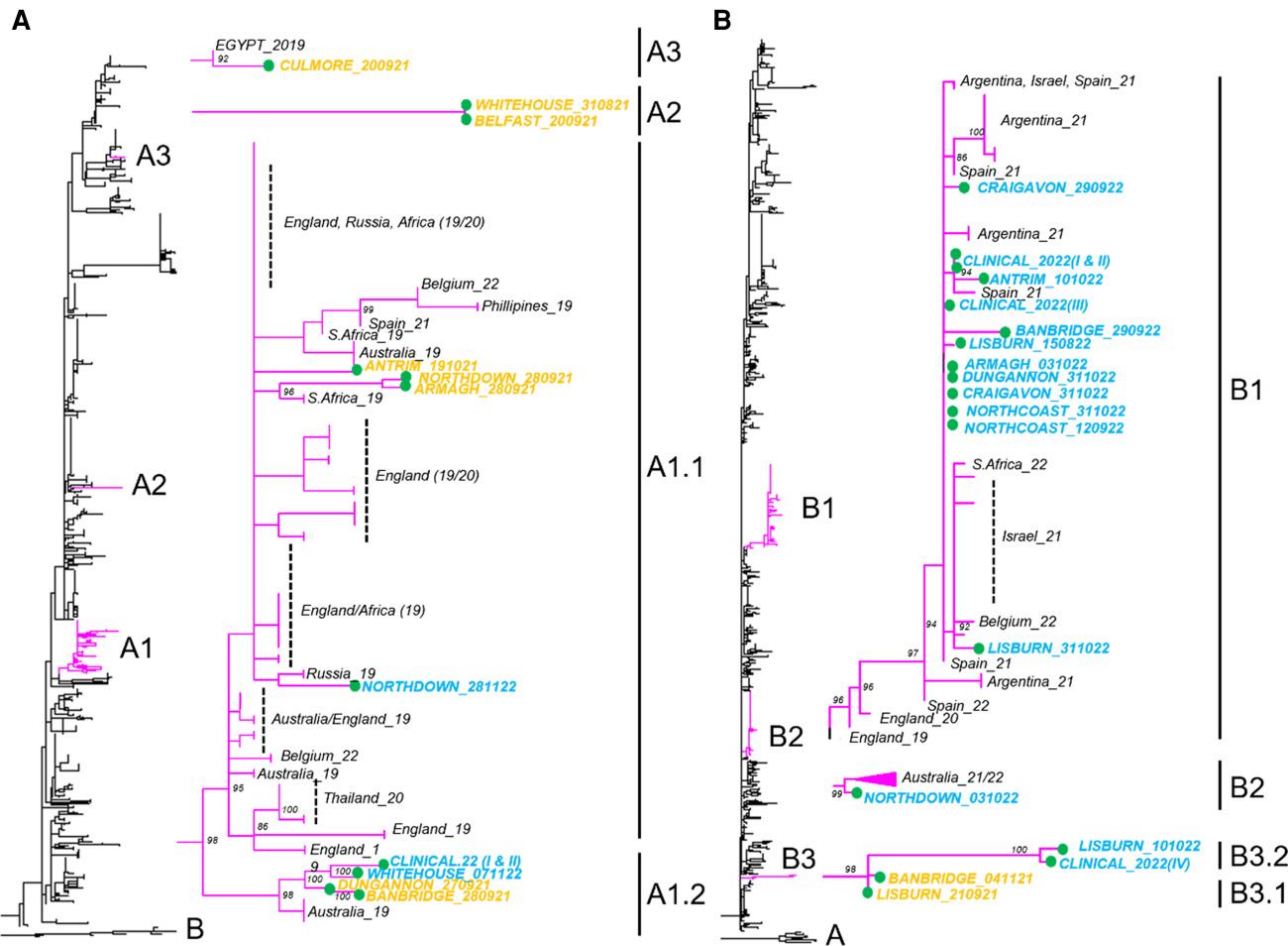
Maximum likelihood phylogenetic trees of generated Northern Ireland (NI) respiratory syncytial virus (RSV) A (*A*) and B (*B*) based on G-gene sequences alongside contemporary sequences (available through GISAID), with 1000 bootstrap replicates using IQ-TREE software. The whole phylogeny is shown on the left, with sequences generated in this study highlighted alongside focused clades containing NI sequences (*inset*). In the inset, taxa are labeled with date and geographic origin metadata for all derived NI sequences and for certain global sequences. For clarity, only bootstraps >70 are shown.

RSV A was detected more frequently in the 2021 season (8 of 10 sequences), whereas RSV B was detected more frequently in the 2022 season (12 of 14). The predicted genotypes were clade A3 (G clade GA2.3.5) for all RSV-A sequences and B6 (G clade GB5.0.5a) for all RSV-B sequences. A more detailed phylogenetic analysis of derived sequences, in the context of contemporary global RSV sequences, suggested the presence of several distinct local clades of both genotypes (A3 and B6) across both seasons in NI. For both RSV A and B, 3 major local clades were detected, herein referred to as RSV A1–3 and B1–3 (Figure 2). Clades A1 and B3 were further subdivided into 2 groups each (X.1 and X.2) due to intraclade genetic differences. However, inferred clade nomenclature could potentially differ with more sequence data for each sample (eg, whole-genome sequencing). Clinical sequences were found in the same clade as wastewater sequences.

A total of 24 RSV partial sequences from 2 seasons were generated in this study, which may inform our understanding of the spatial and temporal distribution of RSV. During the 2021 season all 3 distinct clades of RSV A (dominated by A1) and only 1 RSV B clade (B3.1) were found in wastewater samples, while during the 2022 season only 1 RSV A clade (A1.2) and all 3 RSV B clades (B1, B2, and B3.2) were found (dominated by B1). NI RSV sequences often clustered with other contemporary sequences detected globally, but in some instances sequences from both seasons clustered only with NI sequences (A1.2 and B3).

Clade A1 is subdivided into A1.1 and A1.2 sister lineages, with A1.1 being large and diverse and containing NI sequences from both 2021 and 2022 and sequences found outside NI between 2019 and 2022. In contrast, A1.2 formed its own cluster of mostly NI sequences from both 2021 and 2022, which were found in sister lineages, and a single Australian sequence from 2019 that sits basal to all A1.2 NI sequences. Clade A2 contains 2 NI sequences on a long branch with no clear close relatives in other clades. However, the most closely related sequences were from sequences isolated in Argentina and Philippines from 2019. Clade A3 contains a single NI sequence closely related to a sequence from Egypt from 2019.

Like A1, clade B1 is a large and diverse clade containing NI sequences alongside global sequences from 2021 and 2022. Interestingly, UK sequences from 2019 and 2020 sit basal with respect to the rest of the clade. Clade B2 contains a single NI sequence as a sister lineage to recent Australian sequences. Clade B3 is composed only of NI sequences but interestingly from both 2021 and 2022 and is split into B3.1 (2021) and B3.2 (2022) by the presence of a long branch linking the seasons.

**Figure 3.**
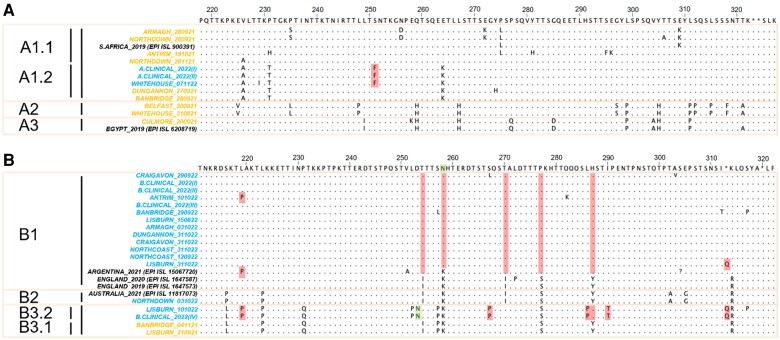
Encoded amino acid sequences for respiratory syncytial virus (RSV) A (*A*) and RSV B (*B*) samples from this study and other relevant non–Northern Ireland sequences within our clusters were visualized using Jalview software. Amino acid sequences were separated into groups based on the clades from the phylogenetic analysis. Mutations shown in reference to the consensus of all sequences [33]. Amino acids the same as consensus were highlighted with a dot. Stop codons were shown (*asterisks*). Mutations of interest were shaded in red, and N-linked glycosylation site in green.

Analysis of the predicted encoded amino acid sequences for our sequences and other select non-NI sequences within our clusters reinforced the presence of ≥3 clades per subtype (Figure 3). While differences were noted across the region, focusing between seasons within a clade several amino acids changes were identified, such as S251F in A1.2 and 8 amino acid mutations in B3.2 (L219P, L252P, D253N, I254T, L286P, Y287H, I290T, and STOP313Q). Considering the emergence of clade B1 after 2020, we identified several derived mutations preceding its emergence (I254 T, K258N, I270 T, S277P, Y287H, and R314 K). Finally, we identified several amino acid changes shared between B clades, including L219P (B1 and B3.2) and STOP313Q (B1 and B3.2) and most prominently in I254 T (B1 and B3.2) and Y287H (B1 and B3.2). Furthermore, mutations at 253 and 258 could result in the addition of an extra N-linked glycosylation site in both B1 and B3.2 in a similar region. In addition, several mutations are noted that are likely to affect O-linked glycosylation patterns through gain (I254 T in B1 and B3.2, I270 T in B1, and I290 T in B1) or loss (S251F in A1.2, T267P in B3.2, and S277P in B1) of sites. No amino acid differences specific to clinical sequences were identified compared with related wastewater sequences.

**Figure 4.**
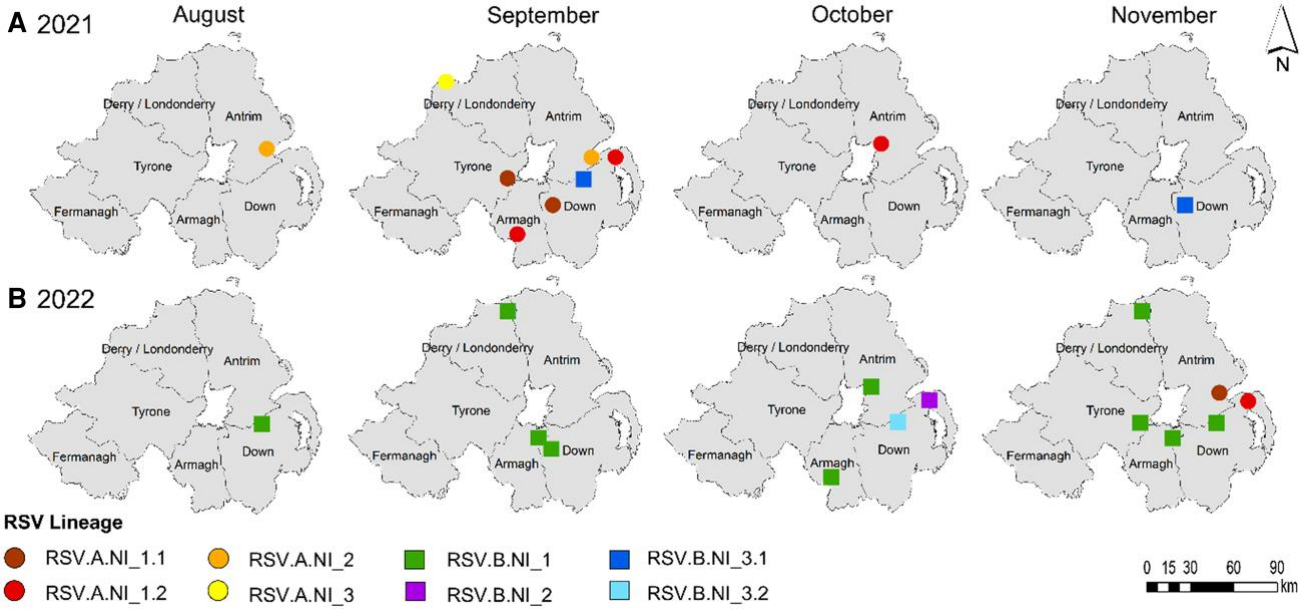
The geospatial detection of the respiratory syncytial virus (RSV) *A* and *B* lineages across 2021 and 2022 seasons in Northern Ireland (NI), divided into 6 counties (Antrim, Armagh, Fermanagh, Tyrone, Londonderry, and Down). RSV *A* and *B* lineages are shown as circles or squares, and the different RSV *A* and *B* lineages (A1.1, A1.2, A2, A3, B1, B2, B3.1, and B3.2) are shown in different colors and shades.

Depending on several factors, including the number and relative timing of introductions, as well as relative fitness levels, the distribution of distinct viral variants across a population can vary, even within a relatively small region like NI [34]. Integrating our WBE platform, the wastewater-derived RSV sequences from NI were mapped across time and space (Figure 4). This suggested that the RSV lineages were not evenly dispersed across NI, with most lineages detected in County Down and Antrim, possibly linked to their higher population density and acting as a transport hub with the capital city Belfast and airports in both. The greatest number of lineages was detected in County Down, with 7 different RSV lineages found over the 2021 and 2022 season, compared with 4 in Antrim, 2 each in Armagh and Derry/Londonderry, and 1 each in Fermanagh and Tyrone. While the dominant lineages like RSV A1.1 and A1.2 and RSV B1 were widespread, other lineages were only found in 1 county, including RSV A2 and A3 in September 2021, RSV B2 in October 2022, and RSV B3. To visualize RSV genetic diversity over time and the relationship to viral load in wastewater, a dashboard was created (http://go.qub.ac.uk/RSV-NI).

## DISCUSSION

This study demonstrated that composite influent wastewater samples followed by population normalization can be used to successfully monitor the prevalence of RSV in wastewater. The preprocessing and concentration method used was successful at detecting RSV virus from the wastewater liquid fraction, and further studies demonstrated the applicability of this method for other viruses [18, 19, 35]. RSV RNA concentration in wastewater was correlated with RSV-positive clinical cases. Furthermore, this study captured 2 unusual RSV epidemics between August 2021 and February 2023, which deviated from the traditional UK winter seasonal patterns [36] but were consistent with off-season RSV epidemics reported worldwide after the removal of COVID-19 pandemic restrictions [8]. Importantly, data generated from RSV wastewater surveillance could aid the RSV clinical response by determining the onset of the RSV season, thereby supporting public health campaigns to raise awareness regarding prevention of RSV infection and transmission. Furthermore, it could help inform clinical decisions regarding the initiation of immunoprophylaxis for high-risk infants at the onset of the RSV season.

In general, higher RSV RNA concentrations were observed in 2022 than in 2021, while the numbers of reported RSV-positive clinical cases were similar. Several factors could have contributed to these differences. First, clinical testing only detects severe disease, and therefore the burden of RSV infections in the community infected is unknown. Second, it is unknown whether RSV shedding rates in feces differ by ages, disease state, or RSV subtype, and if these factors differed between epidemics, then RSV RNA concentrations in the wastewater could vary. Third, RSV RNA concentrations were tested retrospectively for 2021 compared with 2022, and partial RNA degradation during storage may therefore have contributed to the observed lower RSV RNA concentrations [37].

This study highlights the utility of WBE for tracking RSV genetic diversity, as evidenced by the epidemiologically informative RSV G-gene sequences derived from wastewater. RSV seasons in NI were characterized by the introduction and circulation of multiple lineages, including both subtypes (RSV A and B), represented by a single genotype each but encompassing several genetically distinct clades, consistent with other studies [38, 39]. In this study, the NI sequences were similar to sequences that were concomitantly circulating worldwide and hence were reflective of global circulation patterns. The spread of acute respiratory infections is strongly influenced by the global movement of people between the southern and northern hemispheres, as well as the tropics [38]. Thus, it is hard to precisely determine the origin of NI RSV strains but, speculatively, it is likely that much of the observed RSV genetic diversity within NI was derived from outside the country. However, only 10% of samples yielded sequences (n = 24 successful), so caution is warranted regarding overinterpretation.

RSV sequences were further subdivided into 3 local clades per genotype/subtype. This is likely an underestimate given the limited number of sequences attained; these data are likely only a subset of all circulating strains in NI at the time, and among all circulating strains there are probably >3 clades, given that within some clades we find phylogenetic structuring suggestive of additional importation events from outside NI (eg, within clade A1.1). One interesting observation was the identification of sequences within clades A1.2 and B3 where the most closely related sequences between epidemics were derived from within NI. This pattern is consistent with epidemiological persistence of RSV that has been noted previously [40]. Indeed, we detected RSV in wastewater samples between seasons, but it was sporadic both spatially and temporally. However, the factors that contribute to the reemergence of RSV in the community are poorly understood, with ambient temperature, humidity, and sunlight thought to play a role on stability and infectivity of RSV, while human behavior, strain variation, and introduction of novel strains are also believed to be integral to transmission [41, 42]. Interestingly, within A1.2 the 2021 and 2022 sequences were in sister clades, which might suggest reintroduction from an unknown source, potentially even outside NI. In addition, the link between B3.1 and B3.2 is on a long branch, with multiple mutations between them, rendering interpretations regarding the origin of the strain difficult.

Importantly, this study highlighted that wastewater-derived RSV sequences were found in similar clades as RSV sequences derived from clinical samples. As such, wastewater sequencing accurately reflects the genotypes circulating in the entire community and causing clinical disease. Interestingly, wastewater-derived sequence data from other studies found that more SARS-CoV-2 lineages were circulating across the sampled communities than were represented in the clinically derived data [15, 43]. Therefore, expanding RSV genomic surveillance to encompass both clinical and environmental samples would greatly expand understanding of the genetic and epidemiological characteristics of the virus at the population level while also rapidly identifying antigenic variations and potential immune escape mutations. This ability to follow RSV circulation, and evolution at the population level will undoubtedly become even more important following the imminent introduction and widespread use of long-acting prophylactic monoclonal antibodies and RSV vaccines [44].

RSV G-gene sequencing offers insights into RSV origin and spread within NI as well as virus biology, as the G protein is involved in binding and entry of RSV [45]. Several findings of note were identified, of which the apparent convergent evolution in RSV B was the most striking. Several variable N-glycosylation sites were identified within B1 and B3.2, potentially affecting G biology and immune evasion [44, 46].

This study had several limitations. First, prepandemic samples were not available for inclusion. Second, only approximately 10% of wastewater samples were successfully sequenced using the developed protocol; however, the reasons for this remain unclear. Wastewater is inherently complex, and PCR inhibitors, variation in viral load, and fragmented viral genomes may contribute to this challenge [47–50]. Third, while the wastewater-derived sequences closely matched the clinically derived sequences, only limited clinical samples were available from 2022 and none from 2021. Fourth, lacking the geographic location and date of clinical data hindered spatial comparison with wastewater lineages. Fifth, the clinical sample positivity rate was unavailable. Finally, while the phylogenetic analysis focused on the second hypervariable region of the G gene, further insights on the genetic and epidemiological characteristics of the virus could be gained from F-gene or whole-genome sequencing. Future research could include a large longitudinal wastewater surveillance study to assess the antigenic evolution of RSV between and within seasons over many years.

Overall, the current study highlights the value of WBE as an effective tool to detect and monitor the circulation and evolution of RSV in the community. Its implementation will undoubtedly be of considerable use to public health teams worldwide to make rapid interventions to help manage RSV outbreaks. Furthermore, continued surveillance of cocirculating RSV genotypes using WBE could help identify antigenic variations and potential immune escape mutations against future pharmaceutical interventions.

## Supplementary Data

Supplementary materials are available at *The Journal of Infectious Diseases* online (http://jid.oxfordjournals.org/). Supplementary materials consist of data provided by the author that are published to benefit the reader. The posted materials are not copyedited. The contents of all supplementary data are the sole responsibility of the authors. Questions or messages regarding errors should be addressed to the author.

## Notes

***Author contributions***. D. M. A., M. I. R., D. G. D., H. E. G., J. W. M., C. G. G. B., and D. F. G. conceptualized the study. D. M. A., M. I. R., P. A., A. L., S. H. B., J. L., J. D. C., S. C., A. J. L., C. M., B. F. N., J. M., T. C., and J. M. M., contributed to data verification and/or preparation. D. M. A., M. I. R., and C. G. G. B. performed analyses. D. M. A., L. J. B., D. G. D., D. M. G., U. F. P., H. E. G., J. W. M., and C. G. G. B. contributed to the acquisition and/or interpretation of the data. D. M. A. drafted the manuscript. All coauthors contributed to the revision of the manuscript and approved the final version for submission.

***Acknowledgments***. We thank Hannah Saadi for her help completing the laboratory work for the revised manuscript. We are thankful to NI Water Ltd and the RPS Group for wastewater sample collection and to the Department of Agriculture, Environment and Rural Affairs, the NI Environment Agency, and the Department for Infrastructure and Department of Finance. We gratefully acknowledge all data contributors (ie, the authors and their originating laboratories) responsible for obtaining the specimens, and their submitting laboratories for generating the genetic sequence and metadata and sharing via the GISAID Initiative, on which this research is based.

***Data availability.*** RSV G-gene sequences were deposited on GISAID (accession nos. EPI_ISL_18110959–EPI_ISL_18110988) and GenBank (accession nos. PP542651–PP542680). The remaining data that support the findings of this study are available from the corresponding author on reasonable request.

***Financial support*.** This study was funded by the Department of Health, Northern Ireland, as part of the Northern Ireland Wastewater Surveillance Programme. The funder of the study had no role in the study design, data collection, data analysis, data interpretation or writing of the report.

## Supplementary Material

jiae205_Supplementary_Data
