# Supplementary material for: Genomic Analysis and Surveillance of Respiratory Syncytial Virus Using Wastewater-Based Epidemiology
Source: J Infect Dis. 2024 Apr 18;230(4):e895–904. doi: 10.1093/infdis/jiae205 (PMC11481326; doi:10.1093/infdis/jiae205)
Supplement: jiae205_Supplementary_Data [file jiae205_supplementary_data.docx]

**Supplementary Material**

**Genomic Analysis and Surveillance of Respiratory Syncytial Virus (RSV) Using Wastewater-Based Epidemiology (WBE)**

Danielle M. Allen ^1^, Marina I. Reyne ^1^, Pearce Allingham ^1^, Ashley Levickas ^1^, Stephen H. Bell ^1^, Jonathan Lock ^1^, Jonathon D. Coey ^1^, Stephen Carson ^1^, Andrew J. Lee ^1^, Cormac McSparron ^2^, Behnam Firoozi Nejad ^2^, James McKenna ^3^, Mark Shannon ^3^, Kathy Li ^3^, Tanya Curran ^3^, Lindsay J. Broadbent ^4^, Damian G. Downey ^5^, Ultan F. Power ^5^, Helen E. Groves ^5^, Jennifer M. McKinley ^2^, John W. McGrath ^1^, Connor G. G. Bamford ^1^, and Deirdre F. Gilpin ^6^


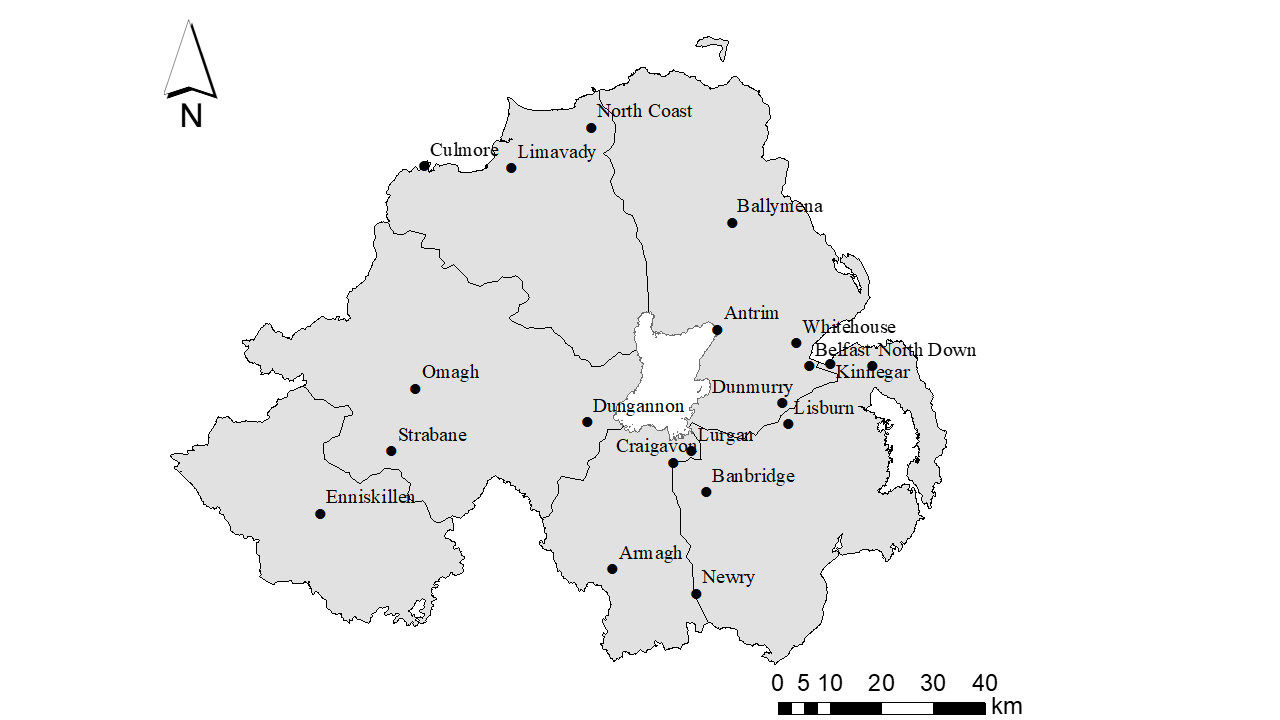


**Figure S1.** Map showing geographical locations of sampled wastewater treatment works (WWTW) across Northern Ireland (NI).

**Table S1.** County and population equivalents (p.e.) for each WWTW

| **County** | **WWTW** | **Population equivalents (p.e.)** |
| --- | --- | --- |
| Londonderry | Culmore | 94,655 |
|  | Limavady | 11,583 |
|  | North Coast | 42,440 |
| Antrim |  |  |
|  | Ballymena | 42,969 |
|  | Antrim | 41,735 |
|  | Dunmurry | 42,397 |
|  | Whitehouse | 68,925 |
|  | Belfast  Lisburn | 228,939 |
| Down |  | 47,377 |
|  | Banbridge | 18,803 |
|  | North Down | 73,384 |
|  | Kinnegar | 97,582 |
|  | Craigavon  Newry | 78,899 |
| Armagh |  | 34,042 |
|  | Armagh | 15,749 |
|  | Lurgan | 29,302 |
| Tyrone | Omagh | 20,200 |
|  | Strabane | 13,251 |
|  | Dungannon | 18,079 |
| Fermanagh | Enniskillen | 15,115 |

**Protocol S1:** RT-qPCR

A final reaction volume (25 *μ*L) containing 12.5*μ*L 2x RTPCR Buffer (AgPath-ID™ One-Step RT-PCR Reagents, ThermoFisher Scientific), 1*μ*L 25x RT-qPCR enzyme (AgPath-ID™ One-Step RT-PCR Reagents (ThermoFisher Scientific), 8.7375 *μ*L DEPC, 0.25*μ*L Bovine Serum Albumin, 0.225*μ*L forward and reverse primer (900nM), 0.0625*μ*L probe (250nM) and 2*μ*L of template. Thermal cycling parameters were one cycle of 50 °C for 10 min, followed by 45 cycles of 95°C for 10 min, and 60°C for 30 s. All wastewater samples, positive and negative controls were performed in triplicate. A sample was considered positive if one out of three replicates amplified (Ct < 40). A standard curve and limit of detection (LOD) were calculated for the assay (Protocol S3, Figure S3).

**Protocol S2:** PCR inhibition

PCR inhibition was dealt with by dilution of the template volume [1]. Template dilution can be used to reduce levels of inhibition of the amplification of targeted DNA fragment. Therefore, three template volumes were selected (2, 3 and 5 *μ*L) were tested. RT-qPCR was performed on the QuantStudio**™** 7 Real-Time PCR System (ThermoFisher Scientific) in triplicate using an RSV A and B N gene assay. Therefore, to test this three template volumes were selected (2, 3 and 5 *μ*L). The mastermix included 12.5*μ*L 2x RTPCR Buffer (AgPath-ID™ One-Step RT-PCR Reagents, ThermoFisher Scientific), 1*μ*L 25x RT-qPCR enzyme (AgPath-ID™ One-Step RT-PCR Reagents (ThermoFisher Scientific), either 8.7375, 7.7375 or 5.7375 *μ*L DEPC, 0.25*μ*L Bovine Serum Albumin, 0.225*μ*L forward and reverse primer (900nM) and 0.0625*μ*L probe (250nM). Thermal cycling parameters were one cycle of 50 °C for 10 min, followed by 45 cycles of 95°C for 10 min, and 60°C for 30 s. Results showed there was no significant difference in the Ct values obtained from the wastewater samples and therefore, a template volume of 2 *μ*L was selected.


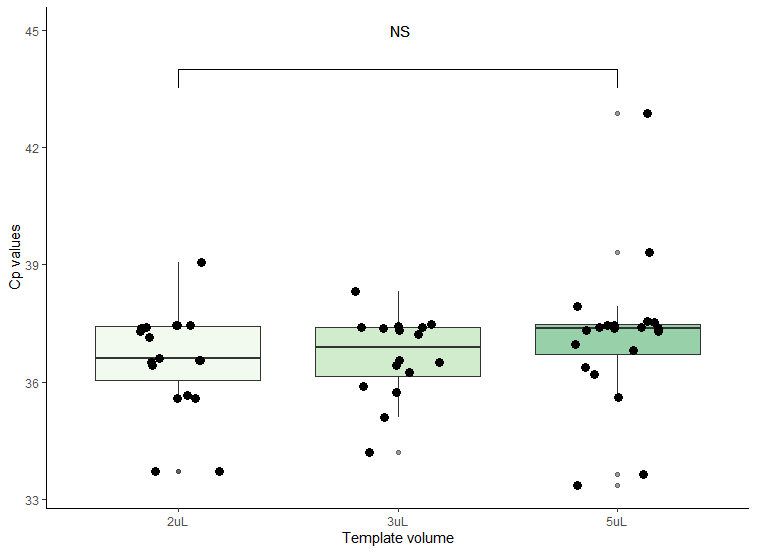


**Figure S2.** Ct values for the different template volumes (2, 3 and 5 *μ*L). NS indicates no significant difference.

**Protocol S3.** Standard curve

RSV Clinical BT2a isolate was kindly supplied by Dr Lindsay Broadbent, isolation and characterisation of this isolate was previously described [2]. RSV B positive control was purchased from ZeptoMetrix (NATtrol RSV positive Control NATRSV-6C, ZeptoMetrix). RSV A and B were quantified using digital droplet PCR (ddPCR). For this assay, ddPCR was performed on 20 µl samples from a 22 µl reaction volume, prepared using a 5.5 µl template, mixed with 5.5 µl of One-Step RT-ddPCR Advanced Kit for Probes (Bio-Rad 1863021), 2.2 µl of 200 U/µl Reverse Transcriptase, 1.1 µl of 300 mM DTT, and primers and probes at a final concentration of 900 and 250 nM, respectively. Droplets were generated using the AutoDG Automated Droplet Generator (Bio-Rad). PCR was performed using C1000 Touch Thermal Cycler with the following cycling conditions: 50°C for 60 mins, 95°C for 10 mins, 40 cycles at 95°C for 30 sec and 55°C for 1 min, and 98 °C for 10 mins. Droplets were analysed using the QX200 Droplet Reader (Bio-Rad), and each well had to have over 10,000 droplets for inclusion in the analysis. Thresholding was done using the QX Manager Software (Bio-Rad, version 1.2). A standard curve was generated for RSV A and B N gene using sixfold serial dilutions of a quantified positive control.


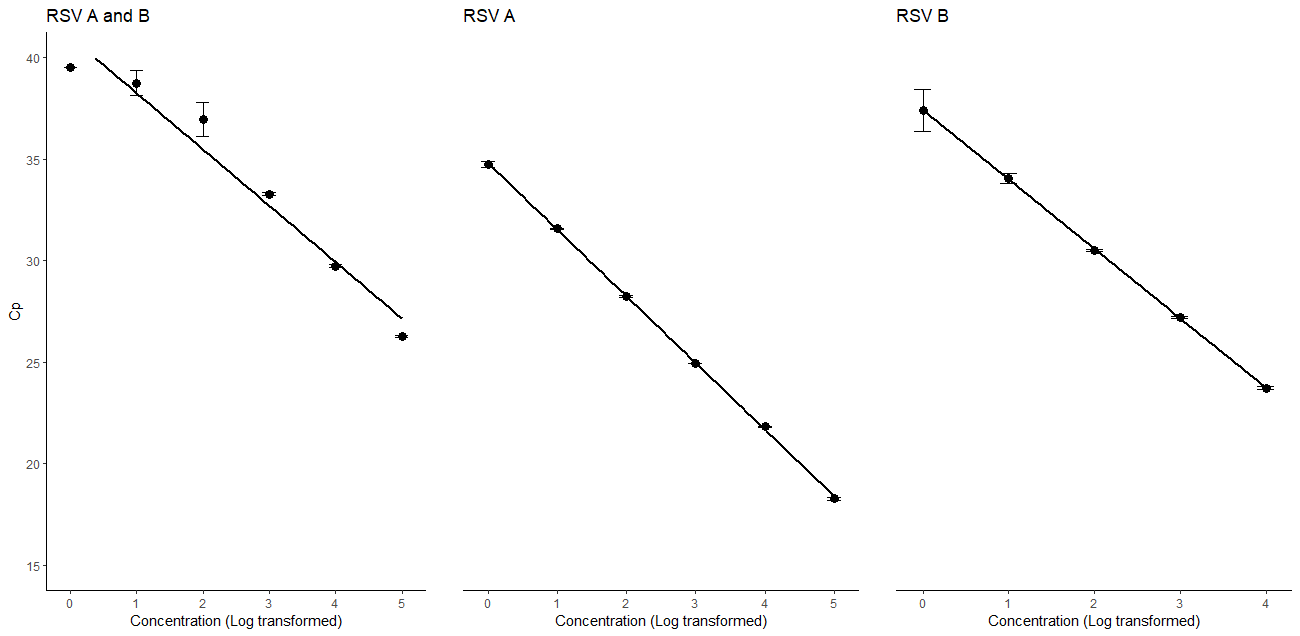


**Figure S3.** Standard curve for RSV A and B N gene.

Amplification efficiency was 129%. The Limit of Detection (LOD) for RSV RNA detection in wastewater was 1.9 copies per reaction

**Protocol S4:** Statistical analysis

Time was treated as an independent variable, different numbers and placements of knots were tested to smooth the data, and the final model was selected based on the Bayesian Information Criterion (BIC)

**Table S2**: B spline model results for the different number and placement of knots. Only the top 3 models are shown in the table. *p* is the significance value of the regression coefficient, *R^2^* is the coefficient of determination and BIC is the Bayesian Information Criterion. * indicates the single best model as selected by BIC.

| **Model** | ***F*** | ***p*** | ***R^2^ adj.*** | **BIC** |
| --- | --- | --- | --- | --- |
| knot = 21* | 7.282 | <0.001 | 0.6379 | 4619 |
| knot = 28 | 5.731 | <0.001 | 0.6338 | 4641 |
| knot = 42 | 4.307 | <0.001 | 0.6343 | 4674 |


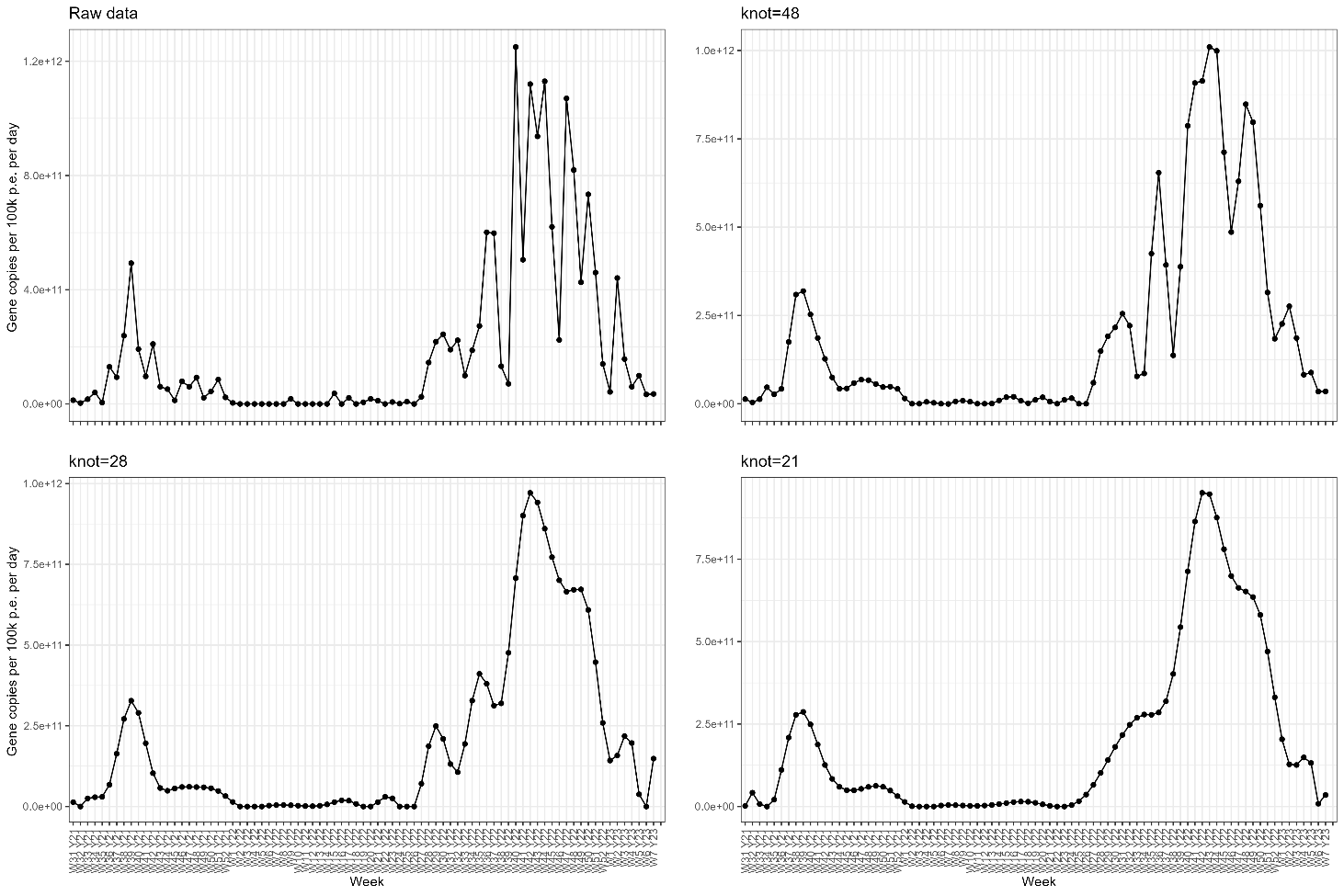
**Figure S4**: Average RSV RNA concentrations (g.c. per 100,000 p.e. per day) across 20 WWTW: raw and smoothed data based on fitting B spline models with different number and placement of knots.

**Protocol S5.** Additional statistical analysis

Additional analyses were performed to evaluate if RSV RNA concentration in wastewater can be used to determine the onset of RSV season. Four linear regression models were generated and compared to determine wastewater lead times. Raw wastewater data for the 2022-23 RSV season was shifted by one, two and three weeks earlier and used as an explanatory variable. Two weeks lead time model performed the best based on AIC/BIC values (Table S2). However, defining the lead time for wastewater is complex and depends on biological, epidemiological, behavioural and health care systems factors [3]. Moreover, current analysis are based on one unusual RSV season and further investigation across several RSV seasons is needed to determine potential lead times.

**Table S3.** Model comparison of the lead times for wastewater RSV concentration.

|  | Adj R2 | AIC | delta AIC | BIC | delta BIC | p-value |
| --- | --- | --- | --- | --- | --- | --- |
| Two weeks lead time | 0.53 | 144.03 | 0.00 | 146.35 | 0.00 | <0.001 |
| Original (no shift) | 0.42 | 147.40 | 3.36 | 149.71 | 3.36 | <0.001 |
| Three weeks lead time | 0.35 | 149.24 | 5.21 | 151.56 | 5.21 | 0.01 |
| One week lead time | 0.34 | 149.47 | 5.44 | 151.79 | 5.44 | 0.01 |
| Four weeks lead time | 0.26 | 151.25 | 7.21 | 153.57 | 7.21 | 0.03 |

**Table S4.** Description of PCR primers used for the genotyping of RSV A and B in wastewater samples.

| **Assay** | **Region** | **Primer name** | **Sequence (5’ - 3’)** | **Final reaction concn (nM)** | **Amplicon size (bp)** |
| --- | --- | --- | --- | --- | --- |
| Sequencing: External PCR | G gene | ABG490 - Forward | ATGATTWYCAYTTTGAAGTGTTC | 500 | A: 607  B: 661  BA: 670 |
|  |  | F164 - Reverse | GTTATGACACTGGTATACCAACC | 500 |  |
| Sequencing: Seminested PCR | G gene | AG655 – RSV A Forward | GATCYCAAACCTCAAACCAC | 500 | A: 460  B: 585  BA: 645 |
|  |  | BG517 – RSV B Forward | TTYGTTCCCTGTAGTATATGTG | 500 |  |
|  |  | F164 – RSV A and B Reverse | GTTATGACACTGGTATACCAACC | 500 |  |

**Table S5:** Reaction mixture conditions for external and semi-nested PCRs

| **Reaction Mixture** | **Final reaction concn (nM)** | **1 reaction (µL)** |
| --- | --- | --- |
| Reaction buffer (New England BioLabs) |  | 5 |
| Q5 High-Fidelity DNA Polymerase (New England BioLabs) |  | 0.25 |
| dNTPs (New England BioLabs) | 10 | 0.5 |
| Q5 High GC Enhancer (New England BioLabs) |  | 5 |
| Forward primer (Eurofins) | 500 | 1.25 |
| Reverse primer (Eurofins) | 500 | 1.25 |
| Nuclease Free Water |  | 9.25 |
| Template |  | 2.5 |
| Total |  | 25 |

**Table S6:** Thermocycling conditions for external and semi-nested PCRs

| **Thermocycling conditions** | **External PCR** | | | **Semi-nested PCR for RSV A** | | | **Semi-nested PCR for RSV B** | | |
| --- | --- | --- | --- | --- | --- | --- | --- | --- | --- |
|  | **Temperature (°C)** | **Time (s)** | **Number of cycles** | **Temperature (°C)** | **Time (s)** | **Number of cycles** | **Temperature (°C)** | **Time**  **(s)** | **Number of cycles** |
| Initial denaturation | 98 | 60 |  | 98 | 60 |  | 98 | 60 |  |
| Annealing | 98 | 10 | 35 | 98 | 10 | 30 | 98 | 10 | 30 |
|  | 54 | 30 |  | 62 | 30 |  | 56 | 30 |  |
|  | 72 | 40 |  | 72 | 40 |  | 72 | 40 |  |
| Final extension | 72 | 120 |  | 72 | 120 |  | 72 | 120 |  |
| Hold | 4 | ∞ |  | 4 | ∞ |  | 4 | ∞ |  |

**Table 7:** Metdata on GISAID RSV A genome sequences used in this study

| **Query** | **GISAID ID** |
| --- | --- |
| >lcl\|Query_54286:5258-5587 | hRSV/A/England/195040643/2019\|EPI_ISL_1834158\|2019-12-09 |
| >lcl\|Query_54217:5267-5596 | hRSV/A/England/194540440/2019\|EPI_ISL_1647385\|2019-11-04 |
| >lcl\|Query_5857:5258-5587 | hRSV/A/England/195040643/2019\|EPI_ISL_1834158\|2019-12-09 |
| >lcl\|Query_5788:5267-5596 | hRSV/A/England/194540440/2019\|EPI_ISL_1647385\|2019-11-04 |
| >lcl\|Query_54223:5255-5584 | hRSV/A/England/195080598/2019\|EPI_ISL_1647391\|2019-12-09 |
| >lcl\|Query_5794:5255-5584 | hRSV/A/England/195080598/2019\|EPI_ISL_1647391\|2019-12-09 |
| >lcl\|Query_54507:5285-5614 | hRSV/A/England/RE20000021/2020\|EPI_ISL_1647411\|2020-01-05 |
| >lcl\|Query_6078:5285-5614 | hRSV/A/England/RE20000021/2020\|EPI_ISL_1647411\|2020-01-05 |
| >lcl\|Query_54212:5265-5594 | hRSV/A/England/RE20000093/2020\|EPI_ISL_1520438\|2020-01-28 |
| >lcl\|Query_5783:5265-5594 | hRSV/A/England/RE20000093/2020\|EPI_ISL_1520438\|2020-01-28 |
| >lcl\|Query_54340:5234-5563 | hRSV/A/Russia/Novosibirsk-138Hp/2019\|EPI_ISL_2582836\|2019-12-06 |
| >lcl\|Query_5911:5234-5563 | hRSV/A/Russia/Novosibirsk-138Hp/2019\|EPI_ISL_2582836\|2019-12-06 |
| >lcl\|Query_54517:5285-5614 | hRSV/A/England/RS20000581/2020\|EPI_ISL_1647421\|2020-02-04 |
| >lcl\|Query_6088:5285-5614 | hRSV/A/England/RS20000581/2020\|EPI_ISL_1647421\|2020-02-04 |
| >lcl\|Query_54284:5245-5574 | hRSV/A/England/194980363/2019\|EPI_ISL_1834154\|2019-12-05 |
| >lcl\|Query_5855:5245-5574 | hRSV/A/England/194980363/2019\|EPI_ISL_1834154\|2019-12-05 |
| >lcl\|Query_54279:5285-5614 | hRSV/A/Australia/VIC-RCH202/2019\|EPI_ISL_1834149\|2019-12-09 |
| >lcl\|Query_5850:5285-5614 | hRSV/A/Australia/VIC-RCH202/2019\|EPI_ISL_1834149\|2019-12-09 |
| >lcl\|Query_54266:5281-5610 | hRSV/A/Australia/VIC-RCH160/2019\|EPI_ISL_1834136\|2019-08-18 |
| >lcl\|Query_5837:5281-5610 | hRSV/A/Australia/VIC-RCH160/2019\|EPI_ISL_1834136\|2019-08-18 |
| >lcl\|Query_54219:5271-5600 | hRSV/A/England/194660588/2019\|EPI_ISL_1647387\|2019-11-13 |
| >lcl\|Query_5790:5271-5600 | hRSV/A/England/194660588/2019\|EPI_ISL_1647387\|2019-11-13 |
| >lcl\|Query_54459:5301-5630 | hRSV/A/Philippines/99079/2019\|EPI_ISL_12970422\|2019-12-07 |
| >lcl\|Query_6030:5301-5630 | hRSV/A/Philippines/99079/2019\|EPI_ISL_12970422\|2019-12-07 |
| >lcl\|Query_54464:5301-5630 | hRSV/A/Philippines/99087/2020\|EPI_ISL_12970428\|2020-03-09 |
| >lcl\|Query_54461:5301-5630 | hRSV/A/Philippines/99082/2020\|EPI_ISL_12970425\|2020-01-02 |
| >lcl\|Query_54460:5301-5630 | hRSV/A/Philippines/99080/2019\|EPI_ISL_12970423\|2019-12-10 |
| >lcl\|Query_54456:5301-5630 | hRSV/A/Philippines/99076/2019\|EPI_ISL_12970419\|2019-11-09 |
| >lcl\|Query_54453:5301-5630 | hRSV/A/Philippines/99073/2019\|EPI_ISL_12970416\|2019-10-15 |
| >lcl\|Query_54451:5301-5630 | hRSV/A/Philippines/99071/2019\|EPI_ISL_12970414\|2019-10-01 |
| >lcl\|Query_6035:5301-5630 | hRSV/A/Philippines/99087/2020\|EPI_ISL_12970428\|2020-03-09 |
| >lcl\|Query_6032:5301-5630 | hRSV/A/Philippines/99082/2020\|EPI_ISL_12970425\|2020-01-02 |
| >lcl\|Query_6031:5301-5630 | hRSV/A/Philippines/99080/2019\|EPI_ISL_12970423\|2019-12-10 |
| >lcl\|Query_6027:5301-5630 | hRSV/A/Philippines/99076/2019\|EPI_ISL_12970419\|2019-11-09 |
| >lcl\|Query_6024:5301-5630 | hRSV/A/Philippines/99073/2019\|EPI_ISL_12970416\|2019-10-15 |
| >lcl\|Query_6022:5301-5630 | hRSV/A/Philippines/99071/2019\|EPI_ISL_12970414\|2019-10-01 |
| >lcl\|Query_54368:5276-5605 | hRSV/A/Egypt/C-CPHL001/2019\|EPI_ISL_6208719\|2019-01-06 |
| >lcl\|Query_5939:5276-5605 | hRSV/A/Egypt/C-CPHL001/2019\|EPI_ISL_6208719\|2019-01-06 |
| >lcl\|Query_54463:5301-5630 | hRSV/A/Philippines/99085/2020\|EPI_ISL_12970427\|2020-02-04 |
| >lcl\|Query_6034:5301-5630 | hRSV/A/Philippines/99085/2020\|EPI_ISL_12970427\|2020-02-04 |
| >lcl\|Query_54332:5274-5603 | hRSV/A/Mongolia/NCCD020/2019\|EPI_ISL_2543840\|2019-03-26 |
| >lcl\|Query_54331:5273-5602 | hRSV/A/Mongolia/NCCD018/2019\|EPI_ISL_2543839\|2019-02-14 |
| >lcl\|Query_54325:5274-5603 | hRSV/A/Mongolia/NCCD008/2019\|EPI_ISL_2543833\|2019-01-17 |
| >lcl\|Query_54321:5273-5602 | hRSV/A/Mongolia/NCCD006/2019\|EPI_ISL_2543829\|2019-01-15 |
| >lcl\|Query_54319:5273-5602 | hRSV/A/Mongolia/NCCD004/2019\|EPI_ISL_2543827\|2019-01-07 |
| >lcl\|Query_5903:5274-5603 | hRSV/A/Mongolia/NCCD020/2019\|EPI_ISL_2543840\|2019-03-26 |
| >lcl\|Query_5902:5273-5602 | hRSV/A/Mongolia/NCCD018/2019\|EPI_ISL_2543839\|2019-02-14 |
| >lcl\|Query_5896:5274-5603 | hRSV/A/Mongolia/NCCD008/2019\|EPI_ISL_2543833\|2019-01-17 |
| >lcl\|Query_5892:5273-5602 | hRSV/A/Mongolia/NCCD006/2019\|EPI_ISL_2543829\|2019-01-15 |
| >lcl\|Query_5890:5273-5602 | hRSV/A/Mongolia/NCCD004/2019\|EPI_ISL_2543827\|2019-01-07 |
| >lcl\|Query_54372:5276-5605 | hRSV/A/Egypt/KB-CPHL004/2020\|EPI_ISL_6208727\|2020-01-08 |
| >lcl\|Query_54369:5281-5610 | hRSV/A/Egypt/KB-CPHL001/2020\|EPI_ISL_6208724\|2020-01-05 |
| >lcl\|Query_5943:5276-5605 | hRSV/A/Egypt/KB-CPHL004/2020\|EPI_ISL_6208727\|2020-01-08 |
| >lcl\|Query_5940:5281-5610 | hRSV/A/Egypt/KB-CPHL001/2020\|EPI_ISL_6208724\|2020-01-05 |
| >lcl\|Query_54371:5272-5601 | hRSV/A/Egypt/KB-CPHL003/2020\|EPI_ISL_6208726\|2020-01-08 |
| >lcl\|Query_54370:5272-5601 | hRSV/A/Egypt/KB-CPHL002/2020\|EPI_ISL_6208725\|2020-01-08 |
| >lcl\|Query_5942:5272-5601 | hRSV/A/Egypt/KB-CPHL003/2020\|EPI_ISL_6208726\|2020-01-08 |
| >lcl\|Query_5941:5272-5601 | hRSV/A/Egypt/KB-CPHL002/2020\|EPI_ISL_6208725\|2020-01-08 |
| >lcl\|Query_54235:5282-5611 | hRSV/A/Australia/VIC-RCH083/2017\|EPI_ISL_1834103\|2017-07-26 |
| >lcl\|Query_5806:5282-5611 | hRSV/A/Australia/VIC-RCH083/2017\|EPI_ISL_1834103\|2017-07-26 |
| >lcl\|Query_54430:5272-5601 | hRSV/A/South_Africa/NICD-R03671/2021\|EPI_ISL_12529637\|2021-03-12 |
| >lcl\|Query_54283:5239-5568 | hRSV/A/England/194980352/2019\|EPI_ISL_1834153\|2019-12-05 |
| >lcl\|Query_6001:5272-5601 | hRSV/A/South_Africa/NICD-R03671/2021\|EPI_ISL_12529637\|2021-03-12 |
| >lcl\|Query_5854:5239-5568 | hRSV/A/England/194980352/2019\|EPI_ISL_1834153\|2019-12-05 |
| >lcl\|Query_54184:5258-5587 | hRSV/A/England/200100153/2019\|EPI_ISL_1520410\|2019-12-24 |
| >lcl\|Query_5755:5258-5587 | hRSV/A/England/200100153/2019\|EPI_ISL_1520410\|2019-12-24 |
| >lcl\|Query_54467:5259-5588 | hRSV/A/Argentina/BA-HNRG-413/2018\|EPI_ISL_15067676\|2018-06-18 |
| >lcl\|Query_54166:5259-5588 | hRSV/A/Argentina/HNRG-413/2018\|EPI_ISL_15055311\|2018-06-18 |
| >lcl\|Query_6038:5259-5588 | hRSV/A/Argentina/BA-HNRG-413/2018\|EPI_ISL_15067676\|2018-06-18 |
| >lcl\|Query_5737:5259-5588 | hRSV/A/Argentina/HNRG-413/2018\|EPI_ISL_15055311\|2018-06-18 |
| >lcl\|Query_54514:5282-5611 | hRSV/A/England/RE20000102/2020\|EPI_ISL_1647418\|2020-02-08 |
| >lcl\|Query_54506:5284-5613 | hRSV/A/England/RE20000020/2020\|EPI_ISL_1647410\|2020-01-06 |
| >lcl\|Query_6085:5282-5611 | hRSV/A/England/RE20000102/2020\|EPI_ISL_1647418\|2020-02-08 |
| >lcl\|Query_6077:5284-5613 | hRSV/A/England/RE20000020/2020\|EPI_ISL_1647410\|2020-01-06 |
| >lcl\|Query_54458:5301-5630 | hRSV/A/Philippines/99078/2019\|EPI_ISL_12970421\|2019-11-28 |
| >lcl\|Query_54448:5301-5630 | hRSV/A/Philippines/99068/2019\|EPI_ISL_12970411\|2019-09-14 |
| >lcl\|Query_54447:5301-5630 | hRSV/A/Philippines/99066/2019\|EPI_ISL_12970409\|2019-08-28 |
| >lcl\|Query_54445:5301-5630 | hRSV/A/Philippines/99064/2019\|EPI_ISL_12970407\|2019-08-19 |
| >lcl\|Query_6029:5301-5630 | hRSV/A/Philippines/99078/2019\|EPI_ISL_12970421\|2019-11-28 |
| >lcl\|Query_6019:5301-5630 | hRSV/A/Philippines/99068/2019\|EPI_ISL_12970411\|2019-09-14 |
| >lcl\|Query_6018:5301-5630 | hRSV/A/Philippines/99066/2019\|EPI_ISL_12970409\|2019-08-28 |
| >lcl\|Query_6016:5301-5630 | hRSV/A/Philippines/99064/2019\|EPI_ISL_12970407\|2019-08-19 |
| >lcl\|Query_54454:5301-5630 | hRSV/A/Philippines/99074/2019\|EPI_ISL_12970417\|2019-10-19 |
| >lcl\|Query_6025:5301-5630 | hRSV/A/Philippines/99074/2019\|EPI_ISL_12970417\|2019-10-19 |
| >lcl\|Query_54483:5258-5587 | hRSV/A/Argentina/BA-HNRG-453/2021\|EPI_ISL_15067692\|2021-08-09 |
| >lcl\|Query_54481:5258-5587 | hRSV/A/Argentina/BA-HNRG-448/2021\|EPI_ISL_15067690\|2021-07-19 |
| >lcl\|Query_54480:5258-5587 | hRSV/A/Argentina/BA-HNRG-443/2021\|EPI_ISL_15067689\|2021-05-31 |
| >lcl\|Query_54479:5258-5587 | hRSV/A/Argentina/BA-HNRG-442/2021\|EPI_ISL_15067688\|2021-05-05 |
| >lcl\|Query_54182:5258-5587 | hRSV/A/Argentina/HNRG-453/2021\|EPI_ISL_15055327\|2021-08-09 |
| >lcl\|Query_54180:5258-5587 | hRSV/A/Argentina/HNRG-448/2021\|EPI_ISL_15055325\|2021-07-19 |
| >lcl\|Query_54179:5258-5587 | hRSV/A/Argentina/HNRG-443/2021\|EPI_ISL_15055324\|2021-05-31 |
| >lcl\|Query_54178:5258-5587 | hRSV/A/Argentina/HNRG-442/2021\|EPI_ISL_15055323\|2021-05-05 |
| >lcl\|Query_6054:5258-5587 | hRSV/A/Argentina/BA-HNRG-453/2021\|EPI_ISL_15067692\|2021-08-09 |
| >lcl\|Query_6052:5258-5587 | hRSV/A/Argentina/BA-HNRG-448/2021\|EPI_ISL_15067690\|2021-07-19 |
| >lcl\|Query_6051:5258-5587 | hRSV/A/Argentina/BA-HNRG-443/2021\|EPI_ISL_15067689\|2021-05-31 |
| >lcl\|Query_6050:5258-5587 | hRSV/A/Argentina/BA-HNRG-442/2021\|EPI_ISL_15067688\|2021-05-05 |
| >lcl\|Query_5753:5258-5587 | hRSV/A/Argentina/HNRG-453/2021\|EPI_ISL_15055327\|2021-08-09 |
| >lcl\|Query_5751:5258-5587 | hRSV/A/Argentina/HNRG-448/2021\|EPI_ISL_15055325\|2021-07-19 |
| >lcl\|Query_5750:5258-5587 | hRSV/A/Argentina/HNRG-443/2021\|EPI_ISL_15055324\|2021-05-31 |
| >lcl\|Query_5749:5258-5587 | hRSV/A/Argentina/HNRG-442/2021\|EPI_ISL_15055323\|2021-05-05 |
| >lcl\|Query_54488:5264-5593 | hRSV/A/England/195180517/2019\|EPI_ISL_1520403\|2019-12-17 |
| >lcl\|Query_6059:5264-5593 | hRSV/A/England/195180517/2019\|EPI_ISL_1520403\|2019-12-17 |
| >lcl\|Query_54484:5258-5587 | hRSV/A/Argentina/BA-HNRG-455/2021\|EPI_ISL_15067693\|2021-08-29 |
| >lcl\|Query_54183:5258-5587 | hRSV/A/Argentina/HNRG-455/2021\|EPI_ISL_15055328\|2021-08-29 |
| >lcl\|Query_6055:5258-5587 | hRSV/A/Argentina/BA-HNRG-455/2021\|EPI_ISL_15067693\|2021-08-29 |
| >lcl\|Query_5754:5258-5587 | hRSV/A/Argentina/HNRG-455/2021\|EPI_ISL_15055328\|2021-08-29 |
| >lcl\|Query_54455:5301-5630 | hRSV/A/Philippines/99075/2019\|EPI_ISL_12970418\|2019-11-04 |
| >lcl\|Query_54450:5301-5630 | hRSV/A/Philippines/99070/2019\|EPI_ISL_12970413\|2019-09-23 |
| >lcl\|Query_54449:5301-5630 | hRSV/A/Philippines/99069/2019\|EPI_ISL_12970412\|2019-09-09 |
| >lcl\|Query_6026:5301-5630 | hRSV/A/Philippines/99075/2019\|EPI_ISL_12970418\|2019-11-04 |
| >lcl\|Query_6021:5301-5630 | hRSV/A/Philippines/99070/2019\|EPI_ISL_12970413\|2019-09-23 |
| >lcl\|Query_6020:5301-5630 | hRSV/A/Philippines/99069/2019\|EPI_ISL_12970412\|2019-09-09 |
| >lcl\|Query_54225:5268-5597 | hRSV/A/England/200200205/2020\|EPI_ISL_1647393\|2020-01-03 |
| >lcl\|Query_5796:5268-5597 | hRSV/A/England/200200205/2020\|EPI_ISL_1647393\|2020-01-03 |
| >lcl\|Query_54385:5285-5614 | hRSV/A/Thailand/CMI-6/2020\|EPI_ISL_11019782\|2020-03-31 |
| >lcl\|Query_5956:5285-5614 | hRSV/A/Thailand/CMI-6/2020\|EPI_ISL_11019782\|2020-03-31 |
| >lcl\|Query_54471:5257-5586 | hRSV/A/Argentina/BA-HNRG-432/2019\|EPI_ISL_15067680\|2019-06-06 |
| >lcl\|Query_54170:5257-5586 | hRSV/A/Argentina/HNRG-432/2019\|EPI_ISL_15055315\|2019-06-06 |
| >lcl\|Query_6042:5257-5586 | hRSV/A/Argentina/BA-HNRG-432/2019\|EPI_ISL_15067680\|2019-06-06 |
| >lcl\|Query_5741:5257-5586 | hRSV/A/Argentina/HNRG-432/2019\|EPI_ISL_15055315\|2019-06-06 |
| >lcl\|Query_54465:5300-5629 | hRSV/A/Belgium/SCI-VD-FD-00001/2022\|EPI_ISL_13297987\|2022-04-14 |
| >lcl\|Query_6036:5300-5629 | hRSV/A/Belgium/SCI-VD-FD-00001/2022\|EPI_ISL_13297987\|2022-04-14 |
| >lcl\|Query_54220:5252-5580 | hRSV/A/England/194660604/2019\|EPI_ISL_1647388\|2019-11-12 |
| >lcl\|Query_5791:5252-5580 | hRSV/A/England/194660604/2019\|EPI_ISL_1647388\|2019-11-12 |
| >lcl\|Query_54442:5320-5649 | hRSV/A/Philippines/99058/2019\|EPI_ISL_12970401\|2019-03-04 |
| >lcl\|Query_6013:5320-5649 | hRSV/A/Philippines/99058/2019\|EPI_ISL_12970401\|2019-03-04 |
| >lcl\|Query_54504:5289-5618 | hRSV/A/England/RE20000012/2020\|EPI_ISL_1647408\|2020-01-01 |
| >lcl\|Query_54231:5290-5619 | hRSV/A/England/RE19003143/2019\|EPI_ISL_1647399\|2019-11-12 |
| >lcl\|Query_54216:5290-5619 | hRSV/A/England/194400253/2019\|EPI_ISL_1647384\|2019-10-24 |
| >lcl\|Query_54211:5279-5608 | hRSV/A/England/RE20000009/2019\|EPI_ISL_1520437\|2019-12-29 |
| >lcl\|Query_54203:5284-5613 | hRSV/A/England/RE19003239/2019\|EPI_ISL_1520429\|2019-12-10 |
| >lcl\|Query_6075:5289-5618 | hRSV/A/England/RE20000012/2020\|EPI_ISL_1647408\|2020-01-01 |
| >lcl\|Query_5802:5290-5619 | hRSV/A/England/RE19003143/2019\|EPI_ISL_1647399\|2019-11-12 |
| >lcl\|Query_5787:5290-5619 | hRSV/A/England/194400253/2019\|EPI_ISL_1647384\|2019-10-24 |
| >lcl\|Query_5782:5279-5608 | hRSV/A/England/RE20000009/2019\|EPI_ISL_1520437\|2019-12-29 |
| >lcl\|Query_5774:5284-5613 | hRSV/A/England/RE19003239/2019\|EPI_ISL_1520429\|2019-12-10 |
| >lcl\|Query_54218:5278-5607 | hRSV/A/England/194600343/2019\|EPI_ISL_1647386\|2019-11-08 |
| >lcl\|Query_5789:5278-5607 | hRSV/A/England/194600343/2019\|EPI_ISL_1647386\|2019-11-08 |
| >lcl\|Query_54291:5250-5579 | hRSV/A/England/RE20000092/2020\|EPI_ISL_1834173\|2020-01-28 |
| >lcl\|Query_54213:5271-5600 | hRSV/A/England/RE20000094/2020\|EPI_ISL_1520439\|2020-01-28 |
| >lcl\|Query_5862:5250-5579 | hRSV/A/England/RE20000092/2020\|EPI_ISL_1834173\|2020-01-28 |
| >lcl\|Query_5784:5271-5600 | hRSV/A/England/RE20000094/2020\|EPI_ISL_1520439\|2020-01-28 |
| >lcl\|Query_54270:5283-5612 | hRSV/A/Australia/VIC-RCH179/2018\|EPI_ISL_1834140\|2018-11-29 |
| >lcl\|Query_5841:5283-5612 | hRSV/A/Australia/VIC-RCH179/2018\|EPI_ISL_1834140\|2018-11-29 |
| >lcl\|Query_54422:5277-5606 | hRSV/A/Australia/VIC-RCH071/2021\|EPI_ISL_11817092\|2021-03-27 |
| >lcl\|Query_54419:5302-5631 | hRSV/A/Australia/QLD-RBWH278/2021\|EPI_ISL_11817078\|2021-04-01 |
| >lcl\|Query_54417:5304-5633 | hRSV/A/Australia/QLD-RBWH264/2021\|EPI_ISL_11817075\|2021-03-27 |
| >lcl\|Query_54416:5304-5633 | hRSV/A/Australia/QLD-RBWH244/2021\|EPI_ISL_11817072\|2021-03-18 |
| >lcl\|Query_54411:5303-5632 | hRSV/A/Australia/QLD-RBWH206/2021\|EPI_ISL_11817065\|2021-03-10 |
| >lcl\|Query_54404:5304-5633 | hRSV/A/Australia/QLD-RBWH075/2021\|EPI_ISL_11817046\|2021-02-09 |
| >lcl\|Query_54402:5304-5633 | hRSV/A/Australia/QLD-RBWH068/2021\|EPI_ISL_11817043\|2021-02-07 |
| >lcl\|Query_54392:5304-5633 | hRSV/A/Australia/QLD-RBWH026/2021\|EPI_ISL_11817026\|2021-01-22 |
| >lcl\|Query_54391:5304-5633 | hRSV/A/Australia/QLD-RBWH022/2021\|EPI_ISL_11817025\|2021-01-18 |
| >lcl\|Query_54389:5304-5633 | hRSV/A/Australia/QLD-RBWH020/2021\|EPI_ISL_11817023\|2021-01-17 |
| >lcl\|Query_54298:5277-5606 | hRSV/A/Australia/VIC-RCH050/2021\|EPI_ISL_2543806\|2021-03-08 |
| >lcl\|Query_5993:5277-5606 | hRSV/A/Australia/VIC-RCH071/2021\|EPI_ISL_11817092\|2021-03-27 |
| >lcl\|Query_5990:5302-5631 | hRSV/A/Australia/QLD-RBWH278/2021\|EPI_ISL_11817078\|2021-04-01 |
| >lcl\|Query_5988:5304-5633 | hRSV/A/Australia/QLD-RBWH264/2021\|EPI_ISL_11817075\|2021-03-27 |
| >lcl\|Query_5987:5304-5633 | hRSV/A/Australia/QLD-RBWH244/2021\|EPI_ISL_11817072\|2021-03-18 |
| >lcl\|Query_5982:5303-5632 | hRSV/A/Australia/QLD-RBWH206/2021\|EPI_ISL_11817065\|2021-03-10 |
| >lcl\|Query_5975:5304-5633 | hRSV/A/Australia/QLD-RBWH075/2021\|EPI_ISL_11817046\|2021-02-09 |
| >lcl\|Query_5973:5304-5633 | hRSV/A/Australia/QLD-RBWH068/2021\|EPI_ISL_11817043\|2021-02-07 |
| >lcl\|Query_5963:5304-5633 | hRSV/A/Australia/QLD-RBWH026/2021\|EPI_ISL_11817026\|2021-01-22 |
| >lcl\|Query_5962:5304-5633 | hRSV/A/Australia/QLD-RBWH022/2021\|EPI_ISL_11817025\|2021-01-18 |
| >lcl\|Query_5960:5304-5633 | hRSV/A/Australia/QLD-RBWH020/2021\|EPI_ISL_11817023\|2021-01-17 |
| >lcl\|Query_5869:5277-5606 | hRSV/A/Australia/VIC-RCH050/2021\|EPI_ISL_2543806\|2021-03-08 |
| >lcl\|Query_54418:5304-5633 | hRSV/A/Australia/QLD-RBWH273/2021\|EPI_ISL_11817076\|2021-03-30 |
| >lcl\|Query_54414:5304-5633 | hRSV/A/Australia/QLD-RBWH229/2021\|EPI_ISL_11817070\|2021-03-15 |
| >lcl\|Query_54413:5304-5633 | hRSV/A/Australia/QLD-RBWH227/2021\|EPI_ISL_11817069\|2021-03-15 |
| >lcl\|Query_5989:5304-5633 | hRSV/A/Australia/QLD-RBWH273/2021\|EPI_ISL_11817076\|2021-03-30 |
| >lcl\|Query_5985:5304-5633 | hRSV/A/Australia/QLD-RBWH229/2021\|EPI_ISL_11817070\|2021-03-15 |
| >lcl\|Query_5984:5304-5633 | hRSV/A/Australia/QLD-RBWH227/2021\|EPI_ISL_11817069\|2021-03-15 |
| >lcl\|Query_54409:5304-5633 | hRSV/A/Australia/QLD-RBWH185/2021\|EPI_ISL_11817061\|2021-03-05 |
| >lcl\|Query_54399:5304-5633 | hRSV/A/Australia/QLD-RBWH060/2021\|EPI_ISL_11817039\|2021-02-04 |
| >lcl\|Query_5980:5304-5633 | hRSV/A/Australia/QLD-RBWH185/2021\|EPI_ISL_11817061\|2021-03-05 |
| >lcl\|Query_5970:5304-5633 | hRSV/A/Australia/QLD-RBWH060/2021\|EPI_ISL_11817039\|2021-02-04 |
| >lcl\|Query_54400:5304-5633 | hRSV/A/Australia/QLD-RBWH065/2021\|EPI_ISL_11817041\|2021-02-05 |
| >lcl\|Query_5971:5304-5633 | hRSV/A/Australia/QLD-RBWH065/2021\|EPI_ISL_11817041\|2021-02-05 |
| >lcl\|Query_54388:5304-5633 | hRSV/A/Australia/QLD-RBWH015/2021\|EPI_ISL_11817021\|2021-01-13 |
| >lcl\|Query_5959:5304-5633 | hRSV/A/Australia/QLD-RBWH015/2021\|EPI_ISL_11817021\|2021-01-13 |
| >lcl\|Query_54468:5259-5588 | hRSV/A/Argentina/BA-HNRG-415/2018\|EPI_ISL_15067677\|2018-06-28 |
| >lcl\|Query_54255:5299-5628 | hRSV/A/Australia/VIC-RCH138/2017\|EPI_ISL_1834125\|2017-09-20 |
| >lcl\|Query_54239:5281-5610 | hRSV/A/Australia/VIC-RCH100/2017\|EPI_ISL_1834107\|2017-08-01 |
| >lcl\|Query_54167:5259-5588 | hRSV/A/Argentina/HNRG-415/2018\|EPI_ISL_15055312\|2018-06-28 |
| >lcl\|Query_6039:5259-5588 | hRSV/A/Argentina/BA-HNRG-415/2018\|EPI_ISL_15067677\|2018-06-28 |
| >lcl\|Query_5826:5299-5628 | hRSV/A/Australia/VIC-RCH138/2017\|EPI_ISL_1834125\|2017-09-20 |
| >lcl\|Query_5810:5281-5610 | hRSV/A/Australia/VIC-RCH100/2017\|EPI_ISL_1834107\|2017-08-01 |
| >lcl\|Query_5738:5259-5588 | hRSV/A/Argentina/HNRG-415/2018\|EPI_ISL_15055312\|2018-06-28 |
| >lcl\|Query_54502:5285-5614 | hRSV/A/England/RE19003253/2019\|EPI_ISL_1647406\|2019-12-15 |
| >lcl\|Query_54232:5310-5639 | hRSV/A/Australia/VIC-RCH070/2019\|EPI_ISL_1834100\|2019-05-18 |
| >lcl\|Query_54210:5268-5597 | hRSV/A/England/RE20000002/2019\|EPI_ISL_1520436\|2019-12-17 |
| >lcl\|Query_54206:5283-5612 | hRSV/A/England/RE19003255/2019\|EPI_ISL_1520432\|2019-12-13 |
| >lcl\|Query_6073:5285-5614 | hRSV/A/England/RE19003253/2019\|EPI_ISL_1647406\|2019-12-15 |
| >lcl\|Query_5803:5310-5639 | hRSV/A/Australia/VIC-RCH070/2019\|EPI_ISL_1834100\|2019-05-18 |
| >lcl\|Query_5781:5268-5597 | hRSV/A/England/RE20000002/2019\|EPI_ISL_1520436\|2019-12-17 |
| >lcl\|Query_5777:5283-5612 | hRSV/A/England/RE19003255/2019\|EPI_ISL_1520432\|2019-12-13 |
| >lcl\|Query_54248:5281-5610 | hRSV/A/Australia/VIC-RCH120/2017\|EPI_ISL_1834117\|2017-08-20 |
| >lcl\|Query_5819:5281-5610 | hRSV/A/Australia/VIC-RCH120/2017\|EPI_ISL_1834117\|2017-08-20 |
| >lcl\|Query_54221:5274-5603 | hRSV/A/England/194840215/2019\|EPI_ISL_1647389\|2019-11-25 |
| >lcl\|Query_5792:5274-5603 | hRSV/A/England/194840215/2019\|EPI_ISL_1647389\|2019-11-25 |
| >lcl\|Query_54466:5257-5586 | hRSV/A/Argentina/BA-HNRG-403/2018\|EPI_ISL_15067675\|2018-05-04 |
| >lcl\|Query_54165:5257-5586 | hRSV/A/Argentina/HNRG-403/2018\|EPI_ISL_15055310\|2018-05-04 |
| >lcl\|Query_6037:5257-5586 | hRSV/A/Argentina/BA-HNRG-403/2018\|EPI_ISL_15067675\|2018-05-04 |
| >lcl\|Query_5736:5257-5586 | hRSV/A/Argentina/HNRG-403/2018\|EPI_ISL_15055310\|2018-05-04 |
| >lcl\|Query_54470:5257-5586 | hRSV/A/Argentina/BA-HNRG-419/2018\|EPI_ISL_15067679\|2018-07-11 |
| >lcl\|Query_54169:5257-5586 | hRSV/A/Argentina/HNRG-419/2018\|EPI_ISL_15055314\|2018-07-11 |
| >lcl\|Query_6041:5257-5586 | hRSV/A/Argentina/BA-HNRG-419/2018\|EPI_ISL_15067679\|2018-07-11 |
| >lcl\|Query_5740:5257-5586 | hRSV/A/Argentina/HNRG-419/2018\|EPI_ISL_15055314\|2018-07-11 |
| >lcl\|Query_54271:5281-5610 | hRSV/A/Australia/VIC-RCH180/2018\|EPI_ISL_1834141\|2018-11-30 |
| >lcl\|Query_54265:5281-5610 | hRSV/A/Australia/VIC-RCH158/2019\|EPI_ISL_1834135\|2019-08-18 |
| >lcl\|Query_54261:5281-5610 | hRSV/A/Australia/VIC-RCH145/2018\|EPI_ISL_1834131\|2018-07-21 |
| >lcl\|Query_54247:5281-5610 | hRSV/A/Australia/VIC-RCH119/2018\|EPI_ISL_1834116\|2018-07-02 |
| >lcl\|Query_5842:5281-5610 | hRSV/A/Australia/VIC-RCH180/2018\|EPI_ISL_1834141\|2018-11-30 |
| >lcl\|Query_5836:5281-5610 | hRSV/A/Australia/VIC-RCH158/2019\|EPI_ISL_1834135\|2019-08-18 |
| >lcl\|Query_5832:5281-5610 | hRSV/A/Australia/VIC-RCH145/2018\|EPI_ISL_1834131\|2018-07-21 |
| >lcl\|Query_5818:5281-5610 | hRSV/A/Australia/VIC-RCH119/2018\|EPI_ISL_1834116\|2018-07-02 |
| >lcl\|Query_54259:5282-5611 | hRSV/A/Australia/VIC-RCH142/2019\|EPI_ISL_1834129\|2019-08-12 |
| >lcl\|Query_5830:5282-5611 | hRSV/A/Australia/VIC-RCH142/2019\|EPI_ISL_1834129\|2019-08-12 |
| >lcl\|Query_54366:5324-5653 | hRSV/A/Australia/VIC-VIDRL002/2018\|EPI_ISL_4602779\|2018-07-25 |
| >lcl\|Query_54257:5281-5610 | hRSV/A/Australia/VIC-RCH140/2018\|EPI_ISL_1834127\|2018-07-22 |
| >lcl\|Query_54254:5281-5610 | hRSV/A/Australia/VIC-RCH137/2018\|EPI_ISL_1834124\|2018-07-16 |
| >lcl\|Query_54237:5281-5610 | hRSV/A/Australia/VIC-RCH088/2018\|EPI_ISL_1834105\|2018-06-11 |
| >lcl\|Query_54234:5281-5610 | hRSV/A/Australia/VIC-RCH073/2018\|EPI_ISL_1834102\|2018-04-30 |
| >lcl\|Query_5937:5324-5653 | hRSV/A/Australia/VIC-VIDRL002/2018\|EPI_ISL_4602779\|2018-07-25 |
| >lcl\|Query_5828:5281-5610 | hRSV/A/Australia/VIC-RCH140/2018\|EPI_ISL_1834127\|2018-07-22 |
| >lcl\|Query_5825:5281-5610 | hRSV/A/Australia/VIC-RCH137/2018\|EPI_ISL_1834124\|2018-07-16 |
| >lcl\|Query_5808:5281-5610 | hRSV/A/Australia/VIC-RCH088/2018\|EPI_ISL_1834105\|2018-06-11 |
| >lcl\|Query_5805:5281-5610 | hRSV/A/Australia/VIC-RCH073/2018\|EPI_ISL_1834102\|2018-04-30 |
| >lcl\|Query_54262:5281-5610 | hRSV/A/Australia/VIC-RCH149/2018\|EPI_ISL_1834132\|2018-07-30 |
| >lcl\|Query_5833:5281-5610 | hRSV/A/Australia/VIC-RCH149/2018\|EPI_ISL_1834132\|2018-07-30 |
| >lcl\|Query_54258:5281-5610 | hRSV/A/Australia/VIC-RCH141/2018\|EPI_ISL_1834128\|2018-07-22 |
| >lcl\|Query_54252:5281-5610 | hRSV/A/Australia/VIC-RCH133/2018\|EPI_ISL_1834121\|2018-07-14 |
| >lcl\|Query_5829:5281-5610 | hRSV/A/Australia/VIC-RCH141/2018\|EPI_ISL_1834128\|2018-07-22 |
| >lcl\|Query_5823:5281-5610 | hRSV/A/Australia/VIC-RCH133/2018\|EPI_ISL_1834121\|2018-07-14 |
| >lcl\|Query_54253:5281-5610 | hRSV/A/Australia/VIC-RCH135/2018\|EPI_ISL_1834123\|2018-07-14 |
| >lcl\|Query_5824:5281-5610 | hRSV/A/Australia/VIC-RCH135/2018\|EPI_ISL_1834123\|2018-07-14 |
| >lcl\|Query_54515:5263-5592 | hRSV/A/England/RS20000579/2020\|EPI_ISL_1647419\|2020-02-16 |
| >lcl\|Query_6086:5263-5592 | hRSV/A/England/RS20000579/2020\|EPI_ISL_1647419\|2020-02-16 |
| >lcl\|Query_54250:5299-5628 | hRSV/A/Australia/VIC-RCH126/2017\|EPI_ISL_1834119\|2017-08-29 |
| >lcl\|Query_54244:5281-5610 | hRSV/A/Australia/VIC-RCH113/2018\|EPI_ISL_1834113\|2018-07-01 |
| >lcl\|Query_5821:5299-5628 | hRSV/A/Australia/VIC-RCH126/2017\|EPI_ISL_1834119\|2017-08-29 |
| >lcl\|Query_5815:5281-5610 | hRSV/A/Australia/VIC-RCH113/2018\|EPI_ISL_1834113\|2018-07-01 |
| >lcl\|Query_54518:5286-5615 | hRSV/A/England/RE19003244/2019\|EPI_ISL_1647422\|2020-12-16 |
| >lcl\|Query_54509:5258-5587 | hRSV/A/England/RE20000025/2020\|EPI_ISL_1647413\|2020-01-09 |
| >lcl\|Query_54508:5285-5614 | hRSV/A/England/RE20000023/2020\|EPI_ISL_1647412\|2020-01-06 |
| >lcl\|Query_54505:5285-5614 | hRSV/A/England/RE20000014/2020\|EPI_ISL_1647409\|2020-01-02 |
| >lcl\|Query_54503:5271-5600 | hRSV/A/England/RE20000010/2019\|EPI_ISL_1647407\|2019-12-29 |
| >lcl\|Query_54501:5286-5615 | hRSV/A/England/RE19003244/2019\|EPI_ISL_1647405\|2019-12-06 |
| >lcl\|Query_54498:5299-5628 | hRSV/A/England/RE19003150/2019\|EPI_ISL_1647402\|2019-11-15 |
| >lcl\|Query_54497:5286-5615 | hRSV/A/England/RE19003148/2019\|EPI_ISL_1647401\|2019-11-20 |
| >lcl\|Query_54230:5284-5613 | hRSV/A/England/RE19003140/2019\|EPI_ISL_1647398\|2019-11-04 |
| >lcl\|Query_54229:5284-5613 | hRSV/A/England/RE19003139/2019\|EPI_ISL_1647397\|2019-12-01 |
| >lcl\|Query_54227:5285-5614 | hRSV/A/England/RE19003127/2019\|EPI_ISL_1647395\|2019-11-27 |
| >lcl\|Query_54222:5285-5614 | hRSV/A/England/195020407/2019\|EPI_ISL_1647390\|2019-12-06 |
| >lcl\|Query_54215:5282-5611 | hRSV/A/England/194380302/2019\|EPI_ISL_1647383\|2019-10-22 |
| >lcl\|Query_54207:5283-5612 | hRSV/A/England/RE19003256/2019\|EPI_ISL_1520433\|2019-12-17 |
| >lcl\|Query_54205:5284-5613 | hRSV/A/England/RE19003243/2019\|EPI_ISL_1520431\|2019-12-06 |
| >lcl\|Query_54204:5283-5612 | hRSV/A/England/RE19003240/2019\|EPI_ISL_1520430\|2019-12-12 |
| >lcl\|Query_54199:5244-5573 | hRSV/A/England/RE19003145/2019\|EPI_ISL_1520425\|2019-11-29 |
| >lcl\|Query_54198:5285-5614 | hRSV/A/England/RE19003141/2019\|EPI_ISL_1520424\|2019-12-05 |
| >lcl\|Query_54197:5284-5613 | hRSV/A/England/RE19003138/2019\|EPI_ISL_1520423\|2019-12-02 |
| >lcl\|Query_54195:5285-5614 | hRSV/A/England/RE19003133/2019\|EPI_ISL_1520421\|2019-11-25 |
| >lcl\|Query_54194:5284-5613 | hRSV/A/England/RE19003132/2019\|EPI_ISL_1520420\|2019-11-23 |
| >lcl\|Query_54193:5282-5611 | hRSV/A/England/RE19003131/2019\|EPI_ISL_1520419\|2019-10-21 |
| >lcl\|Query_54192:5283-5612 | hRSV/A/England/RE19003130/2019\|EPI_ISL_1520418\|2019-12-04 |
| >lcl\|Query_54189:5282-5611 | hRSV/A/England/RE19003124/2019\|EPI_ISL_1520415\|2019-11-30 |
| >lcl\|Query_6089:5286-5615 | hRSV/A/England/RE19003244/2019\|EPI_ISL_1647422\|2020-12-16 |
| >lcl\|Query_6080:5258-5587 | hRSV/A/England/RE20000025/2020\|EPI_ISL_1647413\|2020-01-09 |
| >lcl\|Query_6079:5285-5614 | hRSV/A/England/RE20000023/2020\|EPI_ISL_1647412\|2020-01-06 |
| >lcl\|Query_6076:5285-5614 | hRSV/A/England/RE20000014/2020\|EPI_ISL_1647409\|2020-01-02 |
| >lcl\|Query_6074:5271-5600 | hRSV/A/England/RE20000010/2019\|EPI_ISL_1647407\|2019-12-29 |
| >lcl\|Query_6072:5286-5615 | hRSV/A/England/RE19003244/2019\|EPI_ISL_1647405\|2019-12-06 |
| >lcl\|Query_6069:5299-5628 | hRSV/A/England/RE19003150/2019\|EPI_ISL_1647402\|2019-11-15 |
| >lcl\|Query_6068:5286-5615 | hRSV/A/England/RE19003148/2019\|EPI_ISL_1647401\|2019-11-20 |
| >lcl\|Query_5801:5284-5613 | hRSV/A/England/RE19003140/2019\|EPI_ISL_1647398\|2019-11-04 |
| >lcl\|Query_5800:5284-5613 | hRSV/A/England/RE19003139/2019\|EPI_ISL_1647397\|2019-12-01 |
| >lcl\|Query_5798:5285-5614 | hRSV/A/England/RE19003127/2019\|EPI_ISL_1647395\|2019-11-27 |
| >lcl\|Query_5793:5285-5614 | hRSV/A/England/195020407/2019\|EPI_ISL_1647390\|2019-12-06 |
| >lcl\|Query_5786:5282-5611 | hRSV/A/England/194380302/2019\|EPI_ISL_1647383\|2019-10-22 |
| >lcl\|Query_5778:5283-5612 | hRSV/A/England/RE19003256/2019\|EPI_ISL_1520433\|2019-12-17 |
| >lcl\|Query_5776:5284-5613 | hRSV/A/England/RE19003243/2019\|EPI_ISL_1520431\|2019-12-06 |
| >lcl\|Query_5775:5283-5612 | hRSV/A/England/RE19003240/2019\|EPI_ISL_1520430\|2019-12-12 |
| >lcl\|Query_5770:5244-5573 | hRSV/A/England/RE19003145/2019\|EPI_ISL_1520425\|2019-11-29 |
| >lcl\|Query_5769:5285-5614 | hRSV/A/England/RE19003141/2019\|EPI_ISL_1520424\|2019-12-05 |
| >lcl\|Query_5768:5284-5613 | hRSV/A/England/RE19003138/2019\|EPI_ISL_1520423\|2019-12-02 |
| >lcl\|Query_5766:5285-5614 | hRSV/A/England/RE19003133/2019\|EPI_ISL_1520421\|2019-11-25 |
| >lcl\|Query_5765:5284-5613 | hRSV/A/England/RE19003132/2019\|EPI_ISL_1520420\|2019-11-23 |
| >lcl\|Query_5764:5282-5611 | hRSV/A/England/RE19003131/2019\|EPI_ISL_1520419\|2019-10-21 |
| >lcl\|Query_5763:5283-5612 | hRSV/A/England/RE19003130/2019\|EPI_ISL_1520418\|2019-12-04 |
| >lcl\|Query_5760:5282-5611 | hRSV/A/England/RE19003124/2019\|EPI_ISL_1520415\|2019-11-30 |
| >lcl\|Query_54276:5281-5610 | hRSV/A/Australia/VIC-RCH187/2018\|EPI_ISL_1834146\|2018-11-11 |
| >lcl\|Query_54272:5281-5610 | hRSV/A/Australia/VIC-RCH181/2018\|EPI_ISL_1834142\|2018-10-06 |
| >lcl\|Query_5847:5281-5610 | hRSV/A/Australia/VIC-RCH187/2018\|EPI_ISL_1834146\|2018-11-11 |
| >lcl\|Query_5843:5281-5610 | hRSV/A/Australia/VIC-RCH181/2018\|EPI_ISL_1834142\|2018-10-06 |
| >lcl\|Query_54500:5285-5614 | hRSV/A/England/RE19003238/2019\|EPI_ISL_1647404\|2019-12-02 |
| >lcl\|Query_6071:5285-5614 | hRSV/A/England/RE19003238/2019\|EPI_ISL_1647404\|2019-12-02 |
| >lcl\|Query_54228:5286-5615 | hRSV/A/England/RE19003136/2019\|EPI_ISL_1647396\|2019-11-16 |
| >lcl\|Query_54226:5285-5614 | hRSV/A/England/RE19003126/2019\|EPI_ISL_1647394\|2019-11-15 |
| >lcl\|Query_54200:5279-5608 | hRSV/A/England/RE19003149/2019\|EPI_ISL_1520426\|2019-11-22 |
| >lcl\|Query_54196:5273-5602 | hRSV/A/England/RE19003137/2019\|EPI_ISL_1520422\|2019-12-04 |
| >lcl\|Query_54190:5285-5614 | hRSV/A/England/RE19003125/2019\|EPI_ISL_1520416\|2019-11-13 |
| >lcl\|Query_54187:5285-5614 | hRSV/A/England/RE19003122/2019\|EPI_ISL_1520413\|2019-11-12 |
| >lcl\|Query_54146:5286-5615 | hRSV/A/England/E19003242/2019\|EPI_ISL_11428328\|2019-12-16 |
| >lcl\|Query_5799:5286-5615 | hRSV/A/England/RE19003136/2019\|EPI_ISL_1647396\|2019-11-16 |
| >lcl\|Query_5797:5285-5614 | hRSV/A/England/RE19003126/2019\|EPI_ISL_1647394\|2019-11-15 |
| >lcl\|Query_5771:5279-5608 | hRSV/A/England/RE19003149/2019\|EPI_ISL_1520426\|2019-11-22 |
| >lcl\|Query_5767:5273-5602 | hRSV/A/England/RE19003137/2019\|EPI_ISL_1520422\|2019-12-04 |
| >lcl\|Query_5761:5285-5614 | hRSV/A/England/RE19003125/2019\|EPI_ISL_1520416\|2019-11-13 |
| >lcl\|Query_5758:5285-5614 | hRSV/A/England/RE19003122/2019\|EPI_ISL_1520413\|2019-11-12 |
| >lcl\|Query_5717:5286-5615 | hRSV/A/England/E19003242/2019\|EPI_ISL_11428328\|2019-12-16 |
| >lcl\|Query_54202:5285-5614 | hRSV/A/England/RE19003236/2019\|EPI_ISL_1520428\|2019-12-05 |
| >lcl\|Query_5773:5285-5614 | hRSV/A/England/RE19003236/2019\|EPI_ISL_1520428\|2019-12-05 |
| >lcl\|Query_54475:5259-5588 | hRSV/A/Argentina/BA-HNRG-437/2019\|EPI_ISL_15067684\|2019-06-19 |
| >lcl\|Query_54174:5259-5588 | hRSV/A/Argentina/HNRG-437/2019\|EPI_ISL_15055319\|2019-06-19 |
| >lcl\|Query_6046:5259-5588 | hRSV/A/Argentina/BA-HNRG-437/2019\|EPI_ISL_15067684\|2019-06-19 |
| >lcl\|Query_5745:5259-5588 | hRSV/A/Argentina/HNRG-437/2019\|EPI_ISL_15055319\|2019-06-19 |
| >lcl\|Query_54493:5262-5591 | hRSV/A/England/195200782/2019\|EPI_ISL_1520408\|2019-12-20 |
| >lcl\|Query_6064:5262-5591 | hRSV/A/England/195200782/2019\|EPI_ISL_1520408\|2019-12-20 |
| >lcl\|Query_54280:5248-5577 | hRSV/A/England/194580267/2019\|EPI_ISL_1834150\|2019-11-06 |
| >lcl\|Query_5851:5248-5577 | hRSV/A/England/194580267/2019\|EPI_ISL_1834150\|2019-11-06 |
| >lcl\|Query_54263:5281-5610 | hRSV/A/Australia/VIC-RCH150/2018\|EPI_ISL_1834133\|2018-07-28 |
| >lcl\|Query_54249:5281-5610 | hRSV/A/Australia/VIC-RCH125/2018\|EPI_ISL_1834118\|2018-07-08 |
| >lcl\|Query_5834:5281-5610 | hRSV/A/Australia/VIC-RCH150/2018\|EPI_ISL_1834133\|2018-07-28 |
| >lcl\|Query_5820:5281-5610 | hRSV/A/Australia/VIC-RCH125/2018\|EPI_ISL_1834118\|2018-07-08 |
| >lcl\|Query_54378:5283-5612 | hRSV/A/Argentina/BA-HNRG-324/2017\|EPI_ISL_1074245\|2017-05-24 |
| >lcl\|Query_54125:5293-5622 | hRSV/A/Argentina/BA-HNRG-376/2017\|EPI_ISL_1074127\|2017-06-30 |
| >lcl\|Query_5949:5283-5612 | hRSV/A/Argentina/BA-HNRG-324/2017\|EPI_ISL_1074245\|2017-05-24 |
| >lcl\|Query_5696:5293-5622 | hRSV/A/Argentina/BA-HNRG-376/2017\|EPI_ISL_1074127\|2017-06-30 |
| >lcl\|Query_54374:5286-5615 | hRSV/A/Argentina/BA-HNRG-347/2017\|EPI_ISL_1074212\|2017-06-12 |
| >lcl\|Query_54122:5291-5620 | hRSV/A/Argentina/BA-HNRG-365/2017\|EPI_ISL_1074115\|2017-06-22 |
| >lcl\|Query_54121:5290-5619 | hRSV/A/Argentina/BA-HNRG-382/2017\|EPI_ISL_1074112\|2017-07-05 |
| >lcl\|Query_5945:5286-5615 | hRSV/A/Argentina/BA-HNRG-347/2017\|EPI_ISL_1074212\|2017-06-12 |
| >lcl\|Query_5693:5291-5620 | hRSV/A/Argentina/BA-HNRG-365/2017\|EPI_ISL_1074115\|2017-06-22 |
| >lcl\|Query_5692:5290-5619 | hRSV/A/Argentina/BA-HNRG-382/2017\|EPI_ISL_1074112\|2017-07-05 |
| >lcl\|Query_54201:5278-5607 | hRSV/A/England/RE19003153/2019\|EPI_ISL_1520427\|2019-11-22 |
| >lcl\|Query_5772:5278-5607 | hRSV/A/England/RE19003153/2019\|EPI_ISL_1520427\|2019-11-22 |
| >lcl\|Query_54119:5293-5622 | hRSV/A/Argentina/BA-HNRG-340/2017\|EPI_ISL_1074103\|2017-06-05 |
| >lcl\|Query_5690:5293-5622 | hRSV/A/Argentina/BA-HNRG-340/2017\|EPI_ISL_1074103\|2017-06-05 |
| >lcl\|Query_54496:5285-5614 | hRSV/A/England/RE19003147/2019\|EPI_ISL_1647400\|2019-12-02 |
| >lcl\|Query_6067:5285-5614 | hRSV/A/England/RE19003147/2019\|EPI_ISL_1647400\|2019-12-02 |
| >lcl\|Query_54188:5284-5613 | hRSV/A/England/RE19003123/2019\|EPI_ISL_1520414\|2019-11-17 |
| >lcl\|Query_5759:5284-5613 | hRSV/A/England/RE19003123/2019\|EPI_ISL_1520414\|2019-11-17 |
| >lcl\|Query_54469:5257-5586 | hRSV/A/Argentina/BA-HNRG-416/2018\|EPI_ISL_15067678\|2018-06-29 |
| >lcl\|Query_54168:5257-5586 | hRSV/A/Argentina/HNRG-416/2018\|EPI_ISL_15055313\|2018-06-29 |
| >lcl\|Query_6040:5257-5586 | hRSV/A/Argentina/BA-HNRG-416/2018\|EPI_ISL_15067678\|2018-06-29 |
| >lcl\|Query_5739:5257-5586 | hRSV/A/Argentina/HNRG-416/2018\|EPI_ISL_15055313\|2018-06-29 |
| >lcl\|Query_54415:5304-5633 | hRSV/A/Australia/QLD-RBWH230/2021\|EPI_ISL_11817071\|2021-03-15 |
| >lcl\|Query_54401:5304-5633 | hRSV/A/Australia/QLD-RBWH067/2021\|EPI_ISL_11817042\|2021-02-07 |
| >lcl\|Query_54386:5304-5633 | hRSV/A/Australia/QLD-RBWH008/2021\|EPI_ISL_11817019\|2021-01-08 |
| >lcl\|Query_5986:5304-5633 | hRSV/A/Australia/QLD-RBWH230/2021\|EPI_ISL_11817071\|2021-03-15 |
| >lcl\|Query_5972:5304-5633 | hRSV/A/Australia/QLD-RBWH067/2021\|EPI_ISL_11817042\|2021-02-07 |
| >lcl\|Query_5957:5304-5633 | hRSV/A/Australia/QLD-RBWH008/2021\|EPI_ISL_11817019\|2021-01-08 |
| >lcl\|Query_54376:5291-5620 | hRSV/A/Argentina/BA-HNRG-385/2017\|EPI_ISL_1074214\|2017-07-10 |
| >lcl\|Query_5947:5291-5620 | hRSV/A/Argentina/BA-HNRG-385/2017\|EPI_ISL_1074214\|2017-07-10 |
| >lcl\|Query_54407:5304-5633 | hRSV/A/Australia/QLD-RBWH130/2021\|EPI_ISL_11817053\|2021-02-18 |
| >lcl\|Query_5978:5304-5633 | hRSV/A/Australia/QLD-RBWH130/2021\|EPI_ISL_11817053\|2021-02-18 |
| >lcl\|Query_54397:5304-5633 | hRSV/A/Australia/QLD-RBWH053/2021\|EPI_ISL_11817036\|2021-02-02 |
| >lcl\|Query_54149:5304-5633 | hRSV/A/Australia/QLD-RBWH287/2021\|EPI_ISL_11817083\|2021-04-06 |
| >lcl\|Query_5968:5304-5633 | hRSV/A/Australia/QLD-RBWH053/2021\|EPI_ISL_11817036\|2021-02-02 |
| >lcl\|Query_5720:5304-5633 | hRSV/A/Australia/QLD-RBWH287/2021\|EPI_ISL_11817083\|2021-04-06 |
| >lcl\|Query_54482:5259-5588 | hRSV/A/Argentina/BA-HNRG-452/2021\|EPI_ISL_15067691\|2021-08-03 |
| >lcl\|Query_54181:5259-5588 | hRSV/A/Argentina/HNRG-452/2021\|EPI_ISL_15055326\|2021-08-03 |
| >lcl\|Query_6053:5259-5588 | hRSV/A/Argentina/BA-HNRG-452/2021\|EPI_ISL_15067691\|2021-08-03 |
| >lcl\|Query_5752:5259-5588 | hRSV/A/Argentina/HNRG-452/2021\|EPI_ISL_15055326\|2021-08-03 |
| >lcl\|Query_54472:5260-5589 | hRSV/A/Argentina/BA-HNRG-433/2019\|EPI_ISL_15067681\|2019-06-06 |
| >lcl\|Query_54171:5260-5589 | hRSV/A/Argentina/HNRG-433/2019\|EPI_ISL_15055316\|2019-06-06 |
| >lcl\|Query_6043:5260-5589 | hRSV/A/Argentina/BA-HNRG-433/2019\|EPI_ISL_15067681\|2019-06-06 |
| >lcl\|Query_5742:5260-5589 | hRSV/A/Argentina/HNRG-433/2019\|EPI_ISL_15055316\|2019-06-06 |
| >lcl\|Query_54242:5282-5611 | hRSV/A/Australia/VIC-RCH107/2019\|EPI_ISL_1834110\|2019-07-07 |
| >lcl\|Query_54233:5283-5612 | hRSV/A/Australia/VIC-RCH072/2019\|EPI_ISL_1834101\|2019-05-13 |
| >lcl\|Query_5813:5282-5611 | hRSV/A/Australia/VIC-RCH107/2019\|EPI_ISL_1834110\|2019-07-07 |
| >lcl\|Query_5804:5283-5612 | hRSV/A/Australia/VIC-RCH072/2019\|EPI_ISL_1834101\|2019-05-13 |
| >lcl\|Query_54364:5229-5558 | hRSV/A/Australia/2001479/2020\|EPI_ISL_2839408\|2020-10-21 |
| >lcl\|Query_54341:5313-5642 | hRSV/A/Australia/1514/2021\|EPI_ISL_2835616\|2021-01-18 |
| >lcl\|Query_5935:5229-5558 | hRSV/A/Australia/2001479/2020\|EPI_ISL_2839408\|2020-10-21 |
| >lcl\|Query_5912:5313-5642 | hRSV/A/Australia/1514/2021\|EPI_ISL_2835616\|2021-01-18 |
| >lcl\|Query_54365:5229-5558 | hRSV/A/Australia/7003912/2020\|EPI_ISL_2839409\|2020-11-17 |
| >lcl\|Query_54362:5229-5558 | hRSV/A/Australia/6003147/2020\|EPI_ISL_2839406\|2020-12-06 |
| >lcl\|Query_54361:5229-5558 | hRSV/A/Australia/4009301/2020\|EPI_ISL_2839405\|2020-12-04 |
| >lcl\|Query_5936:5229-5558 | hRSV/A/Australia/7003912/2020\|EPI_ISL_2839409\|2020-11-17 |
| >lcl\|Query_5933:5229-5558 | hRSV/A/Australia/6003147/2020\|EPI_ISL_2839406\|2020-12-06 |
| >lcl\|Query_5932:5229-5558 | hRSV/A/Australia/4009301/2020\|EPI_ISL_2839405\|2020-12-04 |
| >lcl\|Query_54360:5309-5638 | hRSV/A/Australia/3002687/2021\|EPI_ISL_2839404\|2021-01-01 |
| >lcl\|Query_54359:5304-5633 | hRSV/A/Australia/1010209/2021\|EPI_ISL_2839403\|2021-01-11 |
| >lcl\|Query_54358:5313-5642 | hRSV/A/Australia/3004/2021\|EPI_ISL_2839402\|2021-01-09 |
| >lcl\|Query_54357:5312-5641 | hRSV/A/Australia/5009934/2021\|EPI_ISL_2839401\|2021-01-04 |
| >lcl\|Query_54356:5313-5642 | hRSV/A/Australia/3002650/2021\|EPI_ISL_2839400\|2021-01-01 |
| >lcl\|Query_5931:5309-5638 | hRSV/A/Australia/3002687/2021\|EPI_ISL_2839404\|2021-01-01 |
| >lcl\|Query_5930:5304-5633 | hRSV/A/Australia/1010209/2021\|EPI_ISL_2839403\|2021-01-11 |
| >lcl\|Query_5929:5313-5642 | hRSV/A/Australia/3004/2021\|EPI_ISL_2839402\|2021-01-09 |
| >lcl\|Query_5928:5312-5641 | hRSV/A/Australia/5009934/2021\|EPI_ISL_2839401\|2021-01-04 |
| >lcl\|Query_5927:5313-5642 | hRSV/A/Australia/3002650/2021\|EPI_ISL_2839400\|2021-01-01 |
| >lcl\|Query_54363:5229-5558 | hRSV/A/Australia/9002436/2020\|EPI_ISL_2839407\|2020-11-28 |
| >lcl\|Query_5934:5229-5558 | hRSV/A/Australia/9002436/2020\|EPI_ISL_2839407\|2020-11-28 |
| >lcl\|Query_54264:5281-5610 | hRSV/A/Australia/VIC-RCH151/2018\|EPI_ISL_1834134\|2018-07-29 |
| >lcl\|Query_5835:5281-5610 | hRSV/A/Australia/VIC-RCH151/2018\|EPI_ISL_1834134\|2018-07-29 |
| >lcl\|Query_54478:5259-5588 | hRSV/A/Argentina/BA-HNRG-441/2019\|EPI_ISL_15067687\|2019-07-29 |
| >lcl\|Query_54177:5259-5588 | hRSV/A/Argentina/HNRG-441/2019\|EPI_ISL_15055322\|2019-07-29 |
| >lcl\|Query_6049:5259-5588 | hRSV/A/Argentina/BA-HNRG-441/2019\|EPI_ISL_15067687\|2019-07-29 |
| >lcl\|Query_5748:5259-5588 | hRSV/A/Argentina/HNRG-441/2019\|EPI_ISL_15055322\|2019-07-29 |
| >lcl\|Query_54512:5284-5613 | hRSV/A/England/RE20000100/2020\|EPI_ISL_1647416\|2020-01-26 |
| >lcl\|Query_54489:5237-5566 | hRSV/A/England/195180530/2019\|EPI_ISL_1520404\|2019-12-18 |
| >lcl\|Query_54186:5232-5561 | hRSV/A/England/200260431/2020\|EPI_ISL_1520412\|2020-01-07 |
| >lcl\|Query_6083:5284-5613 | hRSV/A/England/RE20000100/2020\|EPI_ISL_1647416\|2020-01-26 |
| >lcl\|Query_6060:5237-5566 | hRSV/A/England/195180530/2019\|EPI_ISL_1520404\|2019-12-18 |
| >lcl\|Query_5757:5232-5561 | hRSV/A/England/200260431/2020\|EPI_ISL_1520412\|2020-01-07 |
| >lcl\|Query_54441:5238-5567 | hRSV/A/South_Africa/NICD-R04582/2021\|EPI_ISL_12529648\|2021-04-08 |
| >lcl\|Query_6012:5238-5567 | hRSV/A/South_Africa/NICD-R04582/2021\|EPI_ISL_12529648\|2021-04-08 |
| >lcl\|Query_54490:5254-5583 | hRSV/A/England/195180535/2019\|EPI_ISL_1520405\|2019-12-17 |
| >lcl\|Query_54185:5264-5593 | hRSV/A/England/200181040/2019\|EPI_ISL_1520411\|2019-12-30 |
| >lcl\|Query_54144:5214-5543 | hRSV/A/England/200181047/2019\|EPI_ISL_11428301\|2019-12-30 |
| >lcl\|Query_6061:5254-5583 | hRSV/A/England/195180535/2019\|EPI_ISL_1520405\|2019-12-17 |
| >lcl\|Query_5756:5264-5593 | hRSV/A/England/200181040/2019\|EPI_ISL_1520411\|2019-12-30 |
| >lcl\|Query_5715:5214-5543 | hRSV/A/England/200181047/2019\|EPI_ISL_11428301\|2019-12-30 |
| >lcl\|Query_54134:5286-5615 | hRSV/A/Argentina/BA-HNRG-316/2017\|EPI_ISL_1074159\|2017-05-15 |
| >lcl\|Query_5705:5286-5615 | hRSV/A/Argentina/BA-HNRG-316/2017\|EPI_ISL_1074159\|2017-05-15 |
| >lcl\|Query_54281:5261-5590 | hRSV/A/England/194680415/2019\|EPI_ISL_1834151\|2019-11-14 |
| >lcl\|Query_54191:5274-5603 | hRSV/A/England/RE19003129/2019\|EPI_ISL_1520417\|2019-12-01 |
| >lcl\|Query_5852:5261-5590 | hRSV/A/England/194680415/2019\|EPI_ISL_1834151\|2019-11-14 |
| >lcl\|Query_5762:5274-5603 | hRSV/A/England/RE19003129/2019\|EPI_ISL_1520417\|2019-12-01 |
| >lcl\|Query_54123:5283-5612 | hRSV/A/Argentina/BA-HNRG-353/2017\|EPI_ISL_1074120\|2017-06-14 |
| >lcl\|Query_5694:5283-5612 | hRSV/A/Argentina/BA-HNRG-353/2017\|EPI_ISL_1074120\|2017-06-14 |
| >lcl\|Query_54491:5261-5590 | hRSV/A/England/195200142/2019\|EPI_ISL_1520406\|2019-12-18 |
| >lcl\|Query_6062:5261-5590 | hRSV/A/England/195200142/2019\|EPI_ISL_1520406\|2019-12-18 |
| >lcl\|Query_54251:5280-5609 | hRSV/A/Australia/VIC-RCH132/2017\|EPI_ISL_1834120\|2017-09-08 |
| >lcl\|Query_5822:5280-5609 | hRSV/A/Australia/VIC-RCH132/2017\|EPI_ISL_1834120\|2017-09-08 |
| >lcl\|Query_54477:5257-5586 | hRSV/A/Argentina/BA-HNRG-440/2019\|EPI_ISL_15067686\|2019-07-29 |
| >lcl\|Query_54176:5257-5586 | hRSV/A/Argentina/HNRG-440/2019\|EPI_ISL_15055321\|2019-07-29 |
| >lcl\|Query_6048:5257-5586 | hRSV/A/Argentina/BA-HNRG-440/2019\|EPI_ISL_15067686\|2019-07-29 |
| >lcl\|Query_5747:5257-5586 | hRSV/A/Argentina/HNRG-440/2019\|EPI_ISL_15055321\|2019-07-29 |
| >lcl\|Query_54320:5264-5593 | hRSV/A/Mongolia/NCCD006/2018\|EPI_ISL_2543828\|2018-07-10 |
| >lcl\|Query_5891:5264-5593 | hRSV/A/Mongolia/NCCD006/2018\|EPI_ISL_2543828\|2018-07-10 |
| >lcl\|Query_54336:5267-5596 | hRSV/A/Mongolia/NCCD039/2018\|EPI_ISL_2543844\|2018-03-07 |
| >lcl\|Query_54333:5267-5596 | hRSV/A/Mongolia/NCCD021/2018\|EPI_ISL_2543841\|2018-01-31 |
| >lcl\|Query_54324:5264-5593 | hRSV/A/Mongolia/NCCD008/2018\|EPI_ISL_2543832\|2018-01-10 |
| >lcl\|Query_54322:5267-5596 | hRSV/A/Mongolia/NCCD007/2018\|EPI_ISL_2543830\|2018-01-10 |
| >lcl\|Query_5907:5267-5596 | hRSV/A/Mongolia/NCCD039/2018\|EPI_ISL_2543844\|2018-03-07 |
| >lcl\|Query_5904:5267-5596 | hRSV/A/Mongolia/NCCD021/2018\|EPI_ISL_2543841\|2018-01-31 |
| >lcl\|Query_5895:5264-5593 | hRSV/A/Mongolia/NCCD008/2018\|EPI_ISL_2543832\|2018-01-10 |
| >lcl\|Query_5893:5267-5596 | hRSV/A/Mongolia/NCCD007/2018\|EPI_ISL_2543830\|2018-01-10 |
| >lcl\|Query_54384:5294-5623 | hRSV/A/Argentina/BA-HNRG-314/2017\|EPI_ISL_1074259\|2017-05-12 |
| >lcl\|Query_54141:5292-5621 | hRSV/A/Argentina/BA-HNRG-388/2017\|EPI_ISL_1074173\|2017-07-17 |
| >lcl\|Query_54133:5288-5617 | hRSV/A/Argentina/BA-HNRG-315/2017\|EPI_ISL_1074158\|2017-05-15 |
| >lcl\|Query_5955:5294-5623 | hRSV/A/Argentina/BA-HNRG-314/2017\|EPI_ISL_1074259\|2017-05-12 |
| >lcl\|Query_5712:5292-5621 | hRSV/A/Argentina/BA-HNRG-388/2017\|EPI_ISL_1074173\|2017-07-17 |
| >lcl\|Query_5704:5288-5617 | hRSV/A/Argentina/BA-HNRG-315/2017\|EPI_ISL_1074158\|2017-05-15 |
| >lcl\|Query_54381:5290-5619 | hRSV/A/Argentina/BA-HNRG-350/2017\|EPI_ISL_1074255\|2017-06-12 |
| >lcl\|Query_54135:5285-5614 | hRSV/A/Argentina/BA-HNRG-336/2017\|EPI_ISL_1074160\|2017-06-01 |
| >lcl\|Query_54131:5286-5615 | hRSV/A/Argentina/BA-HNRG-343/2017\|EPI_ISL_1074156\|2017-06-07 |
| >lcl\|Query_54120:5285-5614 | hRSV/A/Argentina/BA-HNRG-359/2017\|EPI_ISL_1074106\|2017-06-19 |
| >lcl\|Query_5952:5290-5619 | hRSV/A/Argentina/BA-HNRG-350/2017\|EPI_ISL_1074255\|2017-06-12 |
| >lcl\|Query_5706:5285-5614 | hRSV/A/Argentina/BA-HNRG-336/2017\|EPI_ISL_1074160\|2017-06-01 |
| >lcl\|Query_5702:5286-5615 | hRSV/A/Argentina/BA-HNRG-343/2017\|EPI_ISL_1074156\|2017-06-07 |
| >lcl\|Query_5691:5285-5614 | hRSV/A/Argentina/BA-HNRG-359/2017\|EPI_ISL_1074106\|2017-06-19 |
| >lcl\|Query_54383:5286-5615 | hRSV/A/Argentina/BA-HNRG-370/2017\|EPI_ISL_1074257\|2017-06-26 |
| >lcl\|Query_5954:5286-5615 | hRSV/A/Argentina/BA-HNRG-370/2017\|EPI_ISL_1074257\|2017-06-26 |
| >lcl\|Query_54307:5279-5608 | hRSV/A/Cote_d'Ivoire/IPCI-022/2017\|EPI_ISL_2543815\|2017-09-04 |
| >lcl\|Query_5878:5279-5608 | hRSV/A/Cote_d'Ivoire/IPCI-022/2017\|EPI_ISL_2543815\|2017-09-04 |
| >lcl\|Query_54306:5279-5608 | hRSV/A/Cote_d'Ivoire/IPCI-009/2017\|EPI_ISL_2543814\|2017-08-25 |
| >lcl\|Query_5877:5279-5608 | hRSV/A/Cote_d'Ivoire/IPCI-009/2017\|EPI_ISL_2543814\|2017-08-25 |
| >lcl\|Query_54513:5281-5610 | hRSV/A/England/RE20000101/2020\|EPI_ISL_1647417\|2020-02-07 |
| >lcl\|Query_6084:5281-5610 | hRSV/A/England/RE20000101/2020\|EPI_ISL_1647417\|2020-02-07 |
| >lcl\|Query_54367:5324-5653 | hRSV/A/Cote_d'Ivoire/IPCI-014/2019\|EPI_ISL_5522630\|2019-08-27 |
| >lcl\|Query_5938:5324-5653 | hRSV/A/Cote_d'Ivoire/IPCI-014/2019\|EPI_ISL_5522630\|2019-08-27 |
| >lcl\|Query_54290:5279-5608 | hRSV/A/England/RE20000024/2020\|EPI_ISL_1834172\|2020-01-03 |
| >lcl\|Query_54287:5252-5581 | hRSV/A/England/195140709/2019\|EPI_ISL_1834159\|2019-12-12 |
| >lcl\|Query_5861:5279-5608 | hRSV/A/England/RE20000024/2020\|EPI_ISL_1834172\|2020-01-03 |
| >lcl\|Query_5858:5252-5581 | hRSV/A/England/195140709/2019\|EPI_ISL_1834159\|2019-12-12 |
| >lcl\|Query_54142:5285-5614 | hRSV/A/Argentina/BA-HNRG-342/2017\|EPI_ISL_1074188\|2017-06-06 |
| >lcl\|Query_5713:5285-5614 | hRSV/A/Argentina/BA-HNRG-342/2017\|EPI_ISL_1074188\|2017-06-06 |
| >lcl\|Query_54243:5281-5610 | hRSV/A/Australia/VIC-RCH112/2018\|EPI_ISL_1834112\|2018-06-30 |
| >lcl\|Query_5814:5281-5610 | hRSV/A/Australia/VIC-RCH112/2018\|EPI_ISL_1834112\|2018-06-30 |
| >lcl\|Query_54277:5281-5610 | hRSV/A/Australia/VIC-RCH188/2018\|EPI_ISL_1834147\|2018-11-24 |
| >lcl\|Query_54274:5280-5609 | hRSV/A/Australia/VIC-RCH182/2018\|EPI_ISL_1834144\|2018-10-14 |
| >lcl\|Query_5848:5281-5610 | hRSV/A/Australia/VIC-RCH188/2018\|EPI_ISL_1834147\|2018-11-24 |
| >lcl\|Query_5845:5280-5609 | hRSV/A/Australia/VIC-RCH182/2018\|EPI_ISL_1834144\|2018-10-14 |
| >lcl\|Query_54127:5284-5613 | hRSV/A/Argentina/BA-HNRG-377/2017\|EPI_ISL_1074146\|2017-06-30 |
| >lcl\|Query_5698:5284-5613 | hRSV/A/Argentina/BA-HNRG-377/2017\|EPI_ISL_1074146\|2017-06-30 |
| >lcl\|Query_54124:5294-5623 | hRSV/A/Argentina/BA-HNRG-357/2017\|EPI_ISL_1074126\|2017-06-16 |
| >lcl\|Query_5695:5294-5623 | hRSV/A/Argentina/BA-HNRG-357/2017\|EPI_ISL_1074126\|2017-06-16 |
| >lcl\|Query_54375:5289-5618 | hRSV/A/Argentina/BA-HNRG-366/2017\|EPI_ISL_1074213\|2017-06-23 |
| >lcl\|Query_5946:5289-5618 | hRSV/A/Argentina/BA-HNRG-366/2017\|EPI_ISL_1074213\|2017-06-23 |
| >lcl\|Query_54267:5282-5611 | hRSV/A/Australia/VIC-RCH166/2018\|EPI_ISL_1834137\|2018-08-27 |
| >lcl\|Query_5838:5282-5611 | hRSV/A/Australia/VIC-RCH166/2018\|EPI_ISL_1834137\|2018-08-27 |
| >lcl\|Query_54140:5286-5615 | hRSV/A/Argentina/BA-HNRG-332/2017\|EPI_ISL_1074172\|2017-05-30 |
| >lcl\|Query_5711:5286-5615 | hRSV/A/Argentina/BA-HNRG-332/2017\|EPI_ISL_1074172\|2017-05-30 |
| >lcl\|Query_54499:5285-5614 | hRSV/A/England/RE19003235/2019\|EPI_ISL_1647403\|2019-12-05 |
| >lcl\|Query_6070:5285-5614 | hRSV/A/England/RE19003235/2019\|EPI_ISL_1647403\|2019-12-05 |
| >lcl\|Query_54382:5287-5616 | hRSV/A/Argentina/BA-HNRG-338/2017\|EPI_ISL_1074256\|2017-06-02 |
| >lcl\|Query_54138:5287-5616 | hRSV/A/Argentina/BA-HNRG-323/2017\|EPI_ISL_1074170\|2017-05-22 |
| >lcl\|Query_54128:5292-5621 | hRSV/A/Argentina/BA-HNRG-321/2017\|EPI_ISL_1074152\|2017-05-22 |
| >lcl\|Query_54126:5287-5616 | hRSV/A/Argentina/BA-HNRG-330/2017\|EPI_ISL_1074137\|2017-05-29 |
| >lcl\|Query_5953:5287-5616 | hRSV/A/Argentina/BA-HNRG-338/2017\|EPI_ISL_1074256\|2017-06-02 |
| >lcl\|Query_5709:5287-5616 | hRSV/A/Argentina/BA-HNRG-323/2017\|EPI_ISL_1074170\|2017-05-22 |
| >lcl\|Query_5699:5292-5621 | hRSV/A/Argentina/BA-HNRG-321/2017\|EPI_ISL_1074152\|2017-05-22 |
| >lcl\|Query_5697:5287-5616 | hRSV/A/Argentina/BA-HNRG-330/2017\|EPI_ISL_1074137\|2017-05-29 |
| >lcl\|Query_54380:5286-5614 | hRSV/A/Argentina/BA-HNRG-304/2017\|EPI_ISL_1074253\|2017-04-17 |
| >lcl\|Query_5951:5286-5614 | hRSV/A/Argentina/BA-HNRG-304/2017\|EPI_ISL_1074253\|2017-04-17 |
| >lcl\|Query_54139:5293-5621 | hRSV/A/Argentina/BA-HNRG-326/2017\|EPI_ISL_1074171\|2017-05-26 |
| >lcl\|Query_54132:5288-5616 | hRSV/A/Argentina/BA-HNRG-372/2017\|EPI_ISL_1074157\|2017-06-27 |
| >lcl\|Query_5710:5293-5621 | hRSV/A/Argentina/BA-HNRG-326/2017\|EPI_ISL_1074171\|2017-05-26 |
| >lcl\|Query_5703:5288-5616 | hRSV/A/Argentina/BA-HNRG-372/2017\|EPI_ISL_1074157\|2017-06-27 |
| >lcl\|Query_54278:5282-5611 | hRSV/A/Australia/VIC-RCH192/2018\|EPI_ISL_1834148\|2018-12-16 |
| >lcl\|Query_5849:5282-5611 | hRSV/A/Australia/VIC-RCH192/2018\|EPI_ISL_1834148\|2018-12-16 |
| >lcl\|Query_54260:5292-5621 | hRSV/A/Australia/VIC-RCH144/2017\|EPI_ISL_1834130\|2017-12-31 |
| >lcl\|Query_5831:5292-5621 | hRSV/A/Australia/VIC-RCH144/2017\|EPI_ISL_1834130\|2017-12-31 |
| >lcl\|Query_54439:5255-5584 | hRSV/A/South_Africa/NICD-R04470/2021\|EPI_ISL_12529646\|2021-03-30 |
| >lcl\|Query_54438:5273-5602 | hRSV/A/South_Africa/NICD-R04469/2021\|EPI_ISL_12529645\|2021-03-30 |
| >lcl\|Query_54433:5255-5584 | hRSV/A/South_Africa/NICD-R03866/2021\|EPI_ISL_12529640\|2021-03-15 |
| >lcl\|Query_6010:5255-5584 | hRSV/A/South_Africa/NICD-R04470/2021\|EPI_ISL_12529646\|2021-03-30 |
| >lcl\|Query_6009:5273-5602 | hRSV/A/South_Africa/NICD-R04469/2021\|EPI_ISL_12529645\|2021-03-30 |
| >lcl\|Query_6004:5255-5584 | hRSV/A/South_Africa/NICD-R03866/2021\|EPI_ISL_12529640\|2021-03-15 |
| >lcl\|Query_54432:5261-5590 | hRSV/A/South_Africa/NICD-R03864/2021\|EPI_ISL_12529639\|2021-03-15 |
| >lcl\|Query_6003:5261-5590 | hRSV/A/South_Africa/NICD-R03864/2021\|EPI_ISL_12529639\|2021-03-15 |
| >lcl\|Query_54440:5242-5571 | hRSV/A/South_Africa/NICD-R04476/2021\|EPI_ISL_12529647\|2021-03-30 |
| >lcl\|Query_6011:5242-5571 | hRSV/A/South_Africa/NICD-R04476/2021\|EPI_ISL_12529647\|2021-03-30 |
| >lcl\|Query_54437:5261-5590 | hRSV/A/South_Africa/NICD-R04354/2021\|EPI_ISL_12529644\|2021-03-31 |
| >lcl\|Query_54428:5271-5600 | hRSV/A/South_Africa/NICD-R03651/2021\|EPI_ISL_12529634\|2021-03-09 |
| >lcl\|Query_6008:5261-5590 | hRSV/A/South_Africa/NICD-R04354/2021\|EPI_ISL_12529644\|2021-03-31 |
| >lcl\|Query_5999:5271-5600 | hRSV/A/South_Africa/NICD-R03651/2021\|EPI_ISL_12529634\|2021-03-09 |
| >lcl\|Query_54436:5264-5593 | hRSV/A/South_Africa/NICD-R04181/2021\|EPI_ISL_12529643\|2021-03-25 |
| >lcl\|Query_6007:5264-5593 | hRSV/A/South_Africa/NICD-R04181/2021\|EPI_ISL_12529643\|2021-03-25 |
| >lcl\|Query_54434:5266-5595 | hRSV/A/South_Africa/NICD-R03876/2021\|EPI_ISL_12529641\|2021-03-17 |
| >lcl\|Query_6005:5266-5595 | hRSV/A/South_Africa/NICD-R03876/2021\|EPI_ISL_12529641\|2021-03-17 |
| >lcl\|Query_54435:5275-5604 | hRSV/A/South_Africa/NICD-R04004/2021\|EPI_ISL_12529642\|2021-03-19 |
| >lcl\|Query_6006:5275-5604 | hRSV/A/South_Africa/NICD-R04004/2021\|EPI_ISL_12529642\|2021-03-19 |
| >lcl\|Query_54431:5255-5584 | hRSV/A/South_Africa/NICD-R03672/2021\|EPI_ISL_12529638\|2021-03-12 |
| >lcl\|Query_6002:5255-5584 | hRSV/A/South_Africa/NICD-R03672/2021\|EPI_ISL_12529638\|2021-03-12 |
| >lcl\|Query_54429:5272-5601 | hRSV/A/South_Africa/NICD-R03652/2021\|EPI_ISL_12529635\|2021-03-09 |
| >lcl\|Query_6000:5272-5601 | hRSV/A/South_Africa/NICD-R03652/2021\|EPI_ISL_12529635\|2021-03-09 |
| >lcl\|Query_54145:5001-5330 | hRSV/A/England/194860823/2019\|EPI_ISL_11428318\|2019-11-27 |
| >lcl\|Query_5716:5001-5330 | hRSV/A/England/194860823/2019\|EPI_ISL_11428318\|2019-11-27 |
| >lcl\|Query_54525:5282-5611 | hRSV/A/Mozambique/MAP-INS014/2017\|EPI_ISL_1647462\|2017-02-14 |
| >lcl\|Query_6096:5282-5611 | hRSV/A/Mozambique/MAP-INS014/2017\|EPI_ISL_1647462\|2017-02-14 |
| >lcl\|Query_54523:5284-5613 | hRSV/A/Mozambique/MAP-INS011/2018\|EPI_ISL_1647460\|2018-05-10 |
| >lcl\|Query_54522:5281-5610 | hRSV/A/Mozambique/MAP-INS009/2018\|EPI_ISL_1647459\|2018-04-17 |
| >lcl\|Query_54520:5284-5613 | hRSV/A/Mozambique/MAP-INS006/2018\|EPI_ISL_1647457\|2018-04-03 |
| >lcl\|Query_6094:5284-5613 | hRSV/A/Mozambique/MAP-INS011/2018\|EPI_ISL_1647460\|2018-05-10 |
| >lcl\|Query_6093:5281-5610 | hRSV/A/Mozambique/MAP-INS009/2018\|EPI_ISL_1647459\|2018-04-17 |
| >lcl\|Query_6091:5284-5613 | hRSV/A/Mozambique/MAP-INS006/2018\|EPI_ISL_1647457\|2018-04-03 |
| >lcl\|Query_54236:5282-5611 | hRSV/A/Australia/VIC-RCH085/2017\|EPI_ISL_1834104\|2017-07-26 |
| >lcl\|Query_5807:5282-5611 | hRSV/A/Australia/VIC-RCH085/2017\|EPI_ISL_1834104\|2017-07-26 |
| >lcl\|Query_54377:5292-5621 | hRSV/A/Argentina/BA-HNRG-355/2017\|EPI_ISL_1074234\|2017-06-16 |
| >lcl\|Query_54137:5284-5613 | hRSV/A/Argentina/BA-HNRG-320/2017\|EPI_ISL_1074169\|2017-05-19 |
| >lcl\|Query_5948:5292-5621 | hRSV/A/Argentina/BA-HNRG-355/2017\|EPI_ISL_1074234\|2017-06-16 |
| >lcl\|Query_5708:5284-5613 | hRSV/A/Argentina/BA-HNRG-320/2017\|EPI_ISL_1074169\|2017-05-19 |
| >lcl\|Query_54452:5303-5630 | hRSV/A/Philippines/99072/2019\|EPI_ISL_12970415\|2019-10-08 |
| >lcl\|Query_6023:5303-5630 | hRSV/A/Philippines/99072/2019\|EPI_ISL_12970415\|2019-10-08 |
| >lcl\|Query_54246:5281-5610 | hRSV/A/Australia/VIC-RCH114/2018\|EPI_ISL_1834115\|2018-07-01 |
| >lcl\|Query_5817:5281-5610 | hRSV/A/Australia/VIC-RCH114/2018\|EPI_ISL_1834115\|2018-07-01 |
| >lcl\|Query_54118:5290-5620 | hRSV/A/Argentina/BA-HNRG-307/2017\|EPI_ISL_1074100\|2017-05-02 |
| >lcl\|Query_5689:5290-5620 | hRSV/A/Argentina/BA-HNRG-307/2017\|EPI_ISL_1074100\|2017-05-02 |
| >lcl\|Query_54238:5281-5611 | hRSV/A/Australia/VIC-RCH095/2018\|EPI_ISL_1834106\|2018-06-17 |
| >lcl\|Query_5809:5281-5611 | hRSV/A/Australia/VIC-RCH095/2018\|EPI_ISL_1834106\|2018-06-17 |
| >lcl\|Query_54511:5286-5615 | hRSV/A/England/RE20000029/2020\|EPI_ISL_1647415\|2020-01-10 |
| >lcl\|Query_54510:5255-5584 | hRSV/A/England/RE20000027/2020\|EPI_ISL_1647414\|2020-01-09 |
| >lcl\|Query_6082:5286-5615 | hRSV/A/England/RE20000029/2020\|EPI_ISL_1647415\|2020-01-10 |
| >lcl\|Query_6081:5255-5584 | hRSV/A/England/RE20000027/2020\|EPI_ISL_1647414\|2020-01-09 |
| >lcl\|Query_54209:5243-5572 | hRSV/A/England/RE19003299/2019\|EPI_ISL_1520435\|2019-12-18 |
| >lcl\|Query_5780:5243-5572 | hRSV/A/England/RE19003299/2019\|EPI_ISL_1520435\|2019-12-18 |
| >lcl\|Query_54241:5281-5610 | hRSV/A/Australia/VIC-RCH105/2018\|EPI_ISL_1834109\|2018-06-24 |
| >lcl\|Query_5812:5281-5610 | hRSV/A/Australia/VIC-RCH105/2018\|EPI_ISL_1834109\|2018-06-24 |
| >lcl\|Query_54492:5276-5605 | hRSV/A/England/195200145/2019\|EPI_ISL_1520407\|2019-12-20 |
| >lcl\|Query_6063:5276-5605 | hRSV/A/England/195200145/2019\|EPI_ISL_1520407\|2019-12-20 |
| >lcl\|Query_54214:5284-5613 | hRSV/A/England/RE20000103/2020\|EPI_ISL_1520440\|2020-01-24 |
| >lcl\|Query_5785:5284-5613 | hRSV/A/England/RE20000103/2020\|EPI_ISL_1520440\|2020-01-24 |
| >lcl\|Query_54494:5232-5561 | hRSV/A/England/200100151/2019\|EPI_ISL_1520409\|2019-12-23 |
| >lcl\|Query_6065:5232-5561 | hRSV/A/England/200100151/2019\|EPI_ISL_1520409\|2019-12-23 |
| >lcl\|Query_54462:5301-5630 | hRSV/A/Philippines/99084/2020\|EPI_ISL_12970426\|2020-01-22 |
| >lcl\|Query_6033:5301-5630 | hRSV/A/Philippines/99084/2020\|EPI_ISL_12970426\|2020-01-22 |
| >lcl\|Query_54427:5322-5651 | hRSV/A/Australia/VIC-RCH092/2021\|EPI_ISL_11817099\|2021-04-10 |
| >lcl\|Query_54426:5324-5653 | hRSV/A/Australia/VIC-RCH083/2021\|EPI_ISL_11817097\|2021-03-29 |
| >lcl\|Query_54425:5324-5653 | hRSV/A/Australia/VIC-RCH081/2021\|EPI_ISL_11817096\|2021-03-29 |
| >lcl\|Query_54408:5304-5633 | hRSV/A/Australia/QLD-RBWH155/2021\|EPI_ISL_11817056\|2021-02-24 |
| >lcl\|Query_54406:5304-5633 | hRSV/A/Australia/QLD-RBWH114/2021\|EPI_ISL_11817051\|2021-02-15 |
| >lcl\|Query_54405:5304-5633 | hRSV/A/Australia/QLD-RBWH089/2021\|EPI_ISL_11817049\|2021-02-12 |
| >lcl\|Query_54403:5306-5635 | hRSV/A/Australia/QLD-RBWH073/2021\|EPI_ISL_11817045\|2021-02-08 |
| >lcl\|Query_54398:5306-5635 | hRSV/A/Australia/QLD-RBWH056/2021\|EPI_ISL_11817038\|2021-02-03 |
| >lcl\|Query_54396:5304-5633 | hRSV/A/Australia/QLD-RBWH044/2021\|EPI_ISL_11817032\|2021-01-30 |
| >lcl\|Query_54395:5304-5633 | hRSV/A/Australia/QLD-RBWH041/2021\|EPI_ISL_11817030\|2021-01-29 |
| >lcl\|Query_54394:5304-5633 | hRSV/A/Australia/QLD-RBWH035/2021\|EPI_ISL_11817028\|2021-01-27 |
| >lcl\|Query_54393:5304-5633 | hRSV/A/Australia/QLD-RBWH034/2021\|EPI_ISL_11817027\|2021-01-26 |
| >lcl\|Query_54390:5304-5633 | hRSV/A/Australia/QLD-RBWH021/2021\|EPI_ISL_11817024\|2021-01-17 |
| >lcl\|Query_54355:5231-5560 | hRSV/A/Australia/WM3140467/2020\|EPI_ISL_2839279\|2020-11-09 |
| >lcl\|Query_54354:5231-5560 | hRSV/A/Australia/WM3081573/2020\|EPI_ISL_2839275\|2020-11-03 |
| >lcl\|Query_54353:5231-5560 | hRSV/A/Australia/WM3351169/2020\|EPI_ISL_2839273\|2020-11-30 |
| >lcl\|Query_54352:5231-5560 | hRSV/A/Australia/WM3341337/2020\|EPI_ISL_2839271\|2020-11-29 |
| >lcl\|Query_54351:5231-5560 | hRSV/A/Australia/WM3280473/2020\|EPI_ISL_2839269\|2020-11-23 |
| >lcl\|Query_54350:5231-5560 | hRSV/A/Australia/WM3202125/2020\|EPI_ISL_2839266\|2020-11-15 |
| >lcl\|Query_54349:5231-5560 | hRSV/A/Australia/WM3090116/2020\|EPI_ISL_2839264\|2020-11-04 |
| >lcl\|Query_54348:5231-5560 | hRSV/A/Australia/WM3312926/2020\|EPI_ISL_2839262\|2020-11-26 |
| >lcl\|Query_54346:5231-5560 | hRSV/A/Australia/WM3221617/2020\|EPI_ISL_2839258\|2020-11-17 |
| >lcl\|Query_54345:5231-5560 | hRSV/A/Australia/WM3160671/2020\|EPI_ISL_2839256\|2020-11-11 |
| >lcl\|Query_54343:5231-5560 | hRSV/A/Australia/WM3220890/2020\|EPI_ISL_2839252\|2020-11-17 |
| >lcl\|Query_54302:5278-5607 | hRSV/A/Australia/VIC-RCH061/2021\|EPI_ISL_2543810\|2021-02-18 |
| >lcl\|Query_54301:5274-5603 | hRSV/A/Australia/VIC-RCH056/2021\|EPI_ISL_2543809\|2021-03-07 |
| >lcl\|Query_54296:5273-5602 | hRSV/A/Australia/VIC-RCH047/2021\|EPI_ISL_2543804\|2021-03-04 |
| >lcl\|Query_54293:5277-5606 | hRSV/A/Australia/VIC-RCH040/2021\|EPI_ISL_2543801\|2021-02-28 |
| >lcl\|Query_54292:5277-5606 | hRSV/A/Australia/VIC-RCH035/2021\|EPI_ISL_2543800\|2021-02-28 |
| >lcl\|Query_54161:5322-5651 | hRSV/A/Australia/VIC-RCH110/2021\|EPI_ISL_11817109\|2021-05-16 |
| >lcl\|Query_54153:5317-5646 | hRSV/A/Australia/VIC-RCH095/2021\|EPI_ISL_11817101\|2021-04-10 |
| >lcl\|Query_54151:5322-5651 | hRSV/A/Australia/VIC-RCH112/2021\|EPI_ISL_11817088\|2021-03-13 |
| >lcl\|Query_54150:5304-5633 | hRSV/A/Australia/QLD-RBWH291/2021\|EPI_ISL_11817086\|2021-04-12 |
| >lcl\|Query_54147:5306-5635 | hRSV/A/Australia/QLD-RBWH282/2021\|EPI_ISL_11817080\|2021-04-04 |
| >lcl\|Query_5998:5322-5651 | hRSV/A/Australia/VIC-RCH092/2021\|EPI_ISL_11817099\|2021-04-10 |
| >lcl\|Query_5997:5324-5653 | hRSV/A/Australia/VIC-RCH083/2021\|EPI_ISL_11817097\|2021-03-29 |
| >lcl\|Query_5996:5324-5653 | hRSV/A/Australia/VIC-RCH081/2021\|EPI_ISL_11817096\|2021-03-29 |
| >lcl\|Query_5979:5304-5633 | hRSV/A/Australia/QLD-RBWH155/2021\|EPI_ISL_11817056\|2021-02-24 |
| >lcl\|Query_5977:5304-5633 | hRSV/A/Australia/QLD-RBWH114/2021\|EPI_ISL_11817051\|2021-02-15 |
| >lcl\|Query_5976:5304-5633 | hRSV/A/Australia/QLD-RBWH089/2021\|EPI_ISL_11817049\|2021-02-12 |
| >lcl\|Query_5974:5306-5635 | hRSV/A/Australia/QLD-RBWH073/2021\|EPI_ISL_11817045\|2021-02-08 |
| >lcl\|Query_5969:5306-5635 | hRSV/A/Australia/QLD-RBWH056/2021\|EPI_ISL_11817038\|2021-02-03 |
| >lcl\|Query_5967:5304-5633 | hRSV/A/Australia/QLD-RBWH044/2021\|EPI_ISL_11817032\|2021-01-30 |
| >lcl\|Query_5966:5304-5633 | hRSV/A/Australia/QLD-RBWH041/2021\|EPI_ISL_11817030\|2021-01-29 |
| >lcl\|Query_5965:5304-5633 | hRSV/A/Australia/QLD-RBWH035/2021\|EPI_ISL_11817028\|2021-01-27 |
| >lcl\|Query_5964:5304-5633 | hRSV/A/Australia/QLD-RBWH034/2021\|EPI_ISL_11817027\|2021-01-26 |
| >lcl\|Query_5961:5304-5633 | hRSV/A/Australia/QLD-RBWH021/2021\|EPI_ISL_11817024\|2021-01-17 |
| >lcl\|Query_5926:5231-5560 | hRSV/A/Australia/WM3140467/2020\|EPI_ISL_2839279\|2020-11-09 |
| >lcl\|Query_5925:5231-5560 | hRSV/A/Australia/WM3081573/2020\|EPI_ISL_2839275\|2020-11-03 |
| >lcl\|Query_5924:5231-5560 | hRSV/A/Australia/WM3351169/2020\|EPI_ISL_2839273\|2020-11-30 |
| >lcl\|Query_5923:5231-5560 | hRSV/A/Australia/WM3341337/2020\|EPI_ISL_2839271\|2020-11-29 |
| >lcl\|Query_5922:5231-5560 | hRSV/A/Australia/WM3280473/2020\|EPI_ISL_2839269\|2020-11-23 |
| >lcl\|Query_5921:5231-5560 | hRSV/A/Australia/WM3202125/2020\|EPI_ISL_2839266\|2020-11-15 |
| >lcl\|Query_5920:5231-5560 | hRSV/A/Australia/WM3090116/2020\|EPI_ISL_2839264\|2020-11-04 |
| >lcl\|Query_5919:5231-5560 | hRSV/A/Australia/WM3312926/2020\|EPI_ISL_2839262\|2020-11-26 |
| >lcl\|Query_5917:5231-5560 | hRSV/A/Australia/WM3221617/2020\|EPI_ISL_2839258\|2020-11-17 |
| >lcl\|Query_5916:5231-5560 | hRSV/A/Australia/WM3160671/2020\|EPI_ISL_2839256\|2020-11-11 |
| >lcl\|Query_5914:5231-5560 | hRSV/A/Australia/WM3220890/2020\|EPI_ISL_2839252\|2020-11-17 |
| >lcl\|Query_5873:5278-5607 | hRSV/A/Australia/VIC-RCH061/2021\|EPI_ISL_2543810\|2021-02-18 |
| >lcl\|Query_5872:5274-5603 | hRSV/A/Australia/VIC-RCH056/2021\|EPI_ISL_2543809\|2021-03-07 |
| >lcl\|Query_5867:5273-5602 | hRSV/A/Australia/VIC-RCH047/2021\|EPI_ISL_2543804\|2021-03-04 |
| >lcl\|Query_5864:5277-5606 | hRSV/A/Australia/VIC-RCH040/2021\|EPI_ISL_2543801\|2021-02-28 |
| >lcl\|Query_5863:5277-5606 | hRSV/A/Australia/VIC-RCH035/2021\|EPI_ISL_2543800\|2021-02-28 |
| >lcl\|Query_5732:5322-5651 | hRSV/A/Australia/VIC-RCH110/2021\|EPI_ISL_11817109\|2021-05-16 |
| >lcl\|Query_5724:5317-5646 | hRSV/A/Australia/VIC-RCH095/2021\|EPI_ISL_11817101\|2021-04-10 |
| >lcl\|Query_5722:5322-5651 | hRSV/A/Australia/VIC-RCH112/2021\|EPI_ISL_11817088\|2021-03-13 |
| >lcl\|Query_5721:5304-5633 | hRSV/A/Australia/QLD-RBWH291/2021\|EPI_ISL_11817086\|2021-04-12 |
| >lcl\|Query_5718:5306-5635 | hRSV/A/Australia/QLD-RBWH282/2021\|EPI_ISL_11817080\|2021-04-04 |
| >lcl\|Query_54421:5329-5658 | hRSV/A/Australia/VIC-RCH067/2021\|EPI_ISL_11817091\|2021-03-20 |
| >lcl\|Query_5992:5329-5658 | hRSV/A/Australia/VIC-RCH067/2021\|EPI_ISL_11817091\|2021-03-20 |
| >lcl\|Query_54410:5304-5633 | hRSV/A/Australia/QLD-RBWH190/2021\|EPI_ISL_11817062\|2021-03-06 |
| >lcl\|Query_5981:5304-5633 | hRSV/A/Australia/QLD-RBWH190/2021\|EPI_ISL_11817062\|2021-03-06 |
| >lcl\|Query_54387:5304-5633 | hRSV/A/Australia/QLD-RBWH009/2021\|EPI_ISL_11817020\|2021-01-09 |
| >lcl\|Query_5958:5304-5633 | hRSV/A/Australia/QLD-RBWH009/2021\|EPI_ISL_11817020\|2021-01-09 |
| >lcl\|Query_54294:5277-5606 | hRSV/A/Australia/VIC-RCH041/2021\|EPI_ISL_2543802\|2021-03-01 |
| >lcl\|Query_5865:5277-5606 | hRSV/A/Australia/VIC-RCH041/2021\|EPI_ISL_2543802\|2021-03-01 |
| >lcl\|Query_54344:5231-5560 | hRSV/A/Australia/WM3081844/2020\|EPI_ISL_2839254\|2020-11-03 |
| >lcl\|Query_54148:5304-5633 | hRSV/A/Australia/QLD-RBWH286/2021\|EPI_ISL_11817082\|2021-04-06 |
| >lcl\|Query_5915:5231-5560 | hRSV/A/Australia/WM3081844/2020\|EPI_ISL_2839254\|2020-11-03 |
| >lcl\|Query_5719:5304-5633 | hRSV/A/Australia/QLD-RBWH286/2021\|EPI_ISL_11817082\|2021-04-06 |
| >lcl\|Query_54347:5231-5560 | hRSV/A/Australia/WM3060152/2020\|EPI_ISL_2839260\|2020-11-01 |
| >lcl\|Query_5918:5231-5560 | hRSV/A/Australia/WM3060152/2020\|EPI_ISL_2839260\|2020-11-01 |
| >lcl\|Query_54152:5322-5651 | hRSV/A/Australia/VIC-RCH094/2021\|EPI_ISL_11817100\|2021-04-11 |
| >lcl\|Query_5723:5322-5651 | hRSV/A/Australia/VIC-RCH094/2021\|EPI_ISL_11817100\|2021-04-11 |
| >lcl\|Query_54412:5304-5633 | hRSV/A/Australia/QLD-RBWH215/2021\|EPI_ISL_11817066\|2021-03-11 |
| >lcl\|Query_5983:5304-5633 | hRSV/A/Australia/QLD-RBWH215/2021\|EPI_ISL_11817066\|2021-03-11 |
| >lcl\|Query_54342:5231-5560 | hRSV/A/Australia/WM3220266/2020\|EPI_ISL_2839250\|2020-11-17 |
| >lcl\|Query_5913:5231-5560 | hRSV/A/Australia/WM3220266/2020\|EPI_ISL_2839250\|2020-11-17 |
| >lcl\|Query_54159:5322-5651 | hRSV/A/Australia/VIC-RCH103/2021\|EPI_ISL_11817107\|2021-04-25 |
| >lcl\|Query_5730:5322-5651 | hRSV/A/Australia/VIC-RCH103/2021\|EPI_ISL_11817107\|2021-04-25 |
| >lcl\|Query_54158:5322-5651 | hRSV/A/Australia/VIC-RCH101/2021\|EPI_ISL_11817106\|2021-04-18 |
| >lcl\|Query_5729:5322-5651 | hRSV/A/Australia/VIC-RCH101/2021\|EPI_ISL_11817106\|2021-04-18 |
| >lcl\|Query_54424:5280-5609 | hRSV/A/Australia/VIC-RCH078/2021\|EPI_ISL_11817095\|2021-03-29 |
| >lcl\|Query_54420:5324-5653 | hRSV/A/Australia/VIC-RCH063/2021\|EPI_ISL_11817090\|2021-03-14 |
| >lcl\|Query_54300:5277-5606 | hRSV/A/Australia/VIC-RCH052/2021\|EPI_ISL_2543808\|2021-03-07 |
| >lcl\|Query_54297:5277-5606 | hRSV/A/Australia/VIC-RCH048/2021\|EPI_ISL_2543805\|2021-03-07 |
| >lcl\|Query_54295:5273-5602 | hRSV/A/Australia/VIC-RCH042/2021\|EPI_ISL_2543803\|2021-02-28 |
| >lcl\|Query_54157:5325-5654 | hRSV/A/Australia/VIC-RCH100/2021\|EPI_ISL_11817105\|2021-04-18 |
| >lcl\|Query_54156:5322-5651 | hRSV/A/Australia/VIC-RCH098/2021\|EPI_ISL_11817104\|2021-04-13 |
| >lcl\|Query_54155:5325-5654 | hRSV/A/Australia/VIC-RCH097/2021\|EPI_ISL_11817103\|2021-04-17 |
| >lcl\|Query_5995:5280-5609 | hRSV/A/Australia/VIC-RCH078/2021\|EPI_ISL_11817095\|2021-03-29 |
| >lcl\|Query_5991:5324-5653 | hRSV/A/Australia/VIC-RCH063/2021\|EPI_ISL_11817090\|2021-03-14 |
| >lcl\|Query_5871:5277-5606 | hRSV/A/Australia/VIC-RCH052/2021\|EPI_ISL_2543808\|2021-03-07 |
| >lcl\|Query_5868:5277-5606 | hRSV/A/Australia/VIC-RCH048/2021\|EPI_ISL_2543805\|2021-03-07 |
| >lcl\|Query_5866:5273-5602 | hRSV/A/Australia/VIC-RCH042/2021\|EPI_ISL_2543803\|2021-02-28 |
| >lcl\|Query_5728:5325-5654 | hRSV/A/Australia/VIC-RCH100/2021\|EPI_ISL_11817105\|2021-04-18 |
| >lcl\|Query_5727:5322-5651 | hRSV/A/Australia/VIC-RCH098/2021\|EPI_ISL_11817104\|2021-04-13 |
| >lcl\|Query_5726:5325-5654 | hRSV/A/Australia/VIC-RCH097/2021\|EPI_ISL_11817103\|2021-04-17 |
| >lcl\|Query_54423:5324-5653 | hRSV/A/Australia/VIC-RCH075/2021\|EPI_ISL_11817094\|2021-03-27 |
| >lcl\|Query_54154:5324-5653 | hRSV/A/Australia/VIC-RCH096/2021\|EPI_ISL_11817102\|2021-04-17 |
| >lcl\|Query_5994:5324-5653 | hRSV/A/Australia/VIC-RCH075/2021\|EPI_ISL_11817094\|2021-03-27 |
| >lcl\|Query_5725:5324-5653 | hRSV/A/Australia/VIC-RCH096/2021\|EPI_ISL_11817102\|2021-04-17 |
| >lcl\|Query_54299:5274-5603 | hRSV/A/Australia/VIC-RCH051/2021\|EPI_ISL_2543807\|2021-03-08 |
| >lcl\|Query_5870:5274-5603 | hRSV/A/Australia/VIC-RCH051/2021\|EPI_ISL_2543807\|2021-03-08 |
| >lcl\|Query_54160:5324-5653 | hRSV/A/Australia/VIC-RCH109/2021\|EPI_ISL_11817108\|2021-05-09 |
| >lcl\|Query_5731:5324-5653 | hRSV/A/Australia/VIC-RCH109/2021\|EPI_ISL_11817108\|2021-05-09 |
| >lcl\|Query_54164:5280-5481 | hRSV/A/Spain/CHUVI-19478508/2021\|EPI_ISL_14084091\|2021-12-23 |
| >lcl\|Query_5735:5280-5481 | hRSV/A/Spain/CHUVI-19478508/2021\|EPI_ISL_14084091\|2021-12-23 |
| >lcl\|Query_54163:5218-5419 | hRSV/A/Spain/CHUVI-19475176/2021\|EPI_ISL_14084090\|2021-12-21 |
| >lcl\|Query_5734:5218-5419 | hRSV/A/Spain/CHUVI-19475176/2021\|EPI_ISL_14084090\|2021-12-21 |
| >lcl\|Query_54162:5270-5471 | hRSV/A/Spain/CHUVI-19464401/2021\|EPI_ISL_14084089\|2021-12-13 |
| >lcl\|Query_5733:5270-5471 | hRSV/A/Spain/CHUVI-19464401/2021\|EPI_ISL_14084089\|2021-12-13 |
| >lcl\|Query_54495:5337-5540 | hRSV/A/Belgium/0000002/2022\|EPI_ISL_15421344\|2022-03-23 |
| >lcl\|Query_6066:5337-5540 | hRSV/A/Belgium/0000002/2022\|EPI_ISL_15421344\|2022-03-23 |
| >lcl\|Query_54486:5242-5571 | hRSV/A/England/195140828/2019\|EPI_ISL_1520401\|2019-12-16 |
| >lcl\|Query_54312:5275-5604 | hRSV/A/Cote_d'Ivoire/IPCI-008/2019\|EPI_ISL_2543820\|2019-08-14 |
| >lcl\|Query_54285:5239-5568 | hRSV/A/England/195020395/2019\|EPI_ISL_1834155\|2019-12-06 |
| >lcl\|Query_6057:5242-5571 | hRSV/A/England/195140828/2019\|EPI_ISL_1520401\|2019-12-16 |
| >lcl\|Query_5883:5275-5604 | hRSV/A/Cote_d'Ivoire/IPCI-008/2019\|EPI_ISL_2543820\|2019-08-14 |
| >lcl\|Query_5856:5239-5568 | hRSV/A/England/195020395/2019\|EPI_ISL_1834155\|2019-12-06 |
| >lcl\|Query_54485:5228-5557 | hRSV/A/England/195120238/2019\|EPI_ISL_1520400\|2019-12-16 |
| >lcl\|Query_6056:5228-5557 | hRSV/A/England/195120238/2019\|EPI_ISL_1520400\|2019-12-16 |
| >lcl\|Query_54373:5270-5599 | hRSV/A/South_Africa/NICD-R06229/2019\|EPI_ISL_9003918\|2019-05-31 |
| >lcl\|Query_5944:5270-5599 | hRSV/A/South_Africa/NICD-R06229/2019\|EPI_ISL_9003918\|2019-05-31 |
| >lcl\|Query_54245:5282-5611 | hRSV/A/Australia/VIC-RCH113/2019\|EPI_ISL_1834114\|2019-07-08 |
| >lcl\|Query_5816:5282-5611 | hRSV/A/Australia/VIC-RCH113/2019\|EPI_ISL_1834114\|2019-07-08 |
| >lcl\|Query_54224:5274-5603 | hRSV/A/England/195180359/2019\|EPI_ISL_1647392\|2019-12-17 |
| >lcl\|Query_5795:5274-5603 | hRSV/A/England/195180359/2019\|EPI_ISL_1647392\|2019-12-17 |
| >lcl\|Query_54208:5286-5615 | hRSV/A/England/RE19003257/2019\|EPI_ISL_1520434\|2019-12-16 |
| >lcl\|Query_5779:5286-5615 | hRSV/A/England/RE19003257/2019\|EPI_ISL_1520434\|2019-12-16 |
| >lcl\|Query_54256:5282-5611 | hRSV/A/Australia/VIC-RCH139/2019\|EPI_ISL_1834126\|2019-08-04 |
| >lcl\|Query_5827:5282-5611 | hRSV/A/Australia/VIC-RCH139/2019\|EPI_ISL_1834126\|2019-08-04 |
| >lcl\|Query_54339:5274-5603 | hRSV/A/Thailand/Ayuttaya-TNIC015/2020\|EPI_ISL_2543847\|2020-11-10 |
| >lcl\|Query_54337:5274-5603 | hRSV/A/Thailand/Ayuttaya-TNIC006/2020\|EPI_ISL_2543845\|2020-11-04 |
| >lcl\|Query_5910:5274-5603 | hRSV/A/Thailand/Ayuttaya-TNIC015/2020\|EPI_ISL_2543847\|2020-11-10 |
| >lcl\|Query_5908:5274-5603 | hRSV/A/Thailand/Ayuttaya-TNIC006/2020\|EPI_ISL_2543845\|2020-11-04 |
| >lcl\|Query_54338:5274-5603 | hRSV/A/Thailand/Ayuttaya-TNIC013/2020\|EPI_ISL_2543846\|2020-11-05 |
| >lcl\|Query_5909:5274-5603 | hRSV/A/Thailand/Ayuttaya-TNIC013/2020\|EPI_ISL_2543846\|2020-11-05 |
| >lcl\|Query_54288:5234-5563 | hRSV/A/England/RE19003298/2019\|EPI_ISL_1834170\|2019-12-18 |
| >lcl\|Query_5859:5234-5563 | hRSV/A/England/RE19003298/2019\|EPI_ISL_1834170\|2019-12-18 |
| >lcl\|Query_54282:5252-5581 | hRSV/A/England/194780460/2019\|EPI_ISL_1834152\|2019-11-19 |
| >lcl\|Query_5853:5252-5581 | hRSV/A/England/194780460/2019\|EPI_ISL_1834152\|2019-11-19 |
| >lcl\|Query_54335:5276-5605 | hRSV/A/Mongolia/NCCD029/2019\|EPI_ISL_2543843\|2019-03-18 |
| >lcl\|Query_54334:5277-5606 | hRSV/A/Mongolia/NCCD028/2019\|EPI_ISL_2543842\|2019-03-11 |
| >lcl\|Query_54330:5276-5605 | hRSV/A/Mongolia/NCCD017/2019\|EPI_ISL_2543838\|2019-02-02 |
| >lcl\|Query_54329:5273-5602 | hRSV/A/Mongolia/NCCD014/2019\|EPI_ISL_2543837\|2019-01-30 |
| >lcl\|Query_54328:5273-5602 | hRSV/A/Mongolia/NCCD011/2019\|EPI_ISL_2543836\|2019-01-28 |
| >lcl\|Query_54327:5273-5602 | hRSV/A/Mongolia/NCCD010/2019\|EPI_ISL_2543835\|2019-01-21 |
| >lcl\|Query_54326:5273-5602 | hRSV/A/Mongolia/NCCD009/2019\|EPI_ISL_2543834\|2019-01-18 |
| >lcl\|Query_54323:5273-5602 | hRSV/A/Mongolia/NCCD007/2019\|EPI_ISL_2543831\|2019-01-15 |
| >lcl\|Query_5906:5276-5605 | hRSV/A/Mongolia/NCCD029/2019\|EPI_ISL_2543843\|2019-03-18 |
| >lcl\|Query_5905:5277-5606 | hRSV/A/Mongolia/NCCD028/2019\|EPI_ISL_2543842\|2019-03-11 |
| >lcl\|Query_5901:5276-5605 | hRSV/A/Mongolia/NCCD017/2019\|EPI_ISL_2543838\|2019-02-02 |
| >lcl\|Query_5900:5273-5602 | hRSV/A/Mongolia/NCCD014/2019\|EPI_ISL_2543837\|2019-01-30 |
| >lcl\|Query_5899:5273-5602 | hRSV/A/Mongolia/NCCD011/2019\|EPI_ISL_2543836\|2019-01-28 |
| >lcl\|Query_5898:5273-5602 | hRSV/A/Mongolia/NCCD010/2019\|EPI_ISL_2543835\|2019-01-21 |
| >lcl\|Query_5897:5273-5602 | hRSV/A/Mongolia/NCCD009/2019\|EPI_ISL_2543834\|2019-01-18 |
| >lcl\|Query_5894:5273-5602 | hRSV/A/Mongolia/NCCD007/2019\|EPI_ISL_2543831\|2019-01-15 |
| >lcl\|Query_54379:5288-5617 | hRSV/A/Argentina/BA-HNRG-369/2017\|EPI_ISL_1074246\|2017-06-26 |
| >lcl\|Query_54143:5286-5615 | hRSV/A/Argentina/BA-HNRG-380/2017\|EPI_ISL_1074195\|2017-07-04 |
| >lcl\|Query_5950:5288-5617 | hRSV/A/Argentina/BA-HNRG-369/2017\|EPI_ISL_1074246\|2017-06-26 |
| >lcl\|Query_5714:5286-5615 | hRSV/A/Argentina/BA-HNRG-380/2017\|EPI_ISL_1074195\|2017-07-04 |
| >lcl\|Query_54136:5291-5620 | hRSV/A/Argentina/BA-HNRG-352/2017\|EPI_ISL_1074161\|2017-06-13 |
| >lcl\|Query_54130:5289-5618 | hRSV/A/Argentina/BA-HNRG-346/2017\|EPI_ISL_1074154\|2017-06-09 |
| >lcl\|Query_54129:5293-5622 | hRSV/A/Argentina/BA-HNRG-333/2017\|EPI_ISL_1074153\|2017-05-30 |
| >lcl\|Query_5707:5291-5620 | hRSV/A/Argentina/BA-HNRG-352/2017\|EPI_ISL_1074161\|2017-06-13 |
| >lcl\|Query_5701:5289-5618 | hRSV/A/Argentina/BA-HNRG-346/2017\|EPI_ISL_1074154\|2017-06-09 |
| >lcl\|Query_5700:5293-5622 | hRSV/A/Argentina/BA-HNRG-333/2017\|EPI_ISL_1074153\|2017-05-30 |
| >lcl\|Query_54519:5286-5615 | hRSV/A/Mozambique/MAP-INS001/2018\|EPI_ISL_1647456\|2018-02-28 |
| >lcl\|Query_6090:5286-5615 | hRSV/A/Mozambique/MAP-INS001/2018\|EPI_ISL_1647456\|2018-02-28 |
| >lcl\|Query_54521:5285-5614 | hRSV/A/Mozambique/MAP-INS008/2018\|EPI_ISL_1647458\|2018-04-06 |
| >lcl\|Query_6092:5285-5614 | hRSV/A/Mozambique/MAP-INS008/2018\|EPI_ISL_1647458\|2018-04-06 |
| >lcl\|Query_54311:5277-5606 | hRSV/A/Cote_d'Ivoire/IPCI-043/2017\|EPI_ISL_2543819\|2017-10-20 |
| >lcl\|Query_54310:5276-5605 | hRSV/A/Cote_d'Ivoire/IPCI-017/2017\|EPI_ISL_2543818\|2017-09-05 |
| >lcl\|Query_5882:5277-5606 | hRSV/A/Cote_d'Ivoire/IPCI-043/2017\|EPI_ISL_2543819\|2017-10-20 |
| >lcl\|Query_5881:5276-5605 | hRSV/A/Cote_d'Ivoire/IPCI-017/2017\|EPI_ISL_2543818\|2017-09-05 |
| >lcl\|Query_54316:5279-5608 | hRSV/A/Cote_d'Ivoire/IPCI-008/2017\|EPI_ISL_2543824\|2017-08-20 |
| >lcl\|Query_5887:5279-5608 | hRSV/A/Cote_d'Ivoire/IPCI-008/2017\|EPI_ISL_2543824\|2017-08-20 |
| >lcl\|Query_54315:5275-5604 | hRSV/A/Cote_d'Ivoire/IPCI-026/2019\|EPI_ISL_2543823\|2019-10-19 |
| >lcl\|Query_5886:5275-5604 | hRSV/A/Cote_d'Ivoire/IPCI-026/2019\|EPI_ISL_2543823\|2019-10-19 |
| >lcl\|Query_54317:5279-5608 | hRSV/A/Cote_d'Ivoire/IPCI-017/2019\|EPI_ISL_2543825\|2019-09-05 |
| >lcl\|Query_5888:5279-5608 | hRSV/A/Cote_d'Ivoire/IPCI-017/2019\|EPI_ISL_2543825\|2019-09-05 |
| >lcl\|Query_54313:5288-5617 | hRSV/A/Cote_d'Ivoire/IPCI-013/2019\|EPI_ISL_2543821\|2019-08-27 |
| >lcl\|Query_5884:5288-5617 | hRSV/A/Cote_d'Ivoire/IPCI-013/2019\|EPI_ISL_2543821\|2019-08-27 |
| >lcl\|Query_54303:5279-5608 | hRSV/A/Cote_d'Ivoire/IPCI-021/2019\|EPI_ISL_2543811\|2019-09-16 |
| >lcl\|Query_5874:5279-5608 | hRSV/A/Cote_d'Ivoire/IPCI-021/2019\|EPI_ISL_2543811\|2019-09-16 |
| >lcl\|Query_54314:5279-5608 | hRSV/A/Cote_d'Ivoire/IPCI-011/2019\|EPI_ISL_2543822\|2019-08-19 |
| >lcl\|Query_5885:5279-5608 | hRSV/A/Cote_d'Ivoire/IPCI-011/2019\|EPI_ISL_2543822\|2019-08-19 |
| >lcl\|Query_54305:5279-5608 | hRSV/A/Cote_d'Ivoire/IPCI-028/2019\|EPI_ISL_2543813\|2019-10-21 |
| >lcl\|Query_5876:5279-5608 | hRSV/A/Cote_d'Ivoire/IPCI-028/2019\|EPI_ISL_2543813\|2019-10-21 |
| >lcl\|Query_54318:5276-5605 | hRSV/A/Cote_d'Ivoire/IPCI-022/2019\|EPI_ISL_2543826\|2019-09-16 |
| >lcl\|Query_5889:5276-5605 | hRSV/A/Cote_d'Ivoire/IPCI-022/2019\|EPI_ISL_2543826\|2019-09-16 |
| >lcl\|Query_54268:5282-5611 | hRSV/A/Australia/VIC-RCH168/2018\|EPI_ISL_1834138\|2018-08-26 |
| >lcl\|Query_5839:5282-5611 | hRSV/A/Australia/VIC-RCH168/2018\|EPI_ISL_1834138\|2018-08-26 |
| >lcl\|Query_54308:5279-5608 | hRSV/A/Cote_d'Ivoire/IPCI-006/2019\|EPI_ISL_2543816\|2019-07-30 |
| >lcl\|Query_5879:5279-5608 | hRSV/A/Cote_d'Ivoire/IPCI-006/2019\|EPI_ISL_2543816\|2019-07-30 |
| >lcl\|Query_54309:5279-5608 | hRSV/A/Cote_d'Ivoire/IPCI-013/2017\|EPI_ISL_2543817\|2017-09-02 |
| >lcl\|Query_5880:5279-5608 | hRSV/A/Cote_d'Ivoire/IPCI-013/2017\|EPI_ISL_2543817\|2017-09-02 |
| >lcl\|Query_54526:5281-5610 | hRSV/A/Mozambique/MAP-INS014/2018\|EPI_ISL_1647463\|2018-05-25 |
| >lcl\|Query_6097:5281-5610 | hRSV/A/Mozambique/MAP-INS014/2018\|EPI_ISL_1647463\|2018-05-25 |
| >lcl\|Query_54304:5278-5605 | hRSV/A/Cote_d'Ivoire/IPCI-023/2019\|EPI_ISL_2543812\|2019-10-21 |
| >lcl\|Query_5875:5278-5605 | hRSV/A/Cote_d'Ivoire/IPCI-023/2019\|EPI_ISL_2543812\|2019-10-21 |
| >lcl\|Query_54240:5281-5607 | hRSV/A/Australia/VIC-RCH101/2018\|EPI_ISL_1834108\|2018-06-25 |
| >lcl\|Query_5811:5281-5607 | hRSV/A/Australia/VIC-RCH101/2018\|EPI_ISL_1834108\|2018-06-25 |
| >lcl\|Query_54269:5281-5607 | hRSV/A/Australia/VIC-RCH169/2018\|EPI_ISL_1834139\|2018-08-26 |
| >lcl\|Query_5840:5281-5607 | hRSV/A/Australia/VIC-RCH169/2018\|EPI_ISL_1834139\|2018-08-26 |
| >lcl\|Query_54444:5301-5630 | hRSV/A/Philippines/99062/2019\|EPI_ISL_12970405\|2019-08-05 |
| >lcl\|Query_6015:5301-5630 | hRSV/A/Philippines/99062/2019\|EPI_ISL_12970405\|2019-08-05 |
| >lcl\|Query_54476:5257-5586 | hRSV/A/Argentina/BA-HNRG-439/2019\|EPI_ISL_15067685\|2019-07-25 |
| >lcl\|Query_54175:5257-5586 | hRSV/A/Argentina/HNRG-439/2019\|EPI_ISL_15055320\|2019-07-25 |
| >lcl\|Query_6047:5257-5586 | hRSV/A/Argentina/BA-HNRG-439/2019\|EPI_ISL_15067685\|2019-07-25 |
| >lcl\|Query_5746:5257-5586 | hRSV/A/Argentina/HNRG-439/2019\|EPI_ISL_15055320\|2019-07-25 |
| >lcl\|Query_54524:5282-5611 | hRSV/A/Mozambique/MAP-INS012/2018\|EPI_ISL_1647461\|2018-05-11 |
| >lcl\|Query_6095:5282-5611 | hRSV/A/Mozambique/MAP-INS012/2018\|EPI_ISL_1647461\|2018-05-11 |
| >lcl\|Query_54289:5253-5582 | hRSV/A/England/RE20000013/2019\|EPI_ISL_1834171\|2019-12-31 |
| >lcl\|Query_5860:5253-5582 | hRSV/A/England/RE20000013/2019\|EPI_ISL_1834171\|2019-12-31 |
| >lcl\|Query_54275:5299-5628 | hRSV/A/Australia/VIC-RCH182/2019\|EPI_ISL_1834145\|2019-09-02 |
| >lcl\|Query_54273:5281-5610 | hRSV/A/Australia/VIC-RCH181/2019\|EPI_ISL_1834143\|2019-09-02 |
| >lcl\|Query_5846:5299-5628 | hRSV/A/Australia/VIC-RCH182/2019\|EPI_ISL_1834145\|2019-09-02 |
| >lcl\|Query_5844:5281-5610 | hRSV/A/Australia/VIC-RCH181/2019\|EPI_ISL_1834143\|2019-09-02 |
| >lcl\|Query_54474:5258-5587 | hRSV/A/Argentina/BA-HNRG-436/2019\|EPI_ISL_15067683\|2019-06-21 |
| >lcl\|Query_54473:5258-5587 | hRSV/A/Argentina/BA-HNRG-435/2019\|EPI_ISL_15067682\|2019-06-11 |
| >lcl\|Query_54173:5258-5587 | hRSV/A/Argentina/HNRG-436/2019\|EPI_ISL_15055318\|2019-06-21 |
| >lcl\|Query_54172:5258-5587 | hRSV/A/Argentina/HNRG-435/2019\|EPI_ISL_15055317\|2019-06-11 |
| >lcl\|Query_6045:5258-5587 | hRSV/A/Argentina/BA-HNRG-436/2019\|EPI_ISL_15067683\|2019-06-21 |
| >lcl\|Query_6044:5258-5587 | hRSV/A/Argentina/BA-HNRG-435/2019\|EPI_ISL_15067682\|2019-06-11 |
| >lcl\|Query_5744:5258-5587 | hRSV/A/Argentina/HNRG-436/2019\|EPI_ISL_15055318\|2019-06-21 |
| >lcl\|Query_5743:5258-5587 | hRSV/A/Argentina/HNRG-435/2019\|EPI_ISL_15055317\|2019-06-11 |
| >lcl\|Query_54487:5251-5580 | hRSV/A/England/195160214/2019\|EPI_ISL_1520402\|2019-12-16 |
| >lcl\|Query_6058:5251-5580 | hRSV/A/England/195160214/2019\|EPI_ISL_1520402\|2019-12-16 |
| >lcl\|Query_54446:5301-5630 | hRSV/A/Philippines/99065/2019\|EPI_ISL_12970408\|2019-08-25 |
| >lcl\|Query_54443:5301-5630 | hRSV/A/Philippines/99060/2019\|EPI_ISL_12970403\|2019-06-25 |
| >lcl\|Query_6017:5301-5630 | hRSV/A/Philippines/99065/2019\|EPI_ISL_12970408\|2019-08-25 |
| >lcl\|Query_6014:5301-5630 | hRSV/A/Philippines/99060/2019\|EPI_ISL_12970403\|2019-06-25 |
| >lcl\|Query_54516:5275-5604 | hRSV/A/England/RS20000580/2020\|EPI_ISL_1647420\|2020-02-05 |
| >lcl\|Query_6087:5275-5604 | hRSV/A/England/RS20000580/2020\|EPI_ISL_1647420\|2020-02-05 |
| >lcl\|Query_54486:5372-5442 | hRSV/A/England/195140828/2019\|EPI_ISL_1520401\|2019-12-16 |
| >lcl\|Query_54373:5400-5470 | hRSV/A/South_Africa/NICD-R06229/2019\|EPI_ISL_9003918\|2019-05-31 |
| >lcl\|Query_54312:5405-5475 | hRSV/A/Cote_d'Ivoire/IPCI-008/2019\|EPI_ISL_2543820\|2019-08-14 |
| >lcl\|Query_54285:5369-5439 | hRSV/A/England/195020395/2019\|EPI_ISL_1834155\|2019-12-06 |
| >lcl\|Query_54485:5358-5428 | hRSV/A/England/195120238/2019\|EPI_ISL_1520400\|2019-12-16 |
| >lcl\|Query_54340:5364-5434 | hRSV/A/Russia/Novosibirsk-138Hp/2019\|EPI_ISL_2582836\|2019-12-06 |
| >lcl\|Query_54223:5385-5455 | hRSV/A/England/195080598/2019\|EPI_ISL_1647391\|2019-12-09 |
| >lcl\|Query_54208:5416-5486 | hRSV/A/England/RE19003257/2019\|EPI_ISL_1520434\|2019-12-16 |
| >lcl\|Query_54507:5415-5485 | hRSV/A/England/RE20000021/2020\|EPI_ISL_1647411\|2020-01-05 |
| >lcl\|Query_54288:5364-5434 | hRSV/A/England/RE19003298/2019\|EPI_ISL_1834170\|2019-12-18 |
| >lcl\|Query_6057:5372-5442 | hRSV/A/England/195140828/2019\|EPI_ISL_1520401\|2019-12-16 |
| >lcl\|Query_5944:5400-5470 | hRSV/A/South_Africa/NICD-R06229/2019\|EPI_ISL_9003918\|2019-05-31 |
| >lcl\|Query_5883:5405-5475 | hRSV/A/Cote_d'Ivoire/IPCI-008/2019\|EPI_ISL_2543820\|2019-08-14 |
| >lcl\|Query_5856:5369-5439 | hRSV/A/England/195020395/2019\|EPI_ISL_1834155\|2019-12-06 |
| >lcl\|Query_6056:5358-5428 | hRSV/A/England/195120238/2019\|EPI_ISL_1520400\|2019-12-16 |
| >lcl\|Query_5911:5364-5434 | hRSV/A/Russia/Novosibirsk-138Hp/2019\|EPI_ISL_2582836\|2019-12-06 |
| >lcl\|Query_5794:5385-5455 | hRSV/A/England/195080598/2019\|EPI_ISL_1647391\|2019-12-09 |
| >lcl\|Query_5779:5416-5486 | hRSV/A/England/RE19003257/2019\|EPI_ISL_1520434\|2019-12-16 |
| >lcl\|Query_6078:5415-5485 | hRSV/A/England/RE20000021/2020\|EPI_ISL_1647411\|2020-01-05 |
| >lcl\|Query_5859:5364-5434 | hRSV/A/England/RE19003298/2019\|EPI_ISL_1834170\|2019-12-18 |
| >lcl\|Query_54286:5388-5458 | hRSV/A/England/195040643/2019\|EPI_ISL_1834158\|2019-12-09 |
| >lcl\|Query_54217:5397-5467 | hRSV/A/England/194540440/2019\|EPI_ISL_1647385\|2019-11-04 |
| >lcl\|Query_5857:5388-5458 | hRSV/A/England/195040643/2019\|EPI_ISL_1834158\|2019-12-09 |
| >lcl\|Query_5788:5397-5467 | hRSV/A/England/194540440/2019\|EPI_ISL_1647385\|2019-11-04 |
| >lcl\|Query_54245:5412-5482 | hRSV/A/Australia/VIC-RCH113/2019\|EPI_ISL_1834114\|2019-07-08 |
| >lcl\|Query_54224:5404-5474 | hRSV/A/England/195180359/2019\|EPI_ISL_1647392\|2019-12-17 |
| >lcl\|Query_5816:5412-5482 | hRSV/A/Australia/VIC-RCH113/2019\|EPI_ISL_1834114\|2019-07-08 |
| >lcl\|Query_5795:5404-5474 | hRSV/A/England/195180359/2019\|EPI_ISL_1647392\|2019-12-17 |
| >lcl\|Query_54517:5415-5485 | hRSV/A/England/RS20000581/2020\|EPI_ISL_1647421\|2020-02-04 |
| >lcl\|Query_6088:5415-5485 | hRSV/A/England/RS20000581/2020\|EPI_ISL_1647421\|2020-02-04 |
| >lcl\|Query_54279:5415-5485 | hRSV/A/Australia/VIC-RCH202/2019\|EPI_ISL_1834149\|2019-12-09 |
| >lcl\|Query_5850:5415-5485 | hRSV/A/Australia/VIC-RCH202/2019\|EPI_ISL_1834149\|2019-12-09 |
| >lcl\|Query_54266:5411-5481 | hRSV/A/Australia/VIC-RCH160/2019\|EPI_ISL_1834136\|2019-08-18 |
| >lcl\|Query_5837:5411-5481 | hRSV/A/Australia/VIC-RCH160/2019\|EPI_ISL_1834136\|2019-08-18 |
| >lcl\|Query_54212:5395-5465 | hRSV/A/England/RE20000093/2020\|EPI_ISL_1520438\|2020-01-28 |
| >lcl\|Query_5783:5395-5465 | hRSV/A/England/RE20000093/2020\|EPI_ISL_1520438\|2020-01-28 |
| >lcl\|Query_54373:5472-5542 | hRSV/A/South_Africa/NICD-R06229/2019\|EPI_ISL_9003918\|2019-05-31 |
| >lcl\|Query_54282:5382-5452 | hRSV/A/England/194780460/2019\|EPI_ISL_1834152\|2019-11-19 |
| >lcl\|Query_54218:5408-5478 | hRSV/A/England/194600343/2019\|EPI_ISL_1647386\|2019-11-08 |
| >lcl\|Query_54335:5406-5476 | hRSV/A/Mongolia/NCCD029/2019\|EPI_ISL_2543843\|2019-03-18 |
| >lcl\|Query_54334:5407-5477 | hRSV/A/Mongolia/NCCD028/2019\|EPI_ISL_2543842\|2019-03-11 |
| >lcl\|Query_54330:5406-5476 | hRSV/A/Mongolia/NCCD017/2019\|EPI_ISL_2543838\|2019-02-02 |
| >lcl\|Query_54329:5403-5473 | hRSV/A/Mongolia/NCCD014/2019\|EPI_ISL_2543837\|2019-01-30 |
| >lcl\|Query_54328:5403-5473 | hRSV/A/Mongolia/NCCD011/2019\|EPI_ISL_2543836\|2019-01-28 |
| >lcl\|Query_54327:5403-5473 | hRSV/A/Mongolia/NCCD010/2019\|EPI_ISL_2543835\|2019-01-21 |
| >lcl\|Query_54326:5403-5473 | hRSV/A/Mongolia/NCCD009/2019\|EPI_ISL_2543834\|2019-01-18 |
| >lcl\|Query_54323:5403-5473 | hRSV/A/Mongolia/NCCD007/2019\|EPI_ISL_2543831\|2019-01-15 |
| >lcl\|Query_54240:5411-5481 | hRSV/A/Australia/VIC-RCH101/2018\|EPI_ISL_1834108\|2018-06-25 |
| >lcl\|Query_5944:5472-5542 | hRSV/A/South_Africa/NICD-R06229/2019\|EPI_ISL_9003918\|2019-05-31 |
| >lcl\|Query_5853:5382-5452 | hRSV/A/England/194780460/2019\|EPI_ISL_1834152\|2019-11-19 |
| >lcl\|Query_5789:5408-5478 | hRSV/A/England/194600343/2019\|EPI_ISL_1647386\|2019-11-08 |
| >lcl\|Query_5906:5406-5476 | hRSV/A/Mongolia/NCCD029/2019\|EPI_ISL_2543843\|2019-03-18 |
| >lcl\|Query_5905:5407-5477 | hRSV/A/Mongolia/NCCD028/2019\|EPI_ISL_2543842\|2019-03-11 |
| >lcl\|Query_5901:5406-5476 | hRSV/A/Mongolia/NCCD017/2019\|EPI_ISL_2543838\|2019-02-02 |
| >lcl\|Query_5900:5403-5473 | hRSV/A/Mongolia/NCCD014/2019\|EPI_ISL_2543837\|2019-01-30 |
| >lcl\|Query_5899:5403-5473 | hRSV/A/Mongolia/NCCD011/2019\|EPI_ISL_2543836\|2019-01-28 |
| >lcl\|Query_5898:5403-5473 | hRSV/A/Mongolia/NCCD010/2019\|EPI_ISL_2543835\|2019-01-21 |
| >lcl\|Query_5897:5403-5473 | hRSV/A/Mongolia/NCCD009/2019\|EPI_ISL_2543834\|2019-01-18 |
| >lcl\|Query_5894:5403-5473 | hRSV/A/Mongolia/NCCD007/2019\|EPI_ISL_2543831\|2019-01-15 |
| >lcl\|Query_5811:5411-5481 | hRSV/A/Australia/VIC-RCH101/2018\|EPI_ISL_1834108\|2018-06-25 |
| >lcl\|Query_54452:5431-5501 | hRSV/A/Philippines/99072/2019\|EPI_ISL_12970415\|2019-10-08 |
| >lcl\|Query_6023:5431-5501 | hRSV/A/Philippines/99072/2019\|EPI_ISL_12970415\|2019-10-08 |
| >lcl\|Query_54269:5411-5481 | hRSV/A/Australia/VIC-RCH169/2018\|EPI_ISL_1834139\|2018-08-26 |
| >lcl\|Query_5840:5411-5481 | hRSV/A/Australia/VIC-RCH169/2018\|EPI_ISL_1834139\|2018-08-26 |
| >lcl\|Query_54209:5373-5443 | hRSV/A/England/RE19003299/2019\|EPI_ISL_1520435\|2019-12-18 |
| >lcl\|Query_54289:5383-5453 | hRSV/A/England/RE20000013/2019\|EPI_ISL_1834171\|2019-12-31 |
| >lcl\|Query_5780:5373-5443 | hRSV/A/England/RE19003299/2019\|EPI_ISL_1520435\|2019-12-18 |
| >lcl\|Query_5860:5383-5453 | hRSV/A/England/RE20000013/2019\|EPI_ISL_1834171\|2019-12-31 |
| >lcl\|Query_54275:5429-5499 | hRSV/A/Australia/VIC-RCH182/2019\|EPI_ISL_1834145\|2019-09-02 |
| >lcl\|Query_54273:5411-5481 | hRSV/A/Australia/VIC-RCH181/2019\|EPI_ISL_1834143\|2019-09-02 |
| >lcl\|Query_5846:5429-5499 | hRSV/A/Australia/VIC-RCH182/2019\|EPI_ISL_1834145\|2019-09-02 |
| >lcl\|Query_5844:5411-5481 | hRSV/A/Australia/VIC-RCH181/2019\|EPI_ISL_1834143\|2019-09-02 |
| >lcl\|Query_54478:5389-5459 | hRSV/A/Argentina/BA-HNRG-441/2019\|EPI_ISL_15067687\|2019-07-29 |
| >lcl\|Query_54177:5389-5459 | hRSV/A/Argentina/HNRG-441/2019\|EPI_ISL_15055322\|2019-07-29 |
| >lcl\|Query_6049:5389-5459 | hRSV/A/Argentina/BA-HNRG-441/2019\|EPI_ISL_15067687\|2019-07-29 |
| >lcl\|Query_5748:5389-5459 | hRSV/A/Argentina/HNRG-441/2019\|EPI_ISL_15055322\|2019-07-29 |
| >lcl\|Query_54446:5431-5501 | hRSV/A/Philippines/99065/2019\|EPI_ISL_12970408\|2019-08-25 |
| >lcl\|Query_54443:5431-5501 | hRSV/A/Philippines/99060/2019\|EPI_ISL_12970403\|2019-06-25 |
| >lcl\|Query_6017:5431-5501 | hRSV/A/Philippines/99065/2019\|EPI_ISL_12970408\|2019-08-25 |
| >lcl\|Query_6014:5431-5501 | hRSV/A/Philippines/99060/2019\|EPI_ISL_12970403\|2019-06-25 |
| >lcl\|Query_54384:5424-5494 | hRSV/A/Argentina/BA-HNRG-314/2017\|EPI_ISL_1074259\|2017-05-12 |
| >lcl\|Query_54141:5422-5492 | hRSV/A/Argentina/BA-HNRG-388/2017\|EPI_ISL_1074173\|2017-07-17 |
| >lcl\|Query_54133:5418-5488 | hRSV/A/Argentina/BA-HNRG-315/2017\|EPI_ISL_1074158\|2017-05-15 |
| >lcl\|Query_54311:5407-5477 | hRSV/A/Cote_d'Ivoire/IPCI-043/2017\|EPI_ISL_2543819\|2017-10-20 |
| >lcl\|Query_54310:5406-5476 | hRSV/A/Cote_d'Ivoire/IPCI-017/2017\|EPI_ISL_2543818\|2017-09-05 |
| >lcl\|Query_54380:5415-5485 | hRSV/A/Argentina/BA-HNRG-304/2017\|EPI_ISL_1074253\|2017-04-17 |
| >lcl\|Query_54316:5409-5479 | hRSV/A/Cote_d'Ivoire/IPCI-008/2017\|EPI_ISL_2543824\|2017-08-20 |
| >lcl\|Query_54306:5409-5479 | hRSV/A/Cote_d'Ivoire/IPCI-009/2017\|EPI_ISL_2543814\|2017-08-25 |
| >lcl\|Query_54136:5421-5491 | hRSV/A/Argentina/BA-HNRG-352/2017\|EPI_ISL_1074161\|2017-06-13 |
| >lcl\|Query_54130:5419-5489 | hRSV/A/Argentina/BA-HNRG-346/2017\|EPI_ISL_1074154\|2017-06-09 |
| >lcl\|Query_54129:5423-5493 | hRSV/A/Argentina/BA-HNRG-333/2017\|EPI_ISL_1074153\|2017-05-30 |
| >lcl\|Query_54519:5416-5486 | hRSV/A/Mozambique/MAP-INS001/2018\|EPI_ISL_1647456\|2018-02-28 |
| >lcl\|Query_54278:5412-5482 | hRSV/A/Australia/VIC-RCH192/2018\|EPI_ISL_1834148\|2018-12-16 |
| >lcl\|Query_54260:5422-5492 | hRSV/A/Australia/VIC-RCH144/2017\|EPI_ISL_1834130\|2017-12-31 |
| >lcl\|Query_54521:5415-5485 | hRSV/A/Mozambique/MAP-INS008/2018\|EPI_ISL_1647458\|2018-04-06 |
| >lcl\|Query_5955:5424-5494 | hRSV/A/Argentina/BA-HNRG-314/2017\|EPI_ISL_1074259\|2017-05-12 |
| >lcl\|Query_5712:5422-5492 | hRSV/A/Argentina/BA-HNRG-388/2017\|EPI_ISL_1074173\|2017-07-17 |
| >lcl\|Query_5704:5418-5488 | hRSV/A/Argentina/BA-HNRG-315/2017\|EPI_ISL_1074158\|2017-05-15 |
| >lcl\|Query_5882:5407-5477 | hRSV/A/Cote_d'Ivoire/IPCI-043/2017\|EPI_ISL_2543819\|2017-10-20 |
| >lcl\|Query_5881:5406-5476 | hRSV/A/Cote_d'Ivoire/IPCI-017/2017\|EPI_ISL_2543818\|2017-09-05 |
| >lcl\|Query_5951:5415-5485 | hRSV/A/Argentina/BA-HNRG-304/2017\|EPI_ISL_1074253\|2017-04-17 |
| >lcl\|Query_5887:5409-5479 | hRSV/A/Cote_d'Ivoire/IPCI-008/2017\|EPI_ISL_2543824\|2017-08-20 |
| >lcl\|Query_5877:5409-5479 | hRSV/A/Cote_d'Ivoire/IPCI-009/2017\|EPI_ISL_2543814\|2017-08-25 |
| >lcl\|Query_5707:5421-5491 | hRSV/A/Argentina/BA-HNRG-352/2017\|EPI_ISL_1074161\|2017-06-13 |
| >lcl\|Query_5701:5419-5489 | hRSV/A/Argentina/BA-HNRG-346/2017\|EPI_ISL_1074154\|2017-06-09 |
| >lcl\|Query_5700:5423-5493 | hRSV/A/Argentina/BA-HNRG-333/2017\|EPI_ISL_1074153\|2017-05-30 |
| >lcl\|Query_6090:5416-5486 | hRSV/A/Mozambique/MAP-INS001/2018\|EPI_ISL_1647456\|2018-02-28 |
| >lcl\|Query_5849:5412-5482 | hRSV/A/Australia/VIC-RCH192/2018\|EPI_ISL_1834148\|2018-12-16 |
| >lcl\|Query_5831:5422-5492 | hRSV/A/Australia/VIC-RCH144/2017\|EPI_ISL_1834130\|2017-12-31 |
| >lcl\|Query_6092:5415-5485 | hRSV/A/Mozambique/MAP-INS008/2018\|EPI_ISL_1647458\|2018-04-06 |
| >lcl\|Query_54381:5420-5490 | hRSV/A/Argentina/BA-HNRG-350/2017\|EPI_ISL_1074255\|2017-06-12 |
| >lcl\|Query_54135:5415-5485 | hRSV/A/Argentina/BA-HNRG-336/2017\|EPI_ISL_1074160\|2017-06-01 |
| >lcl\|Query_54131:5416-5486 | hRSV/A/Argentina/BA-HNRG-343/2017\|EPI_ISL_1074156\|2017-06-07 |
| >lcl\|Query_54120:5415-5485 | hRSV/A/Argentina/BA-HNRG-359/2017\|EPI_ISL_1074106\|2017-06-19 |
| >lcl\|Query_54383:5416-5486 | hRSV/A/Argentina/BA-HNRG-370/2017\|EPI_ISL_1074257\|2017-06-26 |
| >lcl\|Query_5952:5420-5490 | hRSV/A/Argentina/BA-HNRG-350/2017\|EPI_ISL_1074255\|2017-06-12 |
| >lcl\|Query_5706:5415-5485 | hRSV/A/Argentina/BA-HNRG-336/2017\|EPI_ISL_1074160\|2017-06-01 |
| >lcl\|Query_5702:5416-5486 | hRSV/A/Argentina/BA-HNRG-343/2017\|EPI_ISL_1074156\|2017-06-07 |
| >lcl\|Query_5691:5415-5485 | hRSV/A/Argentina/BA-HNRG-359/2017\|EPI_ISL_1074106\|2017-06-19 |
| >lcl\|Query_5954:5416-5486 | hRSV/A/Argentina/BA-HNRG-370/2017\|EPI_ISL_1074257\|2017-06-26 |
| >lcl\|Query_54139:5422-5492 | hRSV/A/Argentina/BA-HNRG-326/2017\|EPI_ISL_1074171\|2017-05-26 |
| >lcl\|Query_54132:5417-5487 | hRSV/A/Argentina/BA-HNRG-372/2017\|EPI_ISL_1074157\|2017-06-27 |
| >lcl\|Query_54309:5409-5479 | hRSV/A/Cote_d'Ivoire/IPCI-013/2017\|EPI_ISL_2543817\|2017-09-02 |
| >lcl\|Query_5710:5422-5492 | hRSV/A/Argentina/BA-HNRG-326/2017\|EPI_ISL_1074171\|2017-05-26 |
| >lcl\|Query_5703:5417-5487 | hRSV/A/Argentina/BA-HNRG-372/2017\|EPI_ISL_1074157\|2017-06-27 |
| >lcl\|Query_5880:5409-5479 | hRSV/A/Cote_d'Ivoire/IPCI-013/2017\|EPI_ISL_2543817\|2017-09-02 |
| >lcl\|Query_54315:5405-5475 | hRSV/A/Cote_d'Ivoire/IPCI-026/2019\|EPI_ISL_2543823\|2019-10-19 |
| >lcl\|Query_54308:5409-5479 | hRSV/A/Cote_d'Ivoire/IPCI-006/2019\|EPI_ISL_2543816\|2019-07-30 |
| >lcl\|Query_54268:5412-5482 | hRSV/A/Australia/VIC-RCH168/2018\|EPI_ISL_1834138\|2018-08-26 |
| >lcl\|Query_54304:5406-5476 | hRSV/A/Cote_d'Ivoire/IPCI-023/2019\|EPI_ISL_2543812\|2019-10-21 |
| >lcl\|Query_54526:5411-5481 | hRSV/A/Mozambique/MAP-INS014/2018\|EPI_ISL_1647463\|2018-05-25 |
| >lcl\|Query_54317:5409-5479 | hRSV/A/Cote_d'Ivoire/IPCI-017/2019\|EPI_ISL_2543825\|2019-09-05 |
| >lcl\|Query_54314:5409-5479 | hRSV/A/Cote_d'Ivoire/IPCI-011/2019\|EPI_ISL_2543822\|2019-08-19 |
| >lcl\|Query_54303:5409-5479 | hRSV/A/Cote_d'Ivoire/IPCI-021/2019\|EPI_ISL_2543811\|2019-09-16 |
| >lcl\|Query_54305:5409-5479 | hRSV/A/Cote_d'Ivoire/IPCI-028/2019\|EPI_ISL_2543813\|2019-10-21 |
| >lcl\|Query_54318:5406-5476 | hRSV/A/Cote_d'Ivoire/IPCI-022/2019\|EPI_ISL_2543826\|2019-09-16 |
| >lcl\|Query_54243:5411-5481 | hRSV/A/Australia/VIC-RCH112/2018\|EPI_ISL_1834112\|2018-06-30 |
| >lcl\|Query_54127:5414-5484 | hRSV/A/Argentina/BA-HNRG-377/2017\|EPI_ISL_1074146\|2017-06-30 |
| >lcl\|Query_54124:5424-5494 | hRSV/A/Argentina/BA-HNRG-357/2017\|EPI_ISL_1074126\|2017-06-16 |
| >lcl\|Query_54375:5419-5489 | hRSV/A/Argentina/BA-HNRG-366/2017\|EPI_ISL_1074213\|2017-06-23 |
| >lcl\|Query_5886:5405-5475 | hRSV/A/Cote_d'Ivoire/IPCI-026/2019\|EPI_ISL_2543823\|2019-10-19 |
| >lcl\|Query_5879:5409-5479 | hRSV/A/Cote_d'Ivoire/IPCI-006/2019\|EPI_ISL_2543816\|2019-07-30 |
| >lcl\|Query_5839:5412-5482 | hRSV/A/Australia/VIC-RCH168/2018\|EPI_ISL_1834138\|2018-08-26 |
| >lcl\|Query_5875:5406-5476 | hRSV/A/Cote_d'Ivoire/IPCI-023/2019\|EPI_ISL_2543812\|2019-10-21 |
| >lcl\|Query_6097:5411-5481 | hRSV/A/Mozambique/MAP-INS014/2018\|EPI_ISL_1647463\|2018-05-25 |
| >lcl\|Query_5888:5409-5479 | hRSV/A/Cote_d'Ivoire/IPCI-017/2019\|EPI_ISL_2543825\|2019-09-05 |
| >lcl\|Query_5885:5409-5479 | hRSV/A/Cote_d'Ivoire/IPCI-011/2019\|EPI_ISL_2543822\|2019-08-19 |
| >lcl\|Query_5874:5409-5479 | hRSV/A/Cote_d'Ivoire/IPCI-021/2019\|EPI_ISL_2543811\|2019-09-16 |
| >lcl\|Query_5876:5409-5479 | hRSV/A/Cote_d'Ivoire/IPCI-028/2019\|EPI_ISL_2543813\|2019-10-21 |
| >lcl\|Query_5889:5406-5476 | hRSV/A/Cote_d'Ivoire/IPCI-022/2019\|EPI_ISL_2543826\|2019-09-16 |
| >lcl\|Query_5814:5411-5481 | hRSV/A/Australia/VIC-RCH112/2018\|EPI_ISL_1834112\|2018-06-30 |
| >lcl\|Query_5698:5414-5484 | hRSV/A/Argentina/BA-HNRG-377/2017\|EPI_ISL_1074146\|2017-06-30 |
| >lcl\|Query_5695:5424-5494 | hRSV/A/Argentina/BA-HNRG-357/2017\|EPI_ISL_1074126\|2017-06-16 |
| >lcl\|Query_5946:5419-5489 | hRSV/A/Argentina/BA-HNRG-366/2017\|EPI_ISL_1074213\|2017-06-23 |
| >lcl\|Query_54290:5409-5479 | hRSV/A/England/RE20000024/2020\|EPI_ISL_1834172\|2020-01-03 |
| >lcl\|Query_54287:5382-5452 | hRSV/A/England/195140709/2019\|EPI_ISL_1834159\|2019-12-12 |
| >lcl\|Query_5861:5409-5479 | hRSV/A/England/RE20000024/2020\|EPI_ISL_1834172\|2020-01-03 |
| >lcl\|Query_5858:5382-5452 | hRSV/A/England/195140709/2019\|EPI_ISL_1834159\|2019-12-12 |
| >lcl\|Query_54142:5415-5485 | hRSV/A/Argentina/BA-HNRG-342/2017\|EPI_ISL_1074188\|2017-06-06 |
| >lcl\|Query_5713:5415-5485 | hRSV/A/Argentina/BA-HNRG-342/2017\|EPI_ISL_1074188\|2017-06-06 |
| >lcl\|Query_54444:5431-5501 | hRSV/A/Philippines/99062/2019\|EPI_ISL_12970405\|2019-08-05 |
| >lcl\|Query_6015:5431-5501 | hRSV/A/Philippines/99062/2019\|EPI_ISL_12970405\|2019-08-05 |
| >lcl\|Query_54277:5411-5481 | hRSV/A/Australia/VIC-RCH188/2018\|EPI_ISL_1834147\|2018-11-24 |
| >lcl\|Query_54274:5410-5480 | hRSV/A/Australia/VIC-RCH182/2018\|EPI_ISL_1834144\|2018-10-14 |
| >lcl\|Query_5848:5411-5481 | hRSV/A/Australia/VIC-RCH188/2018\|EPI_ISL_1834147\|2018-11-24 |
| >lcl\|Query_5845:5410-5480 | hRSV/A/Australia/VIC-RCH182/2018\|EPI_ISL_1834144\|2018-10-14 |
| >lcl\|Query_54515:5393-5463 | hRSV/A/England/RS20000579/2020\|EPI_ISL_1647419\|2020-02-16 |
| >lcl\|Query_54475:5389-5459 | hRSV/A/Argentina/BA-HNRG-437/2019\|EPI_ISL_15067684\|2019-06-19 |
| >lcl\|Query_54174:5389-5459 | hRSV/A/Argentina/HNRG-437/2019\|EPI_ISL_15055319\|2019-06-19 |
| >lcl\|Query_54235:5412-5482 | hRSV/A/Australia/VIC-RCH083/2017\|EPI_ISL_1834103\|2017-07-26 |
| >lcl\|Query_54430:5402-5472 | hRSV/A/South_Africa/NICD-R03671/2021\|EPI_ISL_12529637\|2021-03-12 |
| >lcl\|Query_54283:5369-5439 | hRSV/A/England/194980352/2019\|EPI_ISL_1834153\|2019-12-05 |
| >lcl\|Query_54184:5388-5458 | hRSV/A/England/200100153/2019\|EPI_ISL_1520410\|2019-12-24 |
| >lcl\|Query_54514:5412-5482 | hRSV/A/England/RE20000102/2020\|EPI_ISL_1647418\|2020-02-08 |
| >lcl\|Query_54506:5414-5484 | hRSV/A/England/RE20000020/2020\|EPI_ISL_1647410\|2020-01-06 |
| >lcl\|Query_54467:5389-5459 | hRSV/A/Argentina/BA-HNRG-413/2018\|EPI_ISL_15067676\|2018-06-18 |
| >lcl\|Query_54458:5431-5501 | hRSV/A/Philippines/99078/2019\|EPI_ISL_12970421\|2019-11-28 |
| >lcl\|Query_54448:5431-5501 | hRSV/A/Philippines/99068/2019\|EPI_ISL_12970411\|2019-09-14 |
| >lcl\|Query_54447:5431-5501 | hRSV/A/Philippines/99066/2019\|EPI_ISL_12970409\|2019-08-28 |
| >lcl\|Query_54445:5431-5501 | hRSV/A/Philippines/99064/2019\|EPI_ISL_12970407\|2019-08-19 |
| >lcl\|Query_54166:5389-5459 | hRSV/A/Argentina/HNRG-413/2018\|EPI_ISL_15055311\|2018-06-18 |
| >lcl\|Query_54454:5431-5501 | hRSV/A/Philippines/99074/2019\|EPI_ISL_12970417\|2019-10-19 |
| >lcl\|Query_6086:5393-5463 | hRSV/A/England/RS20000579/2020\|EPI_ISL_1647419\|2020-02-16 |
| >lcl\|Query_6046:5389-5459 | hRSV/A/Argentina/BA-HNRG-437/2019\|EPI_ISL_15067684\|2019-06-19 |
| >lcl\|Query_5745:5389-5459 | hRSV/A/Argentina/HNRG-437/2019\|EPI_ISL_15055319\|2019-06-19 |
| >lcl\|Query_5806:5412-5482 | hRSV/A/Australia/VIC-RCH083/2017\|EPI_ISL_1834103\|2017-07-26 |
| >lcl\|Query_6001:5402-5472 | hRSV/A/South_Africa/NICD-R03671/2021\|EPI_ISL_12529637\|2021-03-12 |
| >lcl\|Query_5854:5369-5439 | hRSV/A/England/194980352/2019\|EPI_ISL_1834153\|2019-12-05 |
| >lcl\|Query_5755:5388-5458 | hRSV/A/England/200100153/2019\|EPI_ISL_1520410\|2019-12-24 |
| >lcl\|Query_6085:5412-5482 | hRSV/A/England/RE20000102/2020\|EPI_ISL_1647418\|2020-02-08 |
| >lcl\|Query_6077:5414-5484 | hRSV/A/England/RE20000020/2020\|EPI_ISL_1647410\|2020-01-06 |
| >lcl\|Query_6038:5389-5459 | hRSV/A/Argentina/BA-HNRG-413/2018\|EPI_ISL_15067676\|2018-06-18 |
| >lcl\|Query_6029:5431-5501 | hRSV/A/Philippines/99078/2019\|EPI_ISL_12970421\|2019-11-28 |
| >lcl\|Query_6019:5431-5501 | hRSV/A/Philippines/99068/2019\|EPI_ISL_12970411\|2019-09-14 |
| >lcl\|Query_6018:5431-5501 | hRSV/A/Philippines/99066/2019\|EPI_ISL_12970409\|2019-08-28 |
| >lcl\|Query_6016:5431-5501 | hRSV/A/Philippines/99064/2019\|EPI_ISL_12970407\|2019-08-19 |
| >lcl\|Query_5737:5389-5459 | hRSV/A/Argentina/HNRG-413/2018\|EPI_ISL_15055311\|2018-06-18 |
| >lcl\|Query_6025:5431-5501 | hRSV/A/Philippines/99074/2019\|EPI_ISL_12970417\|2019-10-19 |
| >lcl\|Query_54123:5413-5483 | hRSV/A/Argentina/BA-HNRG-353/2017\|EPI_ISL_1074120\|2017-06-14 |
| >lcl\|Query_5694:5413-5483 | hRSV/A/Argentina/BA-HNRG-353/2017\|EPI_ISL_1074120\|2017-06-14 |
| >lcl\|Query_54513:5411-5481 | hRSV/A/England/RE20000101/2020\|EPI_ISL_1647417\|2020-02-07 |
| >lcl\|Query_6084:5411-5481 | hRSV/A/England/RE20000101/2020\|EPI_ISL_1647417\|2020-02-07 |
| >lcl\|Query_54225:5398-5468 | hRSV/A/England/200200205/2020\|EPI_ISL_1647393\|2020-01-03 |
| >lcl\|Query_5796:5398-5468 | hRSV/A/England/200200205/2020\|EPI_ISL_1647393\|2020-01-03 |
| >lcl\|Query_54465:5430-5500 | hRSV/A/Belgium/SCI-VD-FD-00001/2022\|EPI_ISL_13297987\|2022-04-14 |
| >lcl\|Query_6036:5430-5500 | hRSV/A/Belgium/SCI-VD-FD-00001/2022\|EPI_ISL_13297987\|2022-04-14 |
| >lcl\|Query_54493:5392-5462 | hRSV/A/England/195200782/2019\|EPI_ISL_1520408\|2019-12-20 |
| >lcl\|Query_54468:5389-5459 | hRSV/A/Argentina/BA-HNRG-415/2018\|EPI_ISL_15067677\|2018-06-28 |
| >lcl\|Query_54291:5380-5450 | hRSV/A/England/RE20000092/2020\|EPI_ISL_1834173\|2020-01-28 |
| >lcl\|Query_54255:5429-5499 | hRSV/A/Australia/VIC-RCH138/2017\|EPI_ISL_1834125\|2017-09-20 |
| >lcl\|Query_54239:5411-5481 | hRSV/A/Australia/VIC-RCH100/2017\|EPI_ISL_1834107\|2017-08-01 |
| >lcl\|Query_54213:5401-5471 | hRSV/A/England/RE20000094/2020\|EPI_ISL_1520439\|2020-01-28 |
| >lcl\|Query_54167:5389-5459 | hRSV/A/Argentina/HNRG-415/2018\|EPI_ISL_15055312\|2018-06-28 |
| >lcl\|Query_54502:5415-5485 | hRSV/A/England/RE19003253/2019\|EPI_ISL_1647406\|2019-12-15 |
| >lcl\|Query_54466:5387-5457 | hRSV/A/Argentina/BA-HNRG-403/2018\|EPI_ISL_15067675\|2018-05-04 |
| >lcl\|Query_54378:5413-5483 | hRSV/A/Argentina/BA-HNRG-324/2017\|EPI_ISL_1074245\|2017-05-24 |
| >lcl\|Query_54366:5454-5524 | hRSV/A/Australia/VIC-VIDRL002/2018\|EPI_ISL_4602779\|2018-07-25 |
| >lcl\|Query_54271:5411-5481 | hRSV/A/Australia/VIC-RCH180/2018\|EPI_ISL_1834141\|2018-11-30 |
| >lcl\|Query_54265:5411-5481 | hRSV/A/Australia/VIC-RCH158/2019\|EPI_ISL_1834135\|2019-08-18 |
| >lcl\|Query_54261:5411-5481 | hRSV/A/Australia/VIC-RCH145/2018\|EPI_ISL_1834131\|2018-07-21 |
| >lcl\|Query_54257:5411-5481 | hRSV/A/Australia/VIC-RCH140/2018\|EPI_ISL_1834127\|2018-07-22 |
| >lcl\|Query_54254:5411-5481 | hRSV/A/Australia/VIC-RCH137/2018\|EPI_ISL_1834124\|2018-07-16 |
| >lcl\|Query_54247:5411-5481 | hRSV/A/Australia/VIC-RCH119/2018\|EPI_ISL_1834116\|2018-07-02 |
| >lcl\|Query_54241:5411-5481 | hRSV/A/Australia/VIC-RCH105/2018\|EPI_ISL_1834109\|2018-06-24 |
| >lcl\|Query_54237:5411-5481 | hRSV/A/Australia/VIC-RCH088/2018\|EPI_ISL_1834105\|2018-06-11 |
| >lcl\|Query_54234:5411-5481 | hRSV/A/Australia/VIC-RCH073/2018\|EPI_ISL_1834102\|2018-04-30 |
| >lcl\|Query_54232:5440-5510 | hRSV/A/Australia/VIC-RCH070/2019\|EPI_ISL_1834100\|2019-05-18 |
| >lcl\|Query_54210:5398-5468 | hRSV/A/England/RE20000002/2019\|EPI_ISL_1520436\|2019-12-17 |
| >lcl\|Query_54206:5413-5483 | hRSV/A/England/RE19003255/2019\|EPI_ISL_1520432\|2019-12-13 |
| >lcl\|Query_54165:5387-5457 | hRSV/A/Argentina/HNRG-403/2018\|EPI_ISL_15055310\|2018-05-04 |
| >lcl\|Query_54125:5423-5493 | hRSV/A/Argentina/BA-HNRG-376/2017\|EPI_ISL_1074127\|2017-06-30 |
| >lcl\|Query_54482:5389-5459 | hRSV/A/Argentina/BA-HNRG-452/2021\|EPI_ISL_15067691\|2021-08-03 |
| >lcl\|Query_54472:5390-5460 | hRSV/A/Argentina/BA-HNRG-433/2019\|EPI_ISL_15067681\|2019-06-06 |
| >lcl\|Query_54470:5387-5457 | hRSV/A/Argentina/BA-HNRG-419/2018\|EPI_ISL_15067679\|2018-07-11 |
| >lcl\|Query_54469:5387-5457 | hRSV/A/Argentina/BA-HNRG-416/2018\|EPI_ISL_15067678\|2018-06-29 |
| >lcl\|Query_54374:5416-5486 | hRSV/A/Argentina/BA-HNRG-347/2017\|EPI_ISL_1074212\|2017-06-12 |
| >lcl\|Query_54364:5359-5429 | hRSV/A/Australia/2001479/2020\|EPI_ISL_2839408\|2020-10-21 |
| >lcl\|Query_54341:5443-5513 | hRSV/A/Australia/1514/2021\|EPI_ISL_2835616\|2021-01-18 |
| >lcl\|Query_54262:5411-5481 | hRSV/A/Australia/VIC-RCH149/2018\|EPI_ISL_1834132\|2018-07-30 |
| >lcl\|Query_54259:5412-5482 | hRSV/A/Australia/VIC-RCH142/2019\|EPI_ISL_1834129\|2019-08-12 |
| >lcl\|Query_54258:5411-5481 | hRSV/A/Australia/VIC-RCH141/2018\|EPI_ISL_1834128\|2018-07-22 |
| >lcl\|Query_54252:5411-5481 | hRSV/A/Australia/VIC-RCH133/2018\|EPI_ISL_1834121\|2018-07-14 |
| >lcl\|Query_54181:5389-5459 | hRSV/A/Argentina/HNRG-452/2021\|EPI_ISL_15055326\|2021-08-03 |
| >lcl\|Query_54171:5390-5460 | hRSV/A/Argentina/HNRG-433/2019\|EPI_ISL_15055316\|2019-06-06 |
| >lcl\|Query_54169:5387-5457 | hRSV/A/Argentina/HNRG-419/2018\|EPI_ISL_15055314\|2018-07-11 |
| >lcl\|Query_54168:5387-5457 | hRSV/A/Argentina/HNRG-416/2018\|EPI_ISL_15055313\|2018-06-29 |
| >lcl\|Query_54122:5421-5491 | hRSV/A/Argentina/BA-HNRG-365/2017\|EPI_ISL_1074115\|2017-06-22 |
| >lcl\|Query_54121:5420-5490 | hRSV/A/Argentina/BA-HNRG-382/2017\|EPI_ISL_1074112\|2017-07-05 |
| >lcl\|Query_54525:5412-5482 | hRSV/A/Mozambique/MAP-INS014/2017\|EPI_ISL_1647462\|2017-02-14 |
| >lcl\|Query_54365:5359-5429 | hRSV/A/Australia/7003912/2020\|EPI_ISL_2839409\|2020-11-17 |
| >lcl\|Query_54362:5359-5429 | hRSV/A/Australia/6003147/2020\|EPI_ISL_2839406\|2020-12-06 |
| >lcl\|Query_54361:5359-5429 | hRSV/A/Australia/4009301/2020\|EPI_ISL_2839405\|2020-12-04 |
| >lcl\|Query_54360:5439-5509 | hRSV/A/Australia/3002687/2021\|EPI_ISL_2839404\|2021-01-01 |
| >lcl\|Query_54359:5434-5504 | hRSV/A/Australia/1010209/2021\|EPI_ISL_2839403\|2021-01-11 |
| >lcl\|Query_54358:5443-5513 | hRSV/A/Australia/3004/2021\|EPI_ISL_2839402\|2021-01-09 |
| >lcl\|Query_54357:5442-5512 | hRSV/A/Australia/5009934/2021\|EPI_ISL_2839401\|2021-01-04 |
| >lcl\|Query_54356:5443-5513 | hRSV/A/Australia/3002650/2021\|EPI_ISL_2839400\|2021-01-01 |
| >lcl\|Query_54253:5411-5481 | hRSV/A/Australia/VIC-RCH135/2018\|EPI_ISL_1834123\|2018-07-14 |
| >lcl\|Query_54363:5359-5429 | hRSV/A/Australia/9002436/2020\|EPI_ISL_2839407\|2020-11-28 |
| >lcl\|Query_54242:5412-5482 | hRSV/A/Australia/VIC-RCH107/2019\|EPI_ISL_1834110\|2019-07-07 |
| >lcl\|Query_54233:5413-5483 | hRSV/A/Australia/VIC-RCH072/2019\|EPI_ISL_1834101\|2019-05-13 |
| >lcl\|Query_6064:5392-5462 | hRSV/A/England/195200782/2019\|EPI_ISL_1520408\|2019-12-20 |
| >lcl\|Query_6039:5389-5459 | hRSV/A/Argentina/BA-HNRG-415/2018\|EPI_ISL_15067677\|2018-06-28 |
| >lcl\|Query_5862:5380-5450 | hRSV/A/England/RE20000092/2020\|EPI_ISL_1834173\|2020-01-28 |
| >lcl\|Query_5826:5429-5499 | hRSV/A/Australia/VIC-RCH138/2017\|EPI_ISL_1834125\|2017-09-20 |
| >lcl\|Query_5810:5411-5481 | hRSV/A/Australia/VIC-RCH100/2017\|EPI_ISL_1834107\|2017-08-01 |
| >lcl\|Query_5784:5401-5471 | hRSV/A/England/RE20000094/2020\|EPI_ISL_1520439\|2020-01-28 |
| >lcl\|Query_5738:5389-5459 | hRSV/A/Argentina/HNRG-415/2018\|EPI_ISL_15055312\|2018-06-28 |
| >lcl\|Query_6073:5415-5485 | hRSV/A/England/RE19003253/2019\|EPI_ISL_1647406\|2019-12-15 |
| >lcl\|Query_6037:5387-5457 | hRSV/A/Argentina/BA-HNRG-403/2018\|EPI_ISL_15067675\|2018-05-04 |
| >lcl\|Query_5949:5413-5483 | hRSV/A/Argentina/BA-HNRG-324/2017\|EPI_ISL_1074245\|2017-05-24 |
| >lcl\|Query_5937:5454-5524 | hRSV/A/Australia/VIC-VIDRL002/2018\|EPI_ISL_4602779\|2018-07-25 |
| >lcl\|Query_5842:5411-5481 | hRSV/A/Australia/VIC-RCH180/2018\|EPI_ISL_1834141\|2018-11-30 |
| >lcl\|Query_5836:5411-5481 | hRSV/A/Australia/VIC-RCH158/2019\|EPI_ISL_1834135\|2019-08-18 |
| >lcl\|Query_5832:5411-5481 | hRSV/A/Australia/VIC-RCH145/2018\|EPI_ISL_1834131\|2018-07-21 |
| >lcl\|Query_5828:5411-5481 | hRSV/A/Australia/VIC-RCH140/2018\|EPI_ISL_1834127\|2018-07-22 |
| >lcl\|Query_5825:5411-5481 | hRSV/A/Australia/VIC-RCH137/2018\|EPI_ISL_1834124\|2018-07-16 |
| >lcl\|Query_5818:5411-5481 | hRSV/A/Australia/VIC-RCH119/2018\|EPI_ISL_1834116\|2018-07-02 |
| >lcl\|Query_5812:5411-5481 | hRSV/A/Australia/VIC-RCH105/2018\|EPI_ISL_1834109\|2018-06-24 |
| >lcl\|Query_5808:5411-5481 | hRSV/A/Australia/VIC-RCH088/2018\|EPI_ISL_1834105\|2018-06-11 |
| >lcl\|Query_5805:5411-5481 | hRSV/A/Australia/VIC-RCH073/2018\|EPI_ISL_1834102\|2018-04-30 |
| >lcl\|Query_5803:5440-5510 | hRSV/A/Australia/VIC-RCH070/2019\|EPI_ISL_1834100\|2019-05-18 |
| >lcl\|Query_5781:5398-5468 | hRSV/A/England/RE20000002/2019\|EPI_ISL_1520436\|2019-12-17 |
| >lcl\|Query_5777:5413-5483 | hRSV/A/England/RE19003255/2019\|EPI_ISL_1520432\|2019-12-13 |
| >lcl\|Query_5736:5387-5457 | hRSV/A/Argentina/HNRG-403/2018\|EPI_ISL_15055310\|2018-05-04 |
| >lcl\|Query_5696:5423-5493 | hRSV/A/Argentina/BA-HNRG-376/2017\|EPI_ISL_1074127\|2017-06-30 |
| >lcl\|Query_6053:5389-5459 | hRSV/A/Argentina/BA-HNRG-452/2021\|EPI_ISL_15067691\|2021-08-03 |
| >lcl\|Query_6043:5390-5460 | hRSV/A/Argentina/BA-HNRG-433/2019\|EPI_ISL_15067681\|2019-06-06 |
| >lcl\|Query_6041:5387-5457 | hRSV/A/Argentina/BA-HNRG-419/2018\|EPI_ISL_15067679\|2018-07-11 |
| >lcl\|Query_6040:5387-5457 | hRSV/A/Argentina/BA-HNRG-416/2018\|EPI_ISL_15067678\|2018-06-29 |
| >lcl\|Query_5945:5416-5486 | hRSV/A/Argentina/BA-HNRG-347/2017\|EPI_ISL_1074212\|2017-06-12 |
| >lcl\|Query_5935:5359-5429 | hRSV/A/Australia/2001479/2020\|EPI_ISL_2839408\|2020-10-21 |
| >lcl\|Query_5912:5443-5513 | hRSV/A/Australia/1514/2021\|EPI_ISL_2835616\|2021-01-18 |
| >lcl\|Query_5833:5411-5481 | hRSV/A/Australia/VIC-RCH149/2018\|EPI_ISL_1834132\|2018-07-30 |
| >lcl\|Query_5830:5412-5482 | hRSV/A/Australia/VIC-RCH142/2019\|EPI_ISL_1834129\|2019-08-12 |
| >lcl\|Query_5829:5411-5481 | hRSV/A/Australia/VIC-RCH141/2018\|EPI_ISL_1834128\|2018-07-22 |
| >lcl\|Query_5823:5411-5481 | hRSV/A/Australia/VIC-RCH133/2018\|EPI_ISL_1834121\|2018-07-14 |
| >lcl\|Query_5752:5389-5459 | hRSV/A/Argentina/HNRG-452/2021\|EPI_ISL_15055326\|2021-08-03 |
| >lcl\|Query_5742:5390-5460 | hRSV/A/Argentina/HNRG-433/2019\|EPI_ISL_15055316\|2019-06-06 |
| >lcl\|Query_5740:5387-5457 | hRSV/A/Argentina/HNRG-419/2018\|EPI_ISL_15055314\|2018-07-11 |
| >lcl\|Query_5739:5387-5457 | hRSV/A/Argentina/HNRG-416/2018\|EPI_ISL_15055313\|2018-06-29 |
| >lcl\|Query_5693:5421-5491 | hRSV/A/Argentina/BA-HNRG-365/2017\|EPI_ISL_1074115\|2017-06-22 |
| >lcl\|Query_5692:5420-5490 | hRSV/A/Argentina/BA-HNRG-382/2017\|EPI_ISL_1074112\|2017-07-05 |
| >lcl\|Query_6096:5412-5482 | hRSV/A/Mozambique/MAP-INS014/2017\|EPI_ISL_1647462\|2017-02-14 |
| >lcl\|Query_5936:5359-5429 | hRSV/A/Australia/7003912/2020\|EPI_ISL_2839409\|2020-11-17 |
| >lcl\|Query_5933:5359-5429 | hRSV/A/Australia/6003147/2020\|EPI_ISL_2839406\|2020-12-06 |
| >lcl\|Query_5932:5359-5429 | hRSV/A/Australia/4009301/2020\|EPI_ISL_2839405\|2020-12-04 |
| >lcl\|Query_5931:5439-5509 | hRSV/A/Australia/3002687/2021\|EPI_ISL_2839404\|2021-01-01 |
| >lcl\|Query_5930:5434-5504 | hRSV/A/Australia/1010209/2021\|EPI_ISL_2839403\|2021-01-11 |
| >lcl\|Query_5929:5443-5513 | hRSV/A/Australia/3004/2021\|EPI_ISL_2839402\|2021-01-09 |
| >lcl\|Query_5928:5442-5512 | hRSV/A/Australia/5009934/2021\|EPI_ISL_2839401\|2021-01-04 |
| >lcl\|Query_5927:5443-5513 | hRSV/A/Australia/3002650/2021\|EPI_ISL_2839400\|2021-01-01 |
| >lcl\|Query_5824:5411-5481 | hRSV/A/Australia/VIC-RCH135/2018\|EPI_ISL_1834123\|2018-07-14 |
| >lcl\|Query_5934:5359-5429 | hRSV/A/Australia/9002436/2020\|EPI_ISL_2839407\|2020-11-28 |
| >lcl\|Query_5813:5412-5482 | hRSV/A/Australia/VIC-RCH107/2019\|EPI_ISL_1834110\|2019-07-07 |
| >lcl\|Query_5804:5413-5483 | hRSV/A/Australia/VIC-RCH072/2019\|EPI_ISL_1834101\|2019-05-13 |
| >lcl\|Query_54250:5429-5499 | hRSV/A/Australia/VIC-RCH126/2017\|EPI_ISL_1834119\|2017-08-29 |
| >lcl\|Query_54244:5411-5481 | hRSV/A/Australia/VIC-RCH113/2018\|EPI_ISL_1834113\|2018-07-01 |
| >lcl\|Query_54518:5416-5486 | hRSV/A/England/RE19003244/2019\|EPI_ISL_1647422\|2020-12-16 |
| >lcl\|Query_54509:5388-5458 | hRSV/A/England/RE20000025/2020\|EPI_ISL_1647413\|2020-01-09 |
| >lcl\|Query_54508:5415-5485 | hRSV/A/England/RE20000023/2020\|EPI_ISL_1647412\|2020-01-06 |
| >lcl\|Query_54505:5415-5485 | hRSV/A/England/RE20000014/2020\|EPI_ISL_1647409\|2020-01-02 |
| >lcl\|Query_54503:5401-5471 | hRSV/A/England/RE20000010/2019\|EPI_ISL_1647407\|2019-12-29 |
| >lcl\|Query_54501:5416-5486 | hRSV/A/England/RE19003244/2019\|EPI_ISL_1647405\|2019-12-06 |
| >lcl\|Query_54498:5429-5499 | hRSV/A/England/RE19003150/2019\|EPI_ISL_1647402\|2019-11-15 |
| >lcl\|Query_54497:5416-5486 | hRSV/A/England/RE19003148/2019\|EPI_ISL_1647401\|2019-11-20 |
| >lcl\|Query_54276:5411-5481 | hRSV/A/Australia/VIC-RCH187/2018\|EPI_ISL_1834146\|2018-11-11 |
| >lcl\|Query_54272:5411-5481 | hRSV/A/Australia/VIC-RCH181/2018\|EPI_ISL_1834142\|2018-10-06 |
| >lcl\|Query_54230:5414-5484 | hRSV/A/England/RE19003140/2019\|EPI_ISL_1647398\|2019-11-04 |
| >lcl\|Query_54229:5414-5484 | hRSV/A/England/RE19003139/2019\|EPI_ISL_1647397\|2019-12-01 |
| >lcl\|Query_54227:5415-5485 | hRSV/A/England/RE19003127/2019\|EPI_ISL_1647395\|2019-11-27 |
| >lcl\|Query_54222:5415-5485 | hRSV/A/England/195020407/2019\|EPI_ISL_1647390\|2019-12-06 |
| >lcl\|Query_54215:5412-5482 | hRSV/A/England/194380302/2019\|EPI_ISL_1647383\|2019-10-22 |
| >lcl\|Query_54207:5413-5483 | hRSV/A/England/RE19003256/2019\|EPI_ISL_1520433\|2019-12-17 |
| >lcl\|Query_54205:5414-5484 | hRSV/A/England/RE19003243/2019\|EPI_ISL_1520431\|2019-12-06 |
| >lcl\|Query_54204:5413-5483 | hRSV/A/England/RE19003240/2019\|EPI_ISL_1520430\|2019-12-12 |
| >lcl\|Query_54199:5374-5444 | hRSV/A/England/RE19003145/2019\|EPI_ISL_1520425\|2019-11-29 |
| >lcl\|Query_54198:5415-5485 | hRSV/A/England/RE19003141/2019\|EPI_ISL_1520424\|2019-12-05 |
| >lcl\|Query_54197:5414-5484 | hRSV/A/England/RE19003138/2019\|EPI_ISL_1520423\|2019-12-02 |
| >lcl\|Query_54195:5415-5485 | hRSV/A/England/RE19003133/2019\|EPI_ISL_1520421\|2019-11-25 |
| >lcl\|Query_54194:5414-5484 | hRSV/A/England/RE19003132/2019\|EPI_ISL_1520420\|2019-11-23 |
| >lcl\|Query_54193:5412-5482 | hRSV/A/England/RE19003131/2019\|EPI_ISL_1520419\|2019-10-21 |
| >lcl\|Query_54192:5413-5483 | hRSV/A/England/RE19003130/2019\|EPI_ISL_1520418\|2019-12-04 |
| >lcl\|Query_54189:5412-5482 | hRSV/A/England/RE19003124/2019\|EPI_ISL_1520415\|2019-11-30 |
| >lcl\|Query_54500:5415-5485 | hRSV/A/England/RE19003238/2019\|EPI_ISL_1647404\|2019-12-02 |
| >lcl\|Query_54228:5416-5486 | hRSV/A/England/RE19003136/2019\|EPI_ISL_1647396\|2019-11-16 |
| >lcl\|Query_54226:5415-5485 | hRSV/A/England/RE19003126/2019\|EPI_ISL_1647394\|2019-11-15 |
| >lcl\|Query_54202:5415-5485 | hRSV/A/England/RE19003236/2019\|EPI_ISL_1520428\|2019-12-05 |
| >lcl\|Query_54200:5409-5479 | hRSV/A/England/RE19003149/2019\|EPI_ISL_1520426\|2019-11-22 |
| >lcl\|Query_54196:5403-5473 | hRSV/A/England/RE19003137/2019\|EPI_ISL_1520422\|2019-12-04 |
| >lcl\|Query_54190:5415-5485 | hRSV/A/England/RE19003125/2019\|EPI_ISL_1520416\|2019-11-13 |
| >lcl\|Query_54187:5415-5485 | hRSV/A/England/RE19003122/2019\|EPI_ISL_1520413\|2019-11-12 |
| >lcl\|Query_54146:5416-5486 | hRSV/A/England/E19003242/2019\|EPI_ISL_11428328\|2019-12-16 |
| >lcl\|Query_54415:5434-5504 | hRSV/A/Australia/QLD-RBWH230/2021\|EPI_ISL_11817071\|2021-03-15 |
| >lcl\|Query_54401:5434-5504 | hRSV/A/Australia/QLD-RBWH067/2021\|EPI_ISL_11817042\|2021-02-07 |
| >lcl\|Query_54386:5434-5504 | hRSV/A/Australia/QLD-RBWH008/2021\|EPI_ISL_11817019\|2021-01-08 |
| >lcl\|Query_5821:5429-5499 | hRSV/A/Australia/VIC-RCH126/2017\|EPI_ISL_1834119\|2017-08-29 |
| >lcl\|Query_5815:5411-5481 | hRSV/A/Australia/VIC-RCH113/2018\|EPI_ISL_1834113\|2018-07-01 |
| >lcl\|Query_6089:5416-5486 | hRSV/A/England/RE19003244/2019\|EPI_ISL_1647422\|2020-12-16 |
| >lcl\|Query_6080:5388-5458 | hRSV/A/England/RE20000025/2020\|EPI_ISL_1647413\|2020-01-09 |
| >lcl\|Query_6079:5415-5485 | hRSV/A/England/RE20000023/2020\|EPI_ISL_1647412\|2020-01-06 |
| >lcl\|Query_6076:5415-5485 | hRSV/A/England/RE20000014/2020\|EPI_ISL_1647409\|2020-01-02 |
| >lcl\|Query_6074:5401-5471 | hRSV/A/England/RE20000010/2019\|EPI_ISL_1647407\|2019-12-29 |
| >lcl\|Query_6072:5416-5486 | hRSV/A/England/RE19003244/2019\|EPI_ISL_1647405\|2019-12-06 |
| >lcl\|Query_6069:5429-5499 | hRSV/A/England/RE19003150/2019\|EPI_ISL_1647402\|2019-11-15 |
| >lcl\|Query_6068:5416-5486 | hRSV/A/England/RE19003148/2019\|EPI_ISL_1647401\|2019-11-20 |
| >lcl\|Query_5847:5411-5481 | hRSV/A/Australia/VIC-RCH187/2018\|EPI_ISL_1834146\|2018-11-11 |
| >lcl\|Query_5843:5411-5481 | hRSV/A/Australia/VIC-RCH181/2018\|EPI_ISL_1834142\|2018-10-06 |
| >lcl\|Query_5801:5414-5484 | hRSV/A/England/RE19003140/2019\|EPI_ISL_1647398\|2019-11-04 |
| >lcl\|Query_5800:5414-5484 | hRSV/A/England/RE19003139/2019\|EPI_ISL_1647397\|2019-12-01 |
| >lcl\|Query_5798:5415-5485 | hRSV/A/England/RE19003127/2019\|EPI_ISL_1647395\|2019-11-27 |
| >lcl\|Query_5793:5415-5485 | hRSV/A/England/195020407/2019\|EPI_ISL_1647390\|2019-12-06 |
| >lcl\|Query_5786:5412-5482 | hRSV/A/England/194380302/2019\|EPI_ISL_1647383\|2019-10-22 |
| >lcl\|Query_5778:5413-5483 | hRSV/A/England/RE19003256/2019\|EPI_ISL_1520433\|2019-12-17 |
| >lcl\|Query_5776:5414-5484 | hRSV/A/England/RE19003243/2019\|EPI_ISL_1520431\|2019-12-06 |
| >lcl\|Query_5775:5413-5483 | hRSV/A/England/RE19003240/2019\|EPI_ISL_1520430\|2019-12-12 |
| >lcl\|Query_5770:5374-5444 | hRSV/A/England/RE19003145/2019\|EPI_ISL_1520425\|2019-11-29 |
| >lcl\|Query_5769:5415-5485 | hRSV/A/England/RE19003141/2019\|EPI_ISL_1520424\|2019-12-05 |
| >lcl\|Query_5768:5414-5484 | hRSV/A/England/RE19003138/2019\|EPI_ISL_1520423\|2019-12-02 |
| >lcl\|Query_5766:5415-5485 | hRSV/A/England/RE19003133/2019\|EPI_ISL_1520421\|2019-11-25 |
| >lcl\|Query_5765:5414-5484 | hRSV/A/England/RE19003132/2019\|EPI_ISL_1520420\|2019-11-23 |
| >lcl\|Query_5764:5412-5482 | hRSV/A/England/RE19003131/2019\|EPI_ISL_1520419\|2019-10-21 |
| >lcl\|Query_5763:5413-5483 | hRSV/A/England/RE19003130/2019\|EPI_ISL_1520418\|2019-12-04 |
| >lcl\|Query_5760:5412-5482 | hRSV/A/England/RE19003124/2019\|EPI_ISL_1520415\|2019-11-30 |
| >lcl\|Query_6071:5415-5485 | hRSV/A/England/RE19003238/2019\|EPI_ISL_1647404\|2019-12-02 |
| >lcl\|Query_5799:5416-5486 | hRSV/A/England/RE19003136/2019\|EPI_ISL_1647396\|2019-11-16 |
| >lcl\|Query_5797:5415-5485 | hRSV/A/England/RE19003126/2019\|EPI_ISL_1647394\|2019-11-15 |
| >lcl\|Query_5773:5415-5485 | hRSV/A/England/RE19003236/2019\|EPI_ISL_1520428\|2019-12-05 |
| >lcl\|Query_5771:5409-5479 | hRSV/A/England/RE19003149/2019\|EPI_ISL_1520426\|2019-11-22 |
| >lcl\|Query_5767:5403-5473 | hRSV/A/England/RE19003137/2019\|EPI_ISL_1520422\|2019-12-04 |
| >lcl\|Query_5761:5415-5485 | hRSV/A/England/RE19003125/2019\|EPI_ISL_1520416\|2019-11-13 |
| >lcl\|Query_5758:5415-5485 | hRSV/A/England/RE19003122/2019\|EPI_ISL_1520413\|2019-11-12 |
| >lcl\|Query_5717:5416-5486 | hRSV/A/England/E19003242/2019\|EPI_ISL_11428328\|2019-12-16 |
| >lcl\|Query_5986:5434-5504 | hRSV/A/Australia/QLD-RBWH230/2021\|EPI_ISL_11817071\|2021-03-15 |
| >lcl\|Query_5972:5434-5504 | hRSV/A/Australia/QLD-RBWH067/2021\|EPI_ISL_11817042\|2021-02-07 |
| >lcl\|Query_5957:5434-5504 | hRSV/A/Australia/QLD-RBWH008/2021\|EPI_ISL_11817019\|2021-01-08 |
| >lcl\|Query_54248:5411-5481 | hRSV/A/Australia/VIC-RCH120/2017\|EPI_ISL_1834117\|2017-08-20 |
| >lcl\|Query_54264:5411-5481 | hRSV/A/Australia/VIC-RCH151/2018\|EPI_ISL_1834134\|2018-07-29 |
| >lcl\|Query_5819:5411-5481 | hRSV/A/Australia/VIC-RCH120/2017\|EPI_ISL_1834117\|2017-08-20 |
| >lcl\|Query_5835:5411-5481 | hRSV/A/Australia/VIC-RCH151/2018\|EPI_ISL_1834134\|2018-07-29 |
| >lcl\|Query_54492:5406-5476 | hRSV/A/England/195200145/2019\|EPI_ISL_1520407\|2019-12-20 |
| >lcl\|Query_6063:5406-5476 | hRSV/A/England/195200145/2019\|EPI_ISL_1520407\|2019-12-20 |
| >lcl\|Query_54377:5422-5492 | hRSV/A/Argentina/BA-HNRG-355/2017\|EPI_ISL_1074234\|2017-06-16 |
| >lcl\|Query_54137:5414-5484 | hRSV/A/Argentina/BA-HNRG-320/2017\|EPI_ISL_1074169\|2017-05-19 |
| >lcl\|Query_5948:5422-5492 | hRSV/A/Argentina/BA-HNRG-355/2017\|EPI_ISL_1074234\|2017-06-16 |
| >lcl\|Query_5708:5414-5484 | hRSV/A/Argentina/BA-HNRG-320/2017\|EPI_ISL_1074169\|2017-05-19 |
| >lcl\|Query_54119:5423-5493 | hRSV/A/Argentina/BA-HNRG-340/2017\|EPI_ISL_1074103\|2017-06-05 |
| >lcl\|Query_5690:5423-5493 | hRSV/A/Argentina/BA-HNRG-340/2017\|EPI_ISL_1074103\|2017-06-05 |
| >lcl\|Query_54512:5414-5484 | hRSV/A/England/RE20000100/2020\|EPI_ISL_1647416\|2020-01-26 |
| >lcl\|Query_54489:5367-5437 | hRSV/A/England/195180530/2019\|EPI_ISL_1520404\|2019-12-18 |
| >lcl\|Query_54186:5362-5432 | hRSV/A/England/200260431/2020\|EPI_ISL_1520412\|2020-01-07 |
| >lcl\|Query_6083:5414-5484 | hRSV/A/England/RE20000100/2020\|EPI_ISL_1647416\|2020-01-26 |
| >lcl\|Query_6060:5367-5437 | hRSV/A/England/195180530/2019\|EPI_ISL_1520404\|2019-12-18 |
| >lcl\|Query_5757:5362-5432 | hRSV/A/England/200260431/2020\|EPI_ISL_1520412\|2020-01-07 |
| >lcl\|Query_54134:5416-5486 | hRSV/A/Argentina/BA-HNRG-316/2017\|EPI_ISL_1074159\|2017-05-15 |
| >lcl\|Query_5705:5416-5486 | hRSV/A/Argentina/BA-HNRG-316/2017\|EPI_ISL_1074159\|2017-05-15 |
| >lcl\|Query_54523:5414-5484 | hRSV/A/Mozambique/MAP-INS011/2018\|EPI_ISL_1647460\|2018-05-10 |
| >lcl\|Query_54522:5411-5481 | hRSV/A/Mozambique/MAP-INS009/2018\|EPI_ISL_1647459\|2018-04-17 |
| >lcl\|Query_54520:5414-5484 | hRSV/A/Mozambique/MAP-INS006/2018\|EPI_ISL_1647457\|2018-04-03 |
| >lcl\|Query_54524:5412-5482 | hRSV/A/Mozambique/MAP-INS012/2018\|EPI_ISL_1647461\|2018-05-11 |
| >lcl\|Query_6094:5414-5484 | hRSV/A/Mozambique/MAP-INS011/2018\|EPI_ISL_1647460\|2018-05-10 |
| >lcl\|Query_6093:5411-5481 | hRSV/A/Mozambique/MAP-INS009/2018\|EPI_ISL_1647459\|2018-04-17 |
| >lcl\|Query_6091:5414-5484 | hRSV/A/Mozambique/MAP-INS006/2018\|EPI_ISL_1647457\|2018-04-03 |
| >lcl\|Query_6095:5412-5482 | hRSV/A/Mozambique/MAP-INS012/2018\|EPI_ISL_1647461\|2018-05-11 |
| >lcl\|Query_54441:5368-5438 | hRSV/A/South_Africa/NICD-R04582/2021\|EPI_ISL_12529648\|2021-04-08 |
| >lcl\|Query_6012:5368-5438 | hRSV/A/South_Africa/NICD-R04582/2021\|EPI_ISL_12529648\|2021-04-08 |
| >lcl\|Query_54376:5421-5491 | hRSV/A/Argentina/BA-HNRG-385/2017\|EPI_ISL_1074214\|2017-07-10 |
| >lcl\|Query_5947:5421-5491 | hRSV/A/Argentina/BA-HNRG-385/2017\|EPI_ISL_1074214\|2017-07-10 |
| >lcl\|Query_54483:5388-5458 | hRSV/A/Argentina/BA-HNRG-453/2021\|EPI_ISL_15067692\|2021-08-09 |
| >lcl\|Query_54481:5388-5458 | hRSV/A/Argentina/BA-HNRG-448/2021\|EPI_ISL_15067690\|2021-07-19 |
| >lcl\|Query_54480:5388-5458 | hRSV/A/Argentina/BA-HNRG-443/2021\|EPI_ISL_15067689\|2021-05-31 |
| >lcl\|Query_54479:5388-5458 | hRSV/A/Argentina/BA-HNRG-442/2021\|EPI_ISL_15067688\|2021-05-05 |
| >lcl\|Query_54182:5388-5458 | hRSV/A/Argentina/HNRG-453/2021\|EPI_ISL_15055327\|2021-08-09 |
| >lcl\|Query_54180:5388-5458 | hRSV/A/Argentina/HNRG-448/2021\|EPI_ISL_15055325\|2021-07-19 |
| >lcl\|Query_54179:5388-5458 | hRSV/A/Argentina/HNRG-443/2021\|EPI_ISL_15055324\|2021-05-31 |
| >lcl\|Query_54178:5388-5458 | hRSV/A/Argentina/HNRG-442/2021\|EPI_ISL_15055323\|2021-05-05 |
| >lcl\|Query_54488:5394-5464 | hRSV/A/England/195180517/2019\|EPI_ISL_1520403\|2019-12-17 |
| >lcl\|Query_6054:5388-5458 | hRSV/A/Argentina/BA-HNRG-453/2021\|EPI_ISL_15067692\|2021-08-09 |
| >lcl\|Query_6052:5388-5458 | hRSV/A/Argentina/BA-HNRG-448/2021\|EPI_ISL_15067690\|2021-07-19 |
| >lcl\|Query_6051:5388-5458 | hRSV/A/Argentina/BA-HNRG-443/2021\|EPI_ISL_15067689\|2021-05-31 |
| >lcl\|Query_6050:5388-5458 | hRSV/A/Argentina/BA-HNRG-442/2021\|EPI_ISL_15067688\|2021-05-05 |
| >lcl\|Query_5753:5388-5458 | hRSV/A/Argentina/HNRG-453/2021\|EPI_ISL_15055327\|2021-08-09 |
| >lcl\|Query_5751:5388-5458 | hRSV/A/Argentina/HNRG-448/2021\|EPI_ISL_15055325\|2021-07-19 |
| >lcl\|Query_5750:5388-5458 | hRSV/A/Argentina/HNRG-443/2021\|EPI_ISL_15055324\|2021-05-31 |
| >lcl\|Query_5749:5388-5458 | hRSV/A/Argentina/HNRG-442/2021\|EPI_ISL_15055323\|2021-05-05 |
| >lcl\|Query_6059:5394-5464 | hRSV/A/England/195180517/2019\|EPI_ISL_1520403\|2019-12-17 |
| >lcl\|Query_54484:5388-5458 | hRSV/A/Argentina/BA-HNRG-455/2021\|EPI_ISL_15067693\|2021-08-29 |
| >lcl\|Query_54183:5388-5458 | hRSV/A/Argentina/HNRG-455/2021\|EPI_ISL_15055328\|2021-08-29 |
| >lcl\|Query_6055:5388-5458 | hRSV/A/Argentina/BA-HNRG-455/2021\|EPI_ISL_15067693\|2021-08-29 |
| >lcl\|Query_5754:5388-5458 | hRSV/A/Argentina/HNRG-455/2021\|EPI_ISL_15055328\|2021-08-29 |
| >lcl\|Query_54267:5412-5482 | hRSV/A/Australia/VIC-RCH166/2018\|EPI_ISL_1834137\|2018-08-27 |
| >lcl\|Query_5838:5412-5482 | hRSV/A/Australia/VIC-RCH166/2018\|EPI_ISL_1834137\|2018-08-27 |
| >lcl\|Query_54214:5414-5484 | hRSV/A/England/RE20000103/2020\|EPI_ISL_1520440\|2020-01-24 |
| >lcl\|Query_5785:5414-5484 | hRSV/A/England/RE20000103/2020\|EPI_ISL_1520440\|2020-01-24 |
| >lcl\|Query_54382:5417-5487 | hRSV/A/Argentina/BA-HNRG-338/2017\|EPI_ISL_1074256\|2017-06-02 |
| >lcl\|Query_54138:5417-5487 | hRSV/A/Argentina/BA-HNRG-323/2017\|EPI_ISL_1074170\|2017-05-22 |
| >lcl\|Query_54128:5422-5492 | hRSV/A/Argentina/BA-HNRG-321/2017\|EPI_ISL_1074152\|2017-05-22 |
| >lcl\|Query_54126:5417-5487 | hRSV/A/Argentina/BA-HNRG-330/2017\|EPI_ISL_1074137\|2017-05-29 |
| >lcl\|Query_5953:5417-5487 | hRSV/A/Argentina/BA-HNRG-338/2017\|EPI_ISL_1074256\|2017-06-02 |
| >lcl\|Query_5709:5417-5487 | hRSV/A/Argentina/BA-HNRG-323/2017\|EPI_ISL_1074170\|2017-05-22 |
| >lcl\|Query_5699:5422-5492 | hRSV/A/Argentina/BA-HNRG-321/2017\|EPI_ISL_1074152\|2017-05-22 |
| >lcl\|Query_5697:5417-5487 | hRSV/A/Argentina/BA-HNRG-330/2017\|EPI_ISL_1074137\|2017-05-29 |
| >lcl\|Query_54307:5409-5479 | hRSV/A/Cote_d'Ivoire/IPCI-022/2017\|EPI_ISL_2543815\|2017-09-04 |
| >lcl\|Query_5878:5409-5479 | hRSV/A/Cote_d'Ivoire/IPCI-022/2017\|EPI_ISL_2543815\|2017-09-04 |
| >lcl\|Query_54220:5381-5451 | hRSV/A/England/194660604/2019\|EPI_ISL_1647388\|2019-11-12 |
| >lcl\|Query_54442:5450-5520 | hRSV/A/Philippines/99058/2019\|EPI_ISL_12970401\|2019-03-04 |
| >lcl\|Query_54504:5419-5489 | hRSV/A/England/RE20000012/2020\|EPI_ISL_1647408\|2020-01-01 |
| >lcl\|Query_54231:5420-5490 | hRSV/A/England/RE19003143/2019\|EPI_ISL_1647399\|2019-11-12 |
| >lcl\|Query_54216:5420-5490 | hRSV/A/England/194400253/2019\|EPI_ISL_1647384\|2019-10-24 |
| >lcl\|Query_54211:5409-5479 | hRSV/A/England/RE20000009/2019\|EPI_ISL_1520437\|2019-12-29 |
| >lcl\|Query_54203:5414-5484 | hRSV/A/England/RE19003239/2019\|EPI_ISL_1520429\|2019-12-10 |
| >lcl\|Query_5791:5381-5451 | hRSV/A/England/194660604/2019\|EPI_ISL_1647388\|2019-11-12 |
| >lcl\|Query_6013:5450-5520 | hRSV/A/Philippines/99058/2019\|EPI_ISL_12970401\|2019-03-04 |
| >lcl\|Query_6075:5419-5489 | hRSV/A/England/RE20000012/2020\|EPI_ISL_1647408\|2020-01-01 |
| >lcl\|Query_5802:5420-5490 | hRSV/A/England/RE19003143/2019\|EPI_ISL_1647399\|2019-11-12 |
| >lcl\|Query_5787:5420-5490 | hRSV/A/England/194400253/2019\|EPI_ISL_1647384\|2019-10-24 |
| >lcl\|Query_5782:5409-5479 | hRSV/A/England/RE20000009/2019\|EPI_ISL_1520437\|2019-12-29 |
| >lcl\|Query_5774:5414-5484 | hRSV/A/England/RE19003239/2019\|EPI_ISL_1520429\|2019-12-10 |
| >lcl\|Query_54494:5362-5432 | hRSV/A/England/200100151/2019\|EPI_ISL_1520409\|2019-12-23 |
| >lcl\|Query_54516:5405-5475 | hRSV/A/England/RS20000580/2020\|EPI_ISL_1647420\|2020-02-05 |
| >lcl\|Query_6065:5362-5432 | hRSV/A/England/200100151/2019\|EPI_ISL_1520409\|2019-12-23 |
| >lcl\|Query_6087:5405-5475 | hRSV/A/England/RS20000580/2020\|EPI_ISL_1647420\|2020-02-05 |
| >lcl\|Query_54118:5420-5490 | hRSV/A/Argentina/BA-HNRG-307/2017\|EPI_ISL_1074100\|2017-05-02 |
| >lcl\|Query_54320:5394-5464 | hRSV/A/Mongolia/NCCD006/2018\|EPI_ISL_2543828\|2018-07-10 |
| >lcl\|Query_54238:5411-5481 | hRSV/A/Australia/VIC-RCH095/2018\|EPI_ISL_1834106\|2018-06-17 |
| >lcl\|Query_54336:5397-5467 | hRSV/A/Mongolia/NCCD039/2018\|EPI_ISL_2543844\|2018-03-07 |
| >lcl\|Query_54333:5397-5467 | hRSV/A/Mongolia/NCCD021/2018\|EPI_ISL_2543841\|2018-01-31 |
| >lcl\|Query_54324:5394-5464 | hRSV/A/Mongolia/NCCD008/2018\|EPI_ISL_2543832\|2018-01-10 |
| >lcl\|Query_54322:5397-5467 | hRSV/A/Mongolia/NCCD007/2018\|EPI_ISL_2543830\|2018-01-10 |
| >lcl\|Query_5689:5420-5490 | hRSV/A/Argentina/BA-HNRG-307/2017\|EPI_ISL_1074100\|2017-05-02 |
| >lcl\|Query_5891:5394-5464 | hRSV/A/Mongolia/NCCD006/2018\|EPI_ISL_2543828\|2018-07-10 |
| >lcl\|Query_5809:5411-5481 | hRSV/A/Australia/VIC-RCH095/2018\|EPI_ISL_1834106\|2018-06-17 |
| >lcl\|Query_5907:5397-5467 | hRSV/A/Mongolia/NCCD039/2018\|EPI_ISL_2543844\|2018-03-07 |
| >lcl\|Query_5904:5397-5467 | hRSV/A/Mongolia/NCCD021/2018\|EPI_ISL_2543841\|2018-01-31 |
| >lcl\|Query_5895:5394-5464 | hRSV/A/Mongolia/NCCD008/2018\|EPI_ISL_2543832\|2018-01-10 |
| >lcl\|Query_5893:5397-5467 | hRSV/A/Mongolia/NCCD007/2018\|EPI_ISL_2543830\|2018-01-10 |
| >lcl\|Query_54251:5410-5480 | hRSV/A/Australia/VIC-RCH132/2017\|EPI_ISL_1834120\|2017-09-08 |
| >lcl\|Query_5822:5410-5480 | hRSV/A/Australia/VIC-RCH132/2017\|EPI_ISL_1834120\|2017-09-08 |
| >lcl\|Query_54476:5387-5457 | hRSV/A/Argentina/BA-HNRG-439/2019\|EPI_ISL_15067685\|2019-07-25 |
| >lcl\|Query_54175:5387-5457 | hRSV/A/Argentina/HNRG-439/2019\|EPI_ISL_15055320\|2019-07-25 |
| >lcl\|Query_54477:5387-5457 | hRSV/A/Argentina/BA-HNRG-440/2019\|EPI_ISL_15067686\|2019-07-29 |
| >lcl\|Query_54176:5387-5457 | hRSV/A/Argentina/HNRG-440/2019\|EPI_ISL_15055321\|2019-07-29 |
| >lcl\|Query_6047:5387-5457 | hRSV/A/Argentina/BA-HNRG-439/2019\|EPI_ISL_15067685\|2019-07-25 |
| >lcl\|Query_5746:5387-5457 | hRSV/A/Argentina/HNRG-439/2019\|EPI_ISL_15055320\|2019-07-25 |
| >lcl\|Query_6048:5387-5457 | hRSV/A/Argentina/BA-HNRG-440/2019\|EPI_ISL_15067686\|2019-07-29 |
| >lcl\|Query_5747:5387-5457 | hRSV/A/Argentina/HNRG-440/2019\|EPI_ISL_15055321\|2019-07-29 |
| >lcl\|Query_54491:5391-5461 | hRSV/A/England/195200142/2019\|EPI_ISL_1520406\|2019-12-18 |
| >lcl\|Query_6062:5391-5461 | hRSV/A/England/195200142/2019\|EPI_ISL_1520406\|2019-12-18 |
| >lcl\|Query_54499:5415-5485 | hRSV/A/England/RE19003235/2019\|EPI_ISL_1647403\|2019-12-05 |
| >lcl\|Query_6070:5415-5485 | hRSV/A/England/RE19003235/2019\|EPI_ISL_1647403\|2019-12-05 |
| >lcl\|Query_54462:5431-5501 | hRSV/A/Philippines/99084/2020\|EPI_ISL_12970426\|2020-01-22 |
| >lcl\|Query_6033:5431-5501 | hRSV/A/Philippines/99084/2020\|EPI_ISL_12970426\|2020-01-22 |
| >lcl\|Query_54459:5431-5501 | hRSV/A/Philippines/99079/2019\|EPI_ISL_12970422\|2019-12-07 |
| >lcl\|Query_6030:5431-5501 | hRSV/A/Philippines/99079/2019\|EPI_ISL_12970422\|2019-12-07 |
| >lcl\|Query_54368:5406-5476 | hRSV/A/Egypt/C-CPHL001/2019\|EPI_ISL_6208719\|2019-01-06 |
| >lcl\|Query_54464:5431-5501 | hRSV/A/Philippines/99087/2020\|EPI_ISL_12970428\|2020-03-09 |
| >lcl\|Query_54461:5431-5501 | hRSV/A/Philippines/99082/2020\|EPI_ISL_12970425\|2020-01-02 |
| >lcl\|Query_54460:5431-5501 | hRSV/A/Philippines/99080/2019\|EPI_ISL_12970423\|2019-12-10 |
| >lcl\|Query_54456:5431-5501 | hRSV/A/Philippines/99076/2019\|EPI_ISL_12970419\|2019-11-09 |
| >lcl\|Query_54453:5431-5501 | hRSV/A/Philippines/99073/2019\|EPI_ISL_12970416\|2019-10-15 |
| >lcl\|Query_54451:5431-5501 | hRSV/A/Philippines/99071/2019\|EPI_ISL_12970414\|2019-10-01 |
| >lcl\|Query_54463:5431-5501 | hRSV/A/Philippines/99085/2020\|EPI_ISL_12970427\|2020-02-04 |
| >lcl\|Query_54372:5406-5476 | hRSV/A/Egypt/KB-CPHL004/2020\|EPI_ISL_6208727\|2020-01-08 |
| >lcl\|Query_54369:5411-5481 | hRSV/A/Egypt/KB-CPHL001/2020\|EPI_ISL_6208724\|2020-01-05 |
| >lcl\|Query_54332:5404-5474 | hRSV/A/Mongolia/NCCD020/2019\|EPI_ISL_2543840\|2019-03-26 |
| >lcl\|Query_54331:5403-5473 | hRSV/A/Mongolia/NCCD018/2019\|EPI_ISL_2543839\|2019-02-14 |
| >lcl\|Query_54325:5404-5474 | hRSV/A/Mongolia/NCCD008/2019\|EPI_ISL_2543833\|2019-01-17 |
| >lcl\|Query_54321:5403-5473 | hRSV/A/Mongolia/NCCD006/2019\|EPI_ISL_2543829\|2019-01-15 |
| >lcl\|Query_54319:5403-5473 | hRSV/A/Mongolia/NCCD004/2019\|EPI_ISL_2543827\|2019-01-07 |
| >lcl\|Query_54371:5402-5472 | hRSV/A/Egypt/KB-CPHL003/2020\|EPI_ISL_6208726\|2020-01-08 |
| >lcl\|Query_54370:5402-5472 | hRSV/A/Egypt/KB-CPHL002/2020\|EPI_ISL_6208725\|2020-01-08 |
| >lcl\|Query_5939:5406-5476 | hRSV/A/Egypt/C-CPHL001/2019\|EPI_ISL_6208719\|2019-01-06 |
| >lcl\|Query_6035:5431-5501 | hRSV/A/Philippines/99087/2020\|EPI_ISL_12970428\|2020-03-09 |
| >lcl\|Query_6032:5431-5501 | hRSV/A/Philippines/99082/2020\|EPI_ISL_12970425\|2020-01-02 |
| >lcl\|Query_6031:5431-5501 | hRSV/A/Philippines/99080/2019\|EPI_ISL_12970423\|2019-12-10 |
| >lcl\|Query_6027:5431-5501 | hRSV/A/Philippines/99076/2019\|EPI_ISL_12970419\|2019-11-09 |
| >lcl\|Query_6024:5431-5501 | hRSV/A/Philippines/99073/2019\|EPI_ISL_12970416\|2019-10-15 |
| >lcl\|Query_6022:5431-5501 | hRSV/A/Philippines/99071/2019\|EPI_ISL_12970414\|2019-10-01 |
| >lcl\|Query_6034:5431-5501 | hRSV/A/Philippines/99085/2020\|EPI_ISL_12970427\|2020-02-04 |
| >lcl\|Query_5943:5406-5476 | hRSV/A/Egypt/KB-CPHL004/2020\|EPI_ISL_6208727\|2020-01-08 |
| >lcl\|Query_5940:5411-5481 | hRSV/A/Egypt/KB-CPHL001/2020\|EPI_ISL_6208724\|2020-01-05 |
| >lcl\|Query_5903:5404-5474 | hRSV/A/Mongolia/NCCD020/2019\|EPI_ISL_2543840\|2019-03-26 |
| >lcl\|Query_5902:5403-5473 | hRSV/A/Mongolia/NCCD018/2019\|EPI_ISL_2543839\|2019-02-14 |
| >lcl\|Query_5896:5404-5474 | hRSV/A/Mongolia/NCCD008/2019\|EPI_ISL_2543833\|2019-01-17 |
| >lcl\|Query_5892:5403-5473 | hRSV/A/Mongolia/NCCD006/2019\|EPI_ISL_2543829\|2019-01-15 |
| >lcl\|Query_5890:5403-5473 | hRSV/A/Mongolia/NCCD004/2019\|EPI_ISL_2543827\|2019-01-07 |
| >lcl\|Query_5942:5402-5472 | hRSV/A/Egypt/KB-CPHL003/2020\|EPI_ISL_6208726\|2020-01-08 |
| >lcl\|Query_5941:5402-5472 | hRSV/A/Egypt/KB-CPHL002/2020\|EPI_ISL_6208725\|2020-01-08 |
| >lcl\|Query_54145:5131-5201 | hRSV/A/England/194860823/2019\|EPI_ISL_11428318\|2019-11-27 |
| >lcl\|Query_5716:5131-5201 | hRSV/A/England/194860823/2019\|EPI_ISL_11428318\|2019-11-27 |
| >lcl\|Query_54367:5454-5524 | hRSV/A/Cote_d'Ivoire/IPCI-014/2019\|EPI_ISL_5522630\|2019-08-27 |
| >lcl\|Query_5938:5454-5524 | hRSV/A/Cote_d'Ivoire/IPCI-014/2019\|EPI_ISL_5522630\|2019-08-27 |
| >lcl\|Query_54490:5384-5454 | hRSV/A/England/195180535/2019\|EPI_ISL_1520405\|2019-12-17 |
| >lcl\|Query_54185:5394-5464 | hRSV/A/England/200181040/2019\|EPI_ISL_1520411\|2019-12-30 |
| >lcl\|Query_54144:5344-5414 | hRSV/A/England/200181047/2019\|EPI_ISL_11428301\|2019-12-30 |
| >lcl\|Query_6061:5384-5454 | hRSV/A/England/195180535/2019\|EPI_ISL_1520405\|2019-12-17 |
| >lcl\|Query_5756:5394-5464 | hRSV/A/England/200181040/2019\|EPI_ISL_1520411\|2019-12-30 |
| >lcl\|Query_5715:5344-5414 | hRSV/A/England/200181047/2019\|EPI_ISL_11428301\|2019-12-30 |
| >lcl\|Query_54486:5444-5515 | hRSV/A/England/195140828/2019\|EPI_ISL_1520401\|2019-12-16 |
| >lcl\|Query_54312:5477-5548 | hRSV/A/Cote_d'Ivoire/IPCI-008/2019\|EPI_ISL_2543820\|2019-08-14 |
| >lcl\|Query_54285:5441-5512 | hRSV/A/England/195020395/2019\|EPI_ISL_1834155\|2019-12-06 |
| >lcl\|Query_54485:5430-5501 | hRSV/A/England/195120238/2019\|EPI_ISL_1520400\|2019-12-16 |
| >lcl\|Query_54286:5460-5531 | hRSV/A/England/195040643/2019\|EPI_ISL_1834158\|2019-12-09 |
| >lcl\|Query_54217:5469-5540 | hRSV/A/England/194540440/2019\|EPI_ISL_1647385\|2019-11-04 |
| >lcl\|Query_54340:5436-5507 | hRSV/A/Russia/Novosibirsk-138Hp/2019\|EPI_ISL_2582836\|2019-12-06 |
| >lcl\|Query_54256:5484-5555 | hRSV/A/Australia/VIC-RCH139/2019\|EPI_ISL_1834126\|2019-08-04 |
| >lcl\|Query_54224:5476-5547 | hRSV/A/England/195180359/2019\|EPI_ISL_1647392\|2019-12-17 |
| >lcl\|Query_54208:5488-5559 | hRSV/A/England/RE19003257/2019\|EPI_ISL_1520434\|2019-12-16 |
| >lcl\|Query_54339:5476-5547 | hRSV/A/Thailand/Ayuttaya-TNIC015/2020\|EPI_ISL_2543847\|2020-11-10 |
| >lcl\|Query_54337:5476-5547 | hRSV/A/Thailand/Ayuttaya-TNIC006/2020\|EPI_ISL_2543845\|2020-11-04 |
| >lcl\|Query_54338:5476-5547 | hRSV/A/Thailand/Ayuttaya-TNIC013/2020\|EPI_ISL_2543846\|2020-11-05 |
| >lcl\|Query_54266:5483-5554 | hRSV/A/Australia/VIC-RCH160/2019\|EPI_ISL_1834136\|2019-08-18 |
| >lcl\|Query_54220:5453-5524 | hRSV/A/England/194660604/2019\|EPI_ISL_1647388\|2019-11-12 |
| >lcl\|Query_54452:5503-5574 | hRSV/A/Philippines/99072/2019\|EPI_ISL_12970415\|2019-10-08 |
| >lcl\|Query_54442:5522-5593 | hRSV/A/Philippines/99058/2019\|EPI_ISL_12970401\|2019-03-04 |
| >lcl\|Query_54219:5473-5544 | hRSV/A/England/194660588/2019\|EPI_ISL_1647387\|2019-11-13 |
| >lcl\|Query_54491:5463-5534 | hRSV/A/England/195200142/2019\|EPI_ISL_1520406\|2019-12-18 |
| >lcl\|Query_54251:5482-5553 | hRSV/A/Australia/VIC-RCH132/2017\|EPI_ISL_1834120\|2017-09-08 |
| >lcl\|Query_54238:5483-5554 | hRSV/A/Australia/VIC-RCH095/2018\|EPI_ISL_1834106\|2018-06-17 |
| >lcl\|Query_6057:5444-5515 | hRSV/A/England/195140828/2019\|EPI_ISL_1520401\|2019-12-16 |
| >lcl\|Query_5883:5477-5548 | hRSV/A/Cote_d'Ivoire/IPCI-008/2019\|EPI_ISL_2543820\|2019-08-14 |
| >lcl\|Query_5856:5441-5512 | hRSV/A/England/195020395/2019\|EPI_ISL_1834155\|2019-12-06 |
| >lcl\|Query_6056:5430-5501 | hRSV/A/England/195120238/2019\|EPI_ISL_1520400\|2019-12-16 |
| >lcl\|Query_5857:5460-5531 | hRSV/A/England/195040643/2019\|EPI_ISL_1834158\|2019-12-09 |
| >lcl\|Query_5788:5469-5540 | hRSV/A/England/194540440/2019\|EPI_ISL_1647385\|2019-11-04 |
| >lcl\|Query_5911:5436-5507 | hRSV/A/Russia/Novosibirsk-138Hp/2019\|EPI_ISL_2582836\|2019-12-06 |
| >lcl\|Query_5827:5484-5555 | hRSV/A/Australia/VIC-RCH139/2019\|EPI_ISL_1834126\|2019-08-04 |
| >lcl\|Query_5795:5476-5547 | hRSV/A/England/195180359/2019\|EPI_ISL_1647392\|2019-12-17 |
| >lcl\|Query_5779:5488-5559 | hRSV/A/England/RE19003257/2019\|EPI_ISL_1520434\|2019-12-16 |
| >lcl\|Query_5910:5476-5547 | hRSV/A/Thailand/Ayuttaya-TNIC015/2020\|EPI_ISL_2543847\|2020-11-10 |
| >lcl\|Query_5908:5476-5547 | hRSV/A/Thailand/Ayuttaya-TNIC006/2020\|EPI_ISL_2543845\|2020-11-04 |
| >lcl\|Query_5909:5476-5547 | hRSV/A/Thailand/Ayuttaya-TNIC013/2020\|EPI_ISL_2543846\|2020-11-05 |
| >lcl\|Query_5837:5483-5554 | hRSV/A/Australia/VIC-RCH160/2019\|EPI_ISL_1834136\|2019-08-18 |
| >lcl\|Query_5791:5453-5524 | hRSV/A/England/194660604/2019\|EPI_ISL_1647388\|2019-11-12 |
| >lcl\|Query_6023:5503-5574 | hRSV/A/Philippines/99072/2019\|EPI_ISL_12970415\|2019-10-08 |
| >lcl\|Query_6013:5522-5593 | hRSV/A/Philippines/99058/2019\|EPI_ISL_12970401\|2019-03-04 |
| >lcl\|Query_5790:5473-5544 | hRSV/A/England/194660588/2019\|EPI_ISL_1647387\|2019-11-13 |
| >lcl\|Query_6062:5463-5534 | hRSV/A/England/195200142/2019\|EPI_ISL_1520406\|2019-12-18 |
| >lcl\|Query_5822:5482-5553 | hRSV/A/Australia/VIC-RCH132/2017\|EPI_ISL_1834120\|2017-09-08 |
| >lcl\|Query_5809:5483-5554 | hRSV/A/Australia/VIC-RCH095/2018\|EPI_ISL_1834106\|2018-06-17 |
| >lcl\|Query_54245:5484-5555 | hRSV/A/Australia/VIC-RCH113/2019\|EPI_ISL_1834114\|2019-07-08 |
| >lcl\|Query_5816:5484-5555 | hRSV/A/Australia/VIC-RCH113/2019\|EPI_ISL_1834114\|2019-07-08 |
| >lcl\|Query_54278:5484-5555 | hRSV/A/Australia/VIC-RCH192/2018\|EPI_ISL_1834148\|2018-12-16 |
| >lcl\|Query_54260:5494-5565 | hRSV/A/Australia/VIC-RCH144/2017\|EPI_ISL_1834130\|2017-12-31 |
| >lcl\|Query_5849:5484-5555 | hRSV/A/Australia/VIC-RCH192/2018\|EPI_ISL_1834148\|2018-12-16 |
| >lcl\|Query_5831:5494-5565 | hRSV/A/Australia/VIC-RCH144/2017\|EPI_ISL_1834130\|2017-12-31 |
| >lcl\|Query_54335:5478-5549 | hRSV/A/Mongolia/NCCD029/2019\|EPI_ISL_2543843\|2019-03-18 |
| >lcl\|Query_54334:5479-5550 | hRSV/A/Mongolia/NCCD028/2019\|EPI_ISL_2543842\|2019-03-11 |
| >lcl\|Query_54330:5478-5549 | hRSV/A/Mongolia/NCCD017/2019\|EPI_ISL_2543838\|2019-02-02 |
| >lcl\|Query_54329:5475-5546 | hRSV/A/Mongolia/NCCD014/2019\|EPI_ISL_2543837\|2019-01-30 |
| >lcl\|Query_54328:5475-5546 | hRSV/A/Mongolia/NCCD011/2019\|EPI_ISL_2543836\|2019-01-28 |
| >lcl\|Query_54327:5475-5546 | hRSV/A/Mongolia/NCCD010/2019\|EPI_ISL_2543835\|2019-01-21 |
| >lcl\|Query_54326:5475-5546 | hRSV/A/Mongolia/NCCD009/2019\|EPI_ISL_2543834\|2019-01-18 |
| >lcl\|Query_54323:5475-5546 | hRSV/A/Mongolia/NCCD007/2019\|EPI_ISL_2543831\|2019-01-15 |
| >lcl\|Query_5906:5478-5549 | hRSV/A/Mongolia/NCCD029/2019\|EPI_ISL_2543843\|2019-03-18 |
| >lcl\|Query_5905:5479-5550 | hRSV/A/Mongolia/NCCD028/2019\|EPI_ISL_2543842\|2019-03-11 |
| >lcl\|Query_5901:5478-5549 | hRSV/A/Mongolia/NCCD017/2019\|EPI_ISL_2543838\|2019-02-02 |
| >lcl\|Query_5900:5475-5546 | hRSV/A/Mongolia/NCCD014/2019\|EPI_ISL_2543837\|2019-01-30 |
| >lcl\|Query_5899:5475-5546 | hRSV/A/Mongolia/NCCD011/2019\|EPI_ISL_2543836\|2019-01-28 |
| >lcl\|Query_5898:5475-5546 | hRSV/A/Mongolia/NCCD010/2019\|EPI_ISL_2543835\|2019-01-21 |
| >lcl\|Query_5897:5475-5546 | hRSV/A/Mongolia/NCCD009/2019\|EPI_ISL_2543834\|2019-01-18 |
| >lcl\|Query_5894:5475-5546 | hRSV/A/Mongolia/NCCD007/2019\|EPI_ISL_2543831\|2019-01-15 |
| >lcl\|Query_54316:5481-5552 | hRSV/A/Cote_d'Ivoire/IPCI-008/2017\|EPI_ISL_2543824\|2017-08-20 |
| >lcl\|Query_5887:5481-5552 | hRSV/A/Cote_d'Ivoire/IPCI-008/2017\|EPI_ISL_2543824\|2017-08-20 |
| >lcl\|Query_54223:5457-5528 | hRSV/A/England/195080598/2019\|EPI_ISL_1647391\|2019-12-09 |
| >lcl\|Query_54507:5487-5558 | hRSV/A/England/RE20000021/2020\|EPI_ISL_1647411\|2020-01-05 |
| >lcl\|Query_54212:5467-5538 | hRSV/A/England/RE20000093/2020\|EPI_ISL_1520438\|2020-01-28 |
| >lcl\|Query_54284:5447-5518 | hRSV/A/England/194980363/2019\|EPI_ISL_1834154\|2019-12-05 |
| >lcl\|Query_54282:5454-5525 | hRSV/A/England/194780460/2019\|EPI_ISL_1834152\|2019-11-19 |
| >lcl\|Query_54218:5480-5551 | hRSV/A/England/194600343/2019\|EPI_ISL_1647386\|2019-11-08 |
| >lcl\|Query_54209:5445-5516 | hRSV/A/England/RE19003299/2019\|EPI_ISL_1520435\|2019-12-18 |
| >lcl\|Query_54311:5479-5550 | hRSV/A/Cote_d'Ivoire/IPCI-043/2017\|EPI_ISL_2543819\|2017-10-20 |
| >lcl\|Query_54310:5478-5549 | hRSV/A/Cote_d'Ivoire/IPCI-017/2017\|EPI_ISL_2543818\|2017-09-05 |
| >lcl\|Query_54291:5452-5523 | hRSV/A/England/RE20000092/2020\|EPI_ISL_1834173\|2020-01-28 |
| >lcl\|Query_54289:5455-5526 | hRSV/A/England/RE20000013/2019\|EPI_ISL_1834171\|2019-12-31 |
| >lcl\|Query_54213:5473-5544 | hRSV/A/England/RE20000094/2020\|EPI_ISL_1520439\|2020-01-28 |
| >lcl\|Query_54380:5487-5558 | hRSV/A/Argentina/BA-HNRG-304/2017\|EPI_ISL_1074253\|2017-04-17 |
| >lcl\|Query_54381:5492-5563 | hRSV/A/Argentina/BA-HNRG-350/2017\|EPI_ISL_1074255\|2017-06-12 |
| >lcl\|Query_54307:5481-5552 | hRSV/A/Cote_d'Ivoire/IPCI-022/2017\|EPI_ISL_2543815\|2017-09-04 |
| >lcl\|Query_54135:5487-5558 | hRSV/A/Argentina/BA-HNRG-336/2017\|EPI_ISL_1074160\|2017-06-01 |
| >lcl\|Query_54131:5488-5559 | hRSV/A/Argentina/BA-HNRG-343/2017\|EPI_ISL_1074156\|2017-06-07 |
| >lcl\|Query_54120:5487-5558 | hRSV/A/Argentina/BA-HNRG-359/2017\|EPI_ISL_1074106\|2017-06-19 |
| >lcl\|Query_54139:5494-5565 | hRSV/A/Argentina/BA-HNRG-326/2017\|EPI_ISL_1074171\|2017-05-26 |
| >lcl\|Query_54132:5489-5560 | hRSV/A/Argentina/BA-HNRG-372/2017\|EPI_ISL_1074157\|2017-06-27 |
| >lcl\|Query_54383:5488-5559 | hRSV/A/Argentina/BA-HNRG-370/2017\|EPI_ISL_1074257\|2017-06-26 |
| >lcl\|Query_54123:5485-5556 | hRSV/A/Argentina/BA-HNRG-353/2017\|EPI_ISL_1074120\|2017-06-14 |
| >lcl\|Query_54526:5483-5554 | hRSV/A/Mozambique/MAP-INS014/2018\|EPI_ISL_1647463\|2018-05-25 |
| >lcl\|Query_54267:5484-5555 | hRSV/A/Australia/VIC-RCH166/2018\|EPI_ISL_1834137\|2018-08-27 |
| >lcl\|Query_54236:5484-5555 | hRSV/A/Australia/VIC-RCH085/2017\|EPI_ISL_1834104\|2017-07-26 |
| >lcl\|Query_5794:5457-5528 | hRSV/A/England/195080598/2019\|EPI_ISL_1647391\|2019-12-09 |
| >lcl\|Query_6078:5487-5558 | hRSV/A/England/RE20000021/2020\|EPI_ISL_1647411\|2020-01-05 |
| >lcl\|Query_5783:5467-5538 | hRSV/A/England/RE20000093/2020\|EPI_ISL_1520438\|2020-01-28 |
| >lcl\|Query_5855:5447-5518 | hRSV/A/England/194980363/2019\|EPI_ISL_1834154\|2019-12-05 |
| >lcl\|Query_5853:5454-5525 | hRSV/A/England/194780460/2019\|EPI_ISL_1834152\|2019-11-19 |
| >lcl\|Query_5789:5480-5551 | hRSV/A/England/194600343/2019\|EPI_ISL_1647386\|2019-11-08 |
| >lcl\|Query_5780:5445-5516 | hRSV/A/England/RE19003299/2019\|EPI_ISL_1520435\|2019-12-18 |
| >lcl\|Query_5882:5479-5550 | hRSV/A/Cote_d'Ivoire/IPCI-043/2017\|EPI_ISL_2543819\|2017-10-20 |
| >lcl\|Query_5881:5478-5549 | hRSV/A/Cote_d'Ivoire/IPCI-017/2017\|EPI_ISL_2543818\|2017-09-05 |
| >lcl\|Query_5862:5452-5523 | hRSV/A/England/RE20000092/2020\|EPI_ISL_1834173\|2020-01-28 |
| >lcl\|Query_5860:5455-5526 | hRSV/A/England/RE20000013/2019\|EPI_ISL_1834171\|2019-12-31 |
| >lcl\|Query_5784:5473-5544 | hRSV/A/England/RE20000094/2020\|EPI_ISL_1520439\|2020-01-28 |
| >lcl\|Query_5951:5487-5558 | hRSV/A/Argentina/BA-HNRG-304/2017\|EPI_ISL_1074253\|2017-04-17 |
| >lcl\|Query_5952:5492-5563 | hRSV/A/Argentina/BA-HNRG-350/2017\|EPI_ISL_1074255\|2017-06-12 |
| >lcl\|Query_5878:5481-5552 | hRSV/A/Cote_d'Ivoire/IPCI-022/2017\|EPI_ISL_2543815\|2017-09-04 |
| >lcl\|Query_5706:5487-5558 | hRSV/A/Argentina/BA-HNRG-336/2017\|EPI_ISL_1074160\|2017-06-01 |
| >lcl\|Query_5702:5488-5559 | hRSV/A/Argentina/BA-HNRG-343/2017\|EPI_ISL_1074156\|2017-06-07 |
| >lcl\|Query_5691:5487-5558 | hRSV/A/Argentina/BA-HNRG-359/2017\|EPI_ISL_1074106\|2017-06-19 |
| >lcl\|Query_5710:5494-5565 | hRSV/A/Argentina/BA-HNRG-326/2017\|EPI_ISL_1074171\|2017-05-26 |
| >lcl\|Query_5703:5489-5560 | hRSV/A/Argentina/BA-HNRG-372/2017\|EPI_ISL_1074157\|2017-06-27 |
| >lcl\|Query_5954:5488-5559 | hRSV/A/Argentina/BA-HNRG-370/2017\|EPI_ISL_1074257\|2017-06-26 |
| >lcl\|Query_5694:5485-5556 | hRSV/A/Argentina/BA-HNRG-353/2017\|EPI_ISL_1074120\|2017-06-14 |
| >lcl\|Query_6097:5483-5554 | hRSV/A/Mozambique/MAP-INS014/2018\|EPI_ISL_1647463\|2018-05-25 |
| >lcl\|Query_5838:5484-5555 | hRSV/A/Australia/VIC-RCH166/2018\|EPI_ISL_1834137\|2018-08-27 |
| >lcl\|Query_5807:5484-5555 | hRSV/A/Australia/VIC-RCH085/2017\|EPI_ISL_1834104\|2017-07-26 |
| >lcl\|Query_54384:5496-5567 | hRSV/A/Argentina/BA-HNRG-314/2017\|EPI_ISL_1074259\|2017-05-12 |
| >lcl\|Query_54141:5494-5565 | hRSV/A/Argentina/BA-HNRG-388/2017\|EPI_ISL_1074173\|2017-07-17 |
| >lcl\|Query_54133:5490-5561 | hRSV/A/Argentina/BA-HNRG-315/2017\|EPI_ISL_1074158\|2017-05-15 |
| >lcl\|Query_54379:5490-5561 | hRSV/A/Argentina/BA-HNRG-369/2017\|EPI_ISL_1074246\|2017-06-26 |
| >lcl\|Query_54143:5488-5559 | hRSV/A/Argentina/BA-HNRG-380/2017\|EPI_ISL_1074195\|2017-07-04 |
| >lcl\|Query_54513:5483-5554 | hRSV/A/England/RE20000101/2020\|EPI_ISL_1647417\|2020-02-07 |
| >lcl\|Query_5955:5496-5567 | hRSV/A/Argentina/BA-HNRG-314/2017\|EPI_ISL_1074259\|2017-05-12 |
| >lcl\|Query_5712:5494-5565 | hRSV/A/Argentina/BA-HNRG-388/2017\|EPI_ISL_1074173\|2017-07-17 |
| >lcl\|Query_5704:5490-5561 | hRSV/A/Argentina/BA-HNRG-315/2017\|EPI_ISL_1074158\|2017-05-15 |
| >lcl\|Query_5950:5490-5561 | hRSV/A/Argentina/BA-HNRG-369/2017\|EPI_ISL_1074246\|2017-06-26 |
| >lcl\|Query_5714:5488-5559 | hRSV/A/Argentina/BA-HNRG-380/2017\|EPI_ISL_1074195\|2017-07-04 |
| >lcl\|Query_6084:5483-5554 | hRSV/A/England/RE20000101/2020\|EPI_ISL_1647417\|2020-02-07 |
| >lcl\|Query_54309:5481-5552 | hRSV/A/Cote_d'Ivoire/IPCI-013/2017\|EPI_ISL_2543817\|2017-09-02 |
| >lcl\|Query_5880:5481-5552 | hRSV/A/Cote_d'Ivoire/IPCI-013/2017\|EPI_ISL_2543817\|2017-09-02 |
| >lcl\|Query_54382:5489-5560 | hRSV/A/Argentina/BA-HNRG-338/2017\|EPI_ISL_1074256\|2017-06-02 |
| >lcl\|Query_54138:5489-5560 | hRSV/A/Argentina/BA-HNRG-323/2017\|EPI_ISL_1074170\|2017-05-22 |
| >lcl\|Query_54128:5494-5565 | hRSV/A/Argentina/BA-HNRG-321/2017\|EPI_ISL_1074152\|2017-05-22 |
| >lcl\|Query_54126:5489-5560 | hRSV/A/Argentina/BA-HNRG-330/2017\|EPI_ISL_1074137\|2017-05-29 |
| >lcl\|Query_5953:5489-5560 | hRSV/A/Argentina/BA-HNRG-338/2017\|EPI_ISL_1074256\|2017-06-02 |
| >lcl\|Query_5709:5489-5560 | hRSV/A/Argentina/BA-HNRG-323/2017\|EPI_ISL_1074170\|2017-05-22 |
| >lcl\|Query_5699:5494-5565 | hRSV/A/Argentina/BA-HNRG-321/2017\|EPI_ISL_1074152\|2017-05-22 |
| >lcl\|Query_5697:5489-5560 | hRSV/A/Argentina/BA-HNRG-330/2017\|EPI_ISL_1074137\|2017-05-29 |
| >lcl\|Query_54315:5477-5548 | hRSV/A/Cote_d'Ivoire/IPCI-026/2019\|EPI_ISL_2543823\|2019-10-19 |
| >lcl\|Query_54304:5478-5549 | hRSV/A/Cote_d'Ivoire/IPCI-023/2019\|EPI_ISL_2543812\|2019-10-21 |
| >lcl\|Query_54313:5490-5561 | hRSV/A/Cote_d'Ivoire/IPCI-013/2019\|EPI_ISL_2543821\|2019-08-27 |
| >lcl\|Query_5886:5477-5548 | hRSV/A/Cote_d'Ivoire/IPCI-026/2019\|EPI_ISL_2543823\|2019-10-19 |
| >lcl\|Query_5875:5478-5549 | hRSV/A/Cote_d'Ivoire/IPCI-023/2019\|EPI_ISL_2543812\|2019-10-21 |
| >lcl\|Query_5884:5490-5561 | hRSV/A/Cote_d'Ivoire/IPCI-013/2019\|EPI_ISL_2543821\|2019-08-27 |
| >lcl\|Query_54314:5481-5552 | hRSV/A/Cote_d'Ivoire/IPCI-011/2019\|EPI_ISL_2543822\|2019-08-19 |
| >lcl\|Query_5885:5481-5552 | hRSV/A/Cote_d'Ivoire/IPCI-011/2019\|EPI_ISL_2543822\|2019-08-19 |
| >lcl\|Query_54496:5415-5486 | hRSV/A/England/RE19003147/2019\|EPI_ISL_1647400\|2019-12-02 |
| >lcl\|Query_54201:5408-5479 | hRSV/A/England/RE19003153/2019\|EPI_ISL_1520427\|2019-11-22 |
| >lcl\|Query_54188:5414-5485 | hRSV/A/England/RE19003123/2019\|EPI_ISL_1520414\|2019-11-17 |
| >lcl\|Query_6067:5415-5486 | hRSV/A/England/RE19003147/2019\|EPI_ISL_1647400\|2019-12-02 |
| >lcl\|Query_5772:5408-5479 | hRSV/A/England/RE19003153/2019\|EPI_ISL_1520427\|2019-11-22 |
| >lcl\|Query_5759:5414-5485 | hRSV/A/England/RE19003123/2019\|EPI_ISL_1520414\|2019-11-17 |
| >lcl\|Query_54455:5431-5502 | hRSV/A/Philippines/99075/2019\|EPI_ISL_12970418\|2019-11-04 |
| >lcl\|Query_54450:5431-5502 | hRSV/A/Philippines/99070/2019\|EPI_ISL_12970413\|2019-09-23 |
| >lcl\|Query_54449:5431-5502 | hRSV/A/Philippines/99069/2019\|EPI_ISL_12970412\|2019-09-09 |
| >lcl\|Query_6026:5431-5502 | hRSV/A/Philippines/99075/2019\|EPI_ISL_12970418\|2019-11-04 |
| >lcl\|Query_6021:5431-5502 | hRSV/A/Philippines/99070/2019\|EPI_ISL_12970413\|2019-09-23 |
| >lcl\|Query_6020:5431-5502 | hRSV/A/Philippines/99069/2019\|EPI_ISL_12970412\|2019-09-09 |
| >lcl\|Query_54523:5486-5557 | hRSV/A/Mozambique/MAP-INS011/2018\|EPI_ISL_1647460\|2018-05-10 |
| >lcl\|Query_54522:5483-5554 | hRSV/A/Mozambique/MAP-INS009/2018\|EPI_ISL_1647459\|2018-04-17 |
| >lcl\|Query_54520:5486-5557 | hRSV/A/Mozambique/MAP-INS006/2018\|EPI_ISL_1647457\|2018-04-03 |
| >lcl\|Query_54524:5484-5555 | hRSV/A/Mozambique/MAP-INS012/2018\|EPI_ISL_1647461\|2018-05-11 |
| >lcl\|Query_6094:5486-5557 | hRSV/A/Mozambique/MAP-INS011/2018\|EPI_ISL_1647460\|2018-05-10 |
| >lcl\|Query_6093:5483-5554 | hRSV/A/Mozambique/MAP-INS009/2018\|EPI_ISL_1647459\|2018-04-17 |
| >lcl\|Query_6091:5486-5557 | hRSV/A/Mozambique/MAP-INS006/2018\|EPI_ISL_1647457\|2018-04-03 |
| >lcl\|Query_6095:5484-5555 | hRSV/A/Mozambique/MAP-INS012/2018\|EPI_ISL_1647461\|2018-05-11 |
| >lcl\|Query_54279:5487-5558 | hRSV/A/Australia/VIC-RCH202/2019\|EPI_ISL_1834149\|2019-12-09 |
| >lcl\|Query_5850:5487-5558 | hRSV/A/Australia/VIC-RCH202/2019\|EPI_ISL_1834149\|2019-12-09 |
| >lcl\|Query_54444:5503-5574 | hRSV/A/Philippines/99062/2019\|EPI_ISL_12970405\|2019-08-05 |
| >lcl\|Query_6015:5503-5574 | hRSV/A/Philippines/99062/2019\|EPI_ISL_12970405\|2019-08-05 |
| >lcl\|Query_54118:5492-5563 | hRSV/A/Argentina/BA-HNRG-307/2017\|EPI_ISL_1074100\|2017-05-02 |
| >lcl\|Query_5689:5492-5563 | hRSV/A/Argentina/BA-HNRG-307/2017\|EPI_ISL_1074100\|2017-05-02 |
| >lcl\|Query_54476:5459-5530 | hRSV/A/Argentina/BA-HNRG-439/2019\|EPI_ISL_15067685\|2019-07-25 |
| >lcl\|Query_54175:5459-5530 | hRSV/A/Argentina/HNRG-439/2019\|EPI_ISL_15055320\|2019-07-25 |
| >lcl\|Query_6047:5459-5530 | hRSV/A/Argentina/BA-HNRG-439/2019\|EPI_ISL_15067685\|2019-07-25 |
| >lcl\|Query_5746:5459-5530 | hRSV/A/Argentina/HNRG-439/2019\|EPI_ISL_15055320\|2019-07-25 |
| >lcl\|Query_54457:5502-5574 | hRSV/A/Philippines/99077/2019\|EPI_ISL_12970420\|2019-11-20 |
| >lcl\|Query_6028:5502-5574 | hRSV/A/Philippines/99077/2019\|EPI_ISL_12970420\|2019-11-20 |
| >lcl\|Query_54317:5482-5552 | hRSV/A/Cote_d'Ivoire/IPCI-017/2019\|EPI_ISL_2543825\|2019-09-05 |
| >lcl\|Query_5888:5482-5552 | hRSV/A/Cote_d'Ivoire/IPCI-017/2019\|EPI_ISL_2543825\|2019-09-05 |
| >lcl\|Query_54268:5486-5555 | hRSV/A/Australia/VIC-RCH168/2018\|EPI_ISL_1834138\|2018-08-26 |
| >lcl\|Query_5839:5486-5555 | hRSV/A/Australia/VIC-RCH168/2018\|EPI_ISL_1834138\|2018-08-26 |
| >lcl\|Query_54263:5412-5481 | hRSV/A/Australia/VIC-RCH150/2018\|EPI_ISL_1834133\|2018-07-28 |
| >lcl\|Query_54249:5412-5481 | hRSV/A/Australia/VIC-RCH125/2018\|EPI_ISL_1834118\|2018-07-08 |
| >lcl\|Query_54281:5392-5461 | hRSV/A/England/194680415/2019\|EPI_ISL_1834151\|2019-11-14 |
| >lcl\|Query_54191:5405-5474 | hRSV/A/England/RE19003129/2019\|EPI_ISL_1520417\|2019-12-01 |
| >lcl\|Query_5834:5412-5481 | hRSV/A/Australia/VIC-RCH150/2018\|EPI_ISL_1834133\|2018-07-28 |
| >lcl\|Query_5820:5412-5481 | hRSV/A/Australia/VIC-RCH125/2018\|EPI_ISL_1834118\|2018-07-08 |
| >lcl\|Query_5852:5392-5461 | hRSV/A/England/194680415/2019\|EPI_ISL_1834151\|2019-11-14 |
| >lcl\|Query_5762:5405-5474 | hRSV/A/England/RE19003129/2019\|EPI_ISL_1520417\|2019-12-01 |
| >lcl\|Query_54288:5436-5508 | hRSV/A/England/RE19003298/2019\|EPI_ISL_1834170\|2019-12-18 |
| >lcl\|Query_5859:5436-5508 | hRSV/A/England/RE19003298/2019\|EPI_ISL_1834170\|2019-12-18 |
| >lcl\|Query_54280:5378-5446 | hRSV/A/England/194580267/2019\|EPI_ISL_1834150\|2019-11-06 |
| >lcl\|Query_5851:5378-5446 | hRSV/A/England/194580267/2019\|EPI_ISL_1834150\|2019-11-06 |
| >lcl\|Query_54313:5418-5487 | hRSV/A/Cote_d'Ivoire/IPCI-013/2019\|EPI_ISL_2543821\|2019-08-27 |
| >lcl\|Query_5884:5418-5487 | hRSV/A/Cote_d'Ivoire/IPCI-013/2019\|EPI_ISL_2543821\|2019-08-27 |
| >lcl\|Query_54439:5385-5452 | hRSV/A/South_Africa/NICD-R04470/2021\|EPI_ISL_12529646\|2021-03-30 |
| >lcl\|Query_54438:5403-5470 | hRSV/A/South_Africa/NICD-R04469/2021\|EPI_ISL_12529645\|2021-03-30 |
| >lcl\|Query_54433:5385-5452 | hRSV/A/South_Africa/NICD-R03866/2021\|EPI_ISL_12529640\|2021-03-15 |
| >lcl\|Query_54432:5391-5458 | hRSV/A/South_Africa/NICD-R03864/2021\|EPI_ISL_12529639\|2021-03-15 |
| >lcl\|Query_54440:5372-5439 | hRSV/A/South_Africa/NICD-R04476/2021\|EPI_ISL_12529647\|2021-03-30 |
| >lcl\|Query_54437:5391-5458 | hRSV/A/South_Africa/NICD-R04354/2021\|EPI_ISL_12529644\|2021-03-31 |
| >lcl\|Query_54436:5394-5461 | hRSV/A/South_Africa/NICD-R04181/2021\|EPI_ISL_12529643\|2021-03-25 |
| >lcl\|Query_54434:5396-5463 | hRSV/A/South_Africa/NICD-R03876/2021\|EPI_ISL_12529641\|2021-03-17 |
| >lcl\|Query_54428:5401-5468 | hRSV/A/South_Africa/NICD-R03651/2021\|EPI_ISL_12529634\|2021-03-09 |
| >lcl\|Query_6010:5385-5452 | hRSV/A/South_Africa/NICD-R04470/2021\|EPI_ISL_12529646\|2021-03-30 |
| >lcl\|Query_6009:5403-5470 | hRSV/A/South_Africa/NICD-R04469/2021\|EPI_ISL_12529645\|2021-03-30 |
| >lcl\|Query_6004:5385-5452 | hRSV/A/South_Africa/NICD-R03866/2021\|EPI_ISL_12529640\|2021-03-15 |
| >lcl\|Query_6003:5391-5458 | hRSV/A/South_Africa/NICD-R03864/2021\|EPI_ISL_12529639\|2021-03-15 |
| >lcl\|Query_6011:5372-5439 | hRSV/A/South_Africa/NICD-R04476/2021\|EPI_ISL_12529647\|2021-03-30 |
| >lcl\|Query_6008:5391-5458 | hRSV/A/South_Africa/NICD-R04354/2021\|EPI_ISL_12529644\|2021-03-31 |
| >lcl\|Query_6007:5394-5461 | hRSV/A/South_Africa/NICD-R04181/2021\|EPI_ISL_12529643\|2021-03-25 |
| >lcl\|Query_6005:5396-5463 | hRSV/A/South_Africa/NICD-R03876/2021\|EPI_ISL_12529641\|2021-03-17 |
| >lcl\|Query_5999:5401-5468 | hRSV/A/South_Africa/NICD-R03651/2021\|EPI_ISL_12529634\|2021-03-09 |
| >lcl\|Query_54435:5405-5472 | hRSV/A/South_Africa/NICD-R04004/2021\|EPI_ISL_12529642\|2021-03-19 |
| >lcl\|Query_6006:5405-5472 | hRSV/A/South_Africa/NICD-R04004/2021\|EPI_ISL_12529642\|2021-03-19 |
| >lcl\|Query_54431:5385-5452 | hRSV/A/South_Africa/NICD-R03672/2021\|EPI_ISL_12529638\|2021-03-12 |
| >lcl\|Query_6002:5385-5452 | hRSV/A/South_Africa/NICD-R03672/2021\|EPI_ISL_12529638\|2021-03-12 |
| >lcl\|Query_54429:5402-5469 | hRSV/A/South_Africa/NICD-R03652/2021\|EPI_ISL_12529635\|2021-03-09 |
| >lcl\|Query_6000:5402-5469 | hRSV/A/South_Africa/NICD-R03652/2021\|EPI_ISL_12529635\|2021-03-09 |
| >lcl\|Query_54240:5483-5551 | hRSV/A/Australia/VIC-RCH101/2018\|EPI_ISL_1834108\|2018-06-25 |
| >lcl\|Query_54269:5483-5551 | hRSV/A/Australia/VIC-RCH169/2018\|EPI_ISL_1834139\|2018-08-26 |
| >lcl\|Query_5811:5483-5551 | hRSV/A/Australia/VIC-RCH101/2018\|EPI_ISL_1834108\|2018-06-25 |
| >lcl\|Query_5840:5483-5551 | hRSV/A/Australia/VIC-RCH169/2018\|EPI_ISL_1834139\|2018-08-26 |
| >lcl\|Query_54162:5400-5527 | hRSV/A/Spain/CHUVI-19464401/2021\|EPI_ISL_14084089\|2021-12-13 |
| >lcl\|Query_5733:5400-5527 | hRSV/A/Spain/CHUVI-19464401/2021\|EPI_ISL_14084089\|2021-12-13 |
| >lcl\|Query_54495:5467-5596 | hRSV/A/Belgium/0000002/2022\|EPI_ISL_15421344\|2022-03-23 |
| >lcl\|Query_6066:5467-5596 | hRSV/A/Belgium/0000002/2022\|EPI_ISL_15421344\|2022-03-23 |
| >lcl\|Query_54164:5410-5537 | hRSV/A/Spain/CHUVI-19478508/2021\|EPI_ISL_14084091\|2021-12-23 |
| >lcl\|Query_5735:5410-5537 | hRSV/A/Spain/CHUVI-19478508/2021\|EPI_ISL_14084091\|2021-12-23 |
| >lcl\|Query_54163:5348-5475 | hRSV/A/Spain/CHUVI-19475176/2021\|EPI_ISL_14084090\|2021-12-21 |
| >lcl\|Query_5734:5348-5475 | hRSV/A/Spain/CHUVI-19475176/2021\|EPI_ISL_14084090\|2021-12-21 |
| >lcl\|Query_54517:5487-5558 | hRSV/A/England/RS20000581/2020\|EPI_ISL_1647421\|2020-02-04 |
| >lcl\|Query_6088:5487-5558 | hRSV/A/England/RS20000581/2020\|EPI_ISL_1647421\|2020-02-04 |
| >lcl\|Query_54457:5431-5502 | hRSV/A/Philippines/99077/2019\|EPI_ISL_12970420\|2019-11-20 |
| >lcl\|Query_6028:5431-5502 | hRSV/A/Philippines/99077/2019\|EPI_ISL_12970420\|2019-11-20 |
| >lcl\|Query_54320:5466-5537 | hRSV/A/Mongolia/NCCD006/2018\|EPI_ISL_2543828\|2018-07-10 |
| >lcl\|Query_54336:5469-5540 | hRSV/A/Mongolia/NCCD039/2018\|EPI_ISL_2543844\|2018-03-07 |
| >lcl\|Query_54333:5469-5540 | hRSV/A/Mongolia/NCCD021/2018\|EPI_ISL_2543841\|2018-01-31 |
| >lcl\|Query_54324:5466-5537 | hRSV/A/Mongolia/NCCD008/2018\|EPI_ISL_2543832\|2018-01-10 |
| >lcl\|Query_54322:5469-5540 | hRSV/A/Mongolia/NCCD007/2018\|EPI_ISL_2543830\|2018-01-10 |
| >lcl\|Query_5891:5466-5537 | hRSV/A/Mongolia/NCCD006/2018\|EPI_ISL_2543828\|2018-07-10 |
| >lcl\|Query_5907:5469-5540 | hRSV/A/Mongolia/NCCD039/2018\|EPI_ISL_2543844\|2018-03-07 |
| >lcl\|Query_5904:5469-5540 | hRSV/A/Mongolia/NCCD021/2018\|EPI_ISL_2543841\|2018-01-31 |
| >lcl\|Query_5895:5466-5537 | hRSV/A/Mongolia/NCCD008/2018\|EPI_ISL_2543832\|2018-01-10 |
| >lcl\|Query_5893:5469-5540 | hRSV/A/Mongolia/NCCD007/2018\|EPI_ISL_2543830\|2018-01-10 |
| >lcl\|Query_54377:5494-5565 | hRSV/A/Argentina/BA-HNRG-355/2017\|EPI_ISL_1074234\|2017-06-16 |
| >lcl\|Query_54137:5486-5557 | hRSV/A/Argentina/BA-HNRG-320/2017\|EPI_ISL_1074169\|2017-05-19 |
| >lcl\|Query_5948:5494-5565 | hRSV/A/Argentina/BA-HNRG-355/2017\|EPI_ISL_1074234\|2017-06-16 |
| >lcl\|Query_5708:5486-5557 | hRSV/A/Argentina/BA-HNRG-320/2017\|EPI_ISL_1074169\|2017-05-19 |
| >lcl\|Query_54270:5413-5483 | hRSV/A/Australia/VIC-RCH179/2018\|EPI_ISL_1834140\|2018-11-29 |
| >lcl\|Query_54422:5407-5477 | hRSV/A/Australia/VIC-RCH071/2021\|EPI_ISL_11817092\|2021-03-27 |
| >lcl\|Query_54419:5432-5502 | hRSV/A/Australia/QLD-RBWH278/2021\|EPI_ISL_11817078\|2021-04-01 |
| >lcl\|Query_54417:5434-5504 | hRSV/A/Australia/QLD-RBWH264/2021\|EPI_ISL_11817075\|2021-03-27 |
| >lcl\|Query_54416:5434-5504 | hRSV/A/Australia/QLD-RBWH244/2021\|EPI_ISL_11817072\|2021-03-18 |
| >lcl\|Query_54411:5433-5503 | hRSV/A/Australia/QLD-RBWH206/2021\|EPI_ISL_11817065\|2021-03-10 |
| >lcl\|Query_54404:5434-5504 | hRSV/A/Australia/QLD-RBWH075/2021\|EPI_ISL_11817046\|2021-02-09 |
| >lcl\|Query_54402:5434-5504 | hRSV/A/Australia/QLD-RBWH068/2021\|EPI_ISL_11817043\|2021-02-07 |
| >lcl\|Query_54392:5434-5504 | hRSV/A/Australia/QLD-RBWH026/2021\|EPI_ISL_11817026\|2021-01-22 |
| >lcl\|Query_54391:5434-5504 | hRSV/A/Australia/QLD-RBWH022/2021\|EPI_ISL_11817025\|2021-01-18 |
| >lcl\|Query_54389:5434-5504 | hRSV/A/Australia/QLD-RBWH020/2021\|EPI_ISL_11817023\|2021-01-17 |
| >lcl\|Query_54298:5407-5477 | hRSV/A/Australia/VIC-RCH050/2021\|EPI_ISL_2543806\|2021-03-08 |
| >lcl\|Query_54418:5434-5504 | hRSV/A/Australia/QLD-RBWH273/2021\|EPI_ISL_11817076\|2021-03-30 |
| >lcl\|Query_54414:5434-5504 | hRSV/A/Australia/QLD-RBWH229/2021\|EPI_ISL_11817070\|2021-03-15 |
| >lcl\|Query_54413:5434-5504 | hRSV/A/Australia/QLD-RBWH227/2021\|EPI_ISL_11817069\|2021-03-15 |
| >lcl\|Query_54409:5434-5504 | hRSV/A/Australia/QLD-RBWH185/2021\|EPI_ISL_11817061\|2021-03-05 |
| >lcl\|Query_54407:5434-5504 | hRSV/A/Australia/QLD-RBWH130/2021\|EPI_ISL_11817053\|2021-02-18 |
| >lcl\|Query_54400:5434-5504 | hRSV/A/Australia/QLD-RBWH065/2021\|EPI_ISL_11817041\|2021-02-05 |
| >lcl\|Query_54399:5434-5504 | hRSV/A/Australia/QLD-RBWH060/2021\|EPI_ISL_11817039\|2021-02-04 |
| >lcl\|Query_54397:5434-5504 | hRSV/A/Australia/QLD-RBWH053/2021\|EPI_ISL_11817036\|2021-02-02 |
| >lcl\|Query_54388:5434-5504 | hRSV/A/Australia/QLD-RBWH015/2021\|EPI_ISL_11817021\|2021-01-13 |
| >lcl\|Query_54221:5404-5474 | hRSV/A/England/194840215/2019\|EPI_ISL_1647389\|2019-11-25 |
| >lcl\|Query_54149:5434-5504 | hRSV/A/Australia/QLD-RBWH287/2021\|EPI_ISL_11817083\|2021-04-06 |
| >lcl\|Query_5841:5413-5483 | hRSV/A/Australia/VIC-RCH179/2018\|EPI_ISL_1834140\|2018-11-29 |
| >lcl\|Query_5993:5407-5477 | hRSV/A/Australia/VIC-RCH071/2021\|EPI_ISL_11817092\|2021-03-27 |
| >lcl\|Query_5990:5432-5502 | hRSV/A/Australia/QLD-RBWH278/2021\|EPI_ISL_11817078\|2021-04-01 |
| >lcl\|Query_5988:5434-5504 | hRSV/A/Australia/QLD-RBWH264/2021\|EPI_ISL_11817075\|2021-03-27 |
| >lcl\|Query_5987:5434-5504 | hRSV/A/Australia/QLD-RBWH244/2021\|EPI_ISL_11817072\|2021-03-18 |
| >lcl\|Query_5982:5433-5503 | hRSV/A/Australia/QLD-RBWH206/2021\|EPI_ISL_11817065\|2021-03-10 |
| >lcl\|Query_5975:5434-5504 | hRSV/A/Australia/QLD-RBWH075/2021\|EPI_ISL_11817046\|2021-02-09 |
| >lcl\|Query_5973:5434-5504 | hRSV/A/Australia/QLD-RBWH068/2021\|EPI_ISL_11817043\|2021-02-07 |
| >lcl\|Query_5963:5434-5504 | hRSV/A/Australia/QLD-RBWH026/2021\|EPI_ISL_11817026\|2021-01-22 |
| >lcl\|Query_5962:5434-5504 | hRSV/A/Australia/QLD-RBWH022/2021\|EPI_ISL_11817025\|2021-01-18 |
| >lcl\|Query_5960:5434-5504 | hRSV/A/Australia/QLD-RBWH020/2021\|EPI_ISL_11817023\|2021-01-17 |
| >lcl\|Query_5869:5407-5477 | hRSV/A/Australia/VIC-RCH050/2021\|EPI_ISL_2543806\|2021-03-08 |
| >lcl\|Query_5989:5434-5504 | hRSV/A/Australia/QLD-RBWH273/2021\|EPI_ISL_11817076\|2021-03-30 |
| >lcl\|Query_5985:5434-5504 | hRSV/A/Australia/QLD-RBWH229/2021\|EPI_ISL_11817070\|2021-03-15 |
| >lcl\|Query_5984:5434-5504 | hRSV/A/Australia/QLD-RBWH227/2021\|EPI_ISL_11817069\|2021-03-15 |
| >lcl\|Query_5980:5434-5504 | hRSV/A/Australia/QLD-RBWH185/2021\|EPI_ISL_11817061\|2021-03-05 |
| >lcl\|Query_5978:5434-5504 | hRSV/A/Australia/QLD-RBWH130/2021\|EPI_ISL_11817053\|2021-02-18 |
| >lcl\|Query_5971:5434-5504 | hRSV/A/Australia/QLD-RBWH065/2021\|EPI_ISL_11817041\|2021-02-05 |
| >lcl\|Query_5970:5434-5504 | hRSV/A/Australia/QLD-RBWH060/2021\|EPI_ISL_11817039\|2021-02-04 |
| >lcl\|Query_5968:5434-5504 | hRSV/A/Australia/QLD-RBWH053/2021\|EPI_ISL_11817036\|2021-02-02 |
| >lcl\|Query_5959:5434-5504 | hRSV/A/Australia/QLD-RBWH015/2021\|EPI_ISL_11817021\|2021-01-13 |
| >lcl\|Query_5792:5404-5474 | hRSV/A/England/194840215/2019\|EPI_ISL_1647389\|2019-11-25 |
| >lcl\|Query_5720:5434-5504 | hRSV/A/Australia/QLD-RBWH287/2021\|EPI_ISL_11817083\|2021-04-06 |
| >lcl\|Query_54140:5416-5486 | hRSV/A/Argentina/BA-HNRG-332/2017\|EPI_ISL_1074172\|2017-05-30 |
| >lcl\|Query_5711:5416-5486 | hRSV/A/Argentina/BA-HNRG-332/2017\|EPI_ISL_1074172\|2017-05-30 |
| >lcl\|Query_54379:5418-5488 | hRSV/A/Argentina/BA-HNRG-369/2017\|EPI_ISL_1074246\|2017-06-26 |
| >lcl\|Query_54143:5416-5486 | hRSV/A/Argentina/BA-HNRG-380/2017\|EPI_ISL_1074195\|2017-07-04 |
| >lcl\|Query_5950:5418-5488 | hRSV/A/Argentina/BA-HNRG-369/2017\|EPI_ISL_1074246\|2017-06-26 |
| >lcl\|Query_5714:5416-5486 | hRSV/A/Argentina/BA-HNRG-380/2017\|EPI_ISL_1074195\|2017-07-04 |
| >lcl\|Query_54457:5301-5630 | hRSV/A/Philippines/99077/2019\|EPI_ISL_12970420\|2019-11-20 |
| >lcl\|Query_6028:5301-5630 | hRSV/A/Philippines/99077/2019\|EPI_ISL_12970420\|2019-11-20 |

**Table 8: Metdata on GISAID RSV B genome sequences used in this study**

| **Query** | **GISAIDID** |
| --- | --- |
| >lcl\|Query_554675:5123-5580 | hRSV/B/Argentina/BA-HNRG-446/2021\|EPI_ISL_15067718\|2021-07-13 |
| >lcl\|Query_554661:5123-5580 | hRSV/B/Argentina/HNRG-446/2021\|EPI_ISL_15055353\|2021-07-13 |
| >lcl\|Query_554680:5123-5580 | hRSV/B/Argentina/BA-HNRG-454/2021\|EPI_ISL_15067723\|2021-08-23 |
| >lcl\|Query_554674:5122-5579 | hRSV/B/Argentina/BA-HNRG-445/2021\|EPI_ISL_15067717\|2021-07-09 |
| >lcl\|Query_554673:5123-5580 | hRSV/B/Argentina/BA-HNRG-444/2021\|EPI_ISL_15067716\|2021-07-08 |
| >lcl\|Query_554671:5123-5580 | hRSV/B/Argentina/BA-HNRG-431/2021\|EPI_ISL_15067714\|2021-07-26 |
| >lcl\|Query_554666:5123-5580 | hRSV/B/Argentina/HNRG-454/2021\|EPI_ISL_15055358\|2021-08-23 |
| >lcl\|Query_554660:5122-5579 | hRSV/B/Argentina/HNRG-445/2021\|EPI_ISL_15055352\|2021-07-09 |
| >lcl\|Query_554659:5123-5580 | hRSV/B/Argentina/HNRG-444/2021\|EPI_ISL_15055351\|2021-07-08 |
| >lcl\|Query_554657:5123-5580 | hRSV/B/Argentina/HNRG-431/2021\|EPI_ISL_15055349\|2021-07-26 |
| >lcl\|Query_108000:5148-5605 | hRSV/B/Belgium/000003/2022\|EPI_ISL_15421405\|2022-03-28 |
| >lcl\|Query_107946:5141-5598 | hRSV/B/South_Africa/NICD-R05794/2022\|EPI_ISL_14769851\|2022-05-20 |
| >lcl\|Query_554679:5123-5580 | hRSV/B/Argentina/BA-HNRG-451/2021\|EPI_ISL_15067722\|2021-07-28 |
| >lcl\|Query_554676:5123-5580 | hRSV/B/Argentina/BA-HNRG-447/2021\|EPI_ISL_15067719\|2021-07-14 |
| >lcl\|Query_554665:5123-5580 | hRSV/B/Argentina/HNRG-451/2021\|EPI_ISL_15055357\|2021-07-28 |
| >lcl\|Query_554662:5123-5580 | hRSV/B/Argentina/HNRG-447/2021\|EPI_ISL_15055354\|2021-07-14 |
| >lcl\|Query_554678:5123-5580 | hRSV/B/Argentina/BA-HNRG-450/2021\|EPI_ISL_15067721\|2021-07-24 |
| >lcl\|Query_554664:5123-5580 | hRSV/B/Argentina/HNRG-450/2021\|EPI_ISL_15055356\|2021-07-24 |
| >lcl\|Query_554677:5123-5580 | hRSV/B/Argentina/BA-HNRG-449/2021\|EPI_ISL_15067720\|2021-07-23 |
| >lcl\|Query_554663:5123-5580 | hRSV/B/Argentina/HNRG-449/2021\|EPI_ISL_15055355\|2021-07-23 |
| >lcl\|Query_554932:5149-5415 | hRSV/B/Israel/4395/2021\|EPI_ISL_13231426\|2021-07-08 |
| >lcl\|Query_554931:5127-5393 | hRSV/B/Israel/4294/2021\|EPI_ISL_13231424\|2021-07-06 |
| >lcl\|Query_554930:5127-5393 | hRSV/B/Israel/4219/2021\|EPI_ISL_13231423\|2021-07-05 |
| >lcl\|Query_554925:5161-5427 | hRSV/B/Israel/3857/2021\|EPI_ISL_13231411\|2021-06-23 |
| >lcl\|Query_554924:5127-5393 | hRSV/B/Israel/3670/2021\|EPI_ISL_13231406\|2021-06-20 |
| >lcl\|Query_554937:5132-5398 | hRSV/B/Israel/4636/2021\|EPI_ISL_13231433\|2021-07-15 |
| >lcl\|Query_554929:5138-5404 | hRSV/B/Israel/4186/2021\|EPI_ISL_13231421\|2021-07-04 |
| >lcl\|Query_554935:5169-5435 | hRSV/B/Israel/4634/2021\|EPI_ISL_13231431\|2021-07-15 |
| >lcl\|Query_107942:5122-5388 | hRSV/B/Israel/4781/2021\|EPI_ISL_13231437\|2021-07-20 |
| >lcl\|Query_107943:5138-5404 | hRSV/B/Israel/4792/2021\|EPI_ISL_13231438\|2021-07-20 |
| >lcl\|Query_554933:5127-5393 | hRSV/B/Israel/4482/2021\|EPI_ISL_13231427\|2021-07-12 |
| >lcl\|Query_554928:5127-5393 | hRSV/B/Israel/4182/2021\|EPI_ISL_13231420\|2021-07-01 |
| >lcl\|Query_554927:5139-5405 | hRSV/B/Israel/4025/2021\|EPI_ISL_13231416\|2021-06-29 |
| >lcl\|Query_107941:5143-5409 | hRSV/B/Israel/3016/2021\|EPI_ISL_13231399\|2021-05-27 |
| >lcl\|Query_554936:5127-5393 | hRSV/B/Israel/4635/2021\|EPI_ISL_13231432\|2021-07-15 |
| >lcl\|Query_554938:5138-5404 | hRSV/B/Israel/342014/2021\|EPI_ISL_13231440\|2021-06-01 |
| >lcl\|Query_554934:5138-5404 | hRSV/B/Israel/4625/2021\|EPI_ISL_13231430\|2021-07-15 |
| >lcl\|Query_554636:5177-5443 | hRSV/B/Spain/CHUVI-21709413/2021\|EPI_ISL_14084082\|2021-10-14 |
| >lcl\|Query_554628:5168-5434 | hRSV/B/Spain/CHUVI-19458518/2021\|EPI_ISL_14084074\|2021-12-06 |
| >lcl\|Query_554629:5131-5397 | hRSV/B/Spain/CHUVI-19458543/2021\|EPI_ISL_14084075\|2021-12-06 |
| >lcl\|Query_554942:5154-5420 | hRSV/B/Spain/CHUVI-34478851/2021\|EPI_ISL_14084086\|2021-12-30 |
| >lcl\|Query_554939:5168-5434 | hRSV/B/Spain/CHUVI-21822405/2022\|EPI_ISL_14084083\|2022-03-03 |
| >lcl\|Query_554634:5168-5434 | hRSV/B/Spain/CHUVI-19482789/2021\|EPI_ISL_14084080\|2021-12-28 |
| >lcl\|Query_554627:5168-5434 | hRSV/B/Spain/CHUVI-19457355/2021\|EPI_ISL_14084073\|2021-12-05 |
| >lcl\|Query_554623:5176-5442 | hRSV/B/Spain/CHUVI-19447304/2021\|EPI_ISL_14084067\|2021-11-14 |
| >lcl\|Query_554941:5168-5434 | hRSV/B/Spain/CHUVI-34269097/2021\|EPI_ISL_14084085\|2021-10-13 |
| >lcl\|Query_554635:5168-5434 | hRSV/B/Spain/CHUVI-19491616/2022\|EPI_ISL_14084081\|2022-01-03 |
| >lcl\|Query_554631:5131-5397 | hRSV/B/Spain/CHUVI-19471353/2021\|EPI_ISL_14084077\|2021-12-18 |
| >lcl\|Query_554626:5179-5445 | hRSV/B/Spain/CHUVI-19454915/2021\|EPI_ISL_14084072\|2021-12-01 |
| >lcl\|Query_554625:5179-5445 | hRSV/B/Spain/CHUVI-19449724/2021\|EPI_ISL_14084070\|2021-11-22 |
| >lcl\|Query_554624:5167-5433 | hRSV/B/Spain/CHUVI-19447968/2021\|EPI_ISL_14084068\|2021-11-17 |
| >lcl\|Query_554622:5131-5397 | hRSV/B/Spain/CHUVI-19446710/2021\|EPI_ISL_14084066\|2021-11-11 |
| >lcl\|Query_554632:5182-5448 | hRSV/B/Spain/CHUVI-19472888/2021\|EPI_ISL_14084078\|2021-12-23 |
| >lcl\|Query_554940:5168-5434 | hRSV/B/Spain/CHUVI-21827199/2022\|EPI_ISL_14084084\|2022-03-10 |
| >lcl\|Query_554630:5177-5443 | hRSV/B/Spain/CHUVI-19470906/2021\|EPI_ISL_14084076\|2021-12-17 |
| >lcl\|Query_554633:5179-5445 | hRSV/B/Spain/CHUVI-19480062/2021\|EPI_ISL_14084079\|2021-12-25 |
| >lcl\|Query_554943:5153-5419 | hRSV/B/Spain/CHUVI-51701569/2021\|EPI_ISL_14084087\|2021-10-11 |
| >lcl\|Query_108016:5177-5631 | hRSV/B/England/194560624/2019\|EPI_ISL_1647504\|2019-11-06 |
| >lcl\|Query_554889:5170-5631 | hRSV/B/Australia/QLD-RBWH102/2021\|EPI_ISL_11817050\|2021-02-14 |
| >lcl\|Query_107925:5187-5643 | hRSV/B/Australia/VIC-MMC067/2020\|EPI_ISL_11817126\|2020-12-28 |
| >lcl\|Query_554825:5139-5596 | hRSV/B/Mongolia/NCCD038/2018\|EPI_ISL_2543889\|2018-02-22 |
| >lcl\|Query_554821:5149-5606 | hRSV/B/Mongolia/NCCD031/2017\|EPI_ISL_2543885\|2017-12-27 |
| >lcl\|Query_554817:5140-5597 | hRSV/B/Mongolia/NCCD025/2017\|EPI_ISL_2543881\|2017-12-25 |
| >lcl\|Query_554813:5143-5600 | hRSV/B/Mongolia/NCCD019/2017\|EPI_ISL_2543877\|2017-12-18 |
| >lcl\|Query_554812:5140-5597 | hRSV/B/Mongolia/NCCD018/2018\|EPI_ISL_2543876\|2018-01-29 |
| >lcl\|Query_554811:5141-5598 | hRSV/B/Mongolia/NCCD018/2017\|EPI_ISL_2543875\|2017-12-18 |
| >lcl\|Query_554808:5142-5599 | hRSV/B/Mongolia/NCCD015/2017\|EPI_ISL_2543872\|2017-12-04 |
| >lcl\|Query_554807:5144-5601 | hRSV/B/Mongolia/NCCD014/2018\|EPI_ISL_2543871\|2018-01-17 |
| >lcl\|Query_554806:5143-5600 | hRSV/B/Mongolia/NCCD014/2017\|EPI_ISL_2543870\|2017-12-04 |
| >lcl\|Query_554805:5140-5597 | hRSV/B/Mongolia/NCCD012/2017\|EPI_ISL_2543869\|2017-11-29 |
| >lcl\|Query_554804:5140-5597 | hRSV/B/Mongolia/NCCD011/2017\|EPI_ISL_2543868\|2017-11-29 |
| >lcl\|Query_554799:5139-5596 | hRSV/B/Mongolia/NCCD004/2017\|EPI_ISL_2543863\|2017-11-13 |
| >lcl\|Query_554796:5139-5596 | hRSV/B/Mongolia/NCCD001/2017\|EPI_ISL_2543860\|2017-10-30 |
| >lcl\|Query_554767:5147-5604 | hRSV/B/Australia/VIC-RCH075/2017\|EPI_ISL_1760383\|2017-07-22 |
| >lcl\|Query_554869:5150-5607 | hRSV/B/Argentina/BA-HNRG-383/2017\|EPI_ISL_1074092\|2017-07-07 |
| >lcl\|Query_554865:5155-5612 | hRSV/B/Argentina/BA-HNRG-325/2017\|EPI_ISL_1074032\|2017-05-24 |
| >lcl\|Query_107863:5150-5607 | hRSV/B/Argentina/BA-HNRG-383/2017\|EPI_ISL_412460\|2017-07-07 |
| >lcl\|Query_554795:5140-5597 | hRSV/B/Cote_d'Ivoire/IPCI-020/2017\|EPI_ISL_2543859\|2017-09-03 |
| >lcl\|Query_554955:5193-5650 | hRSV/B/Mozambique/MAP-INS004/2017\|EPI_ISL_1647475\|2017-03-21 |
| >lcl\|Query_554950:5193-5650 | hRSV/B/Mozambique/MAP-INS001/2017\|EPI_ISL_1647470\|2017-03-17 |
| >lcl\|Query_108005:5193-5650 | hRSV/B/Mozambique/MAP-INS009/2017\|EPI_ISL_1647483\|2017-04-03 |
| >lcl\|Query_108004:5193-5650 | hRSV/B/Mozambique/MAP-INS008/2017\|EPI_ISL_1647482\|2017-04-03 |
| >lcl\|Query_108007:5195-5652 | hRSV/B/Mozambique/MAP-INS010/2018\|EPI_ISL_1647485\|2018-05-08 |
| >lcl\|Query_108003:5193-5650 | hRSV/B/Mozambique/MAP-INS007/2018\|EPI_ISL_1647481\|2018-04-05 |
| >lcl\|Query_108006:5193-5650 | hRSV/B/Mozambique/MAP-INS010/2017\|EPI_ISL_1647484\|2017-04-11 |
| >lcl\|Query_108008:5195-5652 | hRSV/B/Mozambique/MAP-INS011/2017\|EPI_ISL_1647486\|2017-04-18 |
| >lcl\|Query_554793:5143-5600 | hRSV/B/Cote_d'Ivoire/IPCI-003/2017\|EPI_ISL_2543857\|2017-08-22 |
| >lcl\|Query_108134:5188-5645 | hRSV/B/Cote_d'Ivoire/IPCI-007/2017\|EPI_ISL_6174132\|2017-08-20 |
| >lcl\|Query_554841:5173-5630 | hRSV/B/Kenya/KHDSS-204-19-MTO/2017\|EPI_ISL_2585033\|2017-05-04 |
| >lcl\|Query_108120:5178-5635 | hRSV/B/Kenya/KHDSS-33-16-CHA/2017\|EPI_ISL_2584977\|2017-05-10 |
| >lcl\|Query_107819:5121-5578 | hRSV/B/Kenya/KHDSS-22-29-CHA/2017\|EPI_ISL_2585115\|2017-04-04 |
| >lcl\|Query_108115:5162-5619 | hRSV/B/Kenya/KHDSS-194-28-MTO/2017\|EPI_ISL_2584970\|2017-03-22 |
| >lcl\|Query_554840:5146-5603 | hRSV/B/Kenya/KHDSS-20-13-CHA/2017\|EPI_ISL_2585032\|2017-04-12 |
| >lcl\|Query_554837:5145-5602 | hRSV/B/Kenya/KHDSS-17-09-CHA/2017\|EPI_ISL_2585028\|2017-05-12 |
| >lcl\|Query_108095:5178-5635 | hRSV/B/Kenya/KHDSS-259-27-PIN/2017\|EPI_ISL_2584927\|2017-02-20 |
| >lcl\|Query_108094:5178-5635 | hRSV/B/Kenya/KHDSS-233-04-NGE/2017\|EPI_ISL_2584926\|2017-03-29 |
| >lcl\|Query_107835:5146-5603 | hRSV/B/Kenya/KHDSS-72-20-JUN/2017\|EPI_ISL_2585136\|2017-01-19 |
| >lcl\|Query_107793:5178-5635 | hRSV/B/Kenya/KHDSS-26-13-CHA/2017\|EPI_ISL_2584869\|2017-03-13 |
| >lcl\|Query_108085:5178-5635 | hRSV/B/Kenya/KHDSS-173-16-MAV/2017\|EPI_ISL_2584917\|2017-05-16 |
| >lcl\|Query_554958:5193-5650 | hRSV/B/Mozambique/MAP-INS005/2018\|EPI_ISL_1647478\|2018-03-19 |
| >lcl\|Query_107988:5175-5632 | hRSV/B/Morocco/1231-17/2017\|EPI_ISL_15120767\|2017-03-09 |
| >lcl\|Query_554820:5143-5600 | hRSV/B/Mongolia/NCCD029/2017\|EPI_ISL_2543884\|2017-12-25 |
| >lcl\|Query_554819:5143-5600 | hRSV/B/Mongolia/NCCD028/2017\|EPI_ISL_2543883\|2017-12-25 |
| >lcl\|Query_554816:5140-5597 | hRSV/B/Mongolia/NCCD020/2018\|EPI_ISL_2543880\|2018-01-29 |
| >lcl\|Query_554815:5141-5598 | hRSV/B/Mongolia/NCCD020/2017\|EPI_ISL_2543879\|2017-12-18 |
| >lcl\|Query_554802:5139-5596 | hRSV/B/Mongolia/NCCD010/2017\|EPI_ISL_2543866\|2017-11-28 |
| >lcl\|Query_554798:5143-5600 | hRSV/B/Mongolia/NCCD003/2018\|EPI_ISL_2543862\|2018-01-08 |
| >lcl\|Query_554797:5143-5600 | hRSV/B/Mongolia/NCCD001/2018\|EPI_ISL_2543861\|2018-01-02 |
| >lcl\|Query_107967:5176-5633 | hRSV/B/Morocco/650-17/2017\|EPI_ISL_15120746\|2017-01-19 |
| >lcl\|Query_107895:5170-5627 | hRSV/B/England/180480656/2018\|EPI_ISL_732355\|2018-01-23 |
| >lcl\|Query_108138:5190-5647 | hRSV/B/Egypt/C-CPHL002/2019\|EPI_ISL_6174138\|2019-01-08 |
| >lcl\|Query_108135:5188-5645 | hRSV/B/Cote_d'Ivoire/IPCI-029/2017\|EPI_ISL_6174133\|2017-09-20 |
| >lcl\|Query_554959:5193-5650 | hRSV/B/Mozambique/MAP-INS006/2017\|EPI_ISL_1647479\|2017-03-28 |
| >lcl\|Query_554953:5193-5650 | hRSV/B/Mozambique/MAP-INS003/2017\|EPI_ISL_1647473\|2017-03-21 |
| >lcl\|Query_108011:5192-5649 | hRSV/B/Mozambique/MAP-INS020/2017\|EPI_ISL_1647489\|2017-03-14 |
| >lcl\|Query_554957:5195-5652 | hRSV/B/Mozambique/MAP-INS005/2017\|EPI_ISL_1647477\|2017-03-23 |
| >lcl\|Query_108002:5193-5650 | hRSV/B/Mozambique/MAP-INS007/2017\|EPI_ISL_1647480\|2017-04-04 |
| >lcl\|Query_108009:5148-5605 | hRSV/B/Mozambique/MAP-INS016/2017\|EPI_ISL_1647487\|2017-02-15 |
| >lcl\|Query_554956:5147-5604 | hRSV/B/Mozambique/MAP-INS004/2018\|EPI_ISL_1647476\|2018-03-13 |
| >lcl\|Query_107970:5176-5633 | hRSV/B/Morocco/389-17/2017\|EPI_ISL_15120749\|2017-01-02 |
| >lcl\|Query_554866:5152-5609 | hRSV/B/Argentina/BA-HNRG-302/2017\|EPI_ISL_1074085\|2017-04-10 |
| >lcl\|Query_107892:5191-5648 | hRSV/B/England/180160871/2018\|EPI_ISL_732352\|2018-01-02 |
| >lcl\|Query_554855:5178-5635 | hRSV/B/Kenya/KHDSS-48-08-JAR/2017\|EPI_ISL_2585057\|2017-03-07 |
| >lcl\|Query_108123:5145-5602 | hRSV/B/Kenya/KHDSS-52-14-JAR/2017\|EPI_ISL_2584982\|2017-03-08 |
| >lcl\|Query_108121:5146-5603 | hRSV/B/Kenya/KHDSS-49-08-JAR/2017\|EPI_ISL_2584980\|2017-03-08 |
| >lcl\|Query_108077:5145-5602 | hRSV/B/Kenya/KHDSS-45-01-JAR/2017\|EPI_ISL_2584719\|2017-01-25 |
| >lcl\|Query_554856:5145-5602 | hRSV/B/Kenya/KHDSS-54-29-JAR/2017\|EPI_ISL_2585058\|2017-03-14 |
| >lcl\|Query_108116:5178-5635 | hRSV/B/Kenya/KHDSS-257-06-PIN/2017\|EPI_ISL_2584972\|2017-02-06 |
| >lcl\|Query_108125:5145-5602 | hRSV/B/Kenya/KHDSS-83-28-JUN/2017\|EPI_ISL_2584985\|2017-03-20 |
| >lcl\|Query_108124:5145-5602 | hRSV/B/Kenya/KHDSS-75-30-JUN/2017\|EPI_ISL_2584983\|2017-01-30 |
| >lcl\|Query_107802:5178-5635 | hRSV/B/Kenya/KHDSS-74-30-JUN/2017\|EPI_ISL_2585060\|2017-01-24 |
| >lcl\|Query_554854:5178-5635 | hRSV/B/Kenya/KHDSS-47-07-JAR/2017\|EPI_ISL_2585056\|2017-03-01 |
| >lcl\|Query_554771:5147-5604 | hRSV/B/Australia/VIC-RCH024/2017\|EPI_ISL_1760387\|2017-06-23 |
| >lcl\|Query_107969:5176-5633 | hRSV/B/Morocco/694-17/2017\|EPI_ISL_15120748\|2017-01-24 |
| >lcl\|Query_107965:5176-5633 | hRSV/B/Morocco/610-17/2017\|EPI_ISL_15120742\|2017-01-17 |
| >lcl\|Query_107963:5176-5633 | hRSV/B/Morocco/558-17/2017\|EPI_ISL_15120740\|2017-01-12 |
| >lcl\|Query_107992:5176-5633 | hRSV/B/Morocco/220-19/2018\|EPI_ISL_15120784\|2018-12-13 |
| >lcl\|Query_108065:5192-5649 | hRSV/B/Australia/VIC-RCH129/2017\|EPI_ISL_1760436\|2017-08-30 |
| >lcl\|Query_108080:5178-5635 | hRSV/B/Kenya/KHDSS-118-11-MAT/2017\|EPI_ISL_2584912\|2017-05-02 |
| >lcl\|Query_108096:5163-5620 | hRSV/B/Kenya/KHDSS-264-10-PIN/2017\|EPI_ISL_2584928\|2017-04-03 |
| >lcl\|Query_108088:5178-5635 | hRSV/B/Kenya/KHDSS-198-06-MTO/2017\|EPI_ISL_2584920\|2017-04-06 |
| >lcl\|Query_107836:5146-5603 | hRSV/B/Kenya/KHDSS-80-15-JUN/2017\|EPI_ISL_2585137\|2017-03-06 |
| >lcl\|Query_108127:5152-5609 | hRSV/B/Kenya/KHDSS-76-20-JUN/2017\|EPI_ISL_2585183\|2017-01-30 |
| >lcl\|Query_108126:5151-5608 | hRSV/B/Kenya/KHDSS-51-08-JAR/2017\|EPI_ISL_2585181\|2017-03-08 |
| >lcl\|Query_108075:5178-5635 | hRSV/B/Kenya/KHDSS-81-15-JUN/2017\|EPI_ISL_2584504\|2017-03-15 |
| >lcl\|Query_107823:5146-5603 | hRSV/B/Kenya/KHDSS-261-13-PIN/2017\|EPI_ISL_2585121\|2017-02-27 |
| >lcl\|Query_108074:5179-5636 | hRSV/B/Kenya/KHDSS-262-13-PIN/2017\|EPI_ISL_2584500\|2017-04-13 |
| >lcl\|Query_107832:5173-5630 | hRSV/B/Kenya/KHDSS-57-30-JAR/2017\|EPI_ISL_2585132\|2017-03-30 |
| >lcl\|Query_554778:5147-5604 | hRSV/B/Australia/VIC-RCH118/2018\|EPI_ISL_1760394\|2018-07-02 |
| >lcl\|Query_554777:5193-5650 | hRSV/B/Australia/VIC-RCH087/2018\|EPI_ISL_1760393\|2018-06-09 |
| >lcl\|Query_554833:5178-5635 | hRSV/B/Kenya/KHDSS-119-18-MAT/2017\|EPI_ISL_2585020\|2017-05-11 |
| >lcl\|Query_108128:5146-5603 | hRSV/B/Kenya/KHDSS-79-06-JUN/2017\|EPI_ISL_2585184\|2017-03-06 |
| >lcl\|Query_107842:5178-5635 | hRSV/B/Kenya/KHDSS-203-04-MTO/2017\|EPI_ISL_2585165\|2017-04-25 |
| >lcl\|Query_107839:5171-5628 | hRSV/B/Kenya/KHDSS-192-16-MTO/2017\|EPI_ISL_2585162\|2017-03-15 |
| >lcl\|Query_107838:5181-5638 | hRSV/B/Kenya/KHDSS-141-25-MAV/2017\|EPI_ISL_2585160\|2017-01-17 |
| >lcl\|Query_107783:5178-5635 | hRSV/B/Kenya/KHDSS-23-29-CHA/2017\|EPI_ISL_2584494\|2017-05-04 |
| >lcl\|Query_554848:5146-5603 | hRSV/B/Kenya/KHDSS-284-23-SOK/2017\|EPI_ISL_2585049\|2017-01-30 |
| >lcl\|Query_107837:5183-5640 | hRSV/B/Kenya/KHDSS-114-04-MAT/2017\|EPI_ISL_2585158\|2017-03-30 |
| >lcl\|Query_107840:5145-5602 | hRSV/B/Kenya/KHDSS-195-28-MTO/2017\|EPI_ISL_2585163\|2017-03-28 |
| >lcl\|Query_107846:5145-5602 | hRSV/B/Kenya/KHDSS-296-18-SOK/2017\|EPI_ISL_2585176\|2017-04-13 |
| >lcl\|Query_107843:5146-5603 | hRSV/B/Kenya/KHDSS-226-04-NGE/2017\|EPI_ISL_2585168\|2017-04-04 |
| >lcl\|Query_107848:5150-5607 | hRSV/B/Kenya/KHDSS-299-10-SOK/2017\|EPI_ISL_2585178\|2017-04-18 |
| >lcl\|Query_107847:5145-5602 | hRSV/B/Kenya/KHDSS-298-18-SOK/2017\|EPI_ISL_2585177\|2017-04-18 |
| >lcl\|Query_107845:5146-5603 | hRSV/B/Kenya/KHDSS-293-13-SOK/2017\|EPI_ISL_2585175\|2017-04-10 |
| >lcl\|Query_107826:5150-5607 | hRSV/B/Kenya/KHDSS-290-06-SOK/2017\|EPI_ISL_2585125\|2017-04-06 |
| >lcl\|Query_107821:5146-5603 | hRSV/B/Kenya/KHDSS-228-12-NGE/2017\|EPI_ISL_2585117\|2017-04-05 |
| >lcl\|Query_107807:5152-5609 | hRSV/B/Kenya/KHDSS-153-06-MAV/2017\|EPI_ISL_2585101\|2017-04-06 |
| >lcl\|Query_107782:5145-5602 | hRSV/B/Kenya/KHDSS-196-28-MTO/2017\|EPI_ISL_2584492\|2017-03-28 |
| >lcl\|Query_107830:5146-5603 | hRSV/B/Kenya/KHDSS-35-23-CHA/2017\|EPI_ISL_2585130\|2017-05-18 |
| >lcl\|Query_107844:5146-5603 | hRSV/B/Kenya/KHDSS-266-24-PIN/2017\|EPI_ISL_2585172\|2017-04-24 |
| >lcl\|Query_108103:5186-5643 | hRSV/B/Kenya/KHDSS-88-29-JUN/2017\|EPI_ISL_2584935\|2017-05-15 |
| >lcl\|Query_554850:5148-5604 | hRSV/B/Kenya/KHDSS-295-13-SOK/2017\|EPI_ISL_2585051\|2017-04-13 |
| >lcl\|Query_107828:5152-5608 | hRSV/B/Kenya/KHDSS-294-13-SOK/2017\|EPI_ISL_2585127\|2017-04-13 |
| >lcl\|Query_107827:5159-5615 | hRSV/B/Kenya/KHDSS-291-10-SOK/2017\|EPI_ISL_2585126\|2017-05-02 |
| >lcl\|Query_107825:5145-5601 | hRSV/B/Kenya/KHDSS-287-27-SOK/2017\|EPI_ISL_2585124\|2017-03-14 |
| >lcl\|Query_107824:5152-5608 | hRSV/B/Kenya/KHDSS-285-14-SOK/2017\|EPI_ISL_2585123\|2017-02-23 |
| >lcl\|Query_107818:5145-5601 | hRSV/B/Kenya/KHDSS-199-06-MTO/2017\|EPI_ISL_2585113\|2017-04-06 |
| >lcl\|Query_107817:5146-5602 | hRSV/B/Kenya/KHDSS-193-22-MTO/2017\|EPI_ISL_2585112\|2017-03-16 |
| >lcl\|Query_554794:5140-5597 | hRSV/B/Cote_d'Ivoire/IPCI-026/2017\|EPI_ISL_2543858\|2017-09-04 |
| >lcl\|Query_554849:5146-5603 | hRSV/B/Kenya/KHDSS-286-14-SOK/2017\|EPI_ISL_2585050\|2017-03-14 |
| >lcl\|Query_554851:5145-5602 | hRSV/B/Kenya/KHDSS-297-18-SOK/2017\|EPI_ISL_2585052\|2017-04-18 |
| >lcl\|Query_554847:5145-5602 | hRSV/B/Kenya/KHDSS-258-20-PIN/2017\|EPI_ISL_2585043\|2017-02-06 |
| >lcl\|Query_554843:5146-5603 | hRSV/B/Kenya/KHDSS-230-13-NGE/2017\|EPI_ISL_2585037\|2017-04-12 |
| >lcl\|Query_554842:5173-5630 | hRSV/B/Kenya/KHDSS-222-04-NGE/2017\|EPI_ISL_2585036\|2017-03-30 |
| >lcl\|Query_554832:5178-5635 | hRSV/B/Kenya/KHDSS-113-30-MAT/2017\|EPI_ISL_2585019\|2017-03-16 |
| >lcl\|Query_554831:5187-5644 | hRSV/B/Kenya/KHDSS-111-16-MAT/2017\|EPI_ISL_2585018\|2017-03-16 |
| >lcl\|Query_554830:5146-5603 | hRSV/B/Kenya/KHDSS-216-02-NGE/2017\|EPI_ISL_2584591\|2017-02-24 |
| >lcl\|Query_108164:5176-5633 | hRSV/B/Morocco/737-19/2019\|EPI_ISL_15120795\|2019-01-28 |
| >lcl\|Query_108137:5190-5647 | hRSV/B/Cote_d'Ivoire/IPCI-006/2018\|EPI_ISL_6174137\|2018-07-09 |
| >lcl\|Query_108117:5180-5637 | hRSV/B/Kenya/KHDSS-263-03-PIN/2017\|EPI_ISL_2584973\|2017-03-13 |
| >lcl\|Query_108105:5178-5635 | hRSV/B/Kenya/KHDSS-110-16-MAT/2017\|EPI_ISL_2584956\|2017-03-07 |
| >lcl\|Query_108104:5141-5598 | hRSV/B/Kenya/KHDSS-109-07-MAT/2017\|EPI_ISL_2584955\|2017-03-02 |
| >lcl\|Query_108102:5146-5603 | hRSV/B/Kenya/KHDSS-84-10-JUN/2017\|EPI_ISL_2584934\|2017-03-28 |
| >lcl\|Query_108101:5152-5609 | hRSV/B/Kenya/KHDSS-60-11-JAR/2017\|EPI_ISL_2584933\|2017-04-11 |
| >lcl\|Query_108097:5145-5602 | hRSV/B/Kenya/KHDSS-288-06-SOK/2017\|EPI_ISL_2584929\|2017-03-27 |
| >lcl\|Query_108092:5178-5635 | hRSV/B/Kenya/KHDSS-229-12-NGE/2017\|EPI_ISL_2584924\|2017-04-12 |
| >lcl\|Query_108091:5178-5635 | hRSV/B/Kenya/KHDSS-223-04-NGE/2017\|EPI_ISL_2584923\|2017-03-29 |
| >lcl\|Query_108090:5173-5630 | hRSV/B/Kenya/KHDSS-219-30-NGE/2017\|EPI_ISL_2584922\|2017-03-16 |
| >lcl\|Query_108089:5152-5609 | hRSV/B/Kenya/KHDSS-218-16-NGE/2017\|EPI_ISL_2584921\|2017-03-07 |
| >lcl\|Query_108087:5146-5603 | hRSV/B/Kenya/KHDSS-190-15-MTO/2017\|EPI_ISL_2584919\|2017-03-15 |
| >lcl\|Query_108083:5178-5635 | hRSV/B/Kenya/KHDSS-155-06-MAV/2017\|EPI_ISL_2584915\|2017-04-06 |
| >lcl\|Query_107834:5146-5603 | hRSV/B/Kenya/KHDSS-62-24-JAR/2017\|EPI_ISL_2585134\|2017-04-27 |
| >lcl\|Query_107833:5171-5628 | hRSV/B/Kenya/KHDSS-58-07-JAR/2017\|EPI_ISL_2585133\|2017-03-30 |
| >lcl\|Query_107829:5145-5602 | hRSV/B/Kenya/KHDSS-34-18-CHA/2017\|EPI_ISL_2585128\|2017-05-16 |
| >lcl\|Query_107822:5161-5618 | hRSV/B/Kenya/KHDSS-260-27-PIN/2017\|EPI_ISL_2585120\|2017-02-27 |
| >lcl\|Query_107816:5146-5603 | hRSV/B/Kenya/KHDSS-19-08-CHA/2017\|EPI_ISL_2585111\|2017-03-15 |
| >lcl\|Query_107815:5178-5635 | hRSV/B/Kenya/KHDSS-188-23-MTO/2017\|EPI_ISL_2585110\|2017-01-18 |
| >lcl\|Query_107808:5163-5620 | hRSV/B/Kenya/KHDSS-154-06-MAV/2017\|EPI_ISL_2585102\|2017-04-06 |
| >lcl\|Query_107803:5147-5604 | hRSV/B/Kenya/KHDSS-82-20-JUN/2017\|EPI_ISL_2585061\|2017-03-15 |
| >lcl\|Query_107801:5145-5602 | hRSV/B/Kenya/KHDSS-225-04-NGE/2017\|EPI_ISL_2584887\|2017-04-04 |
| >lcl\|Query_107800:5178-5635 | hRSV/B/Kenya/KHDSS-221-30-NGE/2017\|EPI_ISL_2584886\|2017-03-30 |
| >lcl\|Query_107799:5178-5635 | hRSV/B/Kenya/KHDSS-220-30-NGE/2017\|EPI_ISL_2584885\|2017-03-30 |
| >lcl\|Query_107798:5178-5635 | hRSV/B/Kenya/KHDSS-202-25-MTO/2017\|EPI_ISL_2584884\|2017-03-13 |
| >lcl\|Query_107797:5180-5637 | hRSV/B/Kenya/KHDSS-201-12-MTO/2017\|EPI_ISL_2584883\|2017-04-12 |
| >lcl\|Query_107796:5180-5637 | hRSV/B/Kenya/KHDSS-189-15-MTO/2017\|EPI_ISL_2584882\|2017-01-23 |
| >lcl\|Query_107794:5146-5603 | hRSV/B/Kenya/KHDSS-61-27-JAR/2017\|EPI_ISL_2584871\|2017-04-11 |
| >lcl\|Query_107790:5146-5603 | hRSV/B/Kenya/KHDSS-200-12-MTO/2017\|EPI_ISL_2584866\|2017-04-06 |
| >lcl\|Query_107787:5153-5610 | hRSV/B/Kenya/KHDSS-78-06-JUN/2017\|EPI_ISL_2584674\|2017-02-20 |
| >lcl\|Query_107786:5179-5636 | hRSV/B/Kenya/KHDSS-77-20-JUN/2017\|EPI_ISL_2584673\|2017-02-20 |
| >lcl\|Query_107785:5167-5624 | hRSV/B/Kenya/KHDSS-86-24-JUN/2017\|EPI_ISL_2584670\|2017-04-10 |
| >lcl\|Query_107784:5146-5603 | hRSV/B/Kenya/KHDSS-87-15-JUN/2017\|EPI_ISL_2584665\|2017-04-24 |
| >lcl\|Query_554846:5178-5635 | hRSV/B/Kenya/KHDSS-256-06-PIN/2017\|EPI_ISL_2585042\|2017-01-30 |
| >lcl\|Query_554845:5152-5609 | hRSV/B/Kenya/KHDSS-255-30-PIN/2017\|EPI_ISL_2585041\|2017-01-11 |
| >lcl\|Query_554836:5183-5640 | hRSV/B/Kenya/KHDSS-165-28-MAV/2017\|EPI_ISL_2585027\|2017-04-28 |
| >lcl\|Query_108099:5173-5630 | hRSV/B/Kenya/KHDSS-36-23-CHA/2017\|EPI_ISL_2584931\|2017-05-23 |
| >lcl\|Query_108093:5173-5630 | hRSV/B/Kenya/KHDSS-231-13-NGE/2017\|EPI_ISL_2584925\|2017-04-13 |
| >lcl\|Query_108119:5146-5603 | hRSV/B/Kenya/KHDSS-292-10-SOK/2017\|EPI_ISL_2584976\|2017-04-10 |
| >lcl\|Query_108106:5146-5603 | hRSV/B/Kenya/KHDSS-117-02-MAT/2017\|EPI_ISL_2584958\|2017-05-02 |
| >lcl\|Query_107789:5152-5609 | hRSV/B/Kenya/KHDSS-160-11-MAV/2017\|EPI_ISL_2584856\|2017-04-11 |
| >lcl\|Query_554839:5178-5635 | hRSV/B/Kenya/KHDSS-191-15-MTO/2017\|EPI_ISL_2585031\|2017-03-08 |
| >lcl\|Query_108086:5180-5637 | hRSV/B/Kenya/KHDSS-187-18-MTO/2017\|EPI_ISL_2584918\|2017-01-12 |
| >lcl\|Query_107792:5146-5603 | hRSV/B/Kenya/KHDSS-234-30-NGE/2017\|EPI_ISL_2584868\|2017-05-04 |
| >lcl\|Query_107791:5146-5603 | hRSV/B/Kenya/KHDSS-232-04-NGE/2017\|EPI_ISL_2584867\|2017-04-13 |
| >lcl\|Query_107805:5178-5635 | hRSV/B/Kenya/KHDSS-116-02-MAT/2017\|EPI_ISL_2585098\|2017-04-13 |
| >lcl\|Query_108045:5147-5604 | hRSV/B/Australia/VIC-RCH102/2017\|EPI_ISL_1760416\|2017-08-01 |
| >lcl\|Query_108114:5163-5620 | hRSV/B/Kenya/KHDSS-171-16-MAV/2017\|EPI_ISL_2584969\|2017-01-09 |
| >lcl\|Query_108113:5178-5635 | hRSV/B/Kenya/KHDSS-169-03-MAV/2017\|EPI_ISL_2584968\|2017-05-02 |
| >lcl\|Query_108112:5161-5618 | hRSV/B/Kenya/KHDSS-163-21-MAV/2017\|EPI_ISL_2584967\|2017-04-19 |
| >lcl\|Query_108109:5178-5635 | hRSV/B/Kenya/KHDSS-156-11-MAV/2017\|EPI_ISL_2584963\|2017-04-06 |
| >lcl\|Query_108107:5180-5637 | hRSV/B/Kenya/KHDSS-145-08-MAV/2017\|EPI_ISL_2584960\|2017-03-01 |
| >lcl\|Query_108084:5178-5635 | hRSV/B/Kenya/KHDSS-161-19-MAV/2017\|EPI_ISL_2584916\|2017-04-11 |
| >lcl\|Query_108082:5178-5635 | hRSV/B/Kenya/KHDSS-146-14-MAV/2017\|EPI_ISL_2584914\|2017-03-08 |
| >lcl\|Query_108081:5145-5602 | hRSV/B/Kenya/KHDSS-142-01-MAV/2017\|EPI_ISL_2584913\|2017-01-25 |
| >lcl\|Query_107788:5163-5620 | hRSV/B/Kenya/KHDSS-144-01-MAV/2017\|EPI_ISL_2584690\|2017-03-01 |
| >lcl\|Query_554870:5152-5609 | hRSV/B/Argentina/BA-HNRG-393/2017\|EPI_ISL_1074224\|2017-07-24 |
| >lcl\|Query_554868:5151-5608 | hRSV/B/Argentina/BA-HNRG-378/2017\|EPI_ISL_1074087\|2017-07-03 |
| >lcl\|Query_107973:5176-5633 | hRSV/B/Morocco/983-17/2017\|EPI_ISL_15120752\|2017-02-14 |
| >lcl\|Query_107985:5176-5633 | hRSV/B/Morocco/1023-17/2017\|EPI_ISL_15120764\|2017-02-11 |
| >lcl\|Query_554770:5148-5605 | hRSV/B/Australia/VIC-RCH131/2018\|EPI_ISL_1760386\|2018-07-15 |
| >lcl\|Query_554972:5177-5634 | hRSV/B/England/RE20000008/2019\|EPI_ISL_1647598\|2019-12-26 |
| >lcl\|Query_554780:5146-5603 | hRSV/B/Australia/VIC-RCH009/2018\|EPI_ISL_1760396\|2018-01-20 |
| >lcl\|Query_554952:5192-5649 | hRSV/B/Mozambique/MAP-INS002/2018\|EPI_ISL_1647472\|2018-02-27 |
| >lcl\|Query_108040:5149-5606 | hRSV/B/Australia/VIC-RCH097/2017\|EPI_ISL_1760411\|2017-08-01 |
| >lcl\|Query_554766:5190-5647 | hRSV/B/England/RE20000005/2019\|EPI_ISL_1647595\|2019-12-24 |
| >lcl\|Query_554765:5182-5639 | hRSV/B/England/RE19003269/2019\|EPI_ISL_1647594\|2019-12-17 |
| >lcl\|Query_554742:5177-5634 | hRSV/B/England/200100154/2019\|EPI_ISL_1647571\|2019-12-27 |
| >lcl\|Query_554684:5193-5650 | hRSV/B/England/194640447/2019\|EPI_ISL_1647513\|2019-11-11 |
| >lcl\|Query_107913:5139-5596 | hRSV/B/South_Africa/NICD-R08250/2021\|EPI_ISL_11055793\|2021-06-01 |
| >lcl\|Query_554717:5191-5648 | hRSV/B/England/194980228/2019\|EPI_ISL_1647546\|2019-12-04 |
| >lcl\|Query_554984:5194-5651 | hRSV/B/England/200640676/2020\|EPI_ISL_1834183\|2020-02-04 |
| >lcl\|Query_554746:5194-5651 | hRSV/B/England/200181054/2020\|EPI_ISL_1647575\|2020-01-02 |
| >lcl\|Query_554739:5189-5646 | hRSV/B/England/195200238/2019\|EPI_ISL_1647568\|2019-12-20 |
| >lcl\|Query_554701:5176-5633 | hRSV/B/England/194860180/2019\|EPI_ISL_1647530\|2019-11-27 |
| >lcl\|Query_554694:5191-5648 | hRSV/B/England/194740504/2019\|EPI_ISL_1647523\|2019-11-18 |
| >lcl\|Query_554691:5178-5635 | hRSV/B/England/194720340/2019\|EPI_ISL_1647520\|2019-11-18 |
| >lcl\|Query_554682:5193-5650 | hRSV/B/England/194640433/2019\|EPI_ISL_1647511\|2019-11-08 |
| >lcl\|Query_554721:5189-5646 | hRSV/B/England/195040637/2019\|EPI_ISL_1647550\|2019-12-09 |
| >lcl\|Query_554985:5164-5621 | hRSV/B/England/200880459/2020\|EPI_ISL_1834184\|2020-02-19 |
| >lcl\|Query_554916:5139-5596 | hRSV/B/South_Africa/NICD-R09473/2021\|EPI_ISL_12529659\|2021-06-24 |
| >lcl\|Query_554981:5188-5645 | hRSV/B/England/200520542/2020\|EPI_ISL_1834180\|2020-01-23 |
| >lcl\|Query_554970:5194-5651 | hRSV/B/England/RE20000006/2019\|EPI_ISL_1647596\|2019-12-24 |
| >lcl\|Query_554964:5170-5627 | hRSV/B/England/194360578/2019\|EPI_ISL_1647494\|2019-10-23 |
| >lcl\|Query_554745:5192-5649 | hRSV/B/England/200120638/2019\|EPI_ISL_1647574\|2019-12-30 |
| >lcl\|Query_554731:5177-5634 | hRSV/B/England/195100504/2019\|EPI_ISL_1647560\|2019-12-13 |
| >lcl\|Query_554697:5177-5634 | hRSV/B/England/194800146/2019\|EPI_ISL_1647526\|2019-11-22 |
| >lcl\|Query_554693:5191-5648 | hRSV/B/England/194740482/2019\|EPI_ISL_1647522\|2019-11-19 |
| >lcl\|Query_554685:5189-5646 | hRSV/B/England/194640818/2019\|EPI_ISL_1647514\|2019-11-08 |
| >lcl\|Query_554681:5189-5646 | hRSV/B/England/194640432/2019\|EPI_ISL_1647510\|2019-11-11 |
| >lcl\|Query_554754:5194-5651 | hRSV/B/England/200280372/2020\|EPI_ISL_1647583\|2020-01-08 |
| >lcl\|Query_554711:5183-5640 | hRSV/B/England/194940300/2019\|EPI_ISL_1647540\|2019-12-03 |
| >lcl\|Query_554699:5194-5651 | hRSV/B/England/194840119/2019\|EPI_ISL_1647528\|2019-11-20 |
| >lcl\|Query_108013:5177-5634 | hRSV/B/England/194520779/2019\|EPI_ISL_1647501\|2019-11-04 |
| >lcl\|Query_108159:5195-5652 | hRSV/B/England/190100016/2018\|EPI_ISL_732373\|2018-12-27 |
| >lcl\|Query_554971:5192-5649 | hRSV/B/England/RE20000007/2019\|EPI_ISL_1647597\|2019-12-19 |
| >lcl\|Query_554763:5198-5655 | hRSV/B/England/RE19003144/2019\|EPI_ISL_1647592\|2019-12-09 |
| >lcl\|Query_554730:5182-5639 | hRSV/B/England/195100393/2019\|EPI_ISL_1647559\|2019-12-10 |
| >lcl\|Query_554727:5192-5649 | hRSV/B/England/195100232/2019\|EPI_ISL_1647556\|2019-12-13 |
| >lcl\|Query_554704:5178-5635 | hRSV/B/England/194860195/2019\|EPI_ISL_1647533\|2019-11-26 |
| >lcl\|Query_108019:5183-5640 | hRSV/B/England/194580271/2019\|EPI_ISL_1647507\|2019-11-06 |
| >lcl\|Query_108014:5197-5654 | hRSV/B/England/194520780/2019\|EPI_ISL_1647502\|2019-11-04 |
| >lcl\|Query_554922:5175-5632 | hRSV/B/Philippines/99067/2019\|EPI_ISL_12970410\|2019-09-02 |
| >lcl\|Query_554918:5176-5633 | hRSV/B/Philippines/99056/2019\|EPI_ISL_12970400\|2019-01-15 |
| >lcl\|Query_554760:5193-5650 | hRSV/B/England/201181421/2020\|EPI_ISL_1647589\|2020-03-12 |
| >lcl\|Query_108066:5142-5599 | hRSV/B/Australia/VIC-RCH018/2018\|EPI_ISL_1760437\|2018-02-22 |
| >lcl\|Query_554781:5146-5603 | hRSV/B/Australia/VIC-RCH055/2017\|EPI_ISL_1760397\|2017-07-09 |
| >lcl\|Query_554988:5192-5649 | hRSV/B/Australia/VIC-RCH013/2017\|EPI_ISL_2156817\|2017-06-08 |
| >lcl\|Query_554976:5188-5645 | hRSV/B/England/194840343/2019\|EPI_ISL_1834175\|2019-11-25 |
| >lcl\|Query_554948:5123-5580 | hRSV/B/Argentina/BA-HNRG-409/2018\|EPI_ISL_15067698\|2018-06-11 |
| >lcl\|Query_554728:5171-5628 | hRSV/B/England/195100338/2019\|EPI_ISL_1647557\|2019-12-13 |
| >lcl\|Query_554725:5172-5629 | hRSV/B/England/195100167/2019\|EPI_ISL_1647554\|2019-12-13 |
| >lcl\|Query_554714:5175-5632 | hRSV/B/England/194960690/2019\|EPI_ISL_1647543\|2019-12-04 |
| >lcl\|Query_554702:5170-5627 | hRSV/B/England/194860182/2019\|EPI_ISL_1647531\|2019-11-26 |
| >lcl\|Query_554700:5162-5619 | hRSV/B/England/194840350/2019\|EPI_ISL_1647529\|2019-11-25 |
| >lcl\|Query_554695:5189-5646 | hRSV/B/England/194760337/2019\|EPI_ISL_1647524\|2019-11-20 |
| >lcl\|Query_554692:5171-5628 | hRSV/B/England/194740388/2019\|EPI_ISL_1647521\|2019-11-19 |
| >lcl\|Query_554689:5194-5651 | hRSV/B/England/194680410/2019\|EPI_ISL_1647518\|2019-11-13 |
| >lcl\|Query_554641:5123-5580 | hRSV/B/Argentina/HNRG-409/2018\|EPI_ISL_15055333\|2018-06-11 |
| >lcl\|Query_108038:5146-5603 | hRSV/B/Australia/VIC-RCH001/2017\|EPI_ISL_1760409\|2017-05-17 |
| >lcl\|Query_108031:5148-5605 | hRSV/B/Australia/VIC-RCH004/2017\|EPI_ISL_1760402\|2017-05-23 |
| >lcl\|Query_107996:5175-5632 | hRSV/B/Morocco/1513-19/2019\|EPI_ISL_15120790\|2019-02-04 |
| >lcl\|Query_107896:5195-5652 | hRSV/B/England/184580320/2018\|EPI_ISL_732356\|2018-11-07 |
| >lcl\|Query_107893:5189-5646 | hRSV/B/England/180400390/2018\|EPI_ISL_732353\|2018-01-19 |
| >lcl\|Query_108063:5147-5604 | hRSV/B/Australia/VIC-RCH128/2018\|EPI_ISL_1760434\|2018-07-09 |
| >lcl\|Query_108053:5147-5604 | hRSV/B/Australia/VIC-RCH184/2018\|EPI_ISL_1760424\|2018-10-15 |
| >lcl\|Query_107889:5190-5647 | hRSV/B/England/174880237/2017\|EPI_ISL_732349\|2017-11-30 |
| >lcl\|Query_108165:5175-5632 | hRSV/B/Morocco/909-19/2019\|EPI_ISL_15120796\|2019-01-31 |
| >lcl\|Query_107998:5175-5632 | hRSV/B/Morocco/1144-19/2019\|EPI_ISL_15120798\|2019-02-04 |
| >lcl\|Query_107993:5175-5632 | hRSV/B/Morocco/709-19/2019\|EPI_ISL_15120787\|2019-01-29 |
| >lcl\|Query_107997:5175-5632 | hRSV/B/Morocco/911-19/2019\|EPI_ISL_15120797\|2019-01-31 |
| >lcl\|Query_554751:5191-5648 | hRSV/B/England/200220906/2020\|EPI_ISL_1647580\|2020-01-06 |
| >lcl\|Query_554980:5191-5648 | hRSV/B/England/200480207/2019\|EPI_ISL_1834179\|2019-12-24 |
| >lcl\|Query_554871:5170-5627 | hRSV/B/Australia/VIC-VIDRL003/2018\|EPI_ISL_10914987\|2018-07-16 |
| >lcl\|Query_554783:5147-5604 | hRSV/B/Australia/VIC-RCH071/2018\|EPI_ISL_1760399\|2018-04-29 |
| >lcl\|Query_554752:5191-5648 | hRSV/B/England/200220919/2020\|EPI_ISL_1647581\|2020-01-06 |
| >lcl\|Query_554734:5191-5648 | hRSV/B/England/195160225/2019\|EPI_ISL_1647563\|2019-12-18 |
| >lcl\|Query_554733:5194-5651 | hRSV/B/England/195140716/2019\|EPI_ISL_1647562\|2019-12-16 |
| >lcl\|Query_554732:5186-5643 | hRSV/B/England/195120244/2019\|EPI_ISL_1647561\|2019-12-10 |
| >lcl\|Query_554719:5177-5634 | hRSV/B/England/195000600/2019\|EPI_ISL_1647548\|2019-12-06 |
| >lcl\|Query_554718:5189-5646 | hRSV/B/England/194980232/2019\|EPI_ISL_1647547\|2019-12-04 |
| >lcl\|Query_554715:5172-5629 | hRSV/B/England/194960700/2019\|EPI_ISL_1647544\|2019-12-03 |
| >lcl\|Query_554707:5171-5628 | hRSV/B/England/194860469/2019\|EPI_ISL_1647536\|2019-11-26 |
| >lcl\|Query_554703:5171-5628 | hRSV/B/England/194860188/2019\|EPI_ISL_1647532\|2019-11-25 |
| >lcl\|Query_554698:5189-5646 | hRSV/B/England/194800196/2019\|EPI_ISL_1647527\|2019-11-22 |
| >lcl\|Query_554688:5174-5631 | hRSV/B/England/194680352/2019\|EPI_ISL_1647517\|2019-11-13 |
| >lcl\|Query_554683:5161-5618 | hRSV/B/England/194640442/2019\|EPI_ISL_1647512\|2019-11-11 |
| >lcl\|Query_108037:5193-5650 | hRSV/B/Australia/VIC-RCH152/2019\|EPI_ISL_1760408\|2019-08-19 |
| >lcl\|Query_108026:5148-5605 | hRSV/B/Australia/VIC-RCH054/2019\|EPI_ISL_1653997\|2019-08-12 |
| >lcl\|Query_108020:5194-5651 | hRSV/B/England/194600336/2019\|EPI_ISL_1647508\|2019-11-07 |
| >lcl\|Query_107901:5194-5651 | hRSV/B/England/184780456/2018\|EPI_ISL_732365\|2018-11-22 |
| >lcl\|Query_554974:5194-5651 | hRSV/B/England/RE20000104/2020\|EPI_ISL_1647600\|2020-01-30 |
| >lcl\|Query_554968:5179-5636 | hRSV/B/England/194420545/2019\|EPI_ISL_1647498\|2019-10-28 |
| >lcl\|Query_554748:5191-5648 | hRSV/B/England/200200201/2020\|EPI_ISL_1647577\|2020-01-02 |
| >lcl\|Query_554741:5189-5646 | hRSV/B/England/195200839/2019\|EPI_ISL_1647570\|2019-12-20 |
| >lcl\|Query_108032:5147-5604 | hRSV/B/Australia/VIC-RCH070/2018\|EPI_ISL_1760403\|2018-04-28 |
| >lcl\|Query_554962:5187-5644 | hRSV/B/England/194360327/2019\|EPI_ISL_1647492\|2019-10-22 |
| >lcl\|Query_554960:5176-5633 | hRSV/B/England/194220417/2019\|EPI_ISL_1647490\|2019-10-14 |
| >lcl\|Query_108017:5165-5622 | hRSV/B/England/194560636/2019\|EPI_ISL_1647505\|2019-11-04 |
| >lcl\|Query_554947:5124-5581 | hRSV/B/Argentina/BA-HNRG-408/2018\|EPI_ISL_15067697\|2018-06-07 |
| >lcl\|Query_554670:5124-5581 | hRSV/B/Argentina/BA-HNRG-429/2018\|EPI_ISL_15067713\|2018-08-06 |
| >lcl\|Query_554656:5124-5581 | hRSV/B/Argentina/HNRG-429/2018\|EPI_ISL_15055348\|2018-08-06 |
| >lcl\|Query_554640:5124-5581 | hRSV/B/Argentina/HNRG-408/2018\|EPI_ISL_15055332\|2018-06-07 |
| >lcl\|Query_554920:5175-5632 | hRSV/B/Philippines/99061/2019\|EPI_ISL_12970404\|2019-07-09 |
| >lcl\|Query_554919:5175-5632 | hRSV/B/Philippines/99059/2019\|EPI_ISL_12970402\|2019-03-15 |
| >lcl\|Query_554829:5096-5553 | hRSV/B/Russia/Novosibirsk-293/2019\|EPI_ISL_2575615\|2019-04-03 |
| >lcl\|Query_554828:5096-5553 | hRSV/B/Russia/Novosibirsk-5019/2019\|EPI_ISL_2575606\|2019-01-20 |
| >lcl\|Query_554723:5179-5636 | hRSV/B/England/195060570/2019\|EPI_ISL_1647552\|2019-12-10 |
| >lcl\|Query_554712:5177-5634 | hRSV/B/England/194940751/2019\|EPI_ISL_1647541\|2019-12-03 |
| >lcl\|Query_107855:5169-5626 | hRSV/B/Australia/M236822/2019\|EPI_ISL_2839450\|2019-07-27 |
| >lcl\|Query_108073:5149-5606 | hRSV/B/Australia/VIC-RCH189/2018\|EPI_ISL_1760444\|2018-12-10 |
| >lcl\|Query_108024:5148-5605 | hRSV/B/Australia/VIC-RCH052/2019\|EPI_ISL_1653995\|2019-06-03 |
| >lcl\|Query_108023:5148-5605 | hRSV/B/Australia/VIC-RCH051/2019\|EPI_ISL_1653994\|2019-06-02 |
| >lcl\|Query_554645:5125-5582 | hRSV/B/Argentina/HNRG-417/2018\|EPI_ISL_15055337\|2018-07-03 |
| >lcl\|Query_108069:5194-5651 | hRSV/B/Australia/VIC-RCH070/2017\|EPI_ISL_1760440\|2017-07-20 |
| >lcl\|Query_108068:5194-5651 | hRSV/B/Australia/VIC-RCH039/2018\|EPI_ISL_1760439\|2018-04-01 |
| >lcl\|Query_108029:5192-5649 | hRSV/B/Australia/VIC-RCH092/2017\|EPI_ISL_1760400\|2017-07-30 |
| >lcl\|Query_108010:5193-5650 | hRSV/B/Mozambique/MAP-INS019/2018\|EPI_ISL_1647488\|2018-08-01 |
| >lcl\|Query_107954:5125-5582 | hRSV/B/Argentina/BA-HNRG-417/2018\|EPI_ISL_15067702\|2018-07-03 |
| >lcl\|Query_107860:5170-5627 | hRSV/B/Australia/M262451/2019\|EPI_ISL_2839456\|2019-09-12 |
| >lcl\|Query_108162:5175-5632 | hRSV/B/Morocco/1515-19/2019\|EPI_ISL_15120793\|2019-02-04 |
| >lcl\|Query_554917:5128-5585 | hRSV/B/South_Africa/NICD-R09811/2021\|EPI_ISL_12529660\|2021-07-01 |
| >lcl\|Query_554875:5139-5596 | hRSV/B/South_Africa/NICD-R10143/2021\|EPI_ISL_11055804\|2021-07-07 |
| >lcl\|Query_107911:5142-5599 | hRSV/B/South_Africa/NICD-R03230/2021\|EPI_ISL_11055787\|2021-02-25 |
| >lcl\|Query_107906:5125-5582 | hRSV/B/South_Africa/NICD-R00259/2021\|EPI_ISL_11055770\|2021-01-05 |
| >lcl\|Query_554874:5142-5599 | hRSV/B/South_Africa/NICD-R09797/2021\|EPI_ISL_11055803\|2021-06-28 |
| >lcl\|Query_554737:5190-5647 | hRSV/B/England/195200234/2019\|EPI_ISL_1647566\|2019-12-19 |
| >lcl\|Query_554724:5192-5649 | hRSV/B/England/195080613/2019\|EPI_ISL_1647553\|2019-12-11 |
| >lcl\|Query_554706:5178-5635 | hRSV/B/England/194860199/2019\|EPI_ISL_1647535\|2019-11-25 |
| >lcl\|Query_554705:5190-5647 | hRSV/B/England/194860198/2019\|EPI_ISL_1647534\|2019-11-25 |
| >lcl\|Query_554647:5126-5583 | hRSV/B/Argentina/HNRG-420/2018\|EPI_ISL_15055339\|2018-07-11 |
| >lcl\|Query_107956:5126-5583 | hRSV/B/Argentina/BA-HNRG-420/2018\|EPI_ISL_15067704\|2018-07-11 |
| >lcl\|Query_554667:5126-5583 | hRSV/B/Argentina/BA-HNRG-426/2018\|EPI_ISL_15067710\|2018-07-23 |
| >lcl\|Query_554653:5126-5583 | hRSV/B/Argentina/HNRG-426/2018\|EPI_ISL_15055345\|2018-07-23 |
| >lcl\|Query_554644:5126-5583 | hRSV/B/Argentina/HNRG-412/2018\|EPI_ISL_15055336\|2018-06-15 |
| >lcl\|Query_554649:5126-5583 | hRSV/B/Argentina/HNRG-422/2018\|EPI_ISL_15055341\|2018-07-13 |
| >lcl\|Query_108028:5153-5610 | hRSV/B/Australia/VIC-RCH056/2019\|EPI_ISL_1653999\|2019-03-04 |
| >lcl\|Query_107953:5126-5583 | hRSV/B/Argentina/BA-HNRG-412/2018\|EPI_ISL_15067701\|2018-06-15 |
| >lcl\|Query_107958:5126-5583 | hRSV/B/Argentina/BA-HNRG-422/2018\|EPI_ISL_15067706\|2018-07-13 |
| >lcl\|Query_554945:5126-5583 | hRSV/B/Argentina/BA-HNRG-406/2018\|EPI_ISL_15067695\|2018-05-11 |
| >lcl\|Query_554638:5126-5583 | hRSV/B/Argentina/HNRG-406/2018\|EPI_ISL_15055330\|2018-05-11 |
| >lcl\|Query_554651:5126-5583 | hRSV/B/Argentina/HNRG-424/2018\|EPI_ISL_15055343\|2018-07-20 |
| >lcl\|Query_554643:5126-5583 | hRSV/B/Argentina/HNRG-411/2018\|EPI_ISL_15055335\|2018-06-15 |
| >lcl\|Query_107960:5126-5583 | hRSV/B/Argentina/BA-HNRG-424/2018\|EPI_ISL_15067708\|2018-07-20 |
| >lcl\|Query_107952:5126-5583 | hRSV/B/Argentina/BA-HNRG-411/2018\|EPI_ISL_15067700\|2018-06-15 |
| >lcl\|Query_554913:5140-5597 | hRSV/B/South_Africa/NICD-R03655/2021\|EPI_ISL_12529655\|2021-03-09 |
| >lcl\|Query_554722:5166-5623 | hRSV/B/England/195040641/2019\|EPI_ISL_1647551\|2019-12-09 |
| >lcl\|Query_554686:5190-5647 | hRSV/B/England/194660606/2019\|EPI_ISL_1647515\|2019-11-13 |
| >lcl\|Query_554710:5194-5651 | hRSV/B/England/194900383/2019\|EPI_ISL_1647539\|2019-11-28 |
| >lcl\|Query_554762:5194-5651 | hRSV/B/England/RE19003142/2019\|EPI_ISL_1647591\|2019-11-09 |
| >lcl\|Query_554743:5189-5646 | hRSV/B/England/200100176/2019\|EPI_ISL_1647572\|2019-12-24 |
| >lcl\|Query_107882:5157-5614 | hRSV/B/China/HB-61255/2021\|EPI_ISL_6314658\|2021-04-02 |
| >lcl\|Query_107878:5158-5615 | hRSV/B/China/HB-58777/2021\|EPI_ISL_6314471\|2021-03-23 |
| >lcl\|Query_554982:5195-5652 | hRSV/B/England/200521044/2020\|EPI_ISL_1834181\|2020-01-27 |
| >lcl\|Query_554729:5171-5628 | hRSV/B/England/195100341/2019\|EPI_ISL_1647558\|2019-12-13 |
| >lcl\|Query_108012:5171-5628 | hRSV/B/England/194480341/2019\|EPI_ISL_1647500\|2019-10-30 |
| >lcl\|Query_554983:5190-5647 | hRSV/B/England/200540572/2020\|EPI_ISL_1834182\|2020-01-27 |
| >lcl\|Query_554977:5195-5652 | hRSV/B/England/195160452/2019\|EPI_ISL_1834176\|2019-12-17 |
| >lcl\|Query_108018:5190-5647 | hRSV/B/England/194560645/2019\|EPI_ISL_1647506\|2019-11-05 |
| >lcl\|Query_554756:5186-5643 | hRSV/B/England/200440221/2020\|EPI_ISL_1647585\|2020-01-20 |
| >lcl\|Query_108021:5190-5647 | hRSV/B/England/194640426/2019\|EPI_ISL_1647509\|2019-11-11 |
| >lcl\|Query_554726:5176-5633 | hRSV/B/England/195100171/2019\|EPI_ISL_1647555\|2019-12-10 |
| >lcl\|Query_554757:5179-5636 | hRSV/B/England/200560685/2020\|EPI_ISL_1647586\|2020-01-28 |
| >lcl\|Query_107856:5170-5627 | hRSV/B/Australia/WM0742913/2020\|EPI_ISL_2839452\|2020-03-14 |
| >lcl\|Query_554966:5185-5642 | hRSV/B/England/194400258/2019\|EPI_ISL_1647496\|2019-10-24 |
| >lcl\|Query_554963:5190-5647 | hRSV/B/England/194360328/2019\|EPI_ISL_1647493\|2019-10-22 |
| >lcl\|Query_554720:5194-5651 | hRSV/B/England/195020397/2019\|EPI_ISL_1647549\|2019-12-09 |
| >lcl\|Query_554768:5194-5651 | hRSV/B/Australia/VIC-RCH077/2019\|EPI_ISL_1760384\|2019-06-02 |
| >lcl\|Query_554969:5178-5635 | hRSV/B/England/194480340/2019\|EPI_ISL_1647499\|2019-10-30 |
| >lcl\|Query_554965:5178-5635 | hRSV/B/England/194400257/2019\|EPI_ISL_1647495\|2019-10-25 |
| >lcl\|Query_107950:5143-5600 | hRSV/B/South_Africa/NICD-R05811/2022\|EPI_ISL_14769858\|2022-05-20 |
| >lcl\|Query_107949:5143-5600 | hRSV/B/South_Africa/NICD-R05802/2022\|EPI_ISL_14769856\|2022-05-20 |
| >lcl\|Query_107948:5143-5600 | hRSV/B/South_Africa/NICD-R05801/2022\|EPI_ISL_14769855\|2022-05-20 |
| >lcl\|Query_107947:5143-5600 | hRSV/B/South_Africa/NICD-R05800/2022\|EPI_ISL_14769854\|2022-05-20 |
| >lcl\|Query_107945:5143-5600 | hRSV/B/South_Africa/NICD-R05781/2022\|EPI_ISL_14769849\|2022-05-20 |
| >lcl\|Query_107907:5143-5600 | hRSV/B/South_Africa/NICD-R00422/2021\|EPI_ISL_11055771\|2021-01-13 |
| >lcl\|Query_107918:5143-5600 | hRSV/B/South_Africa/NICD-R08586/2021\|EPI_ISL_11055798\|2021-06-08 |
| >lcl\|Query_554883:5166-5623 | hRSV/B/Australia/QLD-RBWH052/2021\|EPI_ISL_11817035\|2021-02-01 |
| >lcl\|Query_554904:5167-5624 | hRSV/B/Australia/QLD-RBWH279/2021\|EPI_ISL_11817079\|2021-04-02 |
| >lcl\|Query_554903:5166-5623 | hRSV/B/Australia/QLD-RBWH275/2021\|EPI_ISL_11817077\|2021-03-30 |
| >lcl\|Query_554901:5166-5623 | hRSV/B/Australia/QLD-RBWH248/2021\|EPI_ISL_11817073\|2021-03-21 |
| >lcl\|Query_554896:5166-5623 | hRSV/B/Australia/QLD-RBWH180/2021\|EPI_ISL_11817060\|2021-03-03 |
| >lcl\|Query_554894:5166-5623 | hRSV/B/Australia/QLD-RBWH176/2021\|EPI_ISL_11817058\|2021-03-02 |
| >lcl\|Query_554888:5166-5623 | hRSV/B/Australia/QLD-RBWH087/2021\|EPI_ISL_11817048\|2021-02-11 |
| >lcl\|Query_554887:5166-5623 | hRSV/B/Australia/QLD-RBWH084/2021\|EPI_ISL_11817047\|2021-02-10 |
| >lcl\|Query_554886:5166-5623 | hRSV/B/Australia/QLD-RBWH071/2021\|EPI_ISL_11817044\|2021-02-08 |
| >lcl\|Query_554885:5166-5623 | hRSV/B/Australia/QLD-RBWH062/2021\|EPI_ISL_11817040\|2021-02-05 |
| >lcl\|Query_554882:5166-5623 | hRSV/B/Australia/QLD-RBWH050/2021\|EPI_ISL_11817034\|2021-01-31 |
| >lcl\|Query_554878:5166-5623 | hRSV/B/Australia/QLD-RBWH016/2021\|EPI_ISL_11817022\|2021-01-14 |
| >lcl\|Query_554877:5166-5623 | hRSV/B/Australia/QLD-RBWH006/2021\|EPI_ISL_11817018\|2021-01-06 |
| >lcl\|Query_107932:5186-5643 | hRSV/B/Australia/VIC-MMC024/2021\|EPI_ISL_11817133\|2021-01-04 |
| >lcl\|Query_107922:5186-5643 | hRSV/B/Australia/VIC-MMC040/2020\|EPI_ISL_11817123\|2020-12-23 |
| >lcl\|Query_107921:5184-5641 | hRSV/B/Australia/VIC-MMC037/2020\|EPI_ISL_11817122\|2020-12-22 |
| >lcl\|Query_554905:5166-5623 | hRSV/B/Australia/QLD-RBWH284/2021\|EPI_ISL_11817081\|2021-04-05 |
| >lcl\|Query_554900:5166-5623 | hRSV/B/Australia/QLD-RBWH223/2021\|EPI_ISL_11817068\|2021-03-13 |
| >lcl\|Query_554921:5175-5632 | hRSV/B/Philippines/99063/2019\|EPI_ISL_12970406\|2019-08-10 |
| >lcl\|Query_554857:5190-5647 | hRSV/B/Egypt/C-CPHL004/2019\|EPI_ISL_6208721\|2019-01-27 |
| >lcl\|Query_107859:5170-5627 | hRSV/B/Australia/M236813/2019\|EPI_ISL_2839455\|2019-07-27 |
| >lcl\|Query_554648:5125-5582 | hRSV/B/Argentina/HNRG-421/2018\|EPI_ISL_15055340\|2018-07-12 |
| >lcl\|Query_107957:5125-5582 | hRSV/B/Argentina/BA-HNRG-421/2018\|EPI_ISL_15067705\|2018-07-12 |
| >lcl\|Query_108022:5147-5604 | hRSV/B/Australia/VIC-RCH050/2019\|EPI_ISL_1653993\|2019-05-19 |
| >lcl\|Query_108049:5148-5605 | hRSV/B/Australia/VIC-RCH096/2018\|EPI_ISL_1760420\|2018-06-16 |
| >lcl\|Query_108056:5146-5603 | hRSV/B/Australia/VIC-RCH049/2018\|EPI_ISL_1760427\|2018-04-11 |
| >lcl\|Query_108047:5192-5649 | hRSV/B/Australia/VIC-RCH134/2017\|EPI_ISL_1760418\|2017-09-14 |
| >lcl\|Query_107861:5170-5627 | hRSV/B/Australia/WM0634962/2020\|EPI_ISL_2839457\|2020-03-03 |
| >lcl\|Query_554867:5150-5607 | hRSV/B/Argentina/BA-HNRG-303/2017\|EPI_ISL_1074086\|2017-04-11 |
| >lcl\|Query_554753:5178-5635 | hRSV/B/England/200260605/2020\|EPI_ISL_1647582\|2020-01-08 |
| >lcl\|Query_554687:5189-5646 | hRSV/B/England/194660618/2019\|EPI_ISL_1647516\|2019-11-12 |
| >lcl\|Query_108158:5195-5652 | hRSV/B/England/185160298/2018\|EPI_ISL_732371\|2018-12-17 |
| >lcl\|Query_107903:5194-5651 | hRSV/B/England/185080309/2018\|EPI_ISL_732367\|2018-12-10 |
| >lcl\|Query_108027:5148-5605 | hRSV/B/Australia/VIC-RCH055/2019\|EPI_ISL_1653998\|2019-08-18 |
| >lcl\|Query_108060:5145-5602 | hRSV/B/Australia/VIC-RCH099/2017\|EPI_ISL_1760431\|2017-08-01 |
| >lcl\|Query_108035:5192-5649 | hRSV/B/Australia/VIC-RCH094/2018\|EPI_ISL_1760406\|2018-06-18 |
| >lcl\|Query_108034:5148-5605 | hRSV/B/Australia/VIC-RCH079/2018\|EPI_ISL_1760405\|2018-05-28 |
| >lcl\|Query_108052:5148-5605 | hRSV/B/Australia/VIC-RCH141/2017\|EPI_ISL_1760423\|2017-09-05 |
| >lcl\|Query_554853:5178-5635 | hRSV/B/Kenya/KHDSS-31-02-CHA/2017\|EPI_ISL_2585054\|2017-05-02 |
| >lcl\|Query_554852:5146-5603 | hRSV/B/Kenya/KHDSS-30-02-CHA/2017\|EPI_ISL_2585053\|2017-05-10 |
| >lcl\|Query_108122:5179-5636 | hRSV/B/Kenya/KHDSS-50-08-JAR/2017\|EPI_ISL_2584981\|2017-03-08 |
| >lcl\|Query_108118:5178-5635 | hRSV/B/Kenya/KHDSS-29-02-CHA/2017\|EPI_ISL_2584975\|2017-04-06 |
| >lcl\|Query_108111:5173-5630 | hRSV/B/Kenya/KHDSS-158-11-MAV/2017\|EPI_ISL_2584965\|2017-04-11 |
| >lcl\|Query_108110:5163-5620 | hRSV/B/Kenya/KHDSS-157-11-MAV/2017\|EPI_ISL_2584964\|2017-04-11 |
| >lcl\|Query_108108:5179-5636 | hRSV/B/Kenya/KHDSS-147-21-MAV/2017\|EPI_ISL_2584961\|2017-03-14 |
| >lcl\|Query_108076:5178-5635 | hRSV/B/Kenya/KHDSS-46-01-JAR/2017\|EPI_ISL_2584718\|2017-03-01 |
| >lcl\|Query_107814:5178-5635 | hRSV/B/Kenya/KHDSS-172-16-MAV/2017\|EPI_ISL_2585108\|2017-05-16 |
| >lcl\|Query_107813:5178-5635 | hRSV/B/Kenya/KHDSS-170-12-MAV/2017\|EPI_ISL_2585107\|2017-05-03 |
| >lcl\|Query_107812:5145-5602 | hRSV/B/Kenya/KHDSS-168-02-MAV/2017\|EPI_ISL_2585106\|2017-05-02 |
| >lcl\|Query_107811:5178-5635 | hRSV/B/Kenya/KHDSS-167-02-MAV/2017\|EPI_ISL_2585105\|2017-05-02 |
| >lcl\|Query_107810:5178-5635 | hRSV/B/Kenya/KHDSS-166-02-MAV/2017\|EPI_ISL_2585104\|2017-04-28 |
| >lcl\|Query_107809:5188-5645 | hRSV/B/Kenya/KHDSS-164-28-MAV/2017\|EPI_ISL_2585103\|2017-04-21 |
| >lcl\|Query_108100:5178-5635 | hRSV/B/Kenya/KHDSS-59-11-JAR/2017\|EPI_ISL_2584932\|2017-04-07 |
| >lcl\|Query_108098:5178-5635 | hRSV/B/Kenya/KHDSS-289-06-SOK/2017\|EPI_ISL_2584930\|2017-04-06 |
| >lcl\|Query_554844:5178-5635 | hRSV/B/Kenya/KHDSS-254-11-PIN/2017\|EPI_ISL_2585040\|2017-04-13 |
| >lcl\|Query_554838:5183-5640 | hRSV/B/Kenya/KHDSS-18-03-CHA/2017\|EPI_ISL_2585029\|2017-03-03 |
| >lcl\|Query_554835:5163-5620 | hRSV/B/Kenya/KHDSS-159-11-MAV/2017\|EPI_ISL_2585026\|2017-04-11 |
| >lcl\|Query_554834:5186-5643 | hRSV/B/Kenya/KHDSS-149-30-MAV/2017\|EPI_ISL_2585025\|2017-03-21 |
| >lcl\|Query_107978:5175-5632 | hRSV/B/Morocco/1122-17/2017\|EPI_ISL_15120757\|2017-02-28 |
| >lcl\|Query_107849:5171-5628 | hRSV/B/Kenya/KHDSS-55-30-JAR/2017\|EPI_ISL_2585182\|2017-03-29 |
| >lcl\|Query_107831:5178-5635 | hRSV/B/Kenya/KHDSS-56-30-JAR/2017\|EPI_ISL_2585131\|2017-03-30 |
| >lcl\|Query_107820:5178-5635 | hRSV/B/Kenya/KHDSS-227-05-NGE/2017\|EPI_ISL_2585116\|2017-04-04 |
| >lcl\|Query_107806:5183-5640 | hRSV/B/Kenya/KHDSS-150-30-MAV/2017\|EPI_ISL_2585100\|2017-03-30 |
| >lcl\|Query_107795:5181-5638 | hRSV/B/Kenya/KHDSS-140-17-MAV/2017\|EPI_ISL_2584881\|2017-01-09 |
| >lcl\|Query_107781:5146-5603 | hRSV/B/Kenya/KHDSS-152-06-MAV/2017\|EPI_ISL_2584489\|2017-04-05 |
| >lcl\|Query_108079:5146-5603 | hRSV/B/Kenya/KHDSS-217-07-NGE/2017\|EPI_ISL_2584774\|2017-03-02 |
| >lcl\|Query_108078:5183-5640 | hRSV/B/Kenya/KHDSS-215-24-NGE/2017\|EPI_ISL_2584773\|2017-01-24 |
| >lcl\|Query_107990:5174-5631 | hRSV/B/Morocco/1330-17/2017\|EPI_ISL_15120769\|2017-03-21 |
| >lcl\|Query_107804:5145-5602 | hRSV/B/Kenya/KHDSS-112-16-MAT/2017\|EPI_ISL_2585096\|2017-03-16 |
| >lcl\|Query_107982:5175-5632 | hRSV/B/Morocco/767-17/2017\|EPI_ISL_15120761\|2017-01-15 |
| >lcl\|Query_107977:5175-5632 | hRSV/B/Morocco/1119-17/2017\|EPI_ISL_15120756\|2017-02-28 |
| >lcl\|Query_107850:5178-5635 | hRSV/B/Kenya/KHDSS-85-10-JUN/2017\|EPI_ISL_2585185\|2017-04-10 |
| >lcl\|Query_107841:5146-5603 | hRSV/B/Kenya/KHDSS-197-06-MTO/2017\|EPI_ISL_2585164\|2017-03-28 |
| >lcl\|Query_107986:5175-5632 | hRSV/B/Morocco/1118-17/2017\|EPI_ISL_15120765\|2017-02-28 |
| >lcl\|Query_554776:5148-5605 | hRSV/B/Australia/VIC-RCH016/2018\|EPI_ISL_1760392\|2018-02-04 |
| >lcl\|Query_107984:5175-5632 | hRSV/B/Morocco/1017-17/2017\|EPI_ISL_15120763\|2017-02-12 |
| >lcl\|Query_107980:5175-5632 | hRSV/B/Morocco/1144-17/2017\|EPI_ISL_15120759\|2017-03-01 |
| >lcl\|Query_107979:5175-5632 | hRSV/B/Morocco/1136-17/2017\|EPI_ISL_15120758\|2017-02-27 |
| >lcl\|Query_107976:5175-5632 | hRSV/B/Morocco/1112-17/2017\|EPI_ISL_15120755\|2017-02-17 |
| >lcl\|Query_107972:5175-5632 | hRSV/B/Morocco/692-17/2017\|EPI_ISL_15120751\|2017-01-19 |
| >lcl\|Query_107968:5176-5633 | hRSV/B/Morocco/683-17/2017\|EPI_ISL_15120747\|2017-01-19 |
| >lcl\|Query_107989:5175-5632 | hRSV/B/Morocco/1327-17/2017\|EPI_ISL_15120768\|2017-03-20 |
| >lcl\|Query_107971:5174-5631 | hRSV/B/Morocco/617-17/2017\|EPI_ISL_15120750\|2017-01-14 |
| >lcl\|Query_107891:5195-5652 | hRSV/B/England/180160857/2018\|EPI_ISL_732351\|2018-01-02 |
| >lcl\|Query_107966:5175-5632 | hRSV/B/Morocco/641-17/2017\|EPI_ISL_15120744\|2017-01-18 |
| >lcl\|Query_107962:5175-5632 | hRSV/B/Morocco/387-17/2017\|EPI_ISL_15120737\|2017-01-02 |
| >lcl\|Query_107975:5175-5632 | hRSV/B/Morocco/1030-17/2017\|EPI_ISL_15120754\|2017-02-12 |
| >lcl\|Query_107987:5176-5633 | hRSV/B/Morocco/1379-17/2017\|EPI_ISL_15120766\|2017-03-28 |
| >lcl\|Query_108059:5146-5603 | hRSV/B/Australia/VIC-RCH074/2018\|EPI_ISL_1760430\|2018-05-12 |
| >lcl\|Query_107898:5196-5653 | hRSV/B/England/184740644/2018\|EPI_ISL_732362\|2018-11-20 |
| >lcl\|Query_108044:5148-5605 | hRSV/B/Australia/VIC-RCH090/2018\|EPI_ISL_1760415\|2018-06-11 |
| >lcl\|Query_108036:5148-5605 | hRSV/B/Australia/VIC-RCH117/2018\|EPI_ISL_1760407\|2018-07-01 |
| >lcl\|Query_107890:5188-5645 | hRSV/B/England/175000153/2017\|EPI_ISL_732350\|2017-12-06 |
| >lcl\|Query_107999:5175-5632 | hRSV/B/Morocco/2093-19/2019\|EPI_ISL_15120799\|2019-03-03 |
| >lcl\|Query_108163:5175-5632 | hRSV/B/Morocco/1562-19/2019\|EPI_ISL_15120794\|2019-02-08 |
| >lcl\|Query_107940:5152-5609 | hRSV/B/Vietnam/SARI02CC18136/2018\|EPI_ISL_12544917\|2018-08-31 |
| >lcl\|Query_108071:5150-5607 | hRSV/B/Australia/VIC-RCH109/2018\|EPI_ISL_1760442\|2018-07-02 |
| >lcl\|Query_108070:5150-5607 | hRSV/B/Australia/VIC-RCH108/2018\|EPI_ISL_1760441\|2018-07-02 |
| >lcl\|Query_554923:5175-5632 | hRSV/B/Philippines/99081/2019\|EPI_ISL_12970424\|2019-12-22 |
| >lcl\|Query_554858:5157-5614 | hRSV/B/China/HB-32993/2020\|EPI_ISL_6268594\|2020-11-19 |
| >lcl\|Query_554769:5193-5650 | hRSV/B/Australia/VIC-RCH130/2018\|EPI_ISL_1760385\|2018-07-15 |
| >lcl\|Query_107939:5147-5604 | hRSV/B/Vietnam/SARI02CC17015/2017\|EPI_ISL_12544916\|2017-01-28 |
| >lcl\|Query_107883:5158-5615 | hRSV/B/China/HB-61918/2021\|EPI_ISL_6314659\|2021-04-05 |
| >lcl\|Query_554990:5146-5603 | hRSV/B/Australia/VIC-RCH021/2020\|EPI_ISL_2156819\|2020-02-29 |
| >lcl\|Query_554758:5193-5650 | hRSV/B/England/200600042/2020\|EPI_ISL_1647587\|2020-01-28 |
| >lcl\|Query_554744:5180-5637 | hRSV/B/England/200100191/2019\|EPI_ISL_1647573\|2019-12-27 |
| >lcl\|Query_554967:5174-5631 | hRSV/B/England/194420415/2019\|EPI_ISL_1647497\|2019-10-28 |
| >lcl\|Query_108133:5193-5650 | hRSV/B/Australia/7527H/2019\|EPI_ISL_2839376\|2020-07-21 |
| >lcl\|Query_108025:5147-5604 | hRSV/B/Australia/VIC-RCH053/2019\|EPI_ISL_1653996\|2019-08-12 |
| >lcl\|Query_107858:5170-5627 | hRSV/B/Australia/WM0636501/2020\|EPI_ISL_2839454\|2020-03-03 |
| >lcl\|Query_554997:5096-5553 | hRSV/B/Russia/Novosibirsk-242/2019\|EPI_ISL_2575571\|2019-03-14 |
| >lcl\|Query_107868:5142-5599 | hRSV/B/Cote_d'Ivoire/IPCI-010/2019\|EPI_ISL_5522628\|2019-08-19 |
| >lcl\|Query_108161:5175-5632 | hRSV/B/Morocco/1542-19/2019\|EPI_ISL_15120792\|2019-02-06 |
| >lcl\|Query_108160:5175-5632 | hRSV/B/Morocco/739-19/2019\|EPI_ISL_15120791\|2019-01-28 |
| >lcl\|Query_554672:5124-5581 | hRSV/B/Argentina/BA-HNRG-434/2019\|EPI_ISL_15067715\|2019-06-07 |
| >lcl\|Query_554658:5124-5581 | hRSV/B/Argentina/HNRG-434/2019\|EPI_ISL_15055350\|2019-06-07 |
| >lcl\|Query_554961:5189-5646 | hRSV/B/England/194300233/2019\|EPI_ISL_1647491\|2019-10-17 |
| >lcl\|Query_107994:5176-5633 | hRSV/B/Morocco/1489-19/2019\|EPI_ISL_15120788\|2019-02-07 |
| >lcl\|Query_554652:5126-5583 | hRSV/B/Argentina/HNRG-425/2018\|EPI_ISL_15055344\|2018-07-20 |
| >lcl\|Query_107961:5126-5583 | hRSV/B/Argentina/BA-HNRG-425/2018\|EPI_ISL_15067709\|2018-07-20 |
| >lcl\|Query_554946:5125-5582 | hRSV/B/Argentina/BA-HNRG-407/2018\|EPI_ISL_15067696\|2018-06-05 |
| >lcl\|Query_554639:5125-5582 | hRSV/B/Argentina/HNRG-407/2018\|EPI_ISL_15055331\|2018-06-05 |
| >lcl\|Query_107871:5157-5614 | hRSV/B/China/HB-56392/2021\|EPI_ISL_6314464\|2021-03-13 |
| >lcl\|Query_108145:5158-5615 | hRSV/B/China/HB-37875/2020\|EPI_ISL_6268611\|2020-12-13 |
| >lcl\|Query_108062:5145-5602 | hRSV/B/Australia/VIC-RCH008/2017\|EPI_ISL_1760433\|2017-05-29 |
| >lcl\|Query_108050:5146-5603 | hRSV/B/Australia/VIC-RCH108/2017\|EPI_ISL_1760421\|2017-08-11 |
| >lcl\|Query_554944:5124-5581 | hRSV/B/Argentina/BA-HNRG-405/2018\|EPI_ISL_15067694\|2018-05-11 |
| >lcl\|Query_554669:5124-5581 | hRSV/B/Argentina/BA-HNRG-428/2018\|EPI_ISL_15067712\|2018-07-24 |
| >lcl\|Query_554655:5124-5581 | hRSV/B/Argentina/HNRG-428/2018\|EPI_ISL_15055347\|2018-07-24 |
| >lcl\|Query_554650:5124-5581 | hRSV/B/Argentina/HNRG-423/2018\|EPI_ISL_15055342\|2018-07-16 |
| >lcl\|Query_554646:5124-5581 | hRSV/B/Argentina/HNRG-418/2018\|EPI_ISL_15055338\|2018-07-06 |
| >lcl\|Query_554637:5124-5581 | hRSV/B/Argentina/HNRG-405/2018\|EPI_ISL_15055329\|2018-05-11 |
| >lcl\|Query_107959:5124-5581 | hRSV/B/Argentina/BA-HNRG-423/2018\|EPI_ISL_15067707\|2018-07-16 |
| >lcl\|Query_107955:5124-5581 | hRSV/B/Argentina/BA-HNRG-418/2018\|EPI_ISL_15067703\|2018-07-06 |
| >lcl\|Query_554782:5147-5604 | hRSV/B/Australia/VIC-RCH065/2017\|EPI_ISL_1760398\|2017-07-18 |
| >lcl\|Query_554779:5192-5649 | hRSV/B/Australia/VIC-RCH117/2017\|EPI_ISL_1760395\|2017-08-18 |
| >lcl\|Query_108030:5146-5603 | hRSV/B/Australia/VIC-RCH111/2018\|EPI_ISL_1760401\|2018-06-30 |
| >lcl\|Query_107900:5182-5639 | hRSV/B/England/184760324/2018\|EPI_ISL_732364\|2018-11-19 |
| >lcl\|Query_107899:5194-5651 | hRSV/B/England/184760323/2018\|EPI_ISL_732363\|2018-11-19 |
| >lcl\|Query_554994:5096-5553 | hRSV/B/Russia/Novosibirsk-50Hp/2018\|EPI_ISL_2575500\|2018-03-19 |
| >lcl\|Query_554827:5096-5553 | hRSV/B/Russia/Novosibirsk-50Hl/2018\|EPI_ISL_2575499\|2018-03-19 |
| >lcl\|Query_554951:5194-5651 | hRSV/B/Mozambique/MAP-INS002/2017\|EPI_ISL_1647471\|2017-03-20 |
| >lcl\|Query_108051:5192-5649 | hRSV/B/Australia/VIC-RCH122/2017\|EPI_ISL_1760422\|2017-08-22 |
| >lcl\|Query_554908:5188-5645 | hRSV/B/Australia/VIC-RCH111/2021\|EPI_ISL_11817087\|2021-03-13 |
| >lcl\|Query_554906:5169-5626 | hRSV/B/Australia/QLD-RBWH289/2021\|EPI_ISL_11817084\|2021-04-08 |
| >lcl\|Query_554907:5169-5626 | hRSV/B/Australia/QLD-RBWH290/2021\|EPI_ISL_11817085\|2021-04-09 |
| >lcl\|Query_554899:5170-5627 | hRSV/B/Australia/QLD-RBWH218/2021\|EPI_ISL_11817067\|2021-03-11 |
| >lcl\|Query_554898:5169-5626 | hRSV/B/Australia/QLD-RBWH193/2021\|EPI_ISL_11817064\|2021-03-07 |
| >lcl\|Query_554895:5170-5627 | hRSV/B/Australia/QLD-RBWH178/2021\|EPI_ISL_11817059\|2021-03-03 |
| >lcl\|Query_554892:5169-5626 | hRSV/B/Australia/QLD-RBWH152/2021\|EPI_ISL_11817055\|2021-02-23 |
| >lcl\|Query_554891:5169-5626 | hRSV/B/Australia/QLD-RBWH150/2021\|EPI_ISL_11817054\|2021-02-23 |
| >lcl\|Query_554890:5169-5626 | hRSV/B/Australia/QLD-RBWH129/2021\|EPI_ISL_11817052\|2021-02-17 |
| >lcl\|Query_554884:5170-5627 | hRSV/B/Australia/QLD-RBWH055/2021\|EPI_ISL_11817037\|2021-02-03 |
| >lcl\|Query_554881:5169-5626 | hRSV/B/Australia/QLD-RBWH047/2021\|EPI_ISL_11817033\|2021-01-31 |
| >lcl\|Query_554880:5169-5626 | hRSV/B/Australia/QLD-RBWH042/2021\|EPI_ISL_11817031\|2021-01-30 |
| >lcl\|Query_554789:5140-5597 | hRSV/B/Australia/VIC-RCH060/2021\|EPI_ISL_2543853\|2021-02-22 |
| >lcl\|Query_554787:5139-5596 | hRSV/B/Australia/VIC-RCH057/2021\|EPI_ISL_2543851\|2021-03-06 |
| >lcl\|Query_108132:5169-5626 | hRSV/B/Australia/WM3181190/2020\|EPI_ISL_2839374\|2020-11-13 |
| >lcl\|Query_107928:5189-5646 | hRSV/B/Australia/VIC-MMC058/2020\|EPI_ISL_11817129\|2020-12-31 |
| >lcl\|Query_554902:5169-5626 | hRSV/B/Australia/QLD-RBWH253/2021\|EPI_ISL_11817074\|2021-03-23 |
| >lcl\|Query_554785:5139-5596 | hRSV/B/Australia/VIC-RCH025/2021\|EPI_ISL_2543849\|2021-02-20 |
| >lcl\|Query_554910:5142-5599 | hRSV/B/Australia/VIC-RCH074/2021\|EPI_ISL_11817093\|2021-03-26 |
| >lcl\|Query_554992:5139-5596 | hRSV/B/Australia/VIC-RCH059/2021\|EPI_ISL_2543763\|2021-02-22 |
| >lcl\|Query_554911:5187-5644 | hRSV/B/Australia/VIC-RCH086/2021\|EPI_ISL_11817098\|2021-04-04 |
| >lcl\|Query_554879:5169-5626 | hRSV/B/Australia/QLD-RBWH040/2021\|EPI_ISL_11817029\|2021-01-29 |
| >lcl\|Query_554788:5139-5596 | hRSV/B/Australia/VIC-RCH058/2021\|EPI_ISL_2543852\|2021-02-21 |
| >lcl\|Query_554786:5139-5596 | hRSV/B/Australia/VIC-RCH053/2021\|EPI_ISL_2543850\|2021-03-07 |
| >lcl\|Query_554784:5139-5596 | hRSV/B/Australia/VIC-MMC022/2020\|EPI_ISL_2543848\|2020-12-21 |
| >lcl\|Query_554621:5189-5646 | hRSV/B/Australia/VIC-MMC013/2020\|EPI_ISL_11817119\|2020-12-18 |
| >lcl\|Query_554620:5187-5644 | hRSV/B/Australia/VIC-MMC012/2020\|EPI_ISL_11817118\|2020-12-18 |
| >lcl\|Query_554619:5189-5646 | hRSV/B/Australia/VIC-MMC011/2020\|EPI_ISL_11817117\|2020-12-17 |
| >lcl\|Query_554618:5189-5646 | hRSV/B/Australia/VIC-MMC009/2020\|EPI_ISL_11817116\|2020-12-16 |
| >lcl\|Query_554617:5189-5646 | hRSV/B/Australia/VIC-MMC008/2020\|EPI_ISL_11817115\|2020-12-15 |
| >lcl\|Query_554616:5190-5647 | hRSV/B/Australia/VIC-MMC007/2020\|EPI_ISL_11817114\|2020-12-15 |
| >lcl\|Query_554615:5189-5646 | hRSV/B/Australia/VIC-MMC066/2020\|EPI_ISL_11817113\|2020-12-13 |
| >lcl\|Query_554614:5189-5646 | hRSV/B/Australia/VIC-MMC003/2020\|EPI_ISL_11817112\|2020-12-11 |
| >lcl\|Query_554613:5189-5646 | hRSV/B/Australia/VIC-MMC065/2020\|EPI_ISL_11817111\|2020-12-10 |
| >lcl\|Query_554612:5189-5646 | hRSV/B/Australia/VIC-MMC001/2020\|EPI_ISL_11817110\|2020-12-10 |
| >lcl\|Query_107938:5191-5648 | hRSV/B/Australia/VIC-MMC052/2021\|EPI_ISL_11817139\|2021-01-09 |
| >lcl\|Query_107937:5189-5646 | hRSV/B/Australia/VIC-MMC048/2021\|EPI_ISL_11817138\|2021-01-09 |
| >lcl\|Query_107936:5188-5645 | hRSV/B/Australia/VIC-MMC046/2021\|EPI_ISL_11817137\|2021-01-08 |
| >lcl\|Query_107935:5189-5646 | hRSV/B/Australia/VIC-MMC042/2021\|EPI_ISL_11817136\|2021-01-07 |
| >lcl\|Query_107934:5189-5646 | hRSV/B/Australia/VIC-MMC038/2021\|EPI_ISL_11817135\|2021-01-06 |
| >lcl\|Query_107933:5191-5648 | hRSV/B/Australia/VIC-MMC025/2021\|EPI_ISL_11817134\|2021-01-04 |
| >lcl\|Query_107931:5189-5646 | hRSV/B/Australia/VIC-MMC017/2021\|EPI_ISL_11817132\|2021-01-02 |
| >lcl\|Query_107930:5191-5648 | hRSV/B/Australia/VIC-MMC016/2021\|EPI_ISL_11817131\|2021-01-03 |
| >lcl\|Query_107929:5191-5648 | hRSV/B/Australia/VIC-MMC006/2021\|EPI_ISL_11817130\|2021-01-02 |
| >lcl\|Query_107927:5189-5646 | hRSV/B/Australia/VIC-MMC051/2020\|EPI_ISL_11817128\|2020-12-29 |
| >lcl\|Query_107926:5189-5646 | hRSV/B/Australia/VIC-MMC047/2020\|EPI_ISL_11817127\|2020-12-28 |
| >lcl\|Query_107924:5189-5646 | hRSV/B/Australia/VIC-MMC043/2020\|EPI_ISL_11817125\|2020-12-25 |
| >lcl\|Query_107923:5187-5644 | hRSV/B/Australia/VIC-MMC042/2020\|EPI_ISL_11817124\|2020-12-24 |
| >lcl\|Query_107920:5191-5648 | hRSV/B/Australia/VIC-MMC036/2020\|EPI_ISL_11817121\|2020-12-22 |
| >lcl\|Query_107919:5189-5646 | hRSV/B/Australia/VIC-MMC020/2020\|EPI_ISL_11817120\|2020-12-20 |
| >lcl\|Query_107862:5133-5590 | hRSV/B/Australia/VIC-RCH023/2021\|EPI_ISL_2989828\|2021-02-15 |
| >lcl\|Query_554909:5187-5644 | hRSV/B/Australia/VIC-RCH062/2021\|EPI_ISL_11817089\|2021-03-14 |
| >lcl\|Query_554987:5146-5603 | hRSV/B/Australia/VIC-RCH010/2020\|EPI_ISL_2156816\|2020-01-19 |
| >lcl\|Query_107851:5169-5626 | hRSV/B/Australia/WM3181191/2020\|EPI_ISL_2839416\|2020-11-13 |
| >lcl\|Query_554897:5172-5629 | hRSV/B/Australia/QLD-RBWH192/2021\|EPI_ISL_11817063\|2021-03-07 |
| >lcl\|Query_554893:5169-5626 | hRSV/B/Australia/QLD-RBWH159/2021\|EPI_ISL_11817057\|2021-02-25 |
| >lcl\|Query_554872:5143-5600 | hRSV/B/South_Africa/NICD-R09064/2021\|EPI_ISL_11055801\|2021-06-21 |
| >lcl\|Query_107915:5140-5597 | hRSV/B/South_Africa/NICD-R08478/2021\|EPI_ISL_11055795\|2021-06-07 |
| >lcl\|Query_554912:5124-5581 | hRSV/B/South_Africa/NICD-R03603/2021\|EPI_ISL_12529654\|2021-03-10 |
| >lcl\|Query_107951:5143-5600 | hRSV/B/South_Africa/NICD-R05858/2022\|EPI_ISL_14769860\|2022-05-23 |
| >lcl\|Query_107910:5134-5591 | hRSV/B/South_Africa/NICD-R01840/2022\|EPI_ISL_11055783\|2022-02-15 |
| >lcl\|Query_108129:5173-5630 | hRSV/B/Australia/WM0663762/2020\|EPI_ISL_2839185\|2020-03-06 |
| >lcl\|Query_554864:5143-5600 | hRSV/B/South_Africa/NICD-R06224/2019\|EPI_ISL_9003920\|2019-05-31 |
| >lcl\|Query_107884:5157-5614 | hRSV/B/China/HB-61984/2021\|EPI_ISL_6314660\|2021-04-05 |
| >lcl\|Query_108152:5156-5613 | hRSV/B/China/HB-46486/2021\|EPI_ISL_6268623\|2021-01-23 |
| >lcl\|Query_107887:5156-5613 | hRSV/B/China/HB-62717/2021\|EPI_ISL_6314663\|2021-04-08 |
| >lcl\|Query_107872:5157-5614 | hRSV/B/China/HB-57214/2021\|EPI_ISL_6314465\|2021-03-16 |
| >lcl\|Query_107881:5157-5614 | hRSV/B/China/HB-61266/2021\|EPI_ISL_6314642\|2021-04-02 |
| >lcl\|Query_107877:5157-5614 | hRSV/B/China/HB-58492/2021\|EPI_ISL_6314470\|2021-03-22 |
| >lcl\|Query_107873:5157-5614 | hRSV/B/China/HB-57844/2021\|EPI_ISL_6314466\|2021-03-19 |
| >lcl\|Query_554735:5195-5652 | hRSV/B/England/195180588/2019\|EPI_ISL_1647564\|2019-12-18 |
| >lcl\|Query_554954:5192-5649 | hRSV/B/Mozambique/MAP-INS003/2018\|EPI_ISL_1647474\|2018-03-12 |
| >lcl\|Query_108015:5192-5649 | hRSV/B/England/194540435/2019\|EPI_ISL_1647503\|2019-11-04 |
| >lcl\|Query_107880:5157-5614 | hRSV/B/China/HB-60725/2021\|EPI_ISL_6314641\|2021-03-31 |
| >lcl\|Query_554996:5095-5552 | hRSV/B/Russia/Novosibirsk-66Hp/2018\|EPI_ISL_2575547\|2018-03-22 |
| >lcl\|Query_554995:5095-5552 | hRSV/B/Russia/Novosibirsk-66Hl/2018\|EPI_ISL_2575546\|2018-03-22 |
| >lcl\|Query_107894:5190-5647 | hRSV/B/England/180440410/2018\|EPI_ISL_732354\|2018-01-18 |
| >lcl\|Query_107864:5193-5650 | hRSV/B/England/410/2018\|EPI_ISL_412867\|2018-01-01 |
| >lcl\|Query_107995:5176-5633 | hRSV/B/Morocco/1721-19/2019\|EPI_ISL_15120789\|2019-02-09 |
| >lcl\|Query_554611:5195-5652 | hRSV/B/England/185080311/2018\|EPI_ISL_11428314\|2018-12-07 |
| >lcl\|Query_554759:5178-5635 | hRSV/B/England/201061200/2020\|EPI_ISL_1647588\|2020-03-03 |
| >lcl\|Query_107888:5195-5652 | hRSV/B/England/174680283/2017\|EPI_ISL_732343\|2017-11-15 |
| >lcl\|Query_554761:5185-5642 | hRSV/B/England/201200474/2020\|EPI_ISL_1647590\|2020-03-12 |
| >lcl\|Query_107886:5157-5614 | hRSV/B/China/HB-62566/2021\|EPI_ISL_6314662\|2021-04-07 |
| >lcl\|Query_554668:5124-5581 | hRSV/B/Argentina/BA-HNRG-427/2018\|EPI_ISL_15067711\|2018-07-23 |
| >lcl\|Query_554654:5124-5581 | hRSV/B/Argentina/HNRG-427/2018\|EPI_ISL_15055346\|2018-07-23 |
| >lcl\|Query_108042:5181-5638 | hRSV/B/Australia/VIC-RCH030/2018\|EPI_ISL_1760413\|2018-03-09 |
| >lcl\|Query_554814:5148-5605 | hRSV/B/Mongolia/NCCD019/2018\|EPI_ISL_2543878\|2018-01-29 |
| >lcl\|Query_554803:5139-5596 | hRSV/B/Mongolia/NCCD010/2018\|EPI_ISL_2543867\|2018-01-15 |
| >lcl\|Query_554800:5139-5596 | hRSV/B/Mongolia/NCCD005/2018\|EPI_ISL_2543864\|2018-01-10 |
| >lcl\|Query_554824:5141-5598 | hRSV/B/Mongolia/NCCD037/2017\|EPI_ISL_2543888\|2017-12-28 |
| >lcl\|Query_554818:5148-5605 | hRSV/B/Mongolia/NCCD025/2018\|EPI_ISL_2543882\|2018-02-05 |
| >lcl\|Query_554823:5138-5595 | hRSV/B/Mongolia/NCCD035/2018\|EPI_ISL_2543887\|2018-02-20 |
| >lcl\|Query_108033:5147-5604 | hRSV/B/Australia/VIC-RCH037/2018\|EPI_ISL_1760404\|2018-03-28 |
| >lcl\|Query_107991:5176-5633 | hRSV/B/Morocco/841-17/2017\|EPI_ISL_15120779\|2017-01-19 |
| >lcl\|Query_108041:5148-5605 | hRSV/B/Australia/VIC-RCH015/2018\|EPI_ISL_1760412\|2018-02-02 |
| >lcl\|Query_107944:5140-5597 | hRSV/B/South_Africa/NICD-R05717/2022\|EPI_ISL_14769847\|2022-05-19 |
| >lcl\|Query_554993:5152-5609 | hRSV/B/Japan/take2/2018\|EPI_ISL_2575367\|2018-08-29 |
| >lcl\|Query_108067:5194-5651 | hRSV/B/Australia/VIC-RCH141/2019\|EPI_ISL_1760438\|2019-08-04 |
| >lcl\|Query_554978:5190-5647 | hRSV/B/England/195220376/2019\|EPI_ISL_1834177\|2019-12-19 |
| >lcl\|Query_554975:5189-5646 | hRSV/B/England/194520782/2019\|EPI_ISL_1834174\|2019-11-04 |
| >lcl\|Query_554755:5190-5647 | hRSV/B/England/200280390/2019\|EPI_ISL_1647584\|2019-12-24 |
| >lcl\|Query_554736:5178-5635 | hRSV/B/England/195200138/2019\|EPI_ISL_1647565\|2019-12-19 |
| >lcl\|Query_554708:5191-5648 | hRSV/B/England/194880285/2019\|EPI_ISL_1647537\|2019-11-28 |
| >lcl\|Query_108150:5157-5614 | hRSV/B/China/HB-46126/2021\|EPI_ISL_6268621\|2021-01-21 |
| >lcl\|Query_107852:5169-5626 | hRSV/B/Australia/WM0322845/2020\|EPI_ISL_2839417\|2020-02-01 |
| >lcl\|Query_108151:5157-5614 | hRSV/B/China/HB-46289/2021\|EPI_ISL_6268622\|2021-01-22 |
| >lcl\|Query_107876:5159-5616 | hRSV/B/China/HB-58581/2021\|EPI_ISL_6314469\|2021-03-22 |
| >lcl\|Query_108157:5156-5613 | hRSV/B/China/HB-60337/2021\|EPI_ISL_6314585\|2021-03-29 |
| >lcl\|Query_108153:5157-5614 | hRSV/B/China/HB-49998/2021\|EPI_ISL_6268625\|2021-02-11 |
| >lcl\|Query_107874:5155-5612 | hRSV/B/China/HB-58211/2021\|EPI_ISL_6314467\|2021-03-21 |
| >lcl\|Query_554826:5187-5644 | hRSV/B/Thailand/Ayuttaya-TNIC007/2020\|EPI_ISL_2543890\|2020-11-04 |
| >lcl\|Query_108141:5156-5613 | hRSV/B/China/HB-31344/2020\|EPI_ISL_6268456\|2020-11-11 |
| >lcl\|Query_108147:5157-5614 | hRSV/B/China/HB-38946/2020\|EPI_ISL_6268617\|2020-12-18 |
| >lcl\|Query_554863:5157-5614 | hRSV/B/China/HB-37292/2020\|EPI_ISL_6268599\|2020-12-10 |
| >lcl\|Query_108131:5171-5628 | hRSV/B/Australia/WM1603138/2020\|EPI_ISL_2839368\|2020-06-08 |
| >lcl\|Query_107870:5157-5614 | hRSV/B/China/HB-51132/2021\|EPI_ISL_6314463\|2021-02-19 |
| >lcl\|Query_108140:5158-5615 | hRSV/B/China/HB-31271/2020\|EPI_ISL_6268455\|2020-11-11 |
| >lcl\|Query_107885:5156-5613 | hRSV/B/China/HB-62086/2021\|EPI_ISL_6314661\|2021-04-06 |
| >lcl\|Query_554716:5175-5632 | hRSV/B/England/194960704/2019\|EPI_ISL_1647545\|2019-12-02 |
| >lcl\|Query_108154:5157-5614 | hRSV/B/China/HB-50073/2021\|EPI_ISL_6268626\|2021-02-12 |
| >lcl\|Query_107875:5157-5614 | hRSV/B/China/HB-58490/2021\|EPI_ISL_6314468\|2021-03-22 |
| >lcl\|Query_554914:5134-5591 | hRSV/B/South_Africa/NICD-R03687/2021\|EPI_ISL_12529656\|2021-03-09 |
| >lcl\|Query_107917:5141-5597 | hRSV/B/South_Africa/NICD-R08585/2021\|EPI_ISL_11055797\|2021-06-08 |
| >lcl\|Query_107912:5140-5596 | hRSV/B/South_Africa/NICD-R08243/2021\|EPI_ISL_11055792\|2021-06-01 |
| >lcl\|Query_554873:5144-5601 | hRSV/B/South_Africa/NICD-R09258/2021\|EPI_ISL_11055802\|2021-06-22 |
| >lcl\|Query_107914:5140-5597 | hRSV/B/South_Africa/NICD-R08392/2021\|EPI_ISL_11055794\|2021-06-04 |
| >lcl\|Query_108061:5146-5603 | hRSV/B/Australia/VIC-RCH198/2018\|EPI_ISL_1760432\|2018-12-28 |
| >lcl\|Query_108039:5146-5603 | hRSV/B/Australia/VIC-RCH069/2017\|EPI_ISL_1760410\|2017-07-20 |
| >lcl\|Query_107857:5187-5644 | hRSV/B/Australia/9391H/2019\|EPI_ISL_2839453\|2020-08-10 |
| >lcl\|Query_554810:5140-5597 | hRSV/B/Mongolia/NCCD017/2018\|EPI_ISL_2543874\|2018-01-26 |
| >lcl\|Query_554801:5143-5600 | hRSV/B/Mongolia/NCCD009/2018\|EPI_ISL_2543865\|2018-01-15 |
| >lcl\|Query_554861:5153-5610 | hRSV/B/China/HB-36125/2020\|EPI_ISL_6268597\|2020-12-04 |
| >lcl\|Query_108142:5153-5610 | hRSV/B/China/HB-31505/2020\|EPI_ISL_6268457\|2020-11-12 |
| >lcl\|Query_108072:5194-5651 | hRSV/B/Australia/VIC-RCH114/2019\|EPI_ISL_1760443\|2019-07-08 |
| >lcl\|Query_554750:5178-5635 | hRSV/B/England/200220399/2020\|EPI_ISL_1647579\|2020-01-02 |
| >lcl\|Query_108139:5157-5614 | hRSV/B/China/HB-28820/2020\|EPI_ISL_6268437\|2020-10-31 |
| >lcl\|Query_108148:5157-5614 | hRSV/B/China/HB-39147/2020\|EPI_ISL_6268618\|2020-12-19 |
| >lcl\|Query_108143:5157-5614 | hRSV/B/China/HB-37781/2020\|EPI_ISL_6268606\|2020-12-12 |
| >lcl\|Query_554738:5197-5654 | hRSV/B/England/195200237/2019\|EPI_ISL_1647567\|2019-12-19 |
| >lcl\|Query_108046:5148-5605 | hRSV/B/Australia/VIC-RCH102/2018\|EPI_ISL_1760417\|2018-06-25 |
| >lcl\|Query_108054:5147-5604 | hRSV/B/Australia/VIC-RCH056/2018\|EPI_ISL_1760425\|2018-04-17 |
| >lcl\|Query_108064:5146-5603 | hRSV/B/Australia/VIC-RCH032/2018\|EPI_ISL_1760435\|2018-03-14 |
| >lcl\|Query_108048:5147-5604 | hRSV/B/Australia/VIC-RCH026/2018\|EPI_ISL_1760419\|2018-03-05 |
| >lcl\|Query_107905:5140-5597 | hRSV/B/South_Africa/NICD-R00035/2021\|EPI_ISL_11055769\|2021-01-04 |
| >lcl\|Query_554740:5173-5630 | hRSV/B/England/195200246/2019\|EPI_ISL_1647569\|2019-12-18 |
| >lcl\|Query_554696:5165-5622 | hRSV/B/England/194760455/2019\|EPI_ISL_1647525\|2019-11-18 |
| >lcl\|Query_554764:5192-5649 | hRSV/B/England/RE19003237/2019\|EPI_ISL_1647593\|2019-12-10 |
| >lcl\|Query_554986:5194-5651 | hRSV/B/Australia/VIC-RCH005/2017\|EPI_ISL_2156815\|2017-05-24 |
| >lcl\|Query_108043:5147-5604 | hRSV/B/Australia/VIC-RCH035/2017\|EPI_ISL_1760414\|2017-06-30 |
| >lcl\|Query_107904:5194-5651 | hRSV/B/England/184860361/2018\|EPI_ISL_10954009\|2018-11-26 |
| >lcl\|Query_107897:5196-5653 | hRSV/B/England/184620747/2018\|EPI_ISL_732358\|2018-11-09 |
| >lcl\|Query_554989:5147-5603 | hRSV/B/Australia/VIC-RCH016/2020\|EPI_ISL_2156818\|2020-02-22 |
| >lcl\|Query_554973:5159-5616 | hRSV/B/England/RE20000019/2020\|EPI_ISL_1647599\|2020-01-02 |
| >lcl\|Query_554690:5190-5647 | hRSV/B/England/194700041/2019\|EPI_ISL_1647519\|2019-10-25 |
| >lcl\|Query_107902:5195-5652 | hRSV/B/England/184900130/2018\|EPI_ISL_732366\|2018-11-30 |
| >lcl\|Query_554979:5194-5651 | hRSV/B/England/200360377/2020\|EPI_ISL_1834178\|2020-01-10 |
| >lcl\|Query_554747:5193-5650 | hRSV/B/England/200181093/2019\|EPI_ISL_1647576\|2019-12-31 |
| >lcl\|Query_554713:5183-5640 | hRSV/B/England/194960635/2019\|EPI_ISL_1647542\|2019-12-02 |
| >lcl\|Query_554709:5186-5643 | hRSV/B/England/194880847/2019\|EPI_ISL_1647538\|2019-11-27 |
| >lcl\|Query_108146:5157-5614 | hRSV/B/China/HB-38291/2020\|EPI_ISL_6268612\|2020-12-15 |
| >lcl\|Query_554859:5156-5613 | hRSV/B/China/HB-33784/2020\|EPI_ISL_6268595\|2020-11-23 |
| >lcl\|Query_108155:5157-5614 | hRSV/B/China/HB-50071/2021\|EPI_ISL_6268627\|2021-02-12 |
| >lcl\|Query_554860:5157-5614 | hRSV/B/China/HB-35522/2020\|EPI_ISL_6268596\|2020-12-01 |
| >lcl\|Query_107854:5169-5626 | hRSV/B/Australia/WM0782450/2020\|EPI_ISL_2839449\|2020-03-18 |
| >lcl\|Query_107908:5128-5585 | hRSV/B/South_Africa/NICD-R00448/2022\|EPI_ISL_11055772\|2022-01-11 |
| >lcl\|Query_554610:5151-5609 | hRSV/B/Thailand/AY-1743/2020\|EPI_ISL_11050303\|2020-11-05 |
| >lcl\|Query_108144:5157-5614 | hRSV/B/China/HB-37714/2020\|EPI_ISL_6268607\|2020-12-12 |
| >lcl\|Query_554775:5147-5604 | hRSV/B/Australia/VIC-RCH003/2018\|EPI_ISL_1760391\|2018-01-11 |
| >lcl\|Query_554790:5140-5597 | hRSV/B/Cote_d'Ivoire/IPCI-003/2017\|EPI_ISL_2543854\|2017-04-21 |
| >lcl\|Query_108136:5189-5646 | hRSV/B/Cote_d'Ivoire/IPCI-035/2017\|EPI_ISL_6174134\|2017-10-16 |
| >lcl\|Query_107869:5187-5644 | hRSV/B/Cote_d'Ivoire/IPCI-012/2018\|EPI_ISL_5522629\|2018-10-30 |
| >lcl\|Query_554792:5139-5596 | hRSV/B/Cote_d'Ivoire/IPCI-046/2017\|EPI_ISL_2543856\|2017-10-23 |
| >lcl\|Query_554791:5139-5596 | hRSV/B/Cote_d'Ivoire/IPCI-036/2017\|EPI_ISL_2543855\|2017-10-15 |
| >lcl\|Query_107865:5142-5599 | hRSV/B/Cote_d'Ivoire/IPCI-018/2017\|EPI_ISL_5522625\|2017-09-06 |
| >lcl\|Query_107866:5187-5644 | hRSV/B/Cote_d'Ivoire/IPCI-021/2017\|EPI_ISL_5522626\|2017-09-03 |
| >lcl\|Query_108057:5147-5604 | hRSV/B/Australia/VIC-RCH029/2018\|EPI_ISL_1760428\|2018-03-09 |
| >lcl\|Query_108055:5146-5603 | hRSV/B/Australia/VIC-RCH001/2018\|EPI_ISL_1760426\|2018-01-10 |
| >lcl\|Query_108058:5149-5606 | hRSV/B/Australia/VIC-RCH057/2018\|EPI_ISL_1760429\|2018-04-17 |
| >lcl\|Query_107983:5171-5628 | hRSV/B/Morocco/794-17/2017\|EPI_ISL_15120762\|2017-01-20 |
| >lcl\|Query_108156:5164-5621 | hRSV/B/China/HB-50619/2021\|EPI_ISL_6268628\|2021-02-17 |
| >lcl\|Query_108149:5156-5615 | hRSV/B/China/HB-44330/2021\|EPI_ISL_6268620\|2021-01-13 |
| >lcl\|Query_107879:5157-5615 | hRSV/B/China/HB-60417/2021\|EPI_ISL_6314640\|2021-03-30 |
| >lcl\|Query_107909:5129-5586 | hRSV/B/South_Africa/NICD-R01430/2022\|EPI_ISL_11055778\|2022-02-07 |
| >lcl\|Query_554773:5148-5605 | hRSV/B/Australia/VIC-RCH050/2018\|EPI_ISL_1760389\|2018-04-11 |
| >lcl\|Query_554772:5148-5605 | hRSV/B/Australia/VIC-RCH028/2018\|EPI_ISL_1760388\|2018-03-09 |
| >lcl\|Query_554749:5195-5614 | hRSV/B/England/200220388/2020\|EPI_ISL_1647578\|2020-01-03 |
| >lcl\|Query_107867:5188-5644 | hRSV/B/Cote_d'Ivoire/IPCI-030/2017\|EPI_ISL_5522627\|2017-09-19 |
| >lcl\|Query_554822:5139-5596 | hRSV/B/Mongolia/NCCD034/2018\|EPI_ISL_2543886\|2018-02-14 |
| >lcl\|Query_554809:5139-5596 | hRSV/B/Mongolia/NCCD016/2018\|EPI_ISL_2543873\|2018-01-24 |
| >lcl\|Query_554876:5140-5423 | hRSV/B/South_Africa/NICD-R10250/2021\|EPI_ISL_11055805\|2021-07-09 |
| >lcl\|Query_107916:5140-5411 | hRSV/B/South_Africa/NICD-R08483/2021\|EPI_ISL_11055796\|2021-06-07 |
| >lcl\|Query_554774:5193-5650 | hRSV/B/Australia/VIC-RCH037/2017\|EPI_ISL_1760390\|2017-07-03 |
| >lcl\|Query_554991:5193-5650 | hRSV/B/Australia/VIC-RCH078/2017\|EPI_ISL_2156820\|2017-07-24 |
| >lcl\|Query_108130:5169-5626 | hRSV/B/Australia/WM0421262/2020\|EPI_ISL_2839366\|2020-02-11 |
| >lcl\|Query_107853:5169-5626 | hRSV/B/Australia/WM0653812/2020\|EPI_ISL_2839442\|2020-03-05 |
| >lcl\|Query_554949:5125-5581 | hRSV/B/Argentina/BA-HNRG-410/2018\|EPI_ISL_15067699\|2018-06-14 |
| >lcl\|Query_554642:5125-5581 | hRSV/B/Argentina/HNRG-410/2018\|EPI_ISL_15055334\|2018-06-14 |
| >lcl\|Query_554862:5154-5611 | hRSV/B/China/HB-36915/2020\|EPI_ISL_6268598\|2020-12-08 |
| >lcl\|Query_554609:5151-5608 | hRSV/B/Argentina/BA-HNRG-313/2017\|EPI_ISL_1074192\|2017-05-11 |
| >lcl\|Query_107974:5176-5633 | hRSV/B/Morocco/991-17/2017\|EPI_ISL_15120753\|2017-02-10 |
| >lcl\|Query_107981:5176-5633 | hRSV/B/Morocco/759-17/2017\|EPI_ISL_15120760\|2017-01-17 |
| >lcl\|Query_107964:5176-5633 | hRSV/B/Morocco/584-17/2017\|EPI_ISL_15120741\|2017-01-13 |
| >lcl\|Query_554926:5127-5221 | hRSV/B/Israel/3966/2021\|EPI_ISL_13231414\|2021-06-28 |
| >lcl\|Query_108001:5067-5133 | hRSV/B/North_Macedonia/IPH-MKD-220/2022\|EPI_ISL_15601574\|2022-10-18 |
| >lcl\|Query_554926:5283-5393 | hRSV/B/Israel/3966/2021\|EPI_ISL_13231414\|2021-06-28 |
| >lcl\|Query_108001:5186-5468 | hRSV/B/North_Macedonia/IPH-MKD-220/2022\|EPI_ISL_15601574\|2022-10-18 |
| >lcl\|Query_554631:5318-5528 | hRSV/B/Spain/CHUVI-19471353/2021\|EPI_ISL_14084077\|2021-12-18 |
| >lcl\|Query_554626:5366-5576 | hRSV/B/Spain/CHUVI-19454915/2021\|EPI_ISL_14084072\|2021-12-01 |
| >lcl\|Query_554630:5364-5574 | hRSV/B/Spain/CHUVI-19470906/2021\|EPI_ISL_14084076\|2021-12-17 |
| >lcl\|Query_107974:5439-5502 | hRSV/B/Morocco/991-17/2017\|EPI_ISL_15120753\|2017-02-10 |
| >lcl\|Query_107981:5439-5502 | hRSV/B/Morocco/759-17/2017\|EPI_ISL_15120760\|2017-01-17 |
| >lcl\|Query_107964:5439-5502 | hRSV/B/Morocco/584-17/2017\|EPI_ISL_15120741\|2017-01-13 |

**Table S9**: Analysis of the sequencing process

|  | **RSV A** | **RSV B** |
| --- | --- | --- |
| Number of RT-qPCR positive wastewater samples selected for external and semi-nested PCR | 281 | 233 |
| Number of samples with expected band from the tape station analysis | 159 | 101 |
| Total number of consensus sequences obtained | 10 | 14 |
| Mean Ct value ± 1 standard deviation (SD) of wastewater samples selected for external and semi-nested PCR | 37.15 ± 0.89 | 37.18 ± 0.93 |
| Mean Ct value ± 1 standard deviation (SD) of wastewater samples that were successfully sequenced | 37.23 ± 0.86 | 36.75 ± 0.76 |

**Table S10:** The mean read depth per base ± 1 SD for each consensus sequence.

| **RSV Serotype** | **Sample ID** | **Mean read depth per base ± 1 SD** |
| --- | --- | --- |
| A | AMH280921 | 1217 ± 789 |
|  | ATM191021 | 2831 ± 1417 |
|  | BEL200921 | 3107 ± 1904 |
|  | BNB280921 | 2504 ± 1699 |
|  | CUL200921 | 862 ± 777 |
|  | DNG270921 | 2172 ± 1090 |
|  | NDN280921 | 1479 ± 1294 |
|  | WTH310821 | 7292 ± 1696 |
|  | NDN281122 | 1817 ± 962 |
|  | WTH071122 | 7081 ± 1917 |
|  | V22034499 | 4740 ± 3482 |
|  | V22034533 | 3969 ± 3498 |
| B | BNB041121 | 1186 ± 496 |
|  | LIS210921 | 6575 ± 2398 |
|  | AMH031022 | 6111 ± 2398 |
|  | ATM101022 | 5942 ± 2726 |
|  | BNB290922 | 6820 ± 2283 |
|  | CRG290922 | 6037 ± 2480 |
|  | CRG311022 | 6867 ± 2215 |
|  | DNG311022 | 6639 ± 2252 |
|  | LIS101022 | 6535 ± 2336 |
|  | LIS150822 | 5974 ± 2295 |
|  | LIS311022 | 6767 ± 2243 |
|  | NCT120922 | 5176 ± 2071 |
|  | NCT311022 | 6840 ± 2229 |
|  | NDN031022 | 5841 ± 2783 |
|  | V22033750 | 6210 ± 2186 |
|  | V22034570 | 5263 ± 2136 |
|  | V22034901 | 1855 ± 819 |
|  | V22035143 | 6927 ± 2209 |

**Table S11**: Details on the RSV A and B consensus sequences derived from wastewater and clinical samples.

| **Sample ID** | **Sample type** | **Year** | **WWTW** | **RSV Subtype** | **NextClade G clade** | **Assigned Lineage in this study** |
| --- | --- | --- | --- | --- | --- | --- |
| AMH280921 | Wastewater | 2021 | Armagh | A | GA2.3.5 | RSV.A.NI_1.2 |
| ATM191021 | Wastewater | 2021 | Antrim | A | GA2.3.5 | RSV.A.NI_1.2 |
| BEL200921 | Wastewater | 2021 | Belfast | A | GA2.3.5 | RSV.A.NI_2 |
| BNB280921 | Wastewater | 2021 | Banbridge | A | GA2.3.5 | RSV.A.NI_1.1 |
| CUL200921 | Wastewater | 2021 | Culmore | A | GA2.3.5 | RSV.A.NI_3 |
| DNG270921 | Wastewater | 2021 | Dungannon | A | GA2.3.5 | RSV.A.NI_1.1 |
| NDN280921 | Wastewater | 2021 | North Down | A | GA2.3.5 | RSV.A.NI_1.2 |
| WTH310821 | Wastewater | 2021 | Whitehouse | A | GA2.3.5 | RSV.A.NI_2 |
| BNB041121 | Wastewater | 2021 | Banbridge | B | GB5.0.5a | RSV.B.NI_3.1 |
| LIS210921 | Wastewater | 2021 | Lisburn | B | GB5.0.5a | RSV.B.NI_3.1 |
| NDN281122 | Wastewater | 2022 | North Down | A | GA2.3.5 | RSV.A.NI_1.2 |
| WTH071122 | Wastewater | 2022 | Whitehouse | A | GA2.3.5 | RSV.A.NI_1.1 |
| AMH031022 | Wastewater | 2022 | Armagh | B | GB5.0.5a | RSV.B.NI_1 |
| ATM101022 | Wastewater | 2022 | Antrim | B | GB5.0.5a | RSV.B.NI_1 |
| BNB290922 | Wastewater | 2022 | Banbridge | B | GB5.0.5a | RSV.B.NI_1 |
| CRG290922 | Wastewater | 2022 | Craigavon | B | GB5.0.5a | RSV.B.NI_1 |
| CRG311022 | Wastewater | 2022 | Craigavon | B | GB5.0.5a | RSV.B.NI_1 |
| DNG311022 | Wastewater | 2022 | Dungannon | B | GB5.0.5a | RSV.B.NI_1 |
| LIS101022 | Wastewater | 2022 | Lisburn | B | GB5.0.5a | RSV.B.NI_3.2 |
| LIS150822 | Wastewater | 2022 | Lisburn | B | GB5.0.5a | RSV.B.NI_1 |
| LIS311022 | Wastewater | 2022 | Lisburn | B | GB5.0.5a | RSV.B.NI_1 |
| NCT120922 | Wastewater | 2022 | North Coast | B | GB5.0.5a | RSV.B.NI_1 |
| NCT311022 | Wastewater | 2022 | North Coast | B | GB5.0.5a | RSV.B.NI_1 |
| NDN031022 | Wastewater | 2022 | North Down | B | GB5.0.5a | RSV.B.NI_2 |
| V22034499 | Clinical | 2022 |  | A | GA2.3.5 | RSV.A.NI_1.1 |
| V22034533 | Clinical | 2022 |  | A | GA2.3.5 | RSV.A.NI_1.1 |
| V22033750 | Clinical | 2022 |  | B | GB5.0.5a | RSV.B.NI_1 |
| V22034570 | Clinical | 2022 |  | B | GB5.0.5a | RSV.B.NI_3.2 |
| V22034901 | Clinical | 2022 |  | B | GB5.0.5a | RSV.B.NI_1 |
| V22035143 | Clinical | 2022 |  | B | GB5.0.5a | RSV.B.NI_1 |


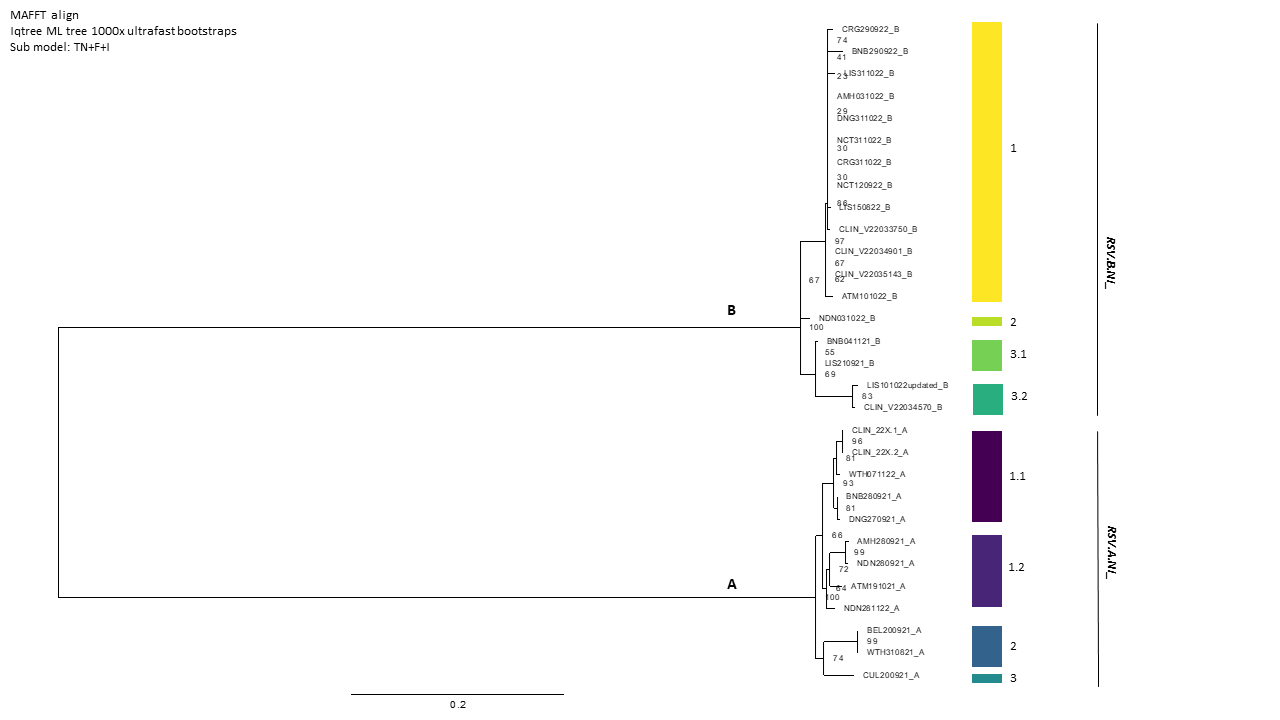


**Figure S5:** Maximum likelihood phylogenetic trees of generated NI RSV A and B based on G gene sequences with 1000 bootstrap replicates using IQtree

**References**

1. Schrader C, Schielke A, Ellerbroek L, Johne R. PCR inhibitors - occurrence, properties and removal. J Appl Microbiol. 2012. p. 1014–1026.

2. Villenave R, O’Donoghue D, Thavagnanam S, et al. Differential cytopathogenesis of respiratory syncytial virus prototypic and clinical isolates in primary pediatric bronchial epithelial cells. Virol J. BioMed Central Ltd; **2011**; 8(43).

3. Olesen SW, Imakaev M, Duvallet C. Making waves: Defining the lead time of wastewater-based epidemiology for COVID-19. Water Res. Elsevier Ltd; 2021.
